# Global Burden, Trends, and Inequalities of *Clostridioides difficile* Infections from 1990 to 2021 and Projections to 2040: A Systematic Analysis

**DOI:** 10.3390/antibiotics14070652

**Published:** 2025-06-27

**Authors:** Zhihui Chen, Jing Wu, Xiangru Ye, Jialin Jin, Wenhong Zhang

**Affiliations:** 1 Shanghai Key Laboratory of Infectious Diseases and Biosafety Emergency Response, Department of Infectious Diseases, National Medical Center for Infectious Diseases, Huashan Hospital, Shanghai Medical College, Fudan University, Shanghai 200040, China; 2 Shanghai Institute of Infectious Disease and Biosecurity, Fudan University, Shanghai 200030, China; 3 Department of Neurocritical Care Unit, Huashan Hospital, Shanghai Medical College, Fudan University, Shanghai 200437, China; 4 Shanghai Sci-Tech Inno Center for Infection & Immunity, Shanghai 200052, China

**Keywords:** *Clostridioides difficile* infection, global burden of disease, health inequality, epidemiology

## Abstract

Background: *Clostridioides difficile* infection (CDI) poses substantial clinical and economic challenges worldwide. This study aimed to evaluate the global burden, trends, and inequalities of CDI from 1990 to 2021, with projections extending to 2040. Methods: We conducted a systematic analysis of the Global Burden of Disease Study 2021 data for 204 countries and territories. CDI-related mortality and disability-adjusted life years (DALYs) were analyzed from 1990 to 2021. Joinpoint regression assessed the trends, a decomposition analysis identified the contributing factors, and cross-country inequalities were measured with slope and concentration indices. A log-linear age–period–cohort model projected future burden to 2040. Results: Global CDI-related deaths increased from 3047 (95% uncertainty interval [UI], 2550–3609) in 1990 to 15,598 (95% UI, 13,418–18,222) in 2021. The age-standardized mortality rate rose from 0.10 to 0.19/100,000 population (average annual percent change [AAPC], 2.26%; 95% confidence interval [CI], 1.77–2.76%), and the age-standardized DALY rate increased from 1.83 to 3.46/100,000 (AAPC, 1.94%; 95% CI, 1.43–2.45%). Epidemiological changes were the primary driver of this burden, contributing 45.46%. Inequalities were intensified, particularly in high sociodemographic index countries, evidenced by increases in the slope index from 2.00 to 4.17 and concentration index from 0.52 to 0.69. The projections suggest that mortality and DALY rates among populations aged ≥80 years will continue to rise through 2040. Conclusions: The global CDI burden has increased significantly over three decades, disproportionately affecting high sociodemographic index countries. The projected rise in CDI burden among older adults through 2040 underscores the urgent need for targeted interventions and strategic planning.

## 1. Introduction

*Clostridioides difficile* infection (CDI) is a daunting health care challenge, causing illness symptoms ranging from mild diarrhea to life-threatening complications such as pseudomembranous colitis, toxic megacolon, and sepsis [1,2,3]. The hospital-associated CDI incidence varies globally (5.3–8.3/10,000 patient days) [4,5,6], with mortality rates of 7–8.9% [6,7,8]. The economic burden of CDI is substantial, up to USD 34,157/patient in the United States (US) [9]. Additionally, health care-associated, community-acquired CDI is increasing [10,11], further complicating its epidemiology and management.

Unlike many other diarrheal diseases, CDI significantly affects countries with advanced health care systems [3,6,7,8], highlighting the complex interplay of health care practices, antibiotic use, and demographics [12,13,14]. The evolution of CDI burden, influenced by changing epidemiological patterns and demographic shifts, presents ongoing challenges for health care systems worldwide [4,5,7,15,16].

The current understanding of the global CDI burden is constrained by several critical limitations. First, much of the available data are outdated, failing to reflect recent changes in CDI epidemiology [17]. Second, comprehensive trend analyses that examine long-term patterns across diverse geographical and demographic contexts are lacking [3,16,17,18]. Third, the geographic coverage of CDI studies remains limited, with a bias toward high-income countries, leaving considerable knowledge gaps about the CDI burden in low- and middle-income settings where diagnostic and surveillance capabilities may be limited [19,20]. Finally, data are scarce on future projections of the global CDI burden. These gaps hinder the development of effective global prevention and management strategies.

We conducted a systematic analysis using Global Burden of Disease (GBD) data for 2021. In our study, we comprehensively examined the CDI burden at the global, regional, and national levels, focusing on current status, temporal trends, and distribution disparities. Our approach included a descriptive analysis, a temporal trend analysis, an inequalities analysis, and future burden projections to provide a robust foundation for evidence-based CDI management strategies globally.

## 2. Results

### 2.1. Global Burden and Trends of CDI from 1990 to 2021

In 2021, CDI caused an estimated 15,598 deaths globally (95% UI, 13,418–18,222), increasing from 3047 deaths in 1990 (95% UI, 2550–3609). The ASMR/100,000 population rose from 0.10 (95% UI, 0.08–0.11) in 1990 to 0.19 (95% UI, 0.16–0.23) in 2021, with an AAPC of 2.26% (95% CI, 1.77–2.76) (Table 1). Concurrently, age-standardized DALY rates increased from 1.83 (95% UI, 1.53–2.18) to 3.46 (95% UI, 3.04–3.96)/100,000 population (AAPC 1.94%; 95% CI, 1.43–2.45) (Table S2).

**Figure 1:**
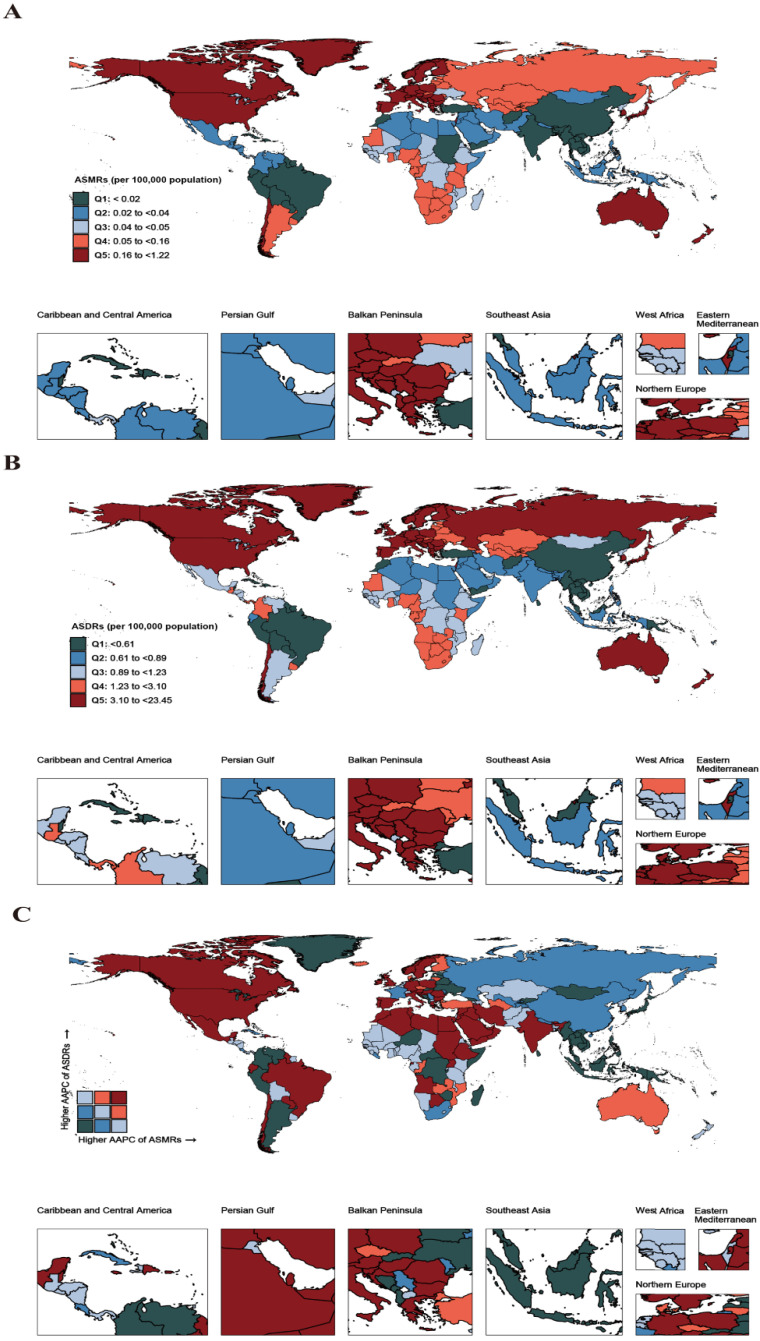
Burden and trends in CDI by country and territory. (**A**) ASMRs of CDI in 2021. (**B**) ASDRs of CDI in 2021. (**C**) AAPC in ASMRs and ASDRs attributable to CDI from 1990 to 2021. The base maps are from the Resource and Environment Science and Data Center, Chinese Academy of Sciences (https://www.resdc.cn/, accessed on 12 January 2025). Abbreviations: CDI, *Clostridioides difficile infection*; ASMRs, age-standardized mortality rates; ASDRs, age-standardized DALY rates; AAPC, average annual percent change; and DALY, disability-adjusted life year.

The CDI burden was concentrated in the elderly population, with both ASMRs and DALY rates increasing with age (Tables S3 and S4; Figure 1A–C). Significant increases in CDI-related ASMRs and DALY rates were observed across all age groups from 1990 to 2021, except in those under 5 years old (Table S5; Figure S1E,F). No significant sex differences were found in CDI burden trends over time (Table S5; Figure S1E,F).

When stratified by SDI quintiles, high-SDI regions experienced the highest CDI-related burden in 2021 (0.53 deaths/100,000; 95% UI, 0.47–0.61 and 10.7 DALYs/100,000; 95% UI, 9.81–11.7). Among the GBD regions, high-income North America had the highest age-standardized mortality (1.16 deaths/100,000; 95% UI, 1.04–1.27) and DALYs (5.39/100,000; 95% UI, 4.67–6.12) in 2021. This region also showed the fastest increase in CDI burden from 1990 to 2021 (AAPC of ASMRs: 6.03%; 95% UI, 5.41–6.66) (Table 1 and Table S2).

At the country level, the US had the highest CDI-related ASMRs (1.22 deaths/100,000; 95% UI, 1.09–1.33) and DALY rates (23.45/100,000; 95% UI, 21.81–24.85) in 2021 (Figure 1A,B). The US also experienced the fastest increase in ASMRs from 1990 to 2021 (AAPC: 6.26%; 95% UI, 5.55–6.98). In contrast, Mongolia showed the fastest decrease in ASMRs (AAPC: −1.87%; 95% UI, −2.34 to −1.39) and Ukraine had the fastest decrease in DALY rates (AAPC: −1.47%; 95% UI, −2.56 to −0.37) during this period (Figure 1C; Table S5).

### 2.2. Decomposition of Changes in the CDI Burden

**Figure 2:**
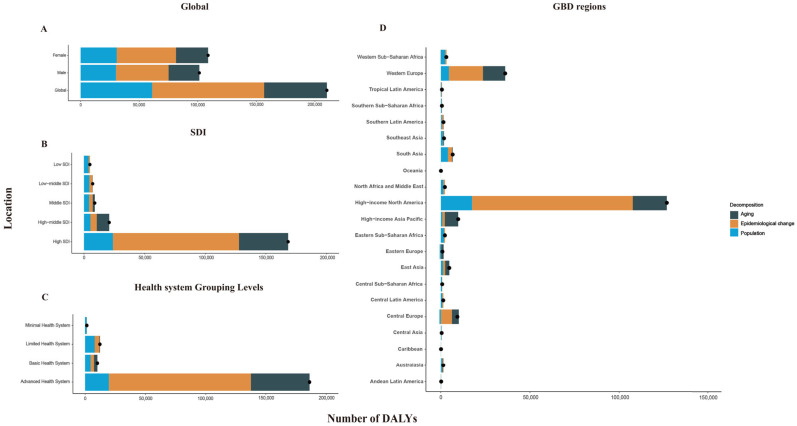
Decomposition of changes in the number of DALYs owing to CDI by region, 1990–2021. (**A**) Decomposition by sex. (**B**) Decomposition by SDI quintiles. (**C**) Decomposition by health system grouping level. (**D**) Decomposition by GBD region. The black dot indicates the total value of change generated by all three components. For each component, the magnitude of a positive value represents a corresponding increase in the number of DALYs caused by CDI attributable to that component; conversely, the magnitude of a negative value represents a corresponding decrease in the number of DALYs caused by CDI attributable to that component. Abbreviations: CDI, *Clostridioides difficile* infection; DALY, disability-adjusted life year; SDI, sociodemographic index; and GBD, Global Burden of Disease.

Using a decomposition analysis, we determined the relative contributions of epidemiological changes, population growth, and population aging to the CDI burden global increase (measured in DALYs) from 1990 to 2021. Globally, epidemiological changes were the largest contributor (45.46%); this was followed by population (29.06%) and population aging (25.48%). This contribution pattern remained largely consistent when stratified according to sex (Figure 2A; Table S7).

The analysis across SDI regions showed varying patterns. In high-SDI regions, epidemiological changes were the primary driver (61.61%), whereas in regions with high–middle SDI, population aging was the largest contributor (49.34%). Population growth was the dominant factor in middle- (46.95%), low–middle- (60.65%), and low-SDI (78.87%) regions (Figure 2B; Table S7). Similar trends were observed in the analysis of health system development. Epidemiological changes were the primary contributor in regions with advanced health systems (63.32%); population growth was the primary factor in areas with basic (45.38%), limited (64.28%), and minimal (90.34%) health systems (Figure 2C; Table S7).

The GBD regional analysis indicated that in most regions, the increase in CDI burden was primarily driven by population growth and epidemiological changes (Figure 2D). However, population aging was the largest contributor in Eastern Europe (158.71%), the high-income Asia Pacific region (77.46%), East Asia (48.58%), and Central Europe (41.63%) (Figure 2D; Table S7).

### 2.3. Inequality Analysis

**Figure 3:**
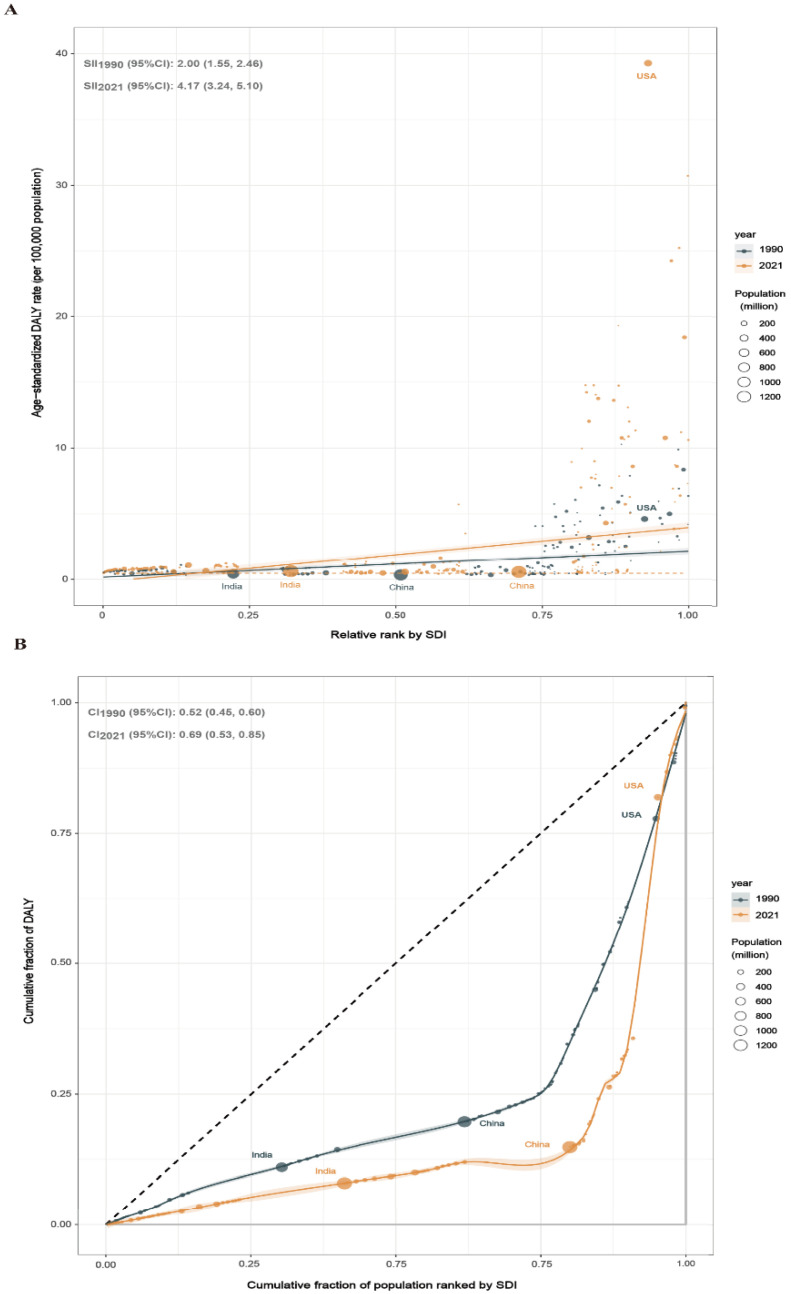
Health inequality regression curves (**A**) and concentration curves (**B**) for ASMRs of CDI. Abbreviations: CDI, *Clostridioides difficile* infection; DALY, disability-adjusted life year; and SDI, sociodemographic index.

The CDI burden presents absolute and relative inequalities associated with SDI, with high-SDI countries bearing a disproportionately higher burden. The slope inequality index (representing the difference in DALY rates between countries with the highest and lowest SDI) rose from 2.00 (95% CI, 1.55–2.46) in 1990 to 4.17 (95% CI, 3.24–5.10) in 2021 (Figure 3A). Additionally, the concentration index, a measure of relative inequality, increased from 0.52 (95% CI, 0.45–0.60) in 1990 to 0.69 (95% CI, 0.53–0.85) in 2021, indicating an increase in inequality in the CDI burden distribution across SDI levels (Figure 3B).

### 2.4. Projected Burden and Trends in CDI

**Figure 4:**
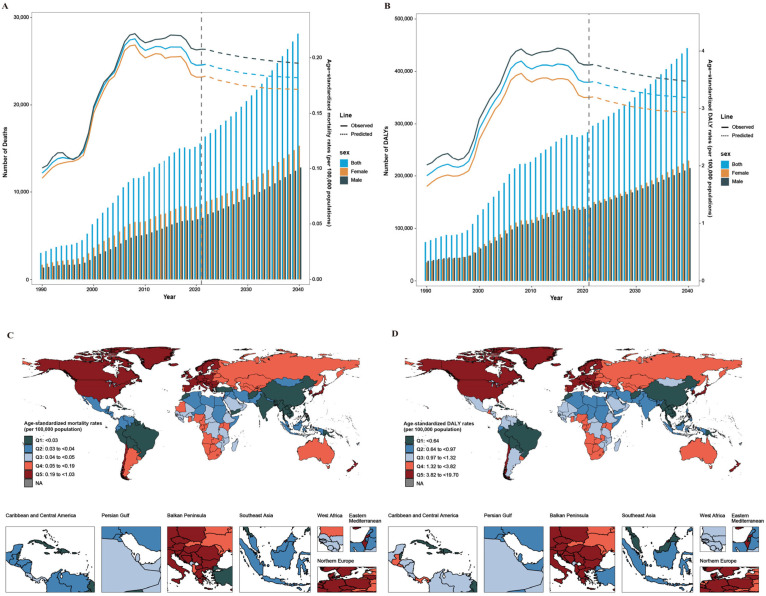
Change trends in ASMRs and ASDRs of CDI from 1990 to 2021, and predicted trends between 2022 and 2040. (**A**) Global change trends in ASMRs and predicted trends. (**B**) Global change trends in ASDRs and predicted trends. (**C**) Predicted ASMRs of CDI in 2040 in 194 countries and territories. (**D**) Predicted ASDRs of CDI in 2040 in 194 countries and territories. The base maps are from the Resource and Environment Science and Data Center, Chinese Academy of Sciences (https://www.resdc.cn/, accessed on 12 January 2025). Abbreviations: CDI, *Clostridioides difficile* infection; ASMR, age-standardized mortality rate; ASDR, age-standardized DALY rates; and DALY, disability-adjusted life year.

These projections are based on a historical trend marked by a significant inflection point around 2010, after which the upward trend of the global CDI burden was moderated. Globally, the number of deaths and DALYs owing to CDI are projected to continue to rise from 15,598 deaths and 284,051 DALYs in 2021 to 28,153 deaths and 444,401 DALYs by 2040 (Figure 4A,B; Table S8). Despite this increase in absolute numbers, ASMRs and DALYs are expected to decline slightly, from 0.19 deaths and 3.46 DALYs/100,000 population in 2022 to 0.18 deaths and 3.19 DALYs/100,000 population in 2040 (Figure 4A,B; Table S8). The overall trends are similar for both men and women (Table S8). Notably, mortality and DALY rates are anticipated to increase among populations aged ≥80 years over the next two decades (Table S9).

At the level of the GBD region, most are projected to experience rising ASMRs and ASDRs between 2022 and 2040, except for high-income Asia Pacific, high-income North America, and Oceania, where declines are expected (Table S10). At the national level, although ASMRs and ASDRs are projected to decrease in the US, that country will continue to have the highest CDI burden through 2040 (Table S11).

## 3. Discussion

The present study provides the latest data on CDI-related mortality and DALYs from 1990 to 2021 at the global, regional, and national levels, and ours is the first study to forecast the burden of CDI through 2040. Although CDI-related mortality and DALYs vary according to region and country, the overall global burden has increased over the past three decades. The decomposition analysis revealed that epidemiological changes are the primary drivers of this increase, followed by population growth and aging, and regional variations exist in the impact of these factors. Our analyses of global inequalities highlighted that high-SDI countries bear a disproportionately high burden of CDI, and these inequalities have intensified over time. Notably, while age-standardized mortality and DALY rates are expected to decrease slightly from 2022 to 2040, the absolute number of cases is projected to continue rising. This finding indicates that controlling and managing CDI will remain a considerable challenge in the coming decades.

The present findings reveal a substantial increase in CDI-related mortality and DALYs worldwide over this period. This rising trend is aligned with previous regional studies reporting increases in CDI incidence and severity [7,15,16], but our finding offers a broader global perspective. Notably, the burden was highest in high-SDI regions and concentrated among older adults, consistent with known risk factors for CDI [3,17,21]. Marked geographic variations, with North America experiencing the fastest increase and highest burden, highlight the complex interplay of factors influencing the epidemiology of CDI, including health care practices, antibiotic use patterns, and population demographics [7,18,22,23]. These disparities underscore the need for targeted prevention and control strategies tailored to specific regional contexts.

Our findings differ substantially from several previous studies in terms of specific numbers. Our study results showed that globally, there was an increase in CDI-related deaths from 3047 in 1990 to 15,598 in 2021. This contrasts sharply with the estimate of Guh et al. that CDI was associated with approximately 29,000 deaths in the US in 2011 alone [7]. These discrepancies likely arise from differences in the study methods and data sources. Methodological differences are a key factor, with the GBD study attributing each death to a single underlying cause, which may affect assessments of the role of CDI [24,25]. The complexity of attributing causes of death also has a crucial role because CDI may contribute significantly to mortality without being recorded as the primary cause [25,26,27]. Additionally, variations in CDI detection, monitoring, and reporting practices across countries may affect the outcomes [3]. Finally, different definitions and classification criteria for CDI-related deaths across studies may further explain the discrepancies [25,28]. Understanding these differences is essential to accurately interpret the global burden of CDI, underscoring the need for more standardized methods and definitions in future research. Despite these variations, our study findings provide valuable insight into the global distribution and trends in CDI, especially within the context of limited comprehensive global data.

The results of our decomposition analysis offer new insights into the factors driving the increasing CDI burden. In the analysis, we quantified the relative contributions of epidemiological changes (45.46%), population growth (29.06%), and aging (25.48%) to the rise in CDI burden, providing a more nuanced understanding than in previous studies. Although earlier research has highlighted the impact of population aging and changes in health care practices [3,8,29], our analysis suggests that epidemiological factors have a more important role than previously emphasized in the literature. This finding is in alignment with recent clinical and epidemiological trends, such as widespread antibiotic resistance in CDI [30,31], the rising proportion of community-acquired CDI [18,23], and the emergence of hypervirulent strains such as PCR ribotype 027 [32,33]. The combined effect of these factors may explain the substantial increase in CDI burden, particularly the dominant role of epidemiological changes. In our analysis, we not only validated these clinical observations but also quantified their relative importance, offering a more comprehensive framework for understanding the epidemiological dynamics of CDI. Additionally, our findings highlight the importance of modifiable factors such as antibiotic use patterns and changes in health care practices, suggesting that interventions targeting these epidemiological factors could have a large impact on reducing the CDI burden. This integrated perspective is crucial for developing more targeted prevention and control strategies to address future challenges in CDI.

The disproportionate burden of CDI in high-SDI regions, particularly in North America, reflects the unique challenges posed by CDI to advanced health care systems [8,18,34,35]. This pattern contrasts with typical trends in infectious diseases, which often cause heavier burdens in lower-income regions [36]. In high-income countries, factors contributing to this disparity are likely to include more frequent use of broad-spectrum antibiotics [37] and an aging population [38]. Additionally, better diagnostic capabilities in these regions may lead to higher detection rates [7,8,16]. The emergence of hypervirulent strains, such as ribotype 027, has affected high-income countries in particular, further exacerbating this disparity [17,32]. These findings suggest that with health care system advances in lower-SDI countries, these may face an increasing CDI risk, emphasizing the need for proactive prevention strategies in developing health care systems.

The trend inflection around 2010 marks a major turning point in the battle against CDI, representing the moment when public health interventions successfully countered the crisis caused by the hypervirulent RT027 strain [18,39,40]. By implementing targeted antibiotic stewardship programs to restrict fluoroquinolones [41]. Our projections reveal a complex trajectory for the global CDI burden through 2040. While the absolute numbers of deaths and DALYs are expected to rise, age-standardized rates demonstrate a slight decline, reflecting the significant impact of demographic changes. The projected increase in mortality and DALY rates among populations aged ≥80 years is particularly concerning, highlighting the growing vulnerability of the oldest populations to CDI [42]. This trend underscores the need for targeted interventions in geriatric care settings [43]. The variations in regional projections, with some high-income regions expected to see declines while most other areas face increases, point to the potential effectiveness of current control measures in certain advanced health care systems [44,45]. However, despite projected decreases, the persistently high burden in the US emphasizes the ongoing challenge posed by CDI even in countries with a robust health care infrastructure. These projections underscore the urgent need for global strategies that address both the rising absolute burden of CDI and the specific challenges faced by different age groups and regions, particularly in the context of aging populations and evolving health care systems worldwide.

Our study has several key limitations. A geographic data bias toward high-income countries may limit the generalizability of our findings, and trend estimates for regions with sparse data or low case counts have high uncertainty. Crucially, the GBD’s “single underlying cause of death” methodology leads to an underestimation of the true burden, as CDI is often a contributing factor to mortality rather than the primary cause. Other factors include heterogeneity in diagnostic criteria and the inherent lag in GBD’s modeling. Future research should focus on enhancing global surveillance, standardizing criteria, and improving data collection in under-represented regions.

## 4. Materials and Methods

### 4.1. Data Source and Definitions

Our analysis was performed within the framework of GBD 2021 data [22]. Following the International Classification of Diseases (ICD) principles in the GBD 2021 study, each death is attributed to a single underlying cause [24]. Diarrheal diseases were mapped to the GBD cause list using specific ICD-10 and ICD-9 codes (Table S1) [24]. The burden attributable to CDI was estimated using the population attributable fraction within the GBD framework [25,26]. Details of the data inputs, processing, synthesis, and final models are available in GBD 2021 publications [24,46,47]. Herein, we quantified the CDI burden using deaths and DALYs, which provide an aggregate measure of years of life lost owing to disability and premature death.

Additionally, a sociodemographic index (SDI) was used to categorize locations worldwide [48]. The SDI index is a composite measure of income per capita, average educational attainment, and fertility rates, expressed on a scale of 0–1. Locations were classified into SDI quintiles: low, low–middle, middle, middle–high, and high.

### 4.2. Temporal Trends and Decomposition Analysis

To evaluate the trends in CDI from 1990 to 2021, we used joinpoint regression to calculate the average annual percent change (AAPC) in the age-standardized mortality rates (ASMRs) and age-standardized DALYs rates (ASDRs) [49]. This statistical method identifies points in time where a trend significantly changes direction by fitting a series of segmented regression lines to the data over the entire period. The AAPC provides a single summary value representing the average rate of change. An upward trend is present when both the AAPC and lower confidence interval (CI) limit are positive; a downward trend is observed when the AAPC and upper CI limit are negative. To further evaluate the relative contributions of demographic and epidemiological factors, we decomposed the trends in DALYs between 1990 and 2021 into three distinct components: population aging, population growth, and changes in age-specific rates.

We first used the decomposition methodology of Das Gupta to perform this analysis [50]. The total number of DALYs at each location was conceptualized with the following formula:

$${DALY}_{ay,py,ey}=\sum_{i=1}^{32}\left(a_{i,y}*p_{y}*e_{i,y}\right)$$

where

*a_i,y_* represents the proportion of the population in age category *i* for a given year *y*, corresponding to population aging.
*p_y_* represents the total population in a given year *y*, corresponding to population growth.
*e_i,y_* represents the *DALY* rate for a given age category *i* in year *y*, corresponding to changes in age-specific rates (or epidemiologic changes).

The contribution of each of these three factors to the overall change in DALYs from 1990 to 2021 was then calculated by isolating the effect of one factor changing while the other two factors were held constant, following the Das Gupta formula.

### 4.3. Cross-Country Inequality Analysis

To quantify the distributive inequality of the CDI burden across countries, we applied two standard metrics: the slope index of inequality and the concentration index, which measure absolute and relative gradient inequality, respectively [51]. The slope index of inequality was derived by regressing national DALYs rates for the all-ages population on an SDI-associated relative position scale, defined as the midpoint of the cumulative population range and ranked according to SDI. We accounted for heteroskedasticity using a weighted regression model. The concentration index was calculated by numerically integrating the area under the Lorenz concentration curve [52,53], constructed using the cumulative fraction of DALYs and cumulative relative distribution of the population ranked by SDI.

### 4.4. Projection Analysis

To predict the CDI burden from 2022 to 2040 by country, sex, and age, we used a log-linear age–period–cohort model that smooths exponential growth and limits linear trend projection [54]. Based on the available data, we extrapolated the trends using the most recent four to six 5-year observation periods. A power function was used to smooth growth, and the recent 10-year linear trend was attenuated (or accentuated in the case of negative trends) by 25%, 50%, and 75% in the second, third, and fourth prediction periods, respectively. The number of new cases was predicted by averaging the projected rates for the last two prediction periods, followed by applying these rates to GBD national population forecasts [55] and world population age standard [48].

### 4.5. Statistical Analysis

The rates were expressed as the estimate/100,000 population and its 95% uncertainty interval (UI). Joinpoint regression was performed using the Joinpoint Regression Program, version 5.1.0.0 (NCI) [56]. The remaining analyses were performed using R version 4.3.2.

## 5. Conclusions

Our study provides the most comprehensive and up-to-date analysis of the global burden of CDI, offering crucial insights into its trends, determinants, and future projections. Our findings reveal a marked increase in the global CDI-related burden over the past three decades, largely driven by epidemiological changes. Notably, this burden is disproportionately concentrated in high-SDI countries, with widening inequalities among nations. Our study advances the understanding of CDI as a global health challenge, providing an international perspective that transcends regional views. By quantifying the evolving CDI burden and identifying key contributing factors, our research lays a critical foundation for informed decision-making in clinical practice, public health policy, and research prioritization. The projected increase in CDI-related burden among populations aged ≤80 years through 2040 underscores the urgent need for strategic planning and resource allocation. As CDI continues to pose a serious and growing threat to global health, insights from this study will be instrumental in developing targeted interventions and effective strategies to mitigate its impact and improve patient outcomes worldwide.

## Figures and Tables

**Table 1:** Death burden of *Clostridioides difficile* infection and their trends, 1990–2021, by sex and region.

	1990		2021		1990–2021 AAPC (95% CI), %
	Death Count (95% UIs)	ASMRs (95% UIs)	Death Count (95% UIs)	ASMRs (95% UIs)	
**Global**					
Males	1362 (1130 to 1639)	0.10 (0.08 to 0.12)	7060 (6068 to 8224)	0.21 (0.18 to 0.24)	2.36 (1.88 to 2.85)
Females	1685 (1406 to 1988)	0.09 (0.08 to 0.11)	8538 (7276 to 10056)	0.18 (0.16 to 0.21)	2.22 (1.69 to 2.75)
Both	3047 (2550 to 3609)	0.1 (0.08 to 0.11)	15598 (13418 to 18222)	0.19 (0.16 to 0.23)	2.26 (1.77 to 2.76)
**SDI quintiles**					
High SDI	1969 (1687 to 2299)	0.19 (0.16 to 0.22)	12378 (10799 to 14323)	0.53 (0.47 to 0.61)	3.27 (2.6 to 3.94)
High–Middle SDI	699 (586 to 832)	0.09 (0.07 to 0.1)	2119 (1695 to 2628)	0.11 (0.09 to 0.14)	1.01 (0.08 to 1.94)
Middle SDI	192 (127 to 279)	0.02 (0.01 to 0.03)	566 (378 to 842)	0.02 (0.02 to 0.03)	0.69 (0.53 to 0.84)
Low–Middle SDI	121 (75 to 175)	0.02 (0.01 to 0.03)	342 (226 to 495)	0.02 (0.02 to 0.04)	0.97 (0.66 to 1.28)
Low SDI	61 (38 to 89)	0.03 (0.02 to 0.04)	177 (118 to 255)	0.03 (0.02 to 0.05)	0.78 (0.65 to 0.9)
**Health system Grouping Levels**					
Advanced Health System	2583 (2213 to 2995)	0.17 (0.15 to 0.2)	14253 (12298 to 16600)	0.45 (0.4 to 0.52)	3.17 (2.69 to 3.66)
Basic Health System	269 (176 to 390)	0.02 (0.01 to 0.03)	751 (486 to 1120)	0.02 (0.02 to 0.03)	0.43 (0.14 to 0.72)
Limited Health System	171 (109 to 247)	0.02 (0.01 to 0.03)	528 (353 to 765)	0.03 (0.02 to 0.04)	0.91 (0.61 to 1.22)
Minimal Health System	19 (12 to 28)	0.03 (0.02 to 0.05)	51 (33 to 74)	0.04 (0.03 to 0.06)	0.67 (0.61 to 0.73)
**GBD regions**					
Andean Latin America	3 (2 to 4)	0.01 (0.01 to 0.02)	10 (6 to 13)	0.02 (0.01 to 0.02)	1.09 (0.33 to 1.86)
Australasia	39 (28 to 52)	0.17 (0.12 to 0.22)	130 (103 to 160)	0.22 (0.18 to 0.27)	1.05 (0.14 to 1.97)
Caribbean	3 (2 to 4)	0.01 (0.01 to 0.01)	6 (4 to 9)	0.01 (0.01 to 0.02)	0.8 (−0.06 to 1.67)
Central Asia	22 (16 to 31)	0.05 (0.04 to 0.07)	37 (28 to 48)	0.05 (0.04 to 0.07)	0.14 (−0.04 to 0.31)
Central Europe	221 (199 to 242)	0.18 (0.16 to 0.19)	812 (649 to 1030)	0.36 (0.29 to 0.45)	2.14 (0.87 to 3.43)
Central Latin America	25 (16 to 38)	0.02 (0.01 to 0.03)	78 (51 to 117)	0.03 (0.02 to 0.05)	1.28 (0.64 to 1.92)
Central Sub-Saharan Africa	8 (5 to 12)	0.04 (0.03 to 0.06)	26 (17 to 38)	0.05 (0.03 to 0.07)	0.65 (0.34 to 0.96)
East Asia	121 (74 to 183)	0.02 (0.01 to 0.03)	399 (254 to 609)	0.02 (0.01 to 0.03)	0.83 (0.25 to 1.41)
Eastern Europe	247 (195 to 313)	0.1 (0.08 to 0.13)	322 (275 to 376)	0.1 (0.08 to 0.11)	0.41 (−1.4 to 2.25)
Eastern Sub-Saharan Africa	28 (18 to 40)	0.04 (0.02 to 0.05)	82 (56 to 119)	0.05 (0.03 to 0.07)	0.85 (0.78 to 0.91)
High-Income Asia Pacific	328 (245 to 432)	0.19 (0.15 to 0.25)	1236 (911 to 1650)	0.22 (0.17 to 0.28)	0.48 (−0.16 to 1.12)
High-Income North America	646 (573 to 692)	0.18 (0.16 to 0.19)	8011 (7202 to 8860)	1.16 (1.04 to 1.27)	6.03 (5.41 to 6.66)
North Africa and Middle East	29 (18 to 43)	0.02 (0.01 to 0.02)	102 (67 to 150)	0.03 (0.02 to 0.04)	1.39 (1.08 to 1.7)
Oceania	1 (0 to 1)	0.02 (0.01 to 0.03)	2 (1 to 2)	0.03 (0.02 to 0.04)	0.33 (0.18 to 0.48)
South Asia	98 (60 to 147)	0.02 (0.01 to 0.02)	313 (203 to 464)	0.02 (0.01 to 0.03)	1.25 (0.86 to 1.64)
Southeast Asia	53 (34 to 79)	0.02 (0.01 to 0.03)	129 (81 to 198)	0.02 (0.01 to 0.03)	0.29 (0.1 to 0.47)
Southern Latin America	28 (19 to 39)	0.07 (0.05 to 0.09)	102 (79 to 132)	0.12 (0.09 to 0.15)	1.87 (0.27 to 3.49)
Southern Sub-Saharan Africa	16 (11 to 22)	0.06 (0.04 to 0.08)	35 (25 to 50)	0.07 (0.05 to 0.09)	0.55 (0.5 to 0.61)
Tropical Latin America	8 (5 to 12)	0.02 (0.02 to 0.03)	30 (20 to 43)	0.01 (0.01 to 0.02)	2.27 (1.77 to 2.77)
Western Europe	1085 (879 to 1318))	0.19 (0.16 to 0.23)	3628 (2813 to 4600)	0.33 (0.26 to 0.41)	1.77 (0.64 to 2.92)
Western Sub-Saharan Africa	36 (24 to 52)	0.04 (0.03 to 0.06)	107 (75 to 152)	0.05 (0.04 to 0.07)	0.79 (0.7 to 0.89)

Abbreviations: ASMRs, age-standardized mortality rates; UIs, uncertainty intervals; SDI, sociodemographic index; AAPC, average annual percent change; and CI, confidence interval.

## Data Availability

The data will be made available upon request.

## Supplementary Materials

The following supporting information can be downloaded at: https://www.mdpi.com/article/10.3390/antibiotics14070652/s1, Figure S1: Global age- and sex-specific CDI-related deaths and DALYs in 2021, and their AAPC from 1990 to 2021; Table S1: List of International Classification of Diseases (ICD) codes mapped to the Global Burden of Disease cause list for diarrheal diseases deaths; Table S2: DALYs burden of *Clostridioides difficile* infection and their trends, 1990–2021, by sex and region; Table S3: Global numbers and rates of deaths attributable to CDI by sex and age group in 2021; Table S4: Global numbers and rates of DALYs attributable to CDI by sex and age group in 2021; Table S5: AAPC in ASMRs and ASDRs attributable to *Clostridioides difficile* infection by sex and age group, 1990–2021; Table S6: Deaths and DALYs burden of *Clostridioides difficile* infection and their trends, 1990–2021, across 204 countries and territories; Table S7: Contribution of population aging, growth, and epidemiological changes to DALYs caused by CDI, 1990–2021; Table S8: The global trends and projections of deaths and DALYs of CDI between 2022 and 2040 by sex; Table S9: The global trends and projections of deaths and DALYs of CDI between 2022 and 2040 by age groups; Table S10: The global trends and projections of deaths and DALYs of CDI between 2022 and 2040 across 21 GBD regions; Table S11: The global trends and projections of deaths and DALYs of CDI between 2022 and 2040 across 194 countries and territories.
