# Supplementary material for: Global Burden, Trends, and Inequalities of Clostridioides difficile Infections from 1990 to 2021 and Projections to 2040: A Systematic Analysis
Source: Antibiotics (Basel). 2025 Jun 27;14(7):652. doi: 10.3390/antibiotics14070652 (PMC12291758; doi:10.3390/antibiotics14070652)
Supplement: Supplementary file 1 [file antibiotics-14-00652-s001.zip › antibiotics-3659459-supplementary.pdf]

Supplementary Online Content
Global Burden, Trends, and Inequalities of *Clostridioides difficile* Infections from 1990 to 2021 and Projections to 2040: A Systematic Analysis

Table S1: List of International Classification of Diseases (ICD) codes mapped to the Global Burden of Disease cause list for diarrheal diseases deaths..............................2

Table S2: DALYs burden of *Clostridioides difficile* infection and their trends, 1990-2021, by sex and region 4

Table S3: Global numbers and rates of deaths attributable to CDI by sex and age group in 2021 7

Table S4: Global numbers and rates of DALYs attributable to CDI by sex and age group in 2021..............................9

Table S5: AAPC in ASMRs and ASDRs attributable to *Clostridioides difficile* infection by sex and age group, 1990-2021.............................. 11

Table S6: Deaths and DALYs burden of *Clostridioides difficile* infection and their trends, 1990-2021, across 204 countries and territories.............................. 13

Table S7: Contribution of population aging, growth, and epidemiological changes to DALYs caused by CDI, 1990-2021..............................29

Table S8: The global trends and projections of deaths and DALYs of CDI between 2022 and 2040 by sex..............................31

Table S9: The global trends and projections of deaths and DALYs of CDI between 2022 and 2040 by age groups..............................34

Table S10: The global trends and projections of deaths and DALYs of CDI between 2022 and 2040 across 21 GBD regions..............................49

Table S11: The global trends and projections of deaths and DALYs of CDI between 2022 and 2040 across 194 countries and territories..............................64

Figure S1: Global age- and sex-specific CDI-related deaths and DALYs in 2021, and their AAPC from 1990 to 2021..............................196

Table S1: List of International Classification of Diseases (ICD) codes mapped to the Global Burden of Disease cause list for diarrheal diseases deaths.

Cause	ICD10	ICD9
Diarrheal diseases	A00-A00.9, A02-A02.0, A02.8-A07, A07.2-A07.4, A08-A09.9, K52.1-K52.3, R19.7	001-001.9, 003.8-006.9, 007.4-007.8, 008.2-009.9, 558.2-558.9

Table S2: DALYs burden of *Clostridioides difficile* infection and their trends, 1990-2021, by sex and region.

	**1990**			**2021**			**1990-2021 AAPC (95%CI), %**
	**DALYs count (95%UIs)**	**ASDRs (95%UIs)**		**DALYs count (95%UIs)**	**ASDRs (95%UIs)**		
**Global**							

Males	38438<br>(30315 to 46967)	2.01<br>(1.68 to 2.43)		139820<br>(120701 to 163402)	3.76<br>(3.28 to 4.35)		2.01<br>(1.63 to 2.39)
Females	35373<br>(29267 to 42330)	1.64<br>(1.39 to 1.95)		144230<br>(127340 to 164500)	3.19<br>(2.8 to 3.65)		2.01<br>(1.51 to 2.52)
Both	73811<br>(60130 to 89312)	1.83<br>(1.53 to 2.18)		284051<br>(249580 to 325982)	3.46<br>(3.04 to 3.96)		1.94<br>(1.43 to 2.45)
**SDI quintiles**							
High SDI	40192<br>(35272 to 46005)	4.12<br>(3.61 to 4.68)		208501<br>(189101 to 231914)	10.7<br>(9.81 to 11.7)		3<br>(2.5 to 3.51)
High-middle SDI	17034<br>(13858 to 20958)	1.83<br>(1.51 to 2.21)		37833<br>(30890 to 46437)	2.23<br>(1.84 to 2.72)		0.75<br>(-0.14 to 1.64)
Middle SDI	8037<br>(4824 to 12012)	0.56<br>(0.36 to 0.81)		16931<br>(10936 to 24646)	0.71<br>(0.47 to 1.01)		0.78<br>(0.63 to 0.93)
Low-middle SDI	5620<br>(3275 to 8510)	0.55<br>(0.34 to 0.81)		12794<br>(7973 to 18997)	0.75<br>(0.48 to 1.08)		0.94<br>(0.64 to 1.24)
Low SDI	2817<br>(1632 to 4170)	0.71<br>(0.44 to 1.02)		7708<br>(4693 to 11825)	0.89<br>(0.59 to 1.3)		0.77<br>(0.69 to 0.84)
**Health system Grouping Levels**							
Advanced Health System	54506<br>(47768 to 63080)	3.79<br>(3.31 to 4.36)		240684<br>(216052 to 269030)	9.11<br>(8.29 to 10.12)		2.73<br>(2.21 to 3.26)
Basic Health System	10397<br>(6293 to 15383)	0.55<br>(0.35 to 0.8)		20574<br>(13309 to 30155)	0.65<br>(0.43 to 0.94)		0.6<br>(0.45 to 0.76)
Limited Health System	7963<br>(4681 to 11941)	0.6<br>(0.38 to 0.87)		20235<br>(12651 to 30300)	0.81<br>(0.54 to 1.17)		0.96<br>(0.87 to 1.06)

Minimal Health System	833<br>(483 to 1244)	0.81<br>(0.52 to 1.18)		2273<br>(1352 to 3420)	0.98<br>(0.64 to 1.42)		0.59<br>(0.53 to 0.64)
**GBD regions**							
Andean Latin America	157<br>(90 to 245)	0.41<br>(0.25 to 0.63)		370<br>(225 to 529)	0.59<br>(0.36 to 0.84)		0.9<br>(0.19 to 1.61)
Australasia	761<br>(586 to 988)	3.34<br>(2.63 to 4.3)		2157<br>(1812 to 2532)	4.22<br>(3.57 to 4.87)		0.84<br>(-0.07 to 1.76)
Caribbean	147<br>(81 to 225)	0.41<br>(0.24 to 0.62)		234<br>(135 to 346)	0.52<br>(0.3 to 0.78)		0.92<br>(0.66 to 1.18)
Central Asia	716<br>(475 to 1005)	1.24<br>(0.86 to 1.76)		1161<br>(818 to 1630)	1.37<br>(1 to 1.88)		0.41<br>(0.27 to 0.56)
Central Europe	5247<br>(4703 to 5852)	4.08<br>(3.61 to 4.6)		14510<br>(11762 to 18120)	7.62<br>(6.3 to 9.39)		1.94<br>(1.38 to 2.5)
Central Latin America	1351<br>(804 to 2111)	0.83<br>(0.51 to 1.28)		2689<br>(1726 to 4210)	1.14<br>(0.73 to 1.75)		1.08<br>(0.99 to 1.17)
Central Sub-Saharan Africa	384<br>(223 to 591)	0.99<br>(0.66 to 1.42)		1134<br>(690 to 1722)	1.2<br>(0.81 to 1.73)		0.63<br>(0.51 to 0.74)
East Asia	4346<br>(2449 to 6620)	0.45<br>(0.28 to 0.67)		9079<br>(5763 to 13568)	0.54<br>(0.36 to 0.78)		0.67<br>(0.13 to 1.22)
Eastern Europe	6731<br>(5132 to 8974)	2.8<br>(2.13 to 3.69)		7611<br>(6318 to 9226)	2.84<br>(2.31 to 3.44)		0.47<br>(-1.04 to 2)
Eastern Sub-Saharan Africa	1254<br>(742 to 1862)	0.91<br>(0.6 to 1.31)		3522<br>(2159 to 5310)	1.18<br>(0.8 to 1.7)		0.86<br>(0.77 to 0.94)

High-income Asia Pacific	8871<br>(6676 to 11261)	5.2<br>(3.98 to 6.47)		18582<br>(14641 to 23682)	5.31<br>(4.39 to 6.47)		0.04<br>(-0.53 to 0.61)
High-income North America	13009<br>(12021 to 13860)	4.14<br>(3.79 to 4.47)		139788<br>(129438 to 149841)	22.36<br>(20.8 to 23.81)		5.39<br>(4.67 to 6.12)
North Africa and Middle East	1368<br>(759 to 2129)	0.47<br>(0.28 to 0.69)		3622<br>(2201 to 5419)	0.68<br>(0.44 to 0.98)		1.19<br>(1.01 to 1.36)
Oceania	29<br>(16 to 46)	0.6<br>(0.37 to 0.88)		67<br>(38 to 103)	0.65<br>(0.4 to 0.94)		0.22<br>(0.04 to 0.4)
South Asia	4786<br>(2715 to 7399)	0.49<br>(0.3 to 0.74)		11402<br>(7017 to 16834)	0.68<br>(0.44 to 0.99)		0.99<br>(0.63 to 1.35)
Southeast Asia	2130<br>(1242 to 3197)	0.58<br>(0.36 to 0.84)		3887<br>(2392 to 5739)	0.62<br>(0.4 to 0.91)		0.25<br>(0.07 to 0.43)
Southern Latin America	715<br>(511 to 985)	1.54<br>(1.11 to 2.1)		2162<br>(1707 to 2754)	2.73<br>(2.16 to 3.45)		1.84<br>(-0.04 to 3.76)
Southern Sub-Saharan Africa	593<br>(387 to 866)	1.47<br>(1.04 to 2.13)		1172<br>(822 to 1715)	1.75<br>(1.26 to 2.46)		0.56<br>(0.48 to 0.64)
Tropical Latin America	512<br>(315 to 758)	0.33<br>(0.21 to 0.47)		1091<br>(713 to 1561)	0.52(0.33 to 0.73)		1.55<br>(0.71 to 2.39)
Western Europe	19255<br>(16275 to 22536)	3.74<br>(3.26 to 4.29)		55302<br>(44726 to 67947)	6.4(5.39 to 7.65)		1.72<br>(0.55 to 2.91)
Western Sub-Saharan Africa	1451<br>(903 to 2150)	1.01<br>(0.68 to 1.45)		4510<br>(2812 to 6691)	1.3<br>(0.9 to 1.84)		0.81<br>(0.7 to 0.92)

Abbreviations: UIs, uncertainty intervals; SDI, sociodemographic index; CI, confidence interval; ASDRs, age-standardized DALY rates; AAPC, average annual percent change; and DALYs, disability-adjusted life years.

Table S3: Global numbers and rates of deaths attributable to CDI by sex and age group in 2021.

	Number (95% UI)				Rate (95% UI)		
**Age groups**	**Male**	**Female**	**Both**		**Male**	**Female**	**Both**
<5 years	66<br>(36 to 112)	33<br>(17 to 60)	100<br>(53 to 173)		0.02<br>(0.01 to 0.03)	0.01<br>(0.01 to 0.02)	0.02<br>(0.01 to 0.03)

5-9 years	36<br>(16 to 73)	40<br>(18 to 73)	76<br>(35 to 146)		0.01<br>(0 to 0.02)	0.01<br>(0.01 to 0.02)	0.01<br>(0.01 to 0.02)
10-14 years	20<br>(9 to 41)	35<br>(14 to 72)	55<br>(23 to 114)		0.01<br>(0 to 0.01)	0.01<br>(0 to 0.02)	0.01<br>(0 to 0.02)
15-19 years	21<br>(11 to 36)	17<br>(9 to 30)	38<br>(20 to 68)		0.01<br>(0 to 0.01)	0.01<br>(0 to 0.01)	0.01<br>(0 to 0.01)
20-24 years	26<br>(14 to 47)	14<br>(9 to 24)	40<br>(22 to 70)		0.01<br>(0 to 0.02)	0<br>(0 to 0.01)	0.01<br>(0 to 0.01)
25-29 years	36<br>(23 to 58)	20<br>(14 to 31)	56<br>(37 to 88)		0.01<br>(0.01 to 0.02)	0.01<br>(0 to 0.01)	0.01<br>(0.01 to 0.01)
30-34 years	50<br>(36 to 74)	29<br>(22 to 41)	79<br>(57 to 115)		0.02<br>(0.01 to 0.02)	0.01<br>(0.01 to 0.01)	0.01<br>(0.01 to 0.02)
35-39 years	63<br>(46 to 89)	38<br>(29 to 50)	101<br>(75 to 139)		0.02<br>(0.02 to 0.03)	0.01<br>(0.01 to 0.02)	0.02<br>(0.01 to 0.02)
40-44 years	81<br>(60 to 113)	51<br>(42 to 66)	132<br>(102 to 179)		0.03<br>(0.02 to 0.04)	0.02<br>(0.02 to 0.03)	0.03<br>(0.02 to 0.04)
45-49 years	104<br>(77 to 148)	74<br>(60 to 94)	178<br>(138 to 241)		0.04<br>(0.03 to 0.06)	0.03<br>(0.03 to 0.04)	0.04<br>(0.03 to 0.05)
50-54 years	158<br>(120 to 215)	115<br>(91 to 146)	273<br>(210 to 362)		0.07<br>(0.05 to 0.1)	0.05<br>(0.04 to 0.07)	0.06<br>(0.05 to 0.08)
55-59 years	214<br>(152 to 307)	166<br>(120 to 225)	380<br>(275 to 532)		0.11<br>(0.08 to 0.16)	0.08<br>(0.06 to 0.11)	0.1<br>(0.07 to 0.13)
60-64 years	384<br>(311 to 491)	344<br>(284 to 423)	729<br>(596 to 908)		0.25<br>(0.2 to 0.32)	0.21<br>(0.17 to 0.26)	0.23<br>(0.19 to 0.28)
65-69 years	645<br>(542 to 772)	611<br>(527 to 712)	1255<br>(1076 to 1477)		0.49<br>(0.41 to 0.59)	0.42<br>(0.37 to 0.49)	0.46<br>(0.39 to 0.54)
70-74 years	930<br>(774 to 1109)	972<br>(819 to 1143)	1902<br>(1598 to 2252)		0.96<br>(0.8 to 1.15)	0.89<br>(0.75 to 1.04)	0.92<br>(0.78 to 1.09)
75-79 years	1064	1182	2246		1.78	1.64	1.7

	(870 to 1324)	(971 to 1446)	(1839 to 2758)		(1.45 to 2.21)	(1.35 to 2.01)	(1.39 to 2.09)
80-84 years	1231<br>(982 to 1523)	1517<br>(1236 to 1856)	2749<br>(2243 to 3362)		3.36<br>(2.68 to 4.15)	2.98<br>(2.43 to 3.64)	3.14<br>(2.56 to 3.84)
85-89 years	1092<br>(848 to 1414)	1563<br>(1242 to 1990)	2655<br>(2091 to 3390)		6.33<br>(4.91 to 8.2)	5.49<br>(4.36 to 6.99)	5.81<br>(4.57 to 7.41)
90-94 years	610<br>(423 to 865)	1138<br>(812 to 1594)	1748<br>(1234 to 2454)		10.46<br>(7.27 to 14.84)	9.44<br>(6.73 to 13.21)	9.77<br>(6.9 to 13.72)
95+ years	229<br>(138 to 364)	577<br>(357 to 904)	806<br>(494 to 1260)		15.15<br>(9.15 to 24.04)	14.65<br>(9.06 to 22.94)	14.79<br>(9.07 to 23.11)

Abbreviations: CDI, *Clostridioides difficile* infections.

Table S4: Global numbers and rates of DALYs attributable to CDI by sex and age group in 2021.

	**Number (95% UIs)**				**Rate (95% UIs)**		
**Age groups**	**Male**	**Female**	**Both**		**Male**	**Female**	**Both**
<5 years	5833<br>(3140 to 9881)	2951<br>(1532 to 5244)	8784<br>(4664 to 15211)		1.72<br>(0.92 to 2.91)	0.93<br>(0.48 to 1.65)	1.33<br>(0.71 to 2.31)
5-9 years	3003<br>(1363 to 6021)	3286<br>(1516 to 6057)	6289<br>(2899 to 12109)		0.85<br>(0.38 to 1.7)	0.99<br>(0.46 to 1.82)	0.92<br>(0.42 to 1.76)
10-14 years	1581<br>(686 to 3207)	2721<br>(1130 to 5582)	4302<br>(1825 to 8868)		0.46<br>(0.2 to 0.93)	0.84<br>(0.35 to 1.73)	0.65<br>(0.27 to 1.33)

15-19 years	1528<br>(793 to 2663)	1285<br>(651 to 2199)	2813<br>(1436 to 4952)		0.48<br>(0.25 to 0.83)	0.42<br>(0.21 to 0.72)	0.45<br>(0.23 to 0.79)
20-24 years	1753<br>(926 to 3184)	1000<br>(594 to 1674)	2753<br>(1512 to 4805)		0.58<br>(0.31 to 1.05)	0.34<br>(0.2 to 0.57)	0.46<br>(0.25 to 0.8)
25-29 years	2241<br>(1451 to 3671)	1295<br>(886 to 1979)	3536<br>(2346 to 5561)		0.75<br>(0.49 to 1.23)	0.45<br>(0.3 to 0.68)	0.6<br>(0.4 to 0.95)
30-34 years	2921<br>(2076 to 4300)	1704<br>(1272 to 2368)	4625<br>(3345 to 6666)		0.96<br>(0.68 to 1.41)	0.57<br>(0.43 to 0.79)	0.77<br>(0.55 to 1.1)
35-39 years	3348<br>(2440 to 4744)	2024<br>(1580 to 2676)	5372<br>(4037 to 7369)		1.18<br>(0.86 to 1.68)	0.73<br>(0.57 to 0.96)	0.96<br>(0.72 to 1.31)
40-44 years	3878<br>(2896 to 5429)	2487<br>(2021 to 3206)	6365<br>(4926 to 8638)		1.54<br>(1.15 to 2.15)	1<br>(0.81 to 1.29)	1.27<br>(0.98 to 1.73)
45-49 years	4507<br>(3345 to 6372)	3212<br>(2618 to 4063)	7719<br>(5993 to 10436)		1.89<br>(1.41 to 2.68)	1.36<br>(1.11 to 1.72)	1.63<br>(1.27 to 2.2)
50-54 years	6061<br>(4593 to 8227)	4441<br>(3512 to 5635)	10502<br>(8081 to 13891)		2.73<br>(2.07 to 3.71)	1.99<br>(1.58 to 2.53)	2.36<br>(1.82 to 3.12)
55-59 years	7198<br>(5118 to 10297)	5612<br>(4062 to 7577)	12810<br>(9264 to 17905)		3.7<br>(2.63 to 5.29)	2.79<br>(2.02 to 3.77)	3.24<br>(2.34 to 4.52)
60-64 years	11150<br>(9034 to 14202)	10009<br>(8254 to 12276)	21159<br>(17319 to 26343)		7.17<br>(5.81 to 9.13)	6.08<br>(5.02 to 7.46)	6.61<br>(5.41 to 8.23)
65-69 years	15761<br>(13252 to 18889)	14938<br>(12899 to 17428)	30698<br>(26308 to 36155)		11.95<br>(10.05 to 14.33)	10.37<br>(8.96 to 12.1)	11.13<br>(9.54 to 13.11)
70-74 years	18645<br>(15516 to 22217)	19492<br>(16425 to 22915)	38137<br>(32055 to 45141)		19.34<br>(16.1 to 23.05)	17.81<br>(15.01 to 20.94)	18.53<br>(15.57 to 21.93)
75-79 years	17041<br>(13929 to 21193)	18889<br>(15534 to 23099)	35929<br>(29444 to 44116)		28.5<br>(23.3 to 35.45)	26.2<br>(21.55 to 32.04)	27.24<br>(22.33 to 33.45)
80-84 years	15411<br>(12295 to 19040)	18937<br>(15446 to 23127)	34347<br>(28057 to 41999)		42.05<br>(33.54 to 51.95)	37.18<br>(30.33 to 45.41)	39.22<br>(32.03 to 47.95)

85-89 years	10840<br>(8418 to 14037)	15450<br>(12298 to 19691)	26290<br>(20725 to 33600)		62.83<br>(48.79 to 81.36)	54.27<br>(43.2 to 69.17)	57.5<br>(45.33 to 73.49)
90-94 years	5274<br>(3668 to 7467)	9839<br>(7022 to 13765)	15113<br>(10678 to 21196)		90.49<br>(62.94 to 128.12)	81.58<br>(58.22 to 114.13)	84.48<br>(59.69 to 118.48)
95+ years	1846<br>(1118 to 2925)	4660<br>(2887 to 7290)	6506<br>(4000 to 10156)		122.06<br>(73.97 to 193.47)	118.33<br>(73.31 to 185.09)	119.36<br>(73.4 to 186.33)

Abbreviations: DALY, disability-adjusted life-year; CDI, *Clostridioides difficile* infection; UIs, uncertainty intervals.

Table S5: AAPC in ASMRs and ASDRs attributable to *Clostridioides difficile Infections* by sex and age group, 1990-2021.

	**AAPC in ASMRs (95% UIs)**				**AAPC in ASDRs (95% UIs)**		
**Age groups**	**Male**	**Female**	**Both**		**Male**	**Female**	**Both**
<5 years	-0.02<br>(-0.25 to 0.2)	0.01<br>(-0.23 to 0.24)	-0.02<br>(-0.23 to 0.2)		-0.03<br>(-0.26 to 0.2)	-0.01<br>(-0.25 to 0.23)	-0.03<br>(-0.25 to 0.19)

5-9 years	0.2<br>(-0.15 to 0.54)	0.39<br>(0.14 to 0.63)	0.29<br>(0.01 to 0.56)		0.18<br>(-0.16 to 0.53)	0.38<br>(0.13 to 0.62)	0.27<br>(0 to 0.55)
10-14 years	0.37<br>(0.05 to 0.7)	0.53<br>(0.16 to 0.91)	0.47<br>(0.19 to 0.74)		0.36<br>(0.04 to 0.68)	0.52<br>(0.14 to 0.91)	0.45<br>(0.17 to 0.73)
15-19 years	0.42<br>(-0.05 to 0.89)	0.72<br>(0.44 to 1)	0.54<br>(0.17 to 0.9)		0.41<br>(-0.06 to 0.88)	0.71<br>(0.41 to 1)	0.53<br>(0.16 to 0.89)
20-24 years	0.67<br>(0.32 to 1.02)	1.24<br>(0.91 to 1.57)	0.87<br>(0.52 to 1.22)		0.66<br>(0.3 to 1.01)	1.3<br>(0.92 to 1.68)	0.85<br>(0.49 to 1.21)
25-29 years	0.75<br>(0.48 to 1.02)	1.28<br>(0.97 to 1.6)	0.95<br>(0.74 to 1.16)		0.73<br>(0.47 to 0.99)	1.23<br>(0.92 to 1.55)	0.93<br>(0.73 to 1.13)
30-34 years	0.75<br>(0.35 to 1.14)	1.24<br>(0.92 to 1.56)	0.89<br>(0.65 to 1.14)		0.73<br>(0.29 to 1.17)	1.21<br>(0.89 to 1.52)	0.89<br>(0.64 to 1.13)
35-39 years	0.99<br>(0.56 to 1.42)	1.44<br>(0.98 to 1.89)	1.17<br>(0.75 to 1.59)		0.97<br>(0.55 to 1.4)	1.42<br>(1 to 1.84)	1.14<br>(0.72 to 1.56)
40-44 years	1.1<br>(0.57 to 1.63)	1.61<br>(1.09 to 2.12)	1.3<br>(0.84 to 1.75)		1.1<br>(0.67 to 1.54)	1.53<br>(0.97 to 2.09)	1.27<br>(0.83 to 1.71)
45-49 years	1.35<br>(0.82 to 1.87)	2.04<br>(1.71 to 2.36)	1.56<br>(1.09 to 2.04)		1.32<br>(0.8 to 1.84)	1.94<br>(1.56 to 2.32)	1.51<br>(1.04 to 1.98)
50-54 years	1.7<br>(1.29 to 2.1)	2.22<br>(1.52 to 2.93)	1.88<br>(1.26 to 2.5)		1.67<br>(1.27 to 2.08)	2.16<br>(1.41 to 2.9)	1.84<br>(1.18 to 2.51)
55-59 years	2.26<br>(1.63 to 2.89)	2.92<br>(1.96 to 3.9)	2.52<br>(1.92 to 3.12)		2.23<br>(1.6 to 2.86)	2.85<br>(1.82 to 3.88)	2.48<br>(1.84 to 3.12)
60-64 years	2.82<br>(2.1 to 3.56)	3.28<br>(2.62 to 3.95)	2.97<br>(2.39 to 3.54)		2.81<br>(2.09 to 3.53)	3.25<br>(2.57 to 3.92)	2.94<br>(2.36 to 3.53)
65-69 years	2.72<br>(1.86 to 3.58)	2.71<br>(2.04 to 3.38)	2.7<br>(1.85 to 3.57)		2.71<br>(1.88 to 3.54)	2.69<br>(2.01 to 3.39)	2.68<br>(1.85 to 3.52)

70-74 years	3.03<br>(2.28 to 3.78)	3.21<br>(2.4 to 4.02)	3.12<br>(2.33 to 3.92)		3.01<br>(2.3 to 3.73)	3.19<br>(2.41 to 3.97)	3.1<br>(2.36 to 3.85)
75-79 years	2.68<br>(2.28 to 3.09)	2.63<br>(2.13 to 3.14)	2.7<br>(2.26 to 3.15)		2.69<br>(2.29 to 3.08)	2.64<br>(2.15 to 3.14)	2.71<br>(2.27 to 3.15)
80-84 years	2.58<br>(2.22 to 2.95)	2.35<br>(1.8 to 2.9)	2.47<br>(1.98 to 2.96)		2.56<br>(2.18 to 2.93)	2.33<br>(1.78 to 2.88)	2.45<br>(1.96 to 2.94)
85-89 years	2.39<br>(1.36 to 3.44)	1.95<br>(1.24 to 2.67)	2.19<br>(1.48 to 2.91)		2.36<br>(1.33 to 3.39)	1.92<br>(1.21 to 2.63)	2.16<br>(1.45 to 2.87)
90-94 years	2.27<br>(1.41 to 3.13)	1.76<br>(0.92 to 2.6)	1.91<br>(1.11 to 2.73)		2.26<br>(1.41 to 3.12)	1.75<br>(0.92 to 2.59)	1.9<br>(1.1 to 2.71)
95+ years	2.12<br>(1.13 to 3.12)	1.45<br>(0.26 to 2.66)	1.63<br>(0.73 to 2.53)		2.05<br>(1 to 3.11)	1.42<br>(0.23 to 2.62)	1.58<br>(0.7 to 2.48)

Abbreviations: ASMRs, age-standardized mortality rates; ASDRs, age-standardized DALY rates; AAPC, average annual percent change; UIs, uncertainty intervals.

Table S6: Deaths and DALYs burden of *Clostridioides difficile* infection and their trends, 1990-2021, across 204 countries and territories.

	**Deaths (95% UIs)**				**1990-2021 AAPC (95%CI), %**	**DALYs (95% UIs)**				**1990-2021 AAPC (95%CI), %**
	**1990 counts**	**ASR (per 100 000), 1990**	**2021 counts**	**ASR (per 100 000), 2021**		**1990 counts**	**ASR (per 100 000), 1990**	**2021 counts**	**ASR (per 100 000), 2021**	

Afghanistan	1<br>(0 to 1)	0.01<br>(0.01 to 0.02)	2<br>(1 to 4)	0.02<br>(0.01 to 0.03)	0.81<br>(0.36 to 1.26)	41<br>(21 to 69)	0.42<br>(0.23 to 0.67)	141<br>(72 to 242)	0.51<br>(0.29 to 0.79)	0.8<br>(0.46 to 1.14)
Albania	4<br>(3 to 6)	0.24<br>(0.17 to 0.31)	8<br>(5 to 12)	0.21<br>(0.12 to 0.31)	-0.84<br>(-1.41 to -0.26)	135<br>(96 to 176)	5.4<br>(3.93 to 6.88)	153<br>(89 to 229)	4.33<br>(2.69 to 6.29)	-1.07<br>(-1.75 to -0.39)
Algeria	2<br>(1 to 3)	0.02<br>(0.01 to 0.03)	7<br>(5 to 11)	0.03<br>(0.02 to 0.04)	1.2<br>(1.1 to 1.3)	101<br>(56 to 160)	0.48<br>(0.3 to 0.7)	252<br>(155 to 391)	0.65<br>(0.42 to 0.97)	1.04<br>(0.97 to 1.12)
American Samoa	0<br>(0 to 0)	0.03<br>(0.02 to 0.05)	0<br>(0 to 0)	0.04<br>(0.02 to 0.05)	0.23<br>(0.16 to 0.3)	0<br>(0 to 0)	0.8<br>(0.51 to 1.16)	0<br>(0 to 1)	0.84<br>(0.56 to 1.21)	0.16<br>(0.09 to 0.23)
Andorra	0<br>(0 to 0)	0.1<br>(0.05 to 0.15)	0<br>(0 to 0)	0.08<br>(0.04 to 0.13)	-0.75<br>(-1.05 to -0.45)	1<br>(0 to 1)	1.68<br>(1.02 to 2.54)	2<br>(1 to 3)	1.25<br>(0.69 to 1.95)	-0.97<br>(-1.29 to -0.65)
Angola	1<br>(1 to 2)	0.04<br>(0.02 to 0.05)	6<br>(4 to 10)	0.05<br>(0.04 to 0.08)	1.09<br>(0.9 to 1.28)	68<br>(38 to 107)	0.95<br>(0.62 to 1.34)	306<br>(183 to 474)	1.34<br>(0.92 to 1.96)	1.16<br>(1.02 to 1.29)
Antigua and Barbuda	0<br>(0 to 0)	0.01<br>(0.01 to 0.02)	0<br>(0 to 0)	0.01<br>(0.01 to 0.02)	0.82<br>(-0.48 to 2.14)	0<br>(0 to 0)	0.4<br>(0.23 to 0.6)	0<br>(0 to 1)	0.49<br>(0.28 to 0.71)	0.74<br>(-0.59 to 2.08)
Argentina	13<br>(8 to 20)	0.05<br>(0.03 to 0.07)	31<br>(21 to 46)	0.05<br>(0.04 to 0.08)	0.55<br>(-1.05 to 2.18)	304<br>(194 to 452)	0.97<br>(0.63 to 1.42)	585<br>(403 to 815)	1.11<br>(0.77 to 1.54)	0.45<br>(-0.9 to 1.81)
Armenia	1<br>(0 to 1)	0.03<br>(0.02 to 0.04)	2<br>(1 to 2)	0.04<br>(0.03 to 0.05)	0.61<br>(-0.73 to 1.96)	18<br>(11 to 26)	0.64<br>(0.43 to 0.91)	30<br>(22 to 41)	0.83<br>(0.61 to 1.13)	0.76<br>(-0.76 to 2.29)
Australia	2<br>5(18 to 35)	0.13<br>(0.1 to 0.18)	90<br>(70 to 113)	0.18<br>(0.14 to 0.22)	1.27<br>(0.64 to 1.91)	491<br>(351 to 671)	2.57<br>(1.88 to 3.49)	1480<br>(1205 to 1775)	3.4<br>(2.82 to 4.02)	0.72<br>(-0.03 to 1.48)
Austria	15<br>(13 to 17)	0.13<br>(0.11 to 0.15)	78<br>(63 to 97)	0.35<br>(0.29 to 0.42)	2.96<br>(1.59 to 4.35)	255<br>(223 to 282)	2.37<br>(2.12 to 2.6)	1079<br>(917 to 1270)	5.71<br>(5 to 6.52)	2.79<br>(1.74 to 3.85)
Azerbaijan	2<br>(2 to 3)	0.05<br>(0.03 to 0.07)	5<br>(4 to 7)	0.06<br>(0.05 to 0.09)	0.79<br>(0.58 to 1)	73<br>(48 to 108)	1.22<br>(0.83 to 1.73)	140<br>(98 to 200)	1.55<br>(1.12 to 2.19)	0.78<br>(0.63 to 0.92)
Bahamas	0<br>(0 to 0)	0.01<br>(0.01 to 0.02)	0<br>(0 to 0)	0.01<br>(0.01 to 0.02)	0.42<br>(-0.68 to 1.53)	1<br>(1 to 2)	0.51<br>(0.29 to 0.79)	2<br>(1 to 3)	0.58<br>(0.34 to 0.86)	0.38<br>(-0.82 to 1.61)
Bahrain	0<br>(0 to 0)	0.02<br>(0.01 to 0.03)	0<br>(0 to 0)	0.03<br>(0.02 to 0.04)	1.18<br>(1.09 to 1.27)	2<br>(1 to 3)	0.54<br>(0.33 to 0.81)	9<br>(5 to 14)	0.74<br>(0.48 to 1.13)	0.96<br>(0.87 to 1.06)
Bangladesh	10	0.02	30	0.02	0.99	478	0.52	944	0.64	0.65

	(6 to 15)	(0.01 to 0.03)	(18 to 44)	(0.01 to 0.03)	(0.79 to 1.2)	(259 to 750)	(0.3 to 0.78)	(558 to 1423)	(0.39 to 0.95)	(0.41 to 0.89)
Barbados	0<br>(0 to 0)	0.01<br>(0.01 to 0.02)	0<br>(0 to 0)	0.01<br>(0.01 to 0.02)	0.61<br>(-0.36 to 1.59)	1<br>(1 to 2)	0.42<br>(0.25 to 0.63)	1<br>(1 to 2)	0.48<br>(0.28 to 0.71)	0.48<br>(-0.27 to 1.23)
Belarus	10<br>(8 to 12)	0.08<br>(0.07 to 0.1)	10<br>(8 to 12)	0.07<br>(0.05 to 0.08)	-0.67<br>(-1.96 to 0.63)	260<br>(204 to 352)	2.29<br>(1.78 to 3.1)	250<br>(196 to 312)	2.02<br>(1.61 to 2.5)	-0.1<br>(-2.23 to 2.08)
Belgium	34<br>(25 to 46)	0.23<br>(0.17 to 0.3)	78<br>(59 to 105)	0.29<br>(0.22 to 0.37)	0.84<br>(-0.24 to 1.94)	636<br>(475 to 826)	4.77<br>(3.59 to 6.04)	1248<br>(947 to 1625)	6.2<br>(4.71 to 7.99)	0.86<br>(-0.29 to 2.02)
Belize	0<br>(0 to 0)	0.01<br>(0 to 0.01)	0<br>(0 to 0)	0.01<br>(0.01 to 0.02)	1.19<br>(-0.02 to 2.41)	1<br>(0 to 1)	0.36<br>(0.19 to 0.56)	2<br>(1 to 3)	0.48<br>(0.27 to 0.71)	1.17<br>(0.12 to 2.22)
Benin	1<br>(1 to 1)	0.04<br>(0.02 to 0.05)	2<br>(2 to 3)	0.04<br>(0.03 to 0.06)	0.66<br>(0.61 to 0.7)	30<br>(18 to 45)	0.85<br>(0.56 to 1.25)	96<br>(58 to 138)	1.04<br>(0.69 to 1.49)	0.64<br>(0.43 to 0.84)
Bermuda	0<br>(0 to 0)	0.01<br>(0.01 to 0.02)	0<br>(0 to 0)	0.01<br>(0.01 to 0.02)	0.88<br>(-0.08 to 1.86)	0<br>(0 to 0)	0.39<br>(0.23 to 0.58)	0<br>(0 to 1)	0.47<br>(0.28 to 0.67)	0.74<br>(-0.18 to 1.67)
Bhutan	0<br>(0 to 0)	0.02<br>(0.01 to 0.02)	0<br>(0 to 0)	0.02<br>(0.01 to 0.04)	1.39<br>(1.25 to 1.52)	2<br>(1 to 4)	0.45<br>(0.27 to 0.68)	4<br>(3 to 6)	0.64<br>(0.39 to 0.93)	1.1<br>(0.84 to 1.36)
Bolivia (Plurination al State of)	1<br>(0 to 1)	0.01<br>(0.01 to 0.02)	1<br>(1 to 2)	0.01<br>(0.01 to 0.02)	0.74<br>(0.5 to 0.97)	30<br>(16 to 47)	0.44<br>(0.24 to 0.67)	62<br>(34 to 93)	0.55<br>(0.32 to 0.83)	0.71<br>(0.49 to 0.93)
Bosnia and Herzegovina	7<br>(6 to 10)	0.23<br>(0.17 to 0.3)	16<br>(7 to 26)	0.27<br>(0.12 to 0.42)	0.58<br>(0.41 to 0.75)	183<br>(138 to 237)	4.89<br>(3.78 to 6.28)	295<br>(139 to 460)	5.57<br>(2.74 to 8.38)	0.55<br>(0.35 to 0.74)
Botswana	0<br>(0 to 0)	0.05<br>(0.03 to 0.07)	1<br>(1 to 1)	0.07<br>(0.05 to 0.09)	1.08<br>(0.93 to 1.22)	11<br>(7 to 16)	1.2<br>(0.8 to 1.74)	32<br>(22 to 48)	1.72<br>(1.23 to 2.42)	1.16<br>(0.96 to 1.36)
Brazil	8<br>(5 to 11)	0.01<br>(0 to 0.01)	29<br>(19 to 42)	0.01<br>(0.01 to 0.02)	2.31<br>(1.8 to 2.82)	493<br>(305 to 734)	0.32<br>(0.21 to 0.47)	1053<br>(688 to 1508)	0.51<br>(0.33 to 0.73)	1.56<br>(0.7 to 2.43)
Brunei Darussalam	0<br>(0 to 0)	0.22<br>(0.17 to 0.29)	1<br>(1 to 1)	0.26<br>(0.2 to 0.33)	0.47<br>(0.31 to 0.62)	13<br>(10 to 17)	6.33<br>(4.85 to 7.82)	24<br>(17 to 32)	6.53<br>(5 to 8.19)	0.08<br>(-0.15 to 0.3)
Bulgaria	13<br>(11 to 15)	0.15<br>(0.14 to 0.17)	55<br>(42 to 70)	0.39<br>(0.3 to 0.49)	3.25<br>(2.19 to 4.33)	318<br>(282 to 360)	3.56<br>(3.11 to 4.15)	1002<br>(774 to 1243)	8.54<br>(6.88 to 10.39)	3.05<br>(1.99 to 4.11)

Burkina Faso	1<br>(1 to 2)	0.03<br>(0.02 to 0.05)	4<br>(3 to 5)	0.04<br>(0.03 to 0.06)	0.77<br>(0.48 to 1.07)	56<br>(33 to 87)	0.78<br>(0.51 to 1.15)	157<br>(92 to 233)	0.97<br>(0.64 to 1.4)	0.71<br>(0.59 to 0.83)
Burundi	1<br>(1 to 1)	0.04<br>(0.02 to 0.05)	2<br>(1 to 3)	0.04<br>(0.03 to 0.06)	0.42<br>(0.37 to 0.47)	37<br>(21 to 54)	0.89<br>(0.58 to 1.25)	91<br>(53 to 141)	1<br>(0.66 to 1.46)	0.38<br>(0.33 to 0.44)
Cabo Verde	0<br>(0 to 0)	0.03<br>(0.02 to 0.05)	0<br>(0 to 0)	0.05<br>(0.03 to 0.07)	1.39<br>(0.42 to 2.37)	2<br>(1 to 3)	0.8<br>(0.53 to 1.15)	6<br>(4 to 8)	1.18<br>(0.82 to 1.66)	1.56<br>(0.38 to 2.76)
Cambodia	1<br>(0 to 1)	0.02<br>(0.01 to 0.03)	2<br>(1 to 3)	0.02<br>(0.01 to 0.03)	0.2<br>(0 to 0.41)	40<br>(20 to 66)	0.47<br>(0.27 to 0.71)	70<br>(38 to 107)	0.49<br>(0.28 to 0.73)	0.16<br>(0.07 to 0.26)
Cameroon	2<br>(1 to 3)	0.04<br>(0.03 to 0.06)	7<br>(5 to 10)	0.05<br>(0.04 to 0.07)	0.77<br>(0.73 to 0.82)	77<br>(46 to 118)	1.04<br>(0.69 to 1.52)	291<br>(182 to 443)	1.33<br>(0.92 to 1.92)	0.75<br>(0.62 to 0.87)
Canada	74<br>(65 to 83)	0.24<br>(0.21 to 0.26)	534<br>(416 to 662)	0.69<br>(0.54 to 0.84)	3.78<br>(2.68 to 4.89)	1277<br>(1168 to 1382)	4.16<br>(3.79 to 4.53)	9096<br>(7237 to 10936)	13.39<br>(10.87 to 15.78)	3.89<br>(3.26 to 4.51)
Central African Republic	0<br>(0 to 1)	0.04<br>(0.02 to 0.06)	1<br>(1 to 1)	0.04<br>(0.03 to 0.06)	0.4<br>(0.37 to 0.42)	19<br>(11 to 28)	0.96<br>(0.61 to 1.42)	41<br>(24 to 61)	1.09<br>(0.72 to 1.58)	0.34<br>(0.31 to 0.38)
Chad	1<br>(1 to 1)	0.03<br>(0.02 to 0.05)	2<br>(2 to 4)	0.04<br>(0.03 to 0.06)	0.68<br>(0.63 to 0.73)	36<br>(21 to 54)	0.76<br>(0.48 to 1.13)	114<br>(65 to 174)	0.94<br>(0.61 to 1.33)	0.63<br>(0.55 to 0.7)
Chile	11<br>(8 to 15)	0.12<br>(0.09 to 0.15)	64<br>(52 to 81)	0.26<br>(0.21 to 0.32)	2.6<br>(1 to 4.22)	343<br>(249 to 468)	3.02<br>(2.25 to 4.03)	1457<br>(1179 to 1814)	6.55<br>(5.25 to 8.16)	2.71<br>(1.78 to 3.64)
China	108<br>(64 to 165)	0.02<br>(0.01 to 0.02)	340<br>(204 to 542)	0.02<br>(0.01 to 0.03)	0.63<br>(0.06 to 1.2)	3916<br>(2151 to 6080)	0.42<br>(0.25 to 0.63)	7918<br>(4767 to 12246)	0.5<br>(0.31 to 0.73)	0.55<br>(0.13 to 0.98)
Colombia	6<br>(4 to 9)	0.03<br>(0.02 to 0.04)	18<br>(12 to 26)	0.04<br>(0.02 to 0.05)	0.47<br>(0.31 to 0.63)	312<br>(186 to 465)	1.03<br>(0.66 to 1.5)	588<br>(393 to 836)	1.29<br>(0.85 to 1.81)	0.47<br>(0.3 to 0.65)
Comoros	0<br>(0 to 0)	0.04<br>(0.02 to 0.05)	0<br>(0 to 0)	0.05(0.03 to 0.07)	0.7<br>(-0.31 to 1.73)	3<br>(2 to 4)	0.9<br>(0.6 to 1.31)	7<br>(5 to 11)	1.19<br>(0.82 to 1.71)	0.77<br>(-0.79 to 2.36)
Congo	0<br>(0 to 1)	0.05<br>(0.03 to 0.07)	2<br>(1 to 2)	0.06<br>(0.04 to 0.08)	0.87<br>(0.64 to 1.1)	20<br>(12 to 32)	1.2<br>(0.8 to 1.76)	61<br>(41 to 91)	1.56<br>(1.13 to 2.17)	1.01<br>(0.6 to 1.41)

Cook Islands	0<br>(0 to 0)	0.03<br>(0.02 to 0.05)	0<br>(0 to 0)	0.04<br>(0.02 to 0.05)	0.58<br>(0.55 to 0.62)	0<br>(0 to 0)	0.72<br>(0.47 to 1.05)	0<br>(0 to 0)	0.84<br>(0.56 to 1.22)	0.51<br>(0.37 to 0.66)
Costa Rica	1<br>(0 to 1)	0.03<br>(0.02 to 0.04)	2<br>(1 to 3)	0.03<br>(0.02 to 0.05)	0.57<br>(-0.5 to 1.66)	26<br>(16 to 38)	0.93<br>(0.6 to 1.39)	52<br>(35 to 74)	1.14<br>(0.76 to 1.62)	0.8<br>(-0.07 to 1.68)
Croatia	10<br>(9 to 11)	0.2<br>(0.18 to 0.22)	35<br>(26 to 45)	0.37<br>(0.28 to 0.47)	2.21<br>(0.86 to 3.58)	215<br>(195 to 238)	4.22<br>(3.79 to 4.73)	591<br>(456 to 742)	7.62<br>(6.18 to 9.3)	1.91<br>(0.33 to 3.51)
Cuba	1<br>(1 to 1)	0.01<br>(0.01 to 0.01)	2<br>(1 to 3)	0.01<br>(0.01 to 0.02)	0.44<br>(-1.21 to 2.12)	37<br>(21 to 56)	0.37<br>(0.21 to 0.56)	47<br>(27 to 72)	0.41<br>(0.23 to 0.61)	0.6<br>(-1.29 to 2.52)
Cyprus	1<br>(1 to 2)	0.22<br>(0.17 to 0.29)	6<br>(4 to 8)	0.29<br>(0.23 to 0.38)	0.96<br>(0.67 to 1.24)	32<br>(24 to 44)	4.42<br>(3.44 to 5.89)	107<br>(82 to 142)	5.88<br>(4.69 to 7.66)	0.91<br>(0.79 to 1.04)
Czechia	35<br>(28 to 41)	0.27<br>(0.22 to 0.32)	87<br>(65 to 113)	0.39<br>(0.3 to 0.5)	0.62<br>(-2.3 to 3.62)	738<br>(590 to 880)	6.02<br>(4.88 to 7.29)	1567<br>(1186 to 2032)	8.6<br>(6.58 to 10.93)	1.22<br>(-0.86 to 3.34)
Côte d'Ivoire	2<br>(1 to 2)	0.04<br>(0.03 to 0.06)	6<br>(4 to 8)	0.05<br>(0.03 to 0.07)	0.73<br>(0.51 to 0.96)	78<br>(45 to 121)	0.98<br>(0.64 to 1.44)	233<br>(147 to 361)	1.23<br>(0.83 to 1.79)	0.75<br>(0.49 to 1.01)
Democratic People's Republic of Korea	4<br>(2 to 6)	0.03<br>(0.02 to 0.04)	11<br>(7 to 16)	0.04<br>(0.03 to 0.06)	0.67<br>(0.64 to 0.71)	122<br>(74 to 180)	0.74<br>(0.48 to 1.06)	247<br>(156 to 364)	0.89<br>(0.59 to 1.28)	0.59<br>(0.48 to 0.7)
Democratic Republic of the Congo	6<br>(4 to 8)	0.04<br>(0.03 to 0.06)	16<br>(10 to 23)	0.04<br>(0.03 to 0.06)	0.47<br>(0.01 to 0.94)	265<br>(153 to 410)	0.98<br>(0.64 to 1.41)	687<br>(416 to 1043)	1.11<br>(0.74 to 1.6)	0.37<br>(0.22 to 0.52)
Denmark	20<br>(15 to 27)	0.24<br>(0.18 to 0.31)	38<br>(29 to 51)	0.31<br>(0.24 to 0.39)	0.81<br>(0.05 to 1.58)	356<br>(275 to 465)	4.9<br>(3.83 to 6.2)	656<br>(509 to 855)	6.88<br>(5.5 to 8.46)	1.11<br>(-0.33 to 2.58)
Djibouti	0<br>(0 to 0)	0.04<br>(0.03 to 0.06)	0<br>(0 to 0)	0.05<br>(0.03 to 0.07)	0.72<br>(0.58 to 0.87)	3<br>(2 to 4)	0.95<br>(0.63 to 1.37)	10<br>(7 to 16)	1.18<br>(0.8 to 1.73)	0.72<br>(0.61 to 0.83)
Dominica	0<br>(0 to 0)	0.01<br>(0.01 to 0.01)	0<br>(0 to 0)	0.01(0.01 to 0.02)	1.03<br>(0.91 to 1.15)	0<br>(0 to 0)	0.42<br>(0.24 to 0.64)	0<br>(0 to 0)	0.56<br>(0.33 to 0.8)	0.92<br>(0.62 to 1.23)
Dominican Republic	0<br>(0 to 1)	0.01<br>(0 to 0.01)	1<br>(1 to 2)	0.01<br>(0.01 to 0.02)	1.04<br>(0.6 to 1.49)	30<br>(16 to 46)	0.38<br>(0.21 to 0.61)	52<br>(29 to 81)	0.5<br>(0.28 to 0.78)	0.95<br>(0.43 to 1.48)

Ecuador	1<br>(0 to 1)	0.01<br>(0 to 0.01)	4<br>(2 to 5)	0.02<br>(0.01 to 0.03)	3.02<br>(1.19 to 4.89)	32<br>(17 to 50)	0.32<br>(0.19 to 0.49)	129<br>(82 to 178)	0.75<br>(0.48 to 1.03)	2.55<br>(0.6 to 4.55)
Egypt	5<br>(3 to 7)	0.02<br>(0.01 to 0.03)	14<br>(9 to 21)	0.03<br>(0.02 to 0.04)	1.44<br>(1.34 to 1.55)	225<br>(120 to 362)	0.49<br>(0.3 to 0.75)	593<br>(346 to 923)	0.7<br>(0.44 to 1.05)	1.19<br>(1.06 to 1.32)
El Salvador	1<br>(1 to 1)	0.02<br>(0.02 to 0.04)	2<br>(1 to 3)	0.03<br>(0.02 to 0.05)	0.86<br>(0.81 to 0.91)	48<br>(29 to 71)	0.91<br>(0.56 to 1.28)	75<br>(50 to 106)	1.2<br>(0.8 to 1.72)	0.9<br>(0.85 to 0.96)
Equatorial Guinea	0<br>(0 to 0)	0.04<br>(0.02 to 0.05)	0<br>(0 to 1)	0.07<br>(0.05 to 0.09)	1.94<br>(1.74 to 2.14)	3<br>(2 to 5)	0.94<br>(0.63 to 1.38)	19<br>(12 to 28)	1.8<br>(1.31 to 2.51)	2.14<br>(1.92 to 2.36)
Eritrea	0<br>(0 to 1)	0.04<br>(0.02 to 0.05)	1<br>(1 to 2)	0.05<br>(0.03 to 0.07)	0.8<br>(0.67 to 0.94)	20<br>(11 to 31)	0.9<br>(0.59 to 1.3)	53<br>(33 to 83)	1.18<br>(0.81 to 1.72)	0.84<br>(0.62 to 1.06)
Estonia	2<br>(1 to 2)	0.09<br>(0.07 to 0.11)	2<br>(1 to 2)	0.06<br>(0.05 to 0.07)	-0.82<br>(-3 to 1.41)	41<br>(32 to 55)	2.33<br>(1.8 to 3.09)	32<br>(27 to 38)	1.65<br>(1.4 to 1.93)	-0.68<br>(-2.71 to 1.4)
Eswatini	0<br>(0 to 0)	0.05<br>(0.03 to 0.07)	0<br>(0 to 0)	0.06<br>(0.04 to 0.09)	0.85<br>(0.65 to 1.04)	7<br>(4 to 11)	1.24<br>(0.83 to 1.83)	14<br>(9 to 20)	1.63<br>(1.15 to 2.29)	0.88<br>(0.58 to 1.19)
Ethiopia	6<br>(4 to 9)	0.03<br>(0.02 to 0.05)	21<br>(14 to 30)	0.04<br>(0.03 to 0.06)	1<br>(0.83 to 1.17)	307<br>(178 to 464)	0.82<br>(0.54 to 1.18)	874<br>(543 to 1305)	1.12<br>(0.76 to 1.62)	0.98<br>(0.72 to 1.23)
Fiji	0<br>(0 to 0)	0.03<br>(0.02 to 0.04)	0<br>(0 to 0)	0.03<br>(0.02 to 0.05)	0.38<br>(0.14 to 0.62)	4<br>(2 to 6)	0.72<br>(0.45 to 1.07)	6<br>(4 to 9)	0.81<br>(0.53 to 1.2)	0.34<br>(0.24 to 0.43)
Finland	15<br>(11 to 19)	0.21<br>(0.16 to 0.27)	41<br>(31 to 54)	0.28<br>(0.21 to 0.36)	0.8<br>(-0.79 to 2.41)	256<br>(201 to 316)	3.85<br>(3.12 to 4.71)	628<br>(482 to 804)	5.44<br>(4.37 to 6.66)	0.9<br>5(-0.4 to 2.31)
France	186<br>(137 to 249)	0.22<br>(0.16 to 0.29)	468<br>(345 to 620)	0.27<br>(0.21 to 0.35)	0.47<br>(-0.97 to 1.94)	3410<br>(2567 to 4379)	4.53<br>(3.52 to 5.7)	7152<br>(5565 to 9091)	5.75<br>(4.57 to 7.19)	0.77<br>(-0.37 to 1.92)
Gabon	0<br>(0 to 0)	0.05<br>(0.03 to 0.07)	1<br>(0 to 1)	0.06<br>(0.04 to 0.09)	0.86<br>(0.7 to 1.02)	9<br>(6 to 13)	1.18<br>(0.82 to 1.72)	21<br>(14 to 32)	1.57<br>(1.1 to 2.22)	0.94<br>(0.69 to 1.19)
Gambia	0<br>(0 to 0)	0.04<br>(0.02 to 0.05)	0<br>(0 to 1)	0.05<br>(0.03 to 0.07)	0.76<br>(0.21 to 1.32)	6<br>(3 to 9)	0.88<br>(0.57 to 1.28)	18<br>(11 to 28)	1.11<br>(0.75 to 1.63)	0.83<br>(0.71 to 0.95)
Georgia	4<br>(3 to 6)	0.08<br>(0.05 to 0.1)	3<br>(3 to 4)	0.06<br>(0.05 to 0.07)	-0.84<br>(-2.46 to 0.81)	113<br>(80 to 162)	2.04<br>(1.44 to 2.84)	83<br>(67 to 102)	1.94<br>(1.58 to 2.35)	0<br>(-0.24 to 0.24)
Germany	369	0.29	992	0.45	1.58	6684	5.98	15712	9.06	1.56

	(289 to 461)	(0.23 to 0.36)	(769 to 1271)	(0.35 to 0.56)	(0.15 to 3.04)	(5509 to 7903)	(5.08 to 6.9)	(12565 to 19573)	(7.63 to 10.9)	(0.12 to 3.01)
Ghana	3 (2 to 4)	0.04 (0.03 to 0.06)	9 (6 to 13)	0.06 (0.04 to 0.08)	0.87 (0.75 to 0.98)	113 (70 to 168)	1.09 (0.75 to 1.55)	355 (231 to 520)	1.44 (1.01 to 2.05)	0.88 (0.77 to 1)
Greece	8 (7 to 9)	0.06 (0.05 to 0.07)	75 (63 to 85)	0.22 (0.18 to 0.24)	4.22 (3.38 to 5.05)	132 (120 to 143)	1.09 (0.97 to 1.21)	916 (787 to 1022)	3.35 (2.98 to 3.7)	3.63 (2.85 to 4.42)
Greenland	0 (0 to 0)	0.73 (0.59 to 0.9)	0 (0 to 1)	0.85 (0.57 to 1.11)	0.54 (0.44 to 0.64)	6 (4 to 7)	15.82 (12.58 to 19.46)	11 (7 to 14)	17.97 (11.48 to 23.29)	0.45 (0.35 to 0.55)
Grenada	0 (0 to 0)	0.01 (0 to 0.01)	0 (0 to 0)	0.01 (0.01 to 0.02)	1.48 (-0.55 to 3.56)	0 (0 to 1)	0.35 (0.2 to 0.56)	0 (0 to 1)	0.49 (0.29 to 0.74)	1.34 (-0.52 to 3.25)
Guam	0 (0 to 0)	0.03 (0.02 to 0.05)	0 (0 to 0)	0.04 (0.03 to 0.05)	0.27 (-0.35 to 0.89)	1 (1 to 1)	0.85 (0.56 to 1.22)	2 (1 to 2)	0.89 (0.61 to 1.28)	0.13 (-0.24 to 0.51)
Guatemala	1 (1 to 2)	0.02 (0.02 to 0.04)	4 (3 to 6)	0.03 (0.02 to 0.05)	0.86 (-0.31 to 2.05)	91 (52 to 143)	0.96 (0.59 to 1.41)	190 (117 to 283)	1.31 (0.83 to 1.9)	0.65 (-0.29 to 1.61)
Guinea	1 (1 to 2)	0.03 (0.02 to 0.05)	3 (2 to 4)	0.04 (0.03 to 0.06)	0.78 (0.63 to 0.94)	41 (24 to 60)	0.85 (0.53 to 1.26)	104 (65 to 156)	1.08 (0.72 to 1.54)	0.79 (0.56 to 1.03)
Guinea-Bissau	0 (0 to 0)	0.04 (0.02 to 0.05)	0 (0 to 1)	0.05 (0.03 to 0.07)	0.74 (0.04 to 1.45)	7 (4 to 10)	0.93 (0.62 to 1.36)	16 (9 to 24)	1.14 (0.77 to 1.67)	0.8 (-0.27 to 1.88)
Guyana	0 (0 to 0)	0.01 (0.01 to 0.01)	0 (0 to 0)	0.01 (0.01 to 0.02)	1.13 (-0.16 to 2.43)	3 (2 to 6)	0.42 (0.23 to 0.66)	4 (2 to 6)	0.56 (0.32 to 0.84)	0.96 (-0.63 to 2.58)
Haiti	1 (0 to 1)	0.01 (0 to 0.01)	1 (1 to 2)	0.01 (0.01 to 0.02)	0.69 (0.25 to 1.13)	37 (18 to 63)	0.47 (0.24 to 0.74)	74 (38 to 118)	0.55 (0.29 to 0.86)	0.62 (0.12 to 1.12)
Honduras	1 (0 to 1)	0.02 (0.01 to 0.03)	2 (1 to 3)	0.03 (0.02 to 0.04)	0.92 (0.81 to 1.03)	45 (27 to 72)	0.89 (0.53 to 1.33)	108 (66 to 157)	1.17 (0.76 to 1.68)	0.92 (0.84 to 1)
Hungary	28 (24 to 32)	0.22 (0.19 to 0.25)	78 (59 to 103)	0.39 (0.3 to 0.49)	1.94 (-0.77 to 4.72)	630 (532 to 740)	5.16 (4.33 to 6.08)	1417 (1096 to 1826)	8.63 (6.78 to 11.23)	1.79 (-0.08 to 3.69)
Iceland	0 (0 to 1)	0.14 (0.1 to 0.19)	1 (1 to 2)	0.2 (0.15 to 0.27)	0.97 (-0.01 to 1.97)	7 (6 to 9)	2.51 (1.93 to 3.2)	21 (16 to 27)	3.69 (2.85 to 4.64)	1.1 (-0.15 to 2.37)

India	72<br>(44 to 108)	0.01<br>(0.01 to 0.02)	242<br>(155 to 362)	0.02<br>(0.01 to 0.03)	1.42<br>(0.93 to 1.92)	3560<br>(2013 to 5523)	0.47<br>(0.28 to 0.7)	8709<br>(5335 to 13083)	0.67<br>(0.42 to 0.97)	1.17<br>(0.92 to 1.42)
Indonesia	21<br>(13 to 31)	0.02<br>(0.01 to 0.03)	47<br>(28 to 72)	0.02<br>(0.02 to 0.04)	0.31<br>(0.11 to 0.5)	898<br>(520 to 1365)	0.61<br>(0.38 to 0.9)	1578<br>(965 to 2379)	0.67<br>(0.42 to 0.99)	0.3<br>(0.1 to 0.51)
Iran (Islamic Republic of)	5<br>(3 to 8)	0.02<br>(0.01 to 0.03)	20<br>(13 to 29)	0.03<br>(0.02 to 0.04)	1.36<br>(1.15 to 1.58)	274<br>(155 to 428)	0.54<br>(0.33 to 0.8)	618<br>(390 to 931)	0.7<br>9(0.5 to 1.15)	1.11<br>(0.89 to 1.34)
Iraq	2<br>(1 to 2)	0.02<br>(0.01 to 0.02)	6<br>(4 to 8)	0.03<br>(0.02 to 0.04)	1.55<br>(1.26 to 1.84)	73<br>(39 to 117)	0.45<br>(0.27 to 0.69)	232<br>(135 to 361)	0.69<br>(0.44 to 1.01)	1.39<br>(1.12 to 1.65)
Ireland	7<br>(6 to 8)	0.18<br>(0.15 to 0.22)	22<br>(17 to 27)	0.26<br>(0.2 to 0.32)	1.28<br>(-0.2 to 2.79)	122<br>(104 to 138)	3.14<br>(2.71 to 3.58)	342<br>(276 to 415)	4.5<br>(3.74 to 5.38)	1.35<br>(0.01 to 2.7)
Israel	10<br>(7 to 13)	0.22<br>(0.16 to 0.29)	37<br>(28 to 49)	0.27<br>(0.21 to 0.36)	0.71<br>(-0.73 to 2.17)	215<br>(164 to 278)	4.45<br>(3.45 to 5.71)	670<br>(515 to 859)	5.61<br>(4.32 to 7.18)	0.86<br>(-0.72 to 2.47)
Italy	95<br>(85 to 103)	0.12<br>(0.11 to 0.13)	588<br>(454 to 749)	0.32<br>(0.25 to 0.41)	3.85<br>(1.55 to 6.21)	1650<br>(1512 to 1758)	2.29<br>(2.1 to 2.49)	8231<br>(6522 to 10290)	5.99<br>(4.88 to 7.28)	3.18<br>(0.36 to 6.09)
Jamaica	0<br>(0 to 0)	0.01<br>(0.01 to 0.01)	0<br>(0 to 0)	0.01<br>(0.01 to 0.02)	1.1<br>(0.82 to 1.37)	9<br>(5 to 14)	0.36<br>(0.2 to 0.56)	11<br>(7 to 17)	0.44<br>(0.26 to 0.65)	0.96<br>(0.7 to 1.21)
Japan	267<br>(192 to 361)	0.18<br>(0.14 to 0.24)	1007<br>(726 to 1350)	0.21<br>(0.16 to 0.27)	0.43<br>(-0.38 to 1.24)	6297<br>(4714 to 8179)	4.69<br>(3.59 to 5.91)	13755<br>(10576 to 17998)	4.73<br>(3.93 to 5.73)	0.01<br>(-0.79 to 0.82)
Jordan	0<br>(0 to 0)	0.02<br>(0.01 to 0.03)	2<br>(1 to 3)	0.03<br>(0.02 to 0.04)	1.21<br>(1.03 to 1.4)	14<br>(8 to 23)	0.49<br>(0.3 to 0.72)	66<br>(39 to 101)	0.68<br>(0.44 to 1.01)	1.06<br>(0.97 to 1.15)
Kazakhstan	6<br>(4 to 8)	0.05<br>(0.04 to 0.07)	9<br>(7 to 11)	0.06<br>(0.05 to 0.07)	0.73<br>(-0.52 to 1.99)	176<br>(118 to 260)	1.25<br>(0.87 to 1.81)	263<br>(188 to 383)	1.51<br>(1.13 to 2.11)	0.79<br>(-0.58 to 2.18)
Kenya	4<br>(3 to 6)	0.04<br>(0.03 to 0.06)	13<br>(9 to 18)	0.06<br>(0.04 to 0.08)	0.73<br>(0.65 to 0.81)	185<br>(112 to 276)	1.11<br>(0.76 to 1.61)	527<br>(344 to 778)	1.45<br>(1.02 to 2.05)	0.71<br>(0.61 to 0.81)
Kiribati	0<br>(0 to 0)	0.03<br>(0.02 to 0.04)	0<br>(0 to 0)	0.03<br>(0.02 to 0.04)	0.32<br>(0.2 to 0.45)	0<br>(0 to 1)	0.67<br>(0.42 to 0.99)	1<br>(0 to 1)	0.73<br>(0.45 to 1.06)	0.25<br>(0.03 to 0.47)

Kuwait	0<br>(0 to 0)	0.02<br>(0.01 to 0.03)	1<br>(1 to 1)	0.03<br>(0.02 to 0.04)	0.99<br>(-1.99 to 4.06)	7<br>(4 to 12)	0.57<br>(0.36 to 0.85)	28<br>(17 to 44)	0.76<br>(0.51 to 1.13)	0.72<br>(-2.24 to 3.76)
Kyrgyzstan	1<br>(1 to 2)	0.04<br>(0.03 to 0.06)	2<br>(2 to 3)	0.05<br>(0.04 to 0.06)	0.32<br>(-1.52 to 2.2)	40<br>(26 to 58)	1.07<br>(0.72 to 1.53)	70<br>(47 to 105)	1.24<br>(0.88 to 1.78)	0.43<br>(-1.2 to 2.09)
Lao People's Democratic Republic	0<br>(0 to 1)	0.02<br>(0.01 to 0.03)	1<br>(0 to 1)	0.02<br>(0.01 to 0.03)	0.43<br>(0.27 to 0.59)	17<br>(9 to 28)	0.48<br>(0.27 to 0.73)	32<br>(18 to 48)	0.54<br>(0.31 to 0.78)	0.34<br>(0.15 to 0.54)
Latvia	3<br>(2 to 3)	0.08<br>(0.07 to 0.1)	2<br>(2 to 2)	0.06<br>(0.05 to 0.07)	-0.69<br>(-1.8 to 0.44)	70<br>(55 to 96)	2.32<br>(1.8 to 3.11)	46<br>(39 to 55)	1.63<br>(1.36 to 1.93)	-0.83<br>(-3.4 to 1.81)
Lebanon	0<br>(0 to 0)	0.02<br>(0.01 to 0.02)	2<br>(1 to 2)	0.03<br>(0.02 to 0.04)	1.53<br>(1.43 to 1.62)	12<br>(7 to 18)	0.45<br>(0.28 to 0.65)	39<br>(25 to 57)	0.66<br>(0.44 to 0.97)	1.36<br>(1.23 to 1.5)
Lesotho	0<br>(0 to 1)	0.04<br>(0.03 to 0.06)	1<br>(0 to 1)	0.06<br>(0.04 to 0.08)	0.91<br>(0.7 to 1.11)	13<br>(8 to 19)	1.1<br>(0.73 to 1.58)	21<br>(14 to 32)	1.48<br>(1.03 to 2.12)	0.94<br>(0.69 to 1.19)
Liberia	0<br>(0 to 1)	0.04<br>(0.02 to 0.05)	1<br>(1 to 1)	0.04<br>(0.03 to 0.06)	0.62<br>(0.26 to 0.97)	16<br>(9 to 24)	0.89<br>(0.57 to 1.31)	40<br>(24 to 59)	1.07<br>(0.71 to 1.54)	0.58<br>(-0.09 to 1.25)
Libya	0<br>(0 to 1)	0.02<br>(0.01 to 0.03)	1<br>(1 to 2)	0.03<br>(0.02 to 0.04)	1.12<br>(1.02 to 1.23)	18<br>(10 to 29)	0.52<br>(0.32 to 0.76)	41<br>(25 to 62)	0.71<br>(0.46 to 1.04)	0.95<br>(0.57 to 1.34)
Lithuania	4<br>(3 to 5)	0.1<br>(0.08 to 0.12)	8<br>(6 to 9)	0.13<br>(0.1 to 0.15)	1.09<br>(-0.62 to 2.84)	99<br>(76 to 132)	2.47<br>(1.89 to 3.26)	138<br>(112 to 166)	2.94<br>(2.39 to 3.54)	0.82<br>(-1.32 to 3.01)
Luxembourg	1<br>(1 to 2)	0.23<br>(0.18 to 0.3)	3<br>(3 to 4)	0.29<br>(0.22 to 0.38)	0.89<br>(-0.75 to 2.55)	23<br>(18 to 29)	4.72<br>(3.79 to 5.92)	56<br>(44 to 73)	5.84<br>(4.62 to 7.49)	0.69<br>(-0.75 to 2.14)
Madagascar	2<br>(1 to 3)	0.04<br>(0.03 to 0.05)	5<br>(3 to 7)	0.05<br>(0.03 to 0.07)	0.56<br>(0.49 to 0.63)	82<br>(49 to 124)	0.94<br>(0.62 to 1.35)	21<br>6(131 to 340)	1.12<br>(0.76 to 1.64)	0.57<br>(0.36 to 0.79)
Malawi	1<br>(1 to 2)	0.04<br>(0.02 to 0.05)	4<br>(2 to 5)	0.05<br>(0.03 to 0.07)	0.85<br>(0.78 to 0.92)	66<br>(38 to 98)	0.92<br>(0.59 to 1.31)	161<br>(98 to 249)	1.19<br>(0.81 to 1.74)	0.85<br>(0.78 to 0.91)
Malaysia	2<br>(1 to 3)	0.02<br>(0.01 to 0.04)	6<br>(4 to 10)	0.03<br>(0.02 to 0.04)	0.3<br>(-0.03 to 0.63)	73<br>(43 to 110)	0.56<br>(0.35 to 0.81)	172<br>(107 to 252)	0.61<br>(0.39 to 0.89)	0.34<br>(-0.12 to 0.8)
Maldives	0<br>(0 to 0)	0.02<br>(0.01 to 0.03)	0<br>(0 to 0)	0.02<br>(0.01 to 0.03)	0.94<br>(0.76 to 1.12)	1<br>(0 to 1)	0.42<br>(0.25 to 0.62)	2<br>(1 to 3)	0.56<br>(0.34 to 0.81)	0.93<br>(0.79 to 1.08)

Mali	1<br>(1 to 2)	0.03<br>(0.02 to 0.05)	4<br>(2 to 5)	0.04<br>(0.03 to 0.06)	0.74<br>(0.69 to 0.8)	50<br>(29 to 76)	0.77<br>(0.49 to 1.14)	161<br>(92 to 240)	0.96<br>(0.62 to 1.39)	0.77<br>(0.73 to 0.82)
Malta	0<br>(0 to 1)	0.12<br>(0.11 to 0.14)	2<br>(2 to 3)	0.22<br>(0.18 to 0.27)	1.92<br>(0.79 to 3.07)	8<br>(7 to 9)	2.06<br>(1.86 to 2.27)	35<br>(29 to 42)	3.78<br>(3.26 to 4.39)	1.91<br>(0.71 to 3.14)
Marshall Islands	0<br>(0 to 0)	0.03<br>(0.02 to 0.04)	0<br>(0 to 0)	0.03<br>(0.02 to 0.04)	0.32<br>(-0.3 to 0.94)	0<br>(0 to 0)	0.64<br>(0.4 to 0.93)	0<br>(0 to 0)	0.73<br>(0.46 to 1.07)	0.16<br>(-0.91 to 1.24)
Mauritania	0<br>(0 to 1)	0.04<br>(0.03 to 0.06)	1<br>(1 to 2)	0.05<br>(0.03 to 0.07)	0.77<br>(0.47 to 1.07)	14<br>(9 to 21)	0.97<br>(0.67 to 1.39)	39<br>(26 to 59)	1.24<br>(0.86 to 1.76)	0.79<br>(0.33 to 1.26)
Mauritius	0<br>(0 to 0)	0.02<br>(0.01 to 0.03)	0<br>(0 to 1)	0.02<br>(0.02 to 0.04)	0.49<br>(-0.12 to 1.11)	5<br>(3 to 7)	0.55<br>(0.34 to 0.8)	9<br>(5 to 13)	0.58<br>(0.37 to 0.83)	0.42<br>(-0.17 to 1.01)
Mexico	10<br>(6 to 17)	0.02<br>(0.01 to 0.03)	38<br>(22 to 61)	0.03<br>(0.02 to 0.05)	2.06<br>(1.52 to 2.61)	585<br>(313 to 983)	0.67<br>(0.38 to 1.11)	1252<br>(750 to 2063)	1.05<br>(0.63 to 1.69)	1.54<br>(0.84 to 2.24)
Micronesia (Federated States of)	0<br>(0 to 0)	0.03<br>(0.02 to 0.04)	0<br>(0 to 0)	0.03<br>(0.02 to 0.05)	0.16<br>(-0.72 to 1.05)	1<br>(0 to 1)	0.68<br>(0.43 to 0.98)	1<br>(0 to 1)	0.75<br>(0.48 to 1.1)	0<br>(-1.5 to 1.52)
Monaco	0<br>(0 to 0)	0.11<br>(0.06 to 0.2)	0<br>(0 to 0)	0.17<br>(0.08 to 0.24)	1.41<br>(1.34 to 1.48)	1<br>(1 to 2)	2.03<br>(1.19 to 3.56)	3<br>(1 to 4)	2.96<br>(1.46 to 4.18)	1.27<br>(1.16 to 1.38)
Mongolia	0<br>(0 to 1)	0.04<br>(0.03 to 0.06)	1<br>(0 to 1)	0.03<br>(0.02 to 0.04)	-1.87<br>(-2.34 to -1.39)	19<br>(12 to 28)	1.07<br>(0.71 to 1.52)	30<br>(20 to 43)	0.97<br>(0.66 to 1.33)	-0.52<br>(-0.84 to -0.19)
Montenegro	0<br>(0 to 0)	0.05<br>(0.02 to 0.08)	0<br>(0 to 1)	0.04<br>(0.02 to 0.07)	0.04<br>(-0.24 to 0.32)	8<br>(5 to 12)	1.36<br>(0.89 to 2.06)	8<br>(4 to 12)	1.08<br>(0.68 to 1.64)	-0.65<br>(-0.86 to -0.43)
Morocco	2<br>(1 to 3)	0.01<br>(0.01 to 0.02)	6<br>(4 to 10)	0.02<br>(0.01 to 0.03)	1.33<br>(1.12 to 1.55)	92<br>(52 to 142)	0.41<br>(0.25 to 0.64)	198<br>(120 to 302)	0.58<br>(0.36 to 0.86)	1.1<br>(0.88 to 1.33)
Mozambique	2<br>(1 to 3)	0.03<br>(0.02 to 0.05)	5<br>(3 to 7)	0.04<br>(0.03 to 0.06)	0.97<br>(0.93 to 1.02)	81<br>(48 to 123)	0.81<br>(0.51 to 1.19)	232<br>(136 to 355)	1.08<br>(0.71 to 1.59)	0.96<br>(0.71 to 1.2)
Myanmar	4<br>(2 to 6)	0.02<br>(0.01 to 0.03)	9<br>(5 to 13)	0.02<br>(0.01 to 0.03)	0.37<br>(0.15 to 0.6)	171<br>(91 to 257)	0.51<br>(0.29 to 0.75)	285<br>(167 to 427)	0.57<br>(0.34 to 0.84)	0.34<br>(0.11 to 0.58)
Namibia	0<br>(0 to 0)	0.05<br>(0.03 to 0.07)	1<br>(1 to 1)	0.06<br>(0.04 to 0.09)	0.75<br>(0.59 to 0.91)	12<br>(7 to 18)	1.2<br>(0.81 to 1.74)	28<br>(19 to 42)	1.54<br>(1.1 to 2.2)	0.82<br>(0.61 to 1.04)

Nauru	0<br>(0 to 0)	0.03<br>(0.02 to 0.05)	0<br>(0 to 0)	0.03<br>(0.02 to 0.05)	0.12<br>(0.03 to 0.22)	0<br>(0 to 0)	0.84<br>(0.53 to 1.23)	0<br>(0 to 0)	0.86<br>(0.56 to 1.25)	0.08<br>(-0.02 to 0.18)
Nepal	3<br>(2 to 4)	0.03<br>(0.02 to 0.04)	7<br>(5 to 9)	0.03<br>(0.02 to 0.04)	0.69<br>(0.51 to 0.86)	122<br>(71 to 185)	0.77<br>(0.49 to 1.12)	226<br>(144 to 335)	0.86<br>(0.58 to 1.23)	0.35<br>(0.12 to 0.59)
Netherlands	29<br>(21 to 40)	0.15<br>(0.1 to 0.19)	72<br>(49 to 100)	0.19<br>(0.13 to 0.25)	0.73<br>(-0.6 to 2.08)	493<br>(375 to 636)	2.62<br>(2.03 to 3.3)	1100<br>(796 to 1524)	3.42<br>(2.59 to 4.54)	0.84<br>(-0.46 to 2.16)
New Zealand	13<br>(10 to 17)	0.34<br>(0.27 to 0.42)	40<br>(33 to 48)	0.44<br>(0.37 to 0.53)	0.94<br>(-1.75 to 3.71)	270<br>(223 to 320)	7.14<br>(5.95 to 8.42)	677<br>(570 to 787)	8.52<br>(7.35 to 9.79)	0.68<br>(-1.73 to 3.15)
Nicaragua	1<br>(0 to 1)	0.02<br>(0.01 to 0.03)	1<br>(1 to 2)	0.03<br>(0.02 to 0.04)	0.84<br>(0.69 to 1)	34<br>(20 to 53)	0.82<br>(0.51 to 1.19)	64<br>(41 to 92)	1.06<br>(0.7 to 1.53)	0.88<br>(0.8 to 0.97)
Niger	1<br>(1 to 1)	0.03<br>(0.02 to 0.04)	3<br>(2 to 4)	0.03<br>(0.02 to 0.05)	0.38<br>(0.22 to 0.53)	42<br>(25 to 64)	0.73<br>(0.46 to 1.04)	143<br>(83 to 217)	0.81<br>(0.52 to 1.2)	0.33<br>(0.14 to 0.52)
Nigeria	20<br>(14 to 28)	0.04<br>(0.03 to 0.06)	57<br>(40 to 82)	0.06<br>(0.04 to 0.08)	0.87<br>(0.72 to 1.03)	782<br>(497 to 1142)	1.13<br>(0.77 to 1.63)	2474<br>(1589 to 3708)	1.48<br>(1.05 to 2.11)	0.82<br>(0.62 to 1.02)
Niue	0<br>(0 to 0)	0.03<br>(0.02 to 0.04)	0<br>(0 to 0)	0.04<br>(0.02 to 0.05)	0.45<br>(0.35 to 0.54)	0<br>(0 to 0)	0.75<br>(0.48 to 1.1)	0<br>(0 to 0)	0.85<br>(0.56 to 1.23)	0.39<br>(0.29 to 0.49)
North Macedonia	5<br>(4 to 6)	0.29<br>(0.23 to 0.38)	10<br>(7 to 13)	0.38<br>(0.29 to 0.49)	0.84<br>(0.45 to 1.23)	115<br>(88 to 151)	6.46<br>(4.99 to 8.36)	217<br>(159 to 278)	8.1<br>(6.16 to 10.13)	0.81<br>(0.68 to 0.94)
Northern Mariana Islands	0<br>(0 to 0)	0.04<br>(0.02 to 0.05)	0<br>(0 to 0)	0.04<br>(0.03 to 0.05)	0.04<br>(-0.03 to 0.11)	0<br>(0 to 0)	0.87<br>(0.58 to 1.25)	0<br>(0 to 1)	0.87<br>(0.58 to 1.24)	-0.13<br>(-0.23 to -0.02)
Norway	27<br>(22 to 33)	0.36<br>(0.29 to 0.43)	104<br>(83 to 129)	0.92<br>(0.74 to 1.13)	3.45<br>(2.18 to 4.72)	420<br>(359 to 482)	6.37<br>(5.62 to 7.18)	1665<br>(1366 to 2003)	16.5<br>(13.87 to 19.44)	3.25<br>(1.9 to 4.63)
Oman	0<br>(0 to 0)	0.02<br>(0.01 to 0.03)	1<br>(0 to 1)	0.03<br>(0.02 to 0.05)	1.87<br>(1.74 to 1.99)	8<br>(4 to 12)	0.5<br>(0.3 to 0.75)	30<br>(18 to 48)	0.84<br>(0.55 to 1.23)	1.71<br>(1.48 to 1.95)
Pakistan	13<br>(8 to 20)	0.02<br>(0.01 to 0.03)	35<br>(23 to 52)	0.03<br>(0.02 to 0.04)	0.94<br>(0.63 to 1.24)	624<br>(355 to 962)	0.63<br>(0.38 to 0.93)	1518<br>(901 to 2326)	0.79<br>(0.5 to 1.15)	0.71<br>(0.28 to 1.13)
Palau	0	0.04	0	0.04	0.3	0	0.88	0	0.94	0.24

	(0 to 0)	(0.02 to 0.05)	(0 to 0)	(0.03 to 0.06)	(0.19 to 0.41)	(0 to 0)	(0.58 to 1.25)	(0 to 0)	(0.62 to 1.36)	(0.06 to 0.42)
Palestine	0<br>(0 to 0)	0.01<br>(0.01 to 0.02)	1<br>(0 to 1)	0.02<br>(0.01 to 0.03)	1.66<br>(1.45 to 1.88)	6<br>(3 to 10)	0.36<br>(0.21 to 0.56)	22<br>(13 to 34)	0.57<br>(0.35 to 0.85)	1.46<br>(1.05 to 1.86)
Panama	1<br>(0 to 1)	0.03<br>(0.02 to 0.04)	2<br>(1 to 2)	0.04<br>(0.03 to 0.05)	0.6<br>(0.26 to 0.95)	24<br>(15 to 35)	1.07<br>(0.7 to 1.58)	55<br>(38 to 81)	1.32<br>(0.9 to 1.94)	0.67<br>(0.45 to 0.89)
Papua New Guinea	0<br>(0 to 1)	0.02<br>(0.01 to 0.03)	1<br>(1 to 2)	0.02<br>(0.01 to 0.04)	0.35<br>(0.25 to 0.46)	18<br>(9 to 28)	0.55<br>(0.33 to 0.81)	47<br>(25 to 75)	0.6<br>(0.36 to 0.88)	0.26<br>(0.07 to 0.45)
Paraguay	0<br>(0 to 0)	0.01<br>(0.01 to 0.01)	1<br>(1 to 1)	0.01<br>(0.01 to 0.02)	1.18<br>(0.83 to 1.53)	18<br>(10 to 28)	0.41<br>(0.24 to 0.61)	38<br>(22 to 57)	0.57<br>(0.33 to 0.85)	1.14<br>(0.69 to 1.59)
Peru	2<br>(1 to 3)	0.01<br>(0.01 to 0.02)	5<br>(3 to 6)	0.01<br>(0.01 to 0.02)	0.16<br>(-0.24 to 0.55)	95<br>(53 to 152)	0.44<br>(0.26 to 0.69)	179<br>(103 to 264)	0.52<br>(0.31 to 0.77)	0.07<br>(-0.38 to 0.52)
Philippines	8<br>(5 to 12)	0.03<br>(0.02 to 0.04)	18<br>(11 to 27)	0.02<br>(0.02 to 0.04)	-0.17<br>(-0.44 to 0.1)	359<br>(211 to 539)	0.7<br>(0.45 to 1.04)	633<br>(387 to 944)	0.66<br>(0.42 to 0.98)	-0.2<br>(-0.43 to 0.04)
Poland	46<br>(43 to 48)	0.12<br>(0.11 to 0.13)	295<br>(234 to 375)	0.39<br>(0.32 to 0.5)	4.3<br>(1.63 to 7.05)	1088<br>(1014 to 1172)	2.86<br>(2.61 to 3.14)	5209<br>(4230 to 6414)	8.16<br>(6.71 to 9.9)	3.65<br>(1.78 to 5.56)
Portugal	15<br>(10 to 21)	0.12<br>(0.09 to 0.18)	53<br>(36 to 73)	0.17<br>(0.12 to 0.23)	1.08<br>(-0.56 to 2.75)	267<br>(183 to 374)	2.22<br>(1.6 to 2.99)	744<br>(523 to 993)	3.11<br>(2.3 to 4.08)	1.09<br>(-0.46 to 2.67)
Puerto Rico	0<br>(0 to 1)	0.01<br>(0.01 to 0.02)	1<br>(0 to 1)	0.01<br>(0.01 to 0.02)	1.06<br>(-0.28 to 2.42)	14<br>(8 to 21)	0.39<br>(0.23 to 0.58)	19<br>(11 to 28)	0.5<br>(0.3 to 0.72)	0.97<br>(-0.75 to 2.71)
Qatar	0<br>(0 to 0)	0.02<br>(0.02 to 0.03)	0<br>(0 to 1)	0.03<br>(0.02 to 0.05)	1.22<br>(0.95 to 1.49)	2<br>(1 to 3)	0.61<br>(0.37 to 0.89)	18<br>(10 to 28)	0.85<br>(0.57 to 1.22)	1.09<br>(0.67 to 1.52)
Republic of Korea	57<br>(42 to 77)	0.21<br>(0.16 to 0.27)	211<br>(158 to 277)	0.26<br>(0.19 to 0.33)	0.64<br>(0.46 to 0.82)	2411<br>(1723 to 3326)	6.35<br>(4.73 to 8.49)	4443<br>(3294 to 5749)	6.7<br>(4.99 to 8.59)	0.2<br>(0 to 0.39)
Republic of Moldova	4<br>(3 to 6)	0.11<br>(0.08 to 0.15)	5<br>(4 to 7)	0.1<br>(0.08 to 0.13)	-0.08<br>(-0.96 to 0.8)	107<br>(77 to 153)	2.6<br>(1.93 to 3.65)	130<br>(101 to 173)	2.81<br>(2.18 to 3.72)	0.6<br>(-1.11 to 2.34)
Romania	44<br>(39 to 49)	0.2<br>(0.18 to 0.22)	148<br>(112 to 192)	0.39<br>(0.3 to 0.5)	2.11<br>(1.17 to 3.05)	1117<br>(967 to 1280)	4.82<br>(4.08 to 5.63)	2695<br>(2094 to 3460)	8.82<br>(6.96 to 11.28)	1.94<br>(1.16 to 2.71)
Russian Federation	183<br>(140 to 238)	0.12<br>(0.09 to 0.15)	271<br>(228 to 319)	0.12<br>(0.1 to 0.14)	0.48<br>(-1.65 to 2.66)	4848<br>(3637 to 6539)	3.09<br>(2.33 to 4.07)	6244<br>(5089 to 7716)	3.32<br>(2.66 to 4.11)	0.61<br>(-1.21 to 2.47)

Rwanda	1<br>(1 to 2)	0.04<br>(0.03 to 0.05)	3<br>(2 to 4)	0.05<br>(0.03 to 0.07)	0.84<br>(0.59 to 1.09)	49<br>(28 to 75)	0.95<br>(0.63 to 1.38)	116<br>(73 to 172)	1.22<br>(0.82 to 1.79)	0.85<br>(0.57 to 1.13)
Saint Kitts and Nevis	0<br>(0 to 0)	0.01<br>(0.01 to 0.02)	0<br>(0 to 0)	0.01<br>(0.01 to 0.02)	1.06<br>(0.11 to 2.01)	0<br>(0 to 0)	0.45<br>(0.26 to 0.68)	0<br>(0 to 0)	0.59<br>(0.35 to 0.88)	1.2<br>(-0.31 to 2.74)
Saint Lucia	0<br>(0 to 0)	0.01<br>(0.01 to 0.01)	0<br>(0 to 0)	0.01<br>(0.01 to 0.02)	1.15<br>(-0.11 to 2.42)	1<br>(0 to 1)	0.36<br>(0.2 to 0.55)	1<br>(0 to 1)	0.46<br>(0.26 to 0.68)	0.94<br>(-0.01 to 1.89)
Saint Vincent and the Grenadines	0<br>(0 to 0)	0.01<br>(0 to 0.01)	0<br>(0 to 0)	0.01<br>(0.01 to 0.02)	1.03<br>(-0.07 to 2.13)	0<br>(0 to 1)	0.37<br>(0.21 to 0.58)	0<br>(0 to 1)	0.46<br>(0.27 to 0.72)	0.79<br>(-0.47 to 2.08)
Samoa	0<br>(0 to 0)	0.03<br>(0.02 to 0.04)	0<br>(0 to 0)	0.03<br>(0.02 to 0.04)	0.3<br>(0.14 to 0.45)	1<br>(1 to 1)	0.67<br>(0.43 to 0.95)	1<br>(1 to 2)	0.71<br>(0.47 to 1.03)	0.22<br>(0.05 to 0.39)
San Marino	0<br>(0 to 0)	0.13<br>(0.09 to 0.19)	0<br>(0 to 0)	0.09<br>(0.04 to 0.15)	-1.24<br>(-1.77 to -0.72)	1<br>(0 to 1)	2.22<br>(1.59 to 3.18)	1<br>(1 to 2)	1.59<br>(0.84 to 2.46)	-1.14<br>(-1.54 to -0.74)
Sao Tome and Principe	0<br>(0 to 0)	0.04<br>(0.03 to 0.06)	0<br>(0 to 0)	0.05<br>(0.04 to 0.07)	1.07<br>(0.75 to 1.39)	1<br>(1 to 1)	0.9<br>(0.61 to 1.32)	2<br>(1 to 3)	1.23<br>(0.88 to 1.73)	1.12<br>(0.41 to 1.84)
Saudi Arabia	1<br>(1 to 2)	0.02<br>(0.01 to 0.03)	6<br>(4 to 9)	0.03<br>(0.02 to 0.05)	1.77<br>(1.71 to 1.83)	66<br>(37 to 106)	0.53<br>(0.33 to 0.79)	255<br>(147 to 399)	0.88<br>(0.58 to 1.28)	1.64<br>(1.55 to 1.73)
Senegal	1<br>(1 to 2)	0.04<br>(0.02 to 0.05)	3<br>(2 to 5)	0.05<br>(0.03 to 0.07)	0.91<br>(0.5 to 1.31)	46<br>(27 to 70)	0.86<br>(0.56 to 1.26)	126<br>(83 to 185)	1.12<br>(0.76 to 1.58)	0.93<br>(0.29 to 1.57)
Serbia	16<br>(10 to 24)	0.2<br>(0.13 to 0.28)	40<br>(16 to 67)	0.24<br>(0.1 to 0.39)	0.69<br>(0.47 to 0.9)	393<br>(269 to 564)	4.29<br>(3.01 to 6.04)	685<br>(283 to 1128)	4.79<br>(2.22 to 7.44)	0.41<br>(0.01 to 0.81)
Seychelles	0<br>(0 to 0)	0.02<br>(0.02 to 0.04)	0<br>(0 to 0)	0.03<br>(0.02 to 0.04)	0<br>(-0.08 to 0.09)	0<br>(0 to 1)	0.61<br>(0.39 to 0.89)	1<br>(0 to 1)	0.61<br>(0.38 to 0.9)	-0.01<br>(-0.2 to 0.18)
Sierra Leone	1<br>(0 to 1)	0.04<br>(0.02 to 0.05)	2<br>(1 to 2)	0.04<br>(0.03 to 0.07)	0.64<br>(0.46 to 0.82)	29<br>(17 to 42)	0.9<br>(0.57 to 1.31)	68<br>(41 to 104)	1.09<br>(0.71 to 1.61)	0.64<br>(0.36 to 0.92)
Singapore	4<br>(3 to 5)	0.2<br>(0.15 to 0.26)	18<br>(14 to 23)	0.23<br>(0.18 to 0.29)	0.52<br>(-0.39 to 1.43)	149<br>(109 to 195)	5.69<br>(4.3 to 7.37)	360<br>(283 to 450)	5.28<br>(4.38 to 6.34)	-0.16<br>(-1.11 to 0.79)
Slovakia	8	0.14	13	0.14	-0.02	180	3.31	238	3.09	-0.24

	(5 to 12)	(0.08 to 0.22)	(5 to 21)	(0.06 to 0.23)	(-0.22 to 0.19)	(113 to 262)	(2.1 to 4.77)	(107 to 384)	(1.56 to 4.77)	(-0.4 to -0.07)
Slovenia	2<br>(2 to 2)	0.09<br>(0.07 to 0.1)	15<br>(12 to 17)	0.28<br>(0.24 to 0.33)	4.13<br>(2.95 to 5.33)	43<br>(38 to 49)	2.05<br>(1.8 to 2.32)	221<br>(186 to 257)	5.32<br>(4.59 to 6.21)	3.11<br>(1.71 to 4.53)
Solomon Islands	0<br>(0 to 0)	0.02<br>(0.01 to 0.03)	0<br>(0 to 0)	0.02<br>(0.01 to 0.04)	0.37<br>(0.25 to 0.49)	1<br>(1 to 2)	0.54<br>(0.31 to 0.8)	3<br>(2 to 5)	0.59<br>(0.36 to 0.88)	0.19<br>(0.15 to 0.24)
Somalia	1<br>(0 to 1)	0.03<br>(0.02 to 0.04)	2<br>(1 to 3)	0.03<br>(0.02 to 0.04)	0.1<br>(-0.15 to 0.34)	40<br>(21 to 64)	0.72<br>(0.45 to 1.04)	106<br>(57 to 169)	0.72<br>(0.44 to 1.06)	0.06<br>(-0.36 to 0.49)
South Africa	13<br>(9 to 18)	0.06<br>(0.04 to 0.08)	29<br>(21 to 41)	0.07<br>(0.05 to 0.1)	0.55<br>(0.48 to 0.61)	463<br>(306 to 670)	1.57<br>(1.12 to 2.26)	936<br>(661 to 1355)	1.87<br>(1.34 to 2.63)	0.58<br>(0.5 to 0.66)
South Sudan	1<br>(1 to 1)	0.04<br>(0.02 to 0.05)	1<br>(1 to 2)	0.04<br>(0.02 to 0.06)	0.27<br>(0.08 to 0.47)	39<br>(23 to 58)	0.89<br>(0.57 to 1.3)	64<br>(38 to 99)	0.94<br>(0.61 to 1.36)	0.24<br>(-0.26 to 0.75)
Spain	107<br>(84 to 134)	0.21<br>(0.16 to 0.26)	349<br>(273 to 431)	0.3<br>(0.25 to 0.37)	1.3<br>(0.39 to 2.21)	2016<br>(1693 to 2379)	4.19<br>(3.61 to 4.84)	5480<br>(4535 to 6554)	6.25<br>(5.36 to 7.24)	1.35<br>(0.47 to 2.22)
Sri Lanka	2<br>(1 to 3)	0.02<br>(0.01 to 0.03)	5<br>(3 to 8)	0.02<br>(0.01 to 0.03)	0.17<br>(-0.05 to 0.39)	72<br>(42 to 112)	0.55<br>(0.33 to 0.81)	134<br>(82 to 199)	0.57<br>(0.36 to 0.82)	0.14<br>(-0.17 to 0.46)
Sudan	1<br>(1 to 2)	0.01<br>(0.01 to 0.02)	5<br>(3 to 7)	0.02<br>(0.01 to 0.03)	1.77<br>(1.56 to 1.98)	77<br>(38 to 125)	0.41<br>(0.23 to 0.65)	227<br>(130 to 354)	0.63<br>(0.39 to 0.93)	1.44<br>(1.19 to 1.69)
Suriname	0<br>(0 to 0)	0.01<br>(0.01 to 0.01)	0<br>(0 to 0)	0.01<br>(0.01 to 0.02)	0.71<br>(0.11 to 1.31)	2<br>(1 to 3)	0.43<br>(0.24 to 0.66)	3<br>(2 to 4)	0.51<br>(0.29 to 0.78)	0.65<br>(-0.26 to 1.57)
Sweden	27<br>(23 to 30)	0.16<br>(0.14 to 0.18)	167<br>(128 to 214)	0.66<br>(0.52 to 0.83)	4.99<br>(2.89 to 7.14)	364<br>(323 to 401)	2.48<br>(2.22 to 2.71)	2619<br>(2071 to 3289)	12.5<br>(10.27 to 15.06)	5.67<br>(3.69 to 7.68)
Switzerland	25<br>(19 to 33)	0.23<br>(0.18 to 0.3)	62<br>(46 to 81)	0.29<br>(0.22 to 0.37)	0.79<br>(-0.17 to 1.75)	437<br>(346 to 549)	4.55<br>(3.73 to 5.51)	947<br>(722 to 1207)	5.76<br>(4.61 to 7.17)	0.91<br>(-0.05 to 1.89)
Syrian Arab Republic	1<br>(1 to 1)	0.02<br>(0.01 to 0.02)	2<br>(2 to 4)	0.02<br>(0.02 to 0.04)	1.56<br>(1.37 to 1.75)	46<br>(25 to 74)	0.42<br>(0.26 to 0.64)	78<br>(48 to 121)	0.63<br>(0.41 to 0.95)	1.28<br>(1.07 to 1.48)
Taiwan (Province of China)	10<br>(7 to 14)	0.07<br>(0.05 to 0.1)	49<br>(38 to 60)	0.11<br>(0.09 to 0.14)	1.32<br>(-0.2 to 2.87)	307<br>(218 to 450)	1.82<br>(1.33 to 2.55)	914<br>(726 to 1118)	2.56<br>(2.1 to 3.08)	1.31<br>(0.92 to 1.71)

Tajikistan	1<br>(1 to 2)	0.04<br>(0.03 to 0.06)	3<br>(2 to 4)	0.05<br>(0.04 to 0.08)	0.72<br>(0.14 to 1.31)	48<br>(29 to 69)	1.08<br>(0.73 to 1.54)	95<br>(59 to 140)	1.26<br>(0.87 to 1.76)	0.54<br>(0.41 to 0.68)
Thailand	7<br>(4 to 10)	0.02<br>(0.01 to 0.03)	22<br>(14 to 33)	0.02<br>(0.01 to 0.03)	0.16<br>(-0.06 to 0.37)	236<br>(141 to 360)	0.54<br>(0.33 to 0.8)	469<br>(292 to 713)	0.56<br>(0.35 to 0.83)	0.13<br>(-0.2 to 0.47)
Timor-Leste	0<br>(0 to 0)	0.02<br>(0.01 to 0.02)	0<br>(0 to 0)	0.02<br>(0.01 to 0.03)	0.59<br>(0.3 to 0.88)	3<br>(1 to 4)	0.44<br>(0.24 to 0.67)	6<br>(3 to 9)	0.52<br>(0.31 to 0.76)	0.46<br>(0.11 to 0.81)
Togo	1<br>(0 to 1)	0.04<br>(0.03 to 0.06)	2<br>(1 to 2)	0.05<br>(0.03 to 0.07)	0.61<br>(0.48 to 0.73)	23<br>(14 to 36)	0.96<br>(0.64 to 1.37)	68<br>(43 to 106)	1.17<br>(0.8 to 1.71)	0.66<br>(0.52 to 0.79)
Tokelau	0<br>(0 to 0)	0.03<br>(0.02 to 0.04)	0<br>(0 to 0)	0.03<br>(0.02 to 0.05)	0.54<br>(0.48 to 0.61)	0<br>(0 to 0)	0.66<br>(0.42 to 0.97)	0<br>(0 to 0)	0.77<br>(0.51 to 1.11)	0.5<br>(0.41 to 0.58)
Tonga	0<br>(0 to 0)	0.02<br>(0.02 to 0.04)	0<br>(0 to 0)	0.03<br>(0.02 to 0.04)	0.52<br>(0.35 to 0.7)	0<br>(0 to 1)	0.59<br>(0.36 to 0.88)	1<br>(0 to 1)	0.69<br>(0.45 to 1.01)	0.47<br>(0.27 to 0.67)
Trinidad and Tobago	0<br>(0 to 0)	0.01<br>(0.01 to 0.02)	0<br>(0 to 0)	0.01<br>(0.01 to 0.02)	0.96<br>(-0.18 to 2.12)	5<br>(3 to 8)	0.46<br>(0.26 to 0.7)	8<br>(5 to 11)	0.57<br>(0.34 to 0.83)	0.84<br>(-0.22 to 1.92)
Tunisia	1<br>(0 to 1)	0.02<br>(0.01 to 0.02)	3<br>(2 to 4)	0.02<br>(0.02 to 0.04)	1.39<br>(1.32 to 1.46)	31<br>(18 to 49)	0.44<br>(0.27 to 0.65)	75<br>(48 to 113)	0.64<br>(0.41 to 0.94)	1.18<br>(1.13 to 1.22)
Turkey	5<br>(3 to 8)	0.02<br>(0.01 to 0.02)	18<br>(12 to 26)	0.02<br>(0.02 to 0.03)	1.19<br>(0.94 to 1.45)	213<br>(125 to 327)	0.43<br>(0.27 to 0.65)	475<br>(306 to 707)	0.58<br>(0.38 to 0.82)	0.94<br>(0.76 to 1.13)
Turkmenista n	1<br>(1 to 1)	0.05<br>(0.03 to 0.07)	2<br>(1 to 3)	0.06<br>(0.04 to 0.08)	1.07<br>(-0.75 to 2.91)	35<br>(22 to 52)	1.21<br>(0.84 to 1.73)	65<br>(45 to 97)	1.49<br>(1.08 to 2.11)	0.86<br>(-0.91 to 2.66)
Tuvalu	0<br>(0 to 0)	0.02<br>(0.01 to 0.03)	0<br>(0 to 0)	0.03<br>(0.02 to 0.04)	0.75<br>(0.68 to 0.81)	0<br>(0 to 0)	0.6<br>(0.36 to 0.87)	0<br>(0 to 0)	0.74<br>(0.46 to 1.08)	0.72<br>(0.64 to 0.8)
Uganda	2<br>(2 to 3)	0.03<br>(0.02 to 0.05)	8<br>(5 to 12)	0.05<br>(0.03 to 0.07)	1.06<br>(0.87 to 1.26)	106<br>(60 to 159)	0.85<br>(0.55 to 1.23)	358<br>(211 to 549)	1.21<br>(0.82 to 1.76)	1.06<br>(0.81 to 1.32)
Ukraine	41<br>(34 to 49)	0.06<br>(0.05 to 0.08)	25<br>(19 to 31)	0.04<br>(0.03 to 0.05)	-0.65<br>(-2.95 to 1.7)	1304<br>(1014 to 1703)	2.26<br>(1.7 to 3.04)	770<br>(601 to 939)	1.56<br>(1.26 to 1.88)	-1.47<br>(-2.56 to -0.37)
United Arab Emirates	0<br>(0 to 0)	0.02<br>(0.01 to 0.03)	2<br>(1 to 3)	0.04<br>(0.02 to 0.05)	1.49<br>(0.93 to 2.06)	8<br>(4 to 14)	0.61<br>(0.38 to 0.91)	69<br>(39 to 113)	0.92<br>(0.62 to 1.34)	1.29<br>(0.98 to 1.59)
United	91	0.1	387	0.26	3.5	1454	1.74	5840	4.61	3.55

Kingdom	(83 to 97)	(0.09 to 0.11)	(288 to 507)	(0.2 to 0.33)	(-0.17 to 7.3)	(1356 to 1531)	(1.62 to 1.87)	(4609 to 7365)	(3.82 to 5.6)	(0.11 to 7.12)
United Republic of Tanzania	4 (3 to 6)	0.04 (0.03 to 0.06)	13 (9 to 18)	0.05 (0.03 to 0.07)	0.81 (0.65 to 0.98)	175 (105 to 269)	0.94 (0.61 to 1.37)	508 (317 to 749)	1.22 (0.83 to 1.75)	0.82 (0.56 to 1.08)
United States of America	572 (505 to 614)	0.18 (0.16 to 0.19)	7476 (6685 to 8260)	1.22 (1.09 to 1.33)	0.78 (0.49 to 1.08)	11726 (10785 to 12561)	4.14 (3.78 to 4.48)	130679 (120541 to 139631)	23.45 (21.81 to 24.85)	0.52 (0 to 1.05)
United States Virgin Islands	0 (0 to 0)	0.01 (0.01 to 0.02)	0 (0 to 0)	0.01 (0.01 to 0.02)	6.26 (5.55 to 6.98)	0 (0 to 1)	0.46 (0.27 to 0.68)	1 (0 to 1)	0.53 (0.33 to 0.74)	5.54 (4.99 to 6.09)
Uruguay	3 (2 to 4)	0.08 (0.06 to 0.11)	7 (5 to 9)	0.11 (0.08 to 0.15)	0.86 (-0.54 to 2.29)	68 (49 to 93)	1.91 (1.4 to 2.64)	120 (87 to 163)	2.46 (1.81 to 3.4)	0.83 (-0.52 to 2.2)
Uzbekistan	6 (4 to 8)	0.05 (0.03 to 0.07)	11 (8 to 15)	0.05 (0.04 to 0.06)	0.48 (-2.4 to 3.44)	194 (119 to 280)	1.14 (0.76 to 1.66)	384 (255 to 554)	1.32 (0.94 to 1.87)	0.85 (-1.55 to 3.3)
Vanuatu	0 (0 to 0)	0.02 (0.01 to 0.03)	0 (0 to 0)	0.03 (0.02 to 0.04)	0.32 (0.1 to 0.54)	1 (0 to 1)	0.57 (0.34 to 0.83)	1 (1 to 2)	0.61 (0.38 to 0.91)	0.22 (-0.23 to 0.68)
Venezuela (Bolivarian Republic of)	4 (2 to 5)	0.03 (0.02 to 0.04)	9 (6 to 13)	0.03 (0.02 to 0.05)	0.27 (-0.81 to 1.38)	186 (113 to 276)	1.06 (0.69 to 1.54)	305 (205 to 439)	1.16 (0.78 to 1.65)	0.32 (-1.03 to 1.7)
Viet Nam	8 (5 to 11)	0.02 (0.01 to 0.03)	18 (11 to 28)	0.02 (0.01 to 0.03)	0.45 (0.32 to 0.58)	254 (147 to 379)	0.46 (0.28 to 0.68)	491 (286 to 737)	0.53 (0.32 to 0.78)	0.45 (0.29 to 0.61)
Yemen	1 (0 to 1)	0.01 (0.01 to 0.02)	3 (2 to 5)	0.02 (0.01 to 0.03)	1.49 (1.28 to 1.69)	51 (25 to 85)	0.39 (0.21 to 0.61)	153 (83 to 249)	0.55 (0.33 to 0.82)	1.47 (1.21 to 1.74)
Zambia	1 (1 to 2)	0.04 (0.03 to 0.06)	4 (3 to 6)	0.06 (0.04 to 0.08)	0.93 (0.85 to 1.02)	60 (35 to 94)	1.08 (0.71 to 1.59)	195 (121 to 304)	1.45 (0.99 to 2.1)	0.96 (0.83 to 1.09)
Zimbabwe	2 (1 to 3)	0.05 (0.03 to 0.07)	3 (2 to 5)	0.05 (0.04 to 0.08)	0.14 (0.04 to 0.25)	87 (52 to 133)	1.22 (0.82 to 1.79)	142 (89 to 210)	1.32 (0.9 to 1.88)	0.13 (-0.08 to 0.33)

Abbreviations: DALY, disability-adjusted life-year; UIs, uncertainty intervals; AAPC, average annual percent change; CI, confidence Interval.

Table S7: Contribution of population aging, growth, and epidemiological changes to DALYs caused by CDI, 1990-2021.

Location/Metric	Overall difference	Aging	Population	Epidemiological change	Percent change of aging	Percent change of population	Percent change of epidemiological change
**Global**							
Male	101382.02	26260.10	30262.11	44859.81	25.90	29.85	44.25
Female	108857.51	27475.90	30820.20	50561.41	25.24	28.31	46.45
Both	210239.53	53569.17	61092.96	95577.41	25.48	29.06	45.46
**SDI quintiles**							
High SDI	168309.13	40781.66	23827.61	103699.86	24.23	14.16	61.61
High-middle SDI	20798.56	10262.43	5313.33	5222.80	49.34	25.55	25.11
Middle SDI	8893.87	1748.46	4175.28	2970.14	19.66	46.95	33.40
Low-middle SDI	7174.17	223.11	4351.43	2599.64	3.11	60.65	36.24
Low SDI	4890.42	-86.90	3856.84	1120.48	-1.78	78.87	22.91
**Health system Grouping Levels**							
Advanced Health System	186177.28	48563.47	19726.08	117887.73	26.08	10.60	63.32
Basic Health System	10176.80	2804.77	4618.41	2753.62	27.56	45.38	27.06
Limited Health System	12271.37	474.43	7887.80	3909.14	3.87	64.28	31.86
Minimal Health System	1440.70	-79.41	1301.58	218.54	-5.51	90.34	15.17
**GBD regions**							
Andean Latin America	212.81	-8.21	136.29	84.72	-3.86	64.04	39.81
Australasia	1395.89	503.10	562.54	330.26	36.04	40.30	23.66
Caribbean	87.41	-11.42	55.59	43.25	-13.06	63.59	49.48
Central Asia	445.22	38.05	297.24	109.94	8.55	66.76	24.69
Central Europe	9263.01	3856.25	-783.14	6189.91	41.63	-8.45	66.82
Central Latin America	1337.70	-69.21	829.85	577.07	-5.17	62.04	43.14

Central Sub-Saharan Africa	750.28	-10.99	625.94	135.33	-1.46	83.43	18.04
East Asia	4733.11	2299.29	1224.15	1209.67	48.58	25.86	25.56
Eastern Europe	880.50	1397.48	-656.31	139.33	158.71	-74.54	15.82
Eastern Sub-Saharan Africa	2268.64	-41.66	1745.36	564.94	-1.84	76.93	24.90
High-income Asia Pacific	9710.79	7522.15	892.53	1296.11	77.46	9.19	13.35
High-income North America	126778.33	19073.00	17501.65	90203.68	15.04	13.80	71.15
North Africa and Middle East	2254.64	94.16	1385.74	774.74	4.18	61.46	34.36
Oceania	37.99	1.21	34.42	2.35	3.19	90.61	6.19
South Asia	6615.57	426.23	3936.63	2252.72	6.44	59.51	34.05
Southeast Asia	1756.64	367.39	1177.55	211.69	20.91	67.03	12.05
Southern Latin America	1447.20	287.67	411.99	747.55	19.88	28.47	51.65
Southern Sub-Saharan Africa	579.15	74.48	359.93	144.75	12.86	62.15	24.99
Tropical Latin America	578.91	-66.41	301.86	343.46	-11.47	52.14	59.33
Western Europe	36047.13	12485.76	4533.86	19027.51	34.64	12.58	52.79
Western Sub-Saharan Africa	3058.60	-121.51	2484.47	695.64	-3.97	81.23	22.74

Abbreviations: DALY, disability-adjusted life-year; CDI, *Clostridioides difficile infections*.

Table S8: The global trends and projections of deaths and DALYs of CDI between 2022 and 2040 by sex.

Sex	Year	Number of deaths	Number of DALYs	ASMRs	ASDRs
Male	2022	7472	146238	0.2077	3.7672
Male	2023	7662	149002	0.2063	3.741
Male	2024	7881	152047	0.205	3.7147
Male	2025	8123	155335	0.2041	3.6939
Male	2026	8365	158614	0.2032	3.6731
Male	2027	8597	161795	0.2023	3.6523
Male	2028	8842	165089	0.2014	3.6315
Male	2029	9113	168632	0.2006	3.6106
Male	2030	9410	172534	0.1999	3.5951
Male	2031	9710	176439	0.1993	3.5796
Male	2032	10003	180252	0.1987	3.564
Male	2033	10309	184146	0.1981	3.5485
Male	2034	10640	188260	0.1975	3.533
Male	2035	10995	192744	0.1971	3.5228
Male	2036	11351	197201	0.1966	3.5127
Male	2037	11697	201531	0.1962	3.5026
Male	2038	12052	205893	0.1958	3.4924
Male	2039	12428	210427	0.1954	3.4823
Male	2040	12813	215023	0.195	3.4722
Female	2022	8931	150254	0.1837	3.2059
Female	2023	9112	152877	0.182	3.1745
Female	2024	9324	155806	0.1802	3.143
Female	2025	9588	159300	0.1792	3.1214
Female	2026	9852	162803	0.1781	3.0998
Female	2027	10107	166230	0.1771	3.0783

Female	2028	10378	169795	0.1761	3.0567
Female	2029	10679	173641	0.1751	3.0351
Female	2030	11030	178108	0.1746	3.0216
Female	2031	11389	182616	0.1741	3.008
Female	2032	11746	187068	0.1735	2.9945
Female	2033	12120	191637	0.173	2.9809
Female	2034	12525	196457	0.1725	2.9673
Female	2035	12975	201886	0.1723	2.9618
Female	2036	13431	207322	0.1722	2.9563
Female	2037	13882	212662	0.1721	2.9508
Female	2038	14346	218069	0.1719	2.9452
Female	2039	14836	223682	0.1718	2.9397
Female	2040	15340	229378	0.1716	2.9342
Both	2022	16403	296492	0.1943	3.4707
Both	2023	16774	301879	0.1927	3.4415
Both	2024	17205	307853	0.1911	3.4124
Both	2025	17711	314636	0.1902	3.3912
Both	2026	18216	321417	0.1892	3.3699
Both	2027	18704	328025	0.1883	3.3486
Both	2028	19220	334884	0.1873	3.3273
Both	2029	19793	342273	0.1864	3.3061
Both	2030	20440	350642	0.1858	3.2917
Both	2031	21098	359055	0.1853	3.2773
Both	2032	21749	367319	0.1847	3.2629
Both	2033	22429	375783	0.1841	3.2484
Both	2034	23165	384718	0.1836	3.234
Both	2035	23970	394630	0.1833	3.2264

Both	2036	24782	404524	0.1831	3.2188
Both	2037	25579	414193	0.1828	3.2112
Both	2038	26397	423962	0.1826	3.2036
Both	2039	27264	434109	0.1823	3.1961
Both	2040	28153	444401	0.182	3.1885

Abbreviations: CDI, *Clostridioides difficile infections*; ASMRs, age-standardized mortality rates; ASDRs, age-standardized DALY rates; DALY, disability-adjusted life-year.

Table S9: The global trends and projections of deaths and DALYs of CDI between 2022 and 2040 by age groups.

Age group	Year	Number of deaths	Number of DALYs	Death rates	DALY rates

<5 years	2022	97	8753	0.0145	1.3060
<5 years	2023	95	8598	0.0142	1.2852
<5 years	2024	92	8438	0.0138	1.2643
<5 years	2025	90	8311	0.0136	1.2496
<5 years	2026	88	8181	0.0133	1.2349
<5 years	2027	86	8047	0.0131	1.2201
<5 years	2028	84	7911	0.0129	1.2054
<5 years	2029	82	7775	0.0126	1.1907
<5 years	2030	81	7675	0.0125	1.1813
<5 years	2031	80	7577	0.0123	1.1719
<5 years	2032	78	7481	0.0122	1.1625
<5 years	2033	77	7386	0.0120	1.1531
<5 years	2034	76	7293	0.0119	1.1437
<5 years	2035	75	7232	0.0118	1.1391
<5 years	2036	74	7173	0.0117	1.1345
<5 years	2037	74	7115	0.0117	1.1299
<5 years	2038	73	7058	0.0116	1.1254
<5 years	2039	72	7001	0.0115	1.1208
<5 years	2040	71	6944	0.0115	1.1163
5-9 years	2022	70	5993	0.0104	0.8872
5-9 years	2023	68	5871	0.0101	0.8700
5-9 years	2024	66	5741	0.0098	0.8528
5-9 years	2025	65	5646	0.0096	0.8413
5-9 years	2026	63	5550	0.0094	0.8298
5-9 years	2027	62	5448	0.0092	0.8183
5-9 years	2028	60	5363	0.0091	0.8067
5-9 years	2029	59	5274	0.0089	0.7952

5-9 years	2030	58	5208	0.0087	0.7879
5-9 years	2031	57	5140	0.0086	0.7806
5-9 years	2032	56	5070	0.0085	0.7733
5-9 years	2033	55	4998	0.0084	0.7659
5-9 years	2034	54	4926	0.0083	0.7586
5-9 years	2035	53	4880	0.0082	0.7551
5-9 years	2036	52	4834	0.0081	0.7515
5-9 years	2037	52	4789	0.0081	0.7480
5-9 years	2038	51	4745	0.0080	0.7444
5-9 years	2039	51	4701	0.0080	0.7409
5-9 years	2040	50	4659	0.0079	0.7373
10-14 years	2022	48	3864	0.0072	0.5858
10-14 years	2023	45	3739	0.0068	0.5637
10-14 years	2024	43	3611	0.0064	0.5416
10-14 years	2025	43	3605	0.0064	0.5384
10-14 years	2026	43	3596	0.0063	0.5352
10-14 years	2027	42	3586	0.0063	0.5320
10-14 years	2028	42	3561	0.0062	0.5288
10-14 years	2029	42	3531	0.0062	0.5256
10-14 years	2030	41	3484	0.0061	0.5202
10-14 years	2031	40	3436	0.0060	0.5148
10-14 years	2032	39	3385	0.0059	0.5095
10-14 years	2033	39	3344	0.0058	0.5041
10-14 years	2034	38	3301	0.0057	0.4987
10-14 years	2035	37	3273	0.0057	0.4961
10-14 years	2036	37	3244	0.0056	0.4935
10-14 years	2037	37	3213	0.0056	0.4909

10-14 years	2038	36	3181	0.0055	0.4883
10-14 years	2039	36	3149	0.0055	0.4857
10-14 years	2040	35	3117	0.0054	0.4831
15-19 years	2022	34	2610	0.0053	0.4115
15-19 years	2023	32	2525	0.0050	0.3952
15-19 years	2024	30	2439	0.0047	0.3789
15-19 years	2025	30	2413	0.0046	0.3720
15-19 years	2026	29	2386	0.0045	0.3651
15-19 years	2027	29	2356	0.0044	0.3582
15-19 years	2028	28	2324	0.0042	0.3513
15-19 years	2029	27	2290	0.0041	0.3444
15-19 years	2030	28	2297	0.0041	0.3440
15-19 years	2031	28	2302	0.0041	0.3436
15-19 years	2032	28	2307	0.0041	0.3432
15-19 years	2033	28	2302	0.0041	0.3428
15-19 years	2034	28	2294	0.0041	0.3424
15-19 years	2035	27	2275	0.0041	0.3405
15-19 years	2036	27	2255	0.0041	0.3387
15-19 years	2037	27	2233	0.0040	0.3369
15-19 years	2038	26	2217	0.0040	0.3350
15-19 years	2039	26	2200	0.0040	0.3332
15-19 years	2040	26	2181	0.0039	0.3313
20-24 years	2022	37	2650	0.0060	0.4320
20-24 years	2023	35	2577	0.0058	0.4181
20-24 years	2024	34	2505	0.0055	0.4042
20-24 years	2025	33	2461	0.0053	0.3947
20-24 years	2026	32	2417	0.0051	0.3853

20-24 years	2027	31	2372	0.0049	0.3758
20-24 years	2028	30	2329	0.0047	0.3663
20-24 years	2029	29	2286	0.0045	0.3567
20-24 years	2030	29	2272	0.0045	0.3518
20-24 years	2031	29	2257	0.0044	0.3470
20-24 years	2032	28	2241	0.0043	0.3421
20-24 years	2033	28	2222	0.0042	0.3373
20-24 years	2034	28	2201	0.0042	0.3324
20-24 years	2035	28	2220	0.0042	0.3337
20-24 years	2036	28	2236	0.0042	0.3350
20-24 years	2037	28	2252	0.0043	0.3364
20-24 years	2038	29	2259	0.0043	0.3377
20-24 years	2039	29	2263	0.0043	0.3390
20-24 years	2040	29	2265	0.0043	0.3403
25-29 years	2022	51	3364	0.0085	0.5600
25-29 years	2023	49	3255	0.0081	0.5415
25-29 years	2024	46	3151	0.0077	0.5230
25-29 years	2025	46	3114	0.0075	0.5152
25-29 years	2026	45	3080	0.0074	0.5074
25-29 years	2027	44	3046	0.0072	0.4995
25-29 years	2028	43	3013	0.0070	0.4916
25-29 years	2029	42	2980	0.0069	0.4836
25-29 years	2030	41	2943	0.0067	0.4749
25-29 years	2031	41	2907	0.0065	0.4661
25-29 years	2032	40	2871	0.0063	0.4573
25-29 years	2033	39	2836	0.0061	0.4485
25-29 years	2034	38	2801	0.0060	0.4396

25-29 years	2035	38	2800	0.0059	0.4359
25-29 years	2036	38	2797	0.0058	0.4323
25-29 years	2037	38	2792	0.0058	0.4286
25-29 years	2038	38	2785	0.0057	0.4250
25-29 years	2039	37	2776	0.0057	0.4213
25-29 years	2040	37	2764	0.0056	0.4177
30-34 years	2022	74	4471	0.0121	0.7312
30-34 years	2023	71	4351	0.0117	0.7129
30-34 years	2024	68	4215	0.0113	0.6947
30-34 years	2025	66	4108	0.0110	0.6822
30-34 years	2026	64	4007	0.0107	0.6698
30-34 years	2027	62	3922	0.0104	0.6573
30-34 years	2028	61	3850	0.0102	0.6449
30-34 years	2029	59	3785	0.0099	0.6325
30-34 years	2030	59	3760	0.0097	0.6263
30-34 years	2031	58	3738	0.0096	0.6199
30-34 years	2032	57	3716	0.0094	0.6135
30-34 years	2033	57	3696	0.0093	0.6071
30-34 years	2034	56	3676	0.0091	0.6006
30-34 years	2035	55	3650	0.0090	0.5928
30-34 years	2036	55	3625	0.0088	0.5850
30-34 years	2037	54	3601	0.0086	0.5772
30-34 years	2038	53	3577	0.0085	0.5693
30-34 years	2039	52	3555	0.0083	0.5614
30-34 years	2040	52	3533	0.0081	0.5534
35-39 years	2022	99	5399	0.0170	0.9298
35-39 years	2023	96	5331	0.0163	0.9056

35-39 years	2024	94	5249	0.0157	0.8813
35-39 years	2025	93	5225	0.0154	0.8694
35-39 years	2026	92	5186	0.0151	0.8576
35-39 years	2027	90	5129	0.0148	0.8459
35-39 years	2028	88	5049	0.0146	0.8343
35-39 years	2029	86	4950	0.0143	0.8228
35-39 years	2030	84	4848	0.0140	0.8119
35-39 years	2031	82	4753	0.0138	0.8010
35-39 years	2032	80	4675	0.0135	0.7901
35-39 years	2033	79	4615	0.0133	0.7793
35-39 years	2034	77	4561	0.0130	0.7685
35-39 years	2035	77	4553	0.0129	0.7645
35-39 years	2036	77	4548	0.0128	0.7605
35-39 years	2037	76	4545	0.0127	0.7563
35-39 years	2038	76	4543	0.0126	0.7521
35-39 years	2039	76	4542	0.0125	0.7478
35-39 years	2040	75	4543	0.0123	0.7435
40-44 years	2022	130	6446	0.0251	1.2434
40-44 years	2023	129	6469	0.0245	1.2251
40-44 years	2024	129	6515	0.0239	1.2069
40-44 years	2025	129	6563	0.0234	1.1882
40-44 years	2026	129	6600	0.0229	1.1696
40-44 years	2027	129	6608	0.0225	1.1509
40-44 years	2028	128	6591	0.0220	1.1322
40-44 years	2029	127	6558	0.0215	1.1134
40-44 years	2030	126	6561	0.0213	1.1035
40-44 years	2031	126	6543	0.0210	1.0937

40-44 years	2032	124	6504	0.0207	1.0840
40-44 years	2033	123	6434	0.0205	1.0745
40-44 years	2034	120	6341	0.0202	1.0651
40-44 years	2035	118	6240	0.0200	1.0559
40-44 years	2036	116	6148	0.0197	1.0468
40-44 years	2037	114	6077	0.0195	1.0377
40-44 years	2038	113	6029	0.0193	1.0287
40-44 years	2039	112	5991	0.0191	1.0197
40-44 years	2040	111	5959	0.0188	1.0108
45-49 years	2022	180	7974	0.0371	1.6434
45-49 years	2023	178	7937	0.0363	1.6228
45-49 years	2024	175	7899	0.0356	1.6022
45-49 years	2025	175	7914	0.0351	1.5906
45-49 years	2026	175	7944	0.0347	1.5790
45-49 years	2027	175	7997	0.0343	1.5675
45-49 years	2028	176	8087	0.0339	1.5559
45-49 years	2029	178	8208	0.0335	1.5444
45-49 years	2030	180	8309	0.0331	1.5275
45-49 years	2031	181	8395	0.0326	1.5105
45-49 years	2032	182	8448	0.0321	1.4935
45-49 years	2033	182	8469	0.0317	1.4764
45-49 years	2034	181	8472	0.0312	1.4593
45-49 years	2035	182	8514	0.0310	1.4528
45-49 years	2036	182	8532	0.0308	1.4464
45-49 years	2037	181	8520	0.0306	1.4402
45-49 years	2038	180	8468	0.0304	1.4341
45-49 years	2039	177	8384	0.0302	1.4281

45-49 years	2040	175	8289	0.0300	1.4222
50-54 years	2022	283	11051	0.0616	2.4076
50-54 years	2023	280	11022	0.0605	2.3803
50-54 years	2024	277	10961	0.0594	2.3529
50-54 years	2025	275	10947	0.0588	2.3387
50-54 years	2026	274	10936	0.0583	2.3244
50-54 years	2027	273	10945	0.0577	2.3102
50-54 years	2028	273	10966	0.0571	2.2960
50-54 years	2029	272	10988	0.0565	2.2817
50-54 years	2030	273	11056	0.0562	2.2746
50-54 years	2031	274	11146	0.0558	2.2674
50-54 years	2032	277	11270	0.0555	2.2603
50-54 years	2033	280	11448	0.0552	2.2532
50-54 years	2034	285	11673	0.0548	2.2461
50-54 years	2035	289	11872	0.0544	2.2312
50-54 years	2036	293	12053	0.0539	2.2164
50-54 years	2037	296	12186	0.0535	2.2015
50-54 years	2038	298	12276	0.0531	2.1865
50-54 years	2039	299	12342	0.0526	2.1714
50-54 years	2040	300	12376	0.0522	2.1564
55-59 years	2022	389	13270	0.0950	3.2438
55-59 years	2023	385	13191	0.0919	3.1524
55-59 years	2024	378	13048	0.0888	3.0613
55-59 years	2025	380	13132	0.0878	3.0345
55-59 years	2026	380	13185	0.0867	3.0076
55-59 years	2027	380	13217	0.0857	2.9805
55-59 years	2028	379	13213	0.0847	2.9531

55-59 years	2029	377	13171	0.0837	2.9255
55-59 years	2030	377	13204	0.0833	2.9187
55-59 years	2031	377	13243	0.0828	2.9118
55-59 years	2032	378	13307	0.0824	2.9049
55-59 years	2033	379	13385	0.0820	2.8981
55-59 years	2034	380	13466	0.0816	2.8912
55-59 years	2035	383	13601	0.0815	2.8927
55-59 years	2036	387	13765	0.0814	2.8942
55-59 years	2037	392	13973	0.0813	2.8957
55-59 years	2038	399	14251	0.0812	2.8972
55-59 years	2039	408	14589	0.0811	2.8988
55-59 years	2040	417	14950	0.0810	2.9004
60-64 years	2022	714	20968	0.2171	6.3771
60-64 years	2023	721	21258	0.2130	6.2804
60-64 years	2024	733	21697	0.2088	6.1837
60-64 years	2025	744	22065	0.2042	6.0551
60-64 years	2026	754	22377	0.1996	5.9266
60-64 years	2027	758	22548	0.1950	5.7982
60-64 years	2028	758	22561	0.1905	5.6700
60-64 years	2029	754	22468	0.1859	5.5420
60-64 years	2030	761	22711	0.1847	5.5156
60-64 years	2031	766	22903	0.1836	5.4890
60-64 years	2032	770	23058	0.1824	5.4621
60-64 years	2033	772	23154	0.1812	5.4348
60-64 years	2034	772	23184	0.1800	5.4074
60-64 years	2035	775	23328	0.1799	5.4130
60-64 years	2036	779	23481	0.1798	5.4187

60-64 years	2037	785	23680	0.1797	5.4244
60-64 years	2038	791	23907	0.1796	5.4301
60-64 years	2039	797	24139	0.1795	5.4358
60-64 years	2040	805	24401	0.1794	5.4414
65-69 years	2022	1335	32933	0.4741	11.6961
65-69 years	2023	1359	33618	0.4737	11.7219
65-69 years	2024	1374	34110	0.4733	11.7477
65-69 years	2025	1376	34194	0.4686	11.6481
65-69 years	2026	1381	34372	0.4639	11.5486
65-69 years	2027	1395	34775	0.4592	11.4494
65-69 years	2028	1423	35525	0.4545	11.3504
65-69 years	2029	1461	36542	0.4498	11.2515
65-69 years	2030	1494	37377	0.4426	11.0709
65-69 years	2031	1524	38124	0.4354	10.8903
65-69 years	2032	1544	38635	0.4281	10.7099
65-69 years	2033	1554	38886	0.4209	10.5297
65-69 years	2034	1558	38965	0.4137	10.3499
65-69 years	2035	1579	39540	0.4128	10.3350
65-69 years	2036	1598	40028	0.4120	10.3198
65-69 years	2037	1614	40454	0.4111	10.3042
65-69 years	2038	1626	40778	0.4102	10.2880
65-69 years	2039	1633	40990	0.4092	10.2716
65-69 years	2040	1639	41154	0.4083	10.2550
70-74 years	2022	1964	39747	0.9223	18.6691
70-74 years	2023	2023	41038	0.9145	18.5537
70-74 years	2024	2078	42262	0.9067	18.4386
70-74 years	2025	2146	43710	0.9064	18.4572

70-74 years	2026	2210	45064	0.9060	18.4759
70-74 years	2027	2267	46295	0.9057	18.4947
70-74 years	2028	2311	47249	0.9054	18.5134
70-74 years	2029	2341	47939	0.9050	18.5323
70-74 years	2030	2356	48269	0.8995	18.4334
70-74 years	2031	2377	48740	0.8941	18.3349
70-74 years	2032	2414	49537	0.8886	18.2367
70-74 years	2033	2475	50839	0.8831	18.1387
70-74 years	2034	2556	52540	0.8777	18.0407
70-74 years	2035	2630	54013	0.8676	17.8188
70-74 years	2036	2698	55369	0.8575	17.5969
70-74 years	2037	2750	56390	0.8474	17.3752
70-74 years	2038	2784	57038	0.8374	17.1538
70-74 years	2039	2807	57447	0.8273	16.9329
70-74 years	2040	2820	57664	0.8173	16.7121
75-79 years	2022	2436	39384	1.7472	28.2463
75-79 years	2023	2555	41446	1.7527	28.4303
75-79 years	2024	2702	43967	1.7581	28.6131
75-79 years	2025	2826	45986	1.7414	28.3414
75-79 years	2026	2946	47948	1.7246	28.0694
75-79 years	2027	3051	49663	1.7079	27.7977
75-79 years	2028	3144	51168	1.6913	27.5268
75-79 years	2029	3231	52590	1.6746	27.2566
75-79 years	2030	3351	54611	1.6783	27.3476
75-79 years	2031	3465	56519	1.6820	27.4387
75-79 years	2032	3568	58274	1.6858	27.5298
75-79 years	2033	3651	59689	1.6895	27.6209

75-79 years	2034	3715	60795	1.6932	27.7122
75-79 years	2035	3756	61492	1.6883	27.6395
75-79 years	2036	3810	62387	1.6834	27.5671
75-79 years	2037	3889	63708	1.6785	27.4951
75-79 years	2038	4009	65696	1.6736	27.4232
75-79 years	2039	4162	68217	1.6687	27.3514
75-79 years	2040	4327	70938	1.6638	27.2794
80-84 years	2022	2941	36945	3.2202	40.4516
80-84 years	2023	3027	38096	3.2360	40.7311
80-84 years	2024	3110	39222	3.2519	41.0120
80-84 years	2025	3188	40282	3.2525	41.0920
80-84 years	2026	3286	41590	3.2529	41.1701
80-84 years	2027	3411	43247	3.2530	41.2449
80-84 years	2028	3579	45457	3.2529	41.3164
80-84 years	2029	3786	48171	3.2526	41.3861
80-84 years	2030	3985	50679	3.2319	41.1033
80-84 years	2031	4179	53120	3.2111	40.8204
80-84 years	2032	4350	55273	3.1903	40.5381
80-84 years	2033	4504	57207	3.1697	40.2571
80-84 years	2034	4655	59091	3.1492	39.9773
80-84 years	2035	4849	61621	3.1626	40.1922
80-84 years	2036	5033	64029	3.1761	40.4073
80-84 years	2037	5202	66254	3.1895	40.6222
80-84 years	2038	5342	68115	3.2029	40.8371
80-84 years	2039	5459	69678	3.2164	41.0527
80-84 years	2040	5576	71238	3.2300	41.2689
85-89 years	2022	2853	28190	5.8522	57.8292

85-89 years	2023	2902	28671	5.7883	57.1776
85-89 years	2024	2959	29218	5.7247	56.5303
85-89 years	2025	3066	30327	5.7474	56.8555
85-89 years	2026	3176	31476	5.7700	57.1801
85-89 years	2027	3286	32622	5.7924	57.5030
85-89 years	2028	3391	33720	5.8150	57.8275
85-89 years	2029	3498	34846	5.8378	58.1547
85-89 years	2030	3614	36063	5.8512	58.3897
85-89 years	2031	3753	37520	5.8642	58.6203
85-89 years	2032	3924	39293	5.8765	58.8440
85-89 years	2033	4147	41597	5.8882	59.0617
85-89 years	2034	4419	44400	5.8996	59.2767
85-89 years	2035	4685	47041	5.8773	59.0140
85-89 years	2036	4943	49600	5.8549	58.7511
85-89 years	2037	5171	51856	5.8327	58.4896
85-89 years	2038	5381	53923	5.8108	58.2306
85-89 years	2039	5591	55992	5.7890	57.9736
85-89 years	2040	5794	57983	5.7673	57.7175
90-94 years	2022	1880	16151	9.6992	83.3252
90-94 years	2023	1909	16365	9.5399	81.7685
90-94 years	2024	1953	16701	9.3816	80.2213
90-94 years	2025	2022	17291	9.3212	79.7110
90-94 years	2026	2087	17852	9.2604	79.1979
90-94 years	2027	2143	18329	9.1990	78.6795
90-94 years	2028	2200	18814	9.1379	78.1636
90-94 years	2029	2267	19395	9.0777	77.6557
90-94 years	2030	2370	20310	9.1288	78.2429

90-94 years	2031	2474	21248	9.1797	78.8273
90-94 years	2032	2577	22169	9.2300	79.4072
90-94 years	2033	2676	23068	9.2804	79.9874
90-94 years	2034	2783	24026	9.3310	80.5698
90-94 years	2035	2899	25080	9.3684	81.0397
90-94 years	2036	3037	26318	9.4052	81.5037
90-94 years	2037	3200	27782	9.4411	81.9600
90-94 years	2038	3410	29654	9.4764	82.4108
90-94 years	2039	3664	31920	9.5114	82.8600
90-94 years	2040	3936	34345	9.5464	83.3083
95+ years	2022	789	6330	14.3590	115.2197
95+ years	2023	814	6522	14.0872	112.8320
95+ years	2024	864	6906	13.8164	110.4535
95+ years	2025	919	7340	13.6731	109.1526
95+ years	2026	962	7671	13.5301	107.8541
95+ years	2027	989	7873	13.3872	106.5565
95+ years	2028	1023	8134	13.2451	105.2649
95+ years	2029	1075	8527	13.1039	103.9815
95+ years	2030	1135	9004	13.0432	103.5086
95+ years	2031	1190	9441	12.9824	103.0355
95+ years	2032	1235	9804	12.9214	102.5612
95+ years	2033	1286	10206	12.8610	102.0922
95+ years	2034	1349	10711	12.8015	101.6306
95+ years	2035	1434	11404	12.8914	102.5545
95+ years	2036	1518	12102	12.9808	103.4734
95+ years	2037	1599	12773	13.0695	104.3871
95+ years	2038	1682	13461	13.1581	105.2999

95+ years	2039	1775	14233	13.2468	106.2130
95+ years	2040	1880	15099	13.3352	107.1235

Abbreviations: CDI, *Clostridioides difficile infections*; DALY, disability-adjusted life-year.

Table S10: The global trends and projections of deaths and DALYs of CDI between 2022 and 2040 across 21 GBD regions.

GBD regions	Year	Number of Deaths	Number of DALYs	ASMRs	ASDRs
Andean Latin America	2022	10	394	0.0162	0.5970

Andean Latin America	2023	11	404	0.0164	0.6028
Andean Latin America	2024	11	415	0.0166	0.6086
Andean Latin America	2025	12	425	0.0167	0.6128
Andean Latin America	2026	12	435	0.0168	0.6170
Andean Latin America	2027	12	444	0.0169	0.6212
Andean Latin America	2028	13	454	0.0170	0.6254
Andean Latin America	2029	13	464	0.0172	0.6296
Andean Latin America	2030	14	472	0.0172	0.6322
Andean Latin America	2031	14	481	0.0173	0.6348
Andean Latin America	2032	15	490	0.0173	0.6374
Andean Latin America	2033	15	499	0.0174	0.6399
Andean Latin America	2034	15	507	0.0175	0.6425
Andean Latin America	2035	16	515	0.0175	0.6433
Andean Latin America	2036	16	523	0.0175	0.6441
Andean Latin America	2037	17	531	0.0175	0.6449
Andean Latin America	2038	17	539	0.0175	0.6457
Andean Latin America	2039	18	547	0.0175	0.6464
Andean Latin America	2040	18	555	0.0175	0.6472
Australasia	2022	131	2112	0.2191	4.0959
Australasia	2023	135	2157	0.2191	4.0625
Australasia	2024	139	2205	0.2192	4.0291
Australasia	2025	144	2253	0.2189	4.0035
Australasia	2026	148	2302	0.2187	3.9779
Australasia	2027	153	2349	0.2185	3.9523
Australasia	2028	158	2397	0.2182	3.9266
Australasia	2029	163	2446	0.2180	3.9010
Australasia	2030	168	2498	0.2176	3.8808

Australasia	2031	173	2550	0.2173	3.8606
Australasia	2032	178	2599	0.2169	3.8403
Australasia	2033	183	2647	0.2165	3.8199
Australasia	2034	188	2695	0.2161	3.7996
Australasia	2035	193	2746	0.2157	3.7850
Australasia	2036	198	2794	0.2152	3.7702
Australasia	2037	202	2841	0.2147	3.7554
Australasia	2038	207	2886	0.2143	3.7405
Australasia	2039	211	2930	0.2138	3.7256
Australasia	2040	216	2972	0.2133	3.7107
Caribbean	2022	6	238	0.0123	0.5264
Caribbean	2023	6	241	0.0124	0.5308
Caribbean	2024	7	244	0.0124	0.5351
Caribbean	2025	7	247	0.0125	0.5379
Caribbean	2026	7	249	0.0125	0.5408
Caribbean	2027	7	251	0.0126	0.5436
Caribbean	2028	7	254	0.0127	0.5465
Caribbean	2029	7	256	0.0127	0.5494
Caribbean	2030	7	258	0.0127	0.5512
Caribbean	2031	8	259	0.0127	0.5530
Caribbean	2032	8	261	0.0128	0.5548
Caribbean	2033	8	262	0.0128	0.5567
Caribbean	2034	8	264	0.0128	0.5585
Caribbean	2035	8	265	0.0128	0.5594
Caribbean	2036	8	266	0.0128	0.5603
Caribbean	2037	8	267	0.0128	0.5612
Caribbean	2038	9	268	0.0128	0.5621

Caribbean	2039	9	269	0.0128	0.5630
Caribbean	2040	9	270	0.0128	0.5639
Central Asia	2022	37	1183	0.0537	1.3947
Central Asia	2023	38	1211	0.0541	1.4041
Central Asia	2024	39	1242	0.0544	1.4136
Central Asia	2025	41	1271	0.0547	1.4202
Central Asia	2026	42	1301	0.0549	1.4269
Central Asia	2027	43	1330	0.0551	1.4335
Central Asia	2028	45	1361	0.0554	1.4401
Central Asia	2029	46	1392	0.0556	1.4468
Central Asia	2030	48	1424	0.0558	1.4521
Central Asia	2031	50	1456	0.0560	1.4574
Central Asia	2032	51	1488	0.0562	1.4627
Central Asia	2033	53	1521	0.0564	1.4680
Central Asia	2034	55	1556	0.0566	1.4733
Central Asia	2035	57	1590	0.0567	1.4773
Central Asia	2036	59	1625	0.0569	1.4812
Central Asia	2037	61	1659	0.0571	1.4852
Central Asia	2038	64	1695	0.0572	1.4891
Central Asia	2039	66	1732	0.0574	1.4931
Central Asia	2040	68	1769	0.0576	1.4970
Central Europe	2022	852	15284	0.3682	7.8759
Central Europe	2023	876	15718	0.3717	7.9472
Central Europe	2024	905	16197	0.3753	8.0196
Central Europe	2025	929	16558	0.3770	8.0595
Central Europe	2026	954	16920	0.3787	8.0996
Central Europe	2027	978	17273	0.3804	8.1395

Central Europe	2028	1004	17642	0.3821	8.1798
Central Europe	2029	1033	18047	0.3839	8.2210
Central Europe	2030	1059	18343	0.3839	8.2188
Central Europe	2031	1085	18638	0.3839	8.2166
Central Europe	2032	1110	18922	0.3839	8.2142
Central Europe	2033	1136	19216	0.3838	8.2120
Central Europe	2034	1166	19539	0.3839	8.2104
Central Europe	2035	1191	19749	0.3823	8.1698
Central Europe	2036	1215	19948	0.3807	8.1290
Central Europe	2037	1236	20122	0.3791	8.0879
Central Europe	2038	1258	20290	0.3775	8.0468
Central Europe	2039	1280	20474	0.3759	8.0059
Central Europe	2040	1303	20661	0.3743	7.9650
Central Latin America	2022	86	2980	0.0331	1.1450
Central Latin America	2023	90	3056	0.0335	1.1558
Central Latin America	2024	94	3134	0.0339	1.1667
Central Latin America	2025	97	3206	0.0341	1.1747
Central Latin America	2026	101	3278	0.0344	1.1828
Central Latin America	2027	105	3350	0.0346	1.1909
Central Latin America	2028	108	3424	0.0348	1.1990
Central Latin America	2029	112	3499	0.0351	1.2071
Central Latin America	2030	116	3565	0.0352	1.2117
Central Latin America	2031	120	3631	0.0353	1.2163
Central Latin America	2032	124	3698	0.0354	1.2209
Central Latin America	2033	128	3765	0.0356	1.2255
Central Latin America	2034	132	3834	0.0357	1.2302
Central Latin America	2035	136	3891	0.0357	1.2311

Central Latin America	2036	140	3949	0.0356	1.2320
Central Latin America	2037	144	4007	0.0356	1.2329
Central Latin America	2038	148	4064	0.0356	1.2339
Central Latin America	2039	152	4123	0.0356	1.2348
Central Latin America	2040	157	4182	0.0356	1.2357
Central Sub-Saharan Africa	2022	27	1182	0.0474	1.2060
Central Sub-Saharan Africa	2023	28	1229	0.0476	1.2183
Central Sub-Saharan Africa	2024	29	1278	0.0478	1.2306
Central Sub-Saharan Africa	2025	30	1325	0.0480	1.2404
Central Sub-Saharan Africa	2026	32	1373	0.0481	1.2503
Central Sub-Saharan Africa	2027	33	1423	0.0483	1.2601
Central Sub-Saharan Africa	2028	34	1474	0.0485	1.2699
Central Sub-Saharan Africa	2029	36	1526	0.0487	1.2798
Central Sub-Saharan Africa	2030	37	1576	0.0488	1.2862
Central Sub-Saharan Africa	2031	38	1627	0.0489	1.2926
Central Sub-Saharan Africa	2032	40	1678	0.0491	1.2990
Central Sub-Saharan Africa	2033	41	1731	0.0492	1.3054
Central Sub-Saharan Africa	2034	43	1785	0.0493	1.3118
Central Sub-Saharan Africa	2035	45	1835	0.0493	1.3146
Central Sub-Saharan Africa	2036	46	1886	0.0493	1.3173
Central Sub-Saharan Africa	2037	48	1937	0.0494	1.3200
Central Sub-Saharan Africa	2038	50	1990	0.0494	1.3227
Central Sub-Saharan Africa	2039	52	2043	0.0494	1.3254
Central Sub-Saharan Africa	2040	54	2098	0.0495	1.3281
East Asia	2022	436	9658	0.0230	0.5643
East Asia	2023	454	9941	0.0233	0.5709
East Asia	2024	474	10240	0.0235	0.5774

East Asia	2025	495	10527	0.0237	0.5824
East Asia	2026	515	10813	0.0239	0.5873
East Asia	2027	535	11094	0.0241	0.5922
East Asia	2028	557	11392	0.0243	0.5971
East Asia	2029	581	11713	0.0245	0.6020
East Asia	2030	604	12006	0.0246	0.6049
East Asia	2031	628	12299	0.0247	0.6078
East Asia	2032	651	12584	0.0248	0.6107
East Asia	2033	676	12882	0.0249	0.6136
East Asia	2034	703	13201	0.0250	0.6165
East Asia	2035	729	13483	0.0250	0.6173
East Asia	2036	755	13758	0.0250	0.6181
East Asia	2037	780	14015	0.0250	0.6189
East Asia	2038	805	14264	0.0250	0.6196
East Asia	2039	831	14518	0.0250	0.6204
East Asia	2040	858	14770	0.0250	0.6212
Eastern Europe	2022	336	7907	0.1021	2.9556
Eastern Europe	2023	343	8031	0.1035	2.9896
Eastern Europe	2024	352	8159	0.1049	3.0234
Eastern Europe	2025	362	8270	0.1065	3.0469
Eastern Europe	2026	372	8381	0.1080	3.0702
Eastern Europe	2027	382	8490	0.1095	3.0937
Eastern Europe	2028	392	8602	0.1111	3.1172
Eastern Europe	2029	404	8721	0.1126	3.1406
Eastern Europe	2030	416	8813	0.1141	3.1558
Eastern Europe	2031	427	8902	0.1156	3.1710
Eastern Europe	2032	438	8985	0.1171	3.1862

Eastern Europe	2033	449	9063	0.1186	3.2013
Eastern Europe	2034	460	9140	0.1201	3.2164
Eastern Europe	2035	470	9189	0.1214	3.2225
Eastern Europe	2036	481	9239	0.1228	3.2286
Eastern Europe	2037	491	9292	0.1241	3.2348
Eastern Europe	2038	502	9350	0.1254	3.2410
Eastern Europe	2039	514	9413	0.1268	3.2471
Eastern Europe	2040	525	9480	0.1281	3.2532
Eastern Sub-Saharan Africa	2022	91	3865	0.0471	1.2067
Eastern Sub-Saharan Africa	2023	94	4023	0.0474	1.2198
Eastern Sub-Saharan Africa	2024	99	4188	0.0478	1.2329
Eastern Sub-Saharan Africa	2025	103	4347	0.0480	1.2434
Eastern Sub-Saharan Africa	2026	107	4511	0.0483	1.2538
Eastern Sub-Saharan Africa	2027	111	4678	0.0486	1.2642
Eastern Sub-Saharan Africa	2028	116	4850	0.0488	1.2747
Eastern Sub-Saharan Africa	2029	121	5027	0.0491	1.2851
Eastern Sub-Saharan Africa	2030	126	5194	0.0492	1.2919
Eastern Sub-Saharan Africa	2031	131	5366	0.0494	1.2986
Eastern Sub-Saharan Africa	2032	136	5540	0.0496	1.3054
Eastern Sub-Saharan Africa	2033	141	5718	0.0497	1.3122
Eastern Sub-Saharan Africa	2034	147	5901	0.0499	1.3190
Eastern Sub-Saharan Africa	2035	153	6070	0.0499	1.3218
Eastern Sub-Saharan Africa	2036	158	6242	0.0500	1.3245
Eastern Sub-Saharan Africa	2037	164	6417	0.0500	1.3273
Eastern Sub-Saharan Africa	2038	170	6596	0.0501	1.3301
Eastern Sub-Saharan Africa	2039	177	6781	0.0501	1.3329
Eastern Sub-Saharan Africa	2040	184	6970	0.0502	1.3358

High-income Asia Pacific	2022	1237	18443	0.2203	5.2300
High-income Asia Pacific	2023	1275	18690	0.2209	5.1911
High-income Asia Pacific	2024	1317	18961	0.2215	5.1526
High-income Asia Pacific	2025	1358	19253	0.2217	5.1218
High-income Asia Pacific	2026	1397	19533	0.2220	5.0908
High-income Asia Pacific	2027	1432	19791	0.2222	5.0595
High-income Asia Pacific	2028	1469	20057	0.2224	5.0281
High-income Asia Pacific	2029	1508	20347	0.2226	4.9968
High-income Asia Pacific	2030	1546	20660	0.2226	4.9732
High-income Asia Pacific	2031	1583	20962	0.2225	4.9493
High-income Asia Pacific	2032	1618	21238	0.2224	4.9253
High-income Asia Pacific	2033	1652	21514	0.2223	4.9013
High-income Asia Pacific	2034	1690	21811	0.2222	4.8774
High-income Asia Pacific	2035	1725	22123	0.2218	4.8600
High-income Asia Pacific	2036	1759	22414	0.2214	4.8425
High-income Asia Pacific	2037	1789	22670	0.2210	4.8249
High-income Asia Pacific	2038	1816	22911	0.2206	4.8073
High-income Asia Pacific	2039	1845	23159	0.2202	4.7897
High-income Asia Pacific	2040	1873	23399	0.2197	4.7722
High-income North America	2022	8094	140143	1.1284	21.8365
High-income North America	2023	8178	140931	1.1088	21.4142
High-income North America	2024	8276	141781	1.0891	20.9918
High-income North America	2025	8426	143510	1.0762	20.7285
High-income North America	2026	8575	145141	1.0634	20.4652
High-income North America	2027	8715	146599	1.0505	20.2019
High-income North America	2028	8858	147960	1.0376	19.9386
High-income North America	2029	9008	149283	1.0247	19.6752

High-income North America	2030	9216	151533	1.0184	19.5478
High-income North America	2031	9417	153639	1.0120	19.4204
High-income North America	2032	9605	155532	1.0057	19.2931
High-income North America	2033	9787	157262	0.9994	19.1657
High-income North America	2034	9969	158877	0.9930	19.0384
High-income North America	2035	10204	161316	0.9925	19.0254
High-income North America	2036	10426	163586	0.9920	19.0124
High-income North America	2037	10629	165645	0.9915	18.9995
High-income North America	2038	10820	167527	0.9910	18.9866
High-income North America	2039	11004	169270	0.9905	18.9738
High-income North America	2040	11174	170853	0.9900	18.9610
North Africa and Middle East	2022	121	4357	0.0249	0.6825
North Africa and Middle East	2023	126	4493	0.0251	0.6893
North Africa and Middle East	2024	132	4633	0.0254	0.6960
North Africa and Middle East	2025	138	4761	0.0255	0.7009
North Africa and Middle East	2026	144	4892	0.0257	0.7058
North Africa and Middle East	2027	149	5023	0.0258	0.7106
North Africa and Middle East	2028	156	5158	0.0260	0.7155
North Africa and Middle East	2029	162	5299	0.0261	0.7204
North Africa and Middle East	2030	169	5426	0.0262	0.7231
North Africa and Middle East	2031	176	5557	0.0263	0.7257
North Africa and Middle East	2032	183	5691	0.0264	0.7284
North Africa and Middle East	2033	190	5829	0.0264	0.7311
North Africa and Middle East	2034	198	5974	0.0265	0.7338
North Africa and Middle East	2035	206	6103	0.0265	0.7341
North Africa and Middle East	2036	214	6235	0.0265	0.7344
North Africa and Middle East	2037	222	6370	0.0265	0.7348

North Africa and Middle East	2038	231	6508	0.0265	0.7351
North Africa and Middle East	2039	240	6653	0.0264	0.7355
North Africa and Middle East	2040	250	6802	0.0264	0.7358
Oceania	2022	2	68	0.0265	0.6501
Oceania	2023	2	70	0.0265	0.6507
Oceania	2024	2	71	0.0265	0.6513
Oceania	2025	2	73	0.0265	0.6514
Oceania	2026	2	75	0.0265	0.6515
Oceania	2027	2	77	0.0265	0.6516
Oceania	2028	2	78	0.0265	0.6517
Oceania	2029	2	80	0.0265	0.6519
Oceania	2030	2	82	0.0265	0.6515
Oceania	2031	2	83	0.0265	0.6512
Oceania	2032	2	85	0.0265	0.6508
Oceania	2033	2	86	0.0264	0.6505
Oceania	2034	2	88	0.0264	0.6501
Oceania	2035	2	90	0.0264	0.6494
Oceania	2036	2	92	0.0263	0.6486
Oceania	2037	3	94	0.0262	0.6479
Oceania	2038	3	95	0.0262	0.6472
Oceania	2039	3	97	0.0261	0.6465
Oceania	2040	3	99	0.0261	0.6457
South Asia	2022	334	12900	0.0229	0.7038
South Asia	2023	345	13202	0.0231	0.7119
South Asia	2024	356	13511	0.0233	0.7200
South Asia	2025	367	13778	0.0234	0.7261
South Asia	2026	378	14045	0.0236	0.7321

South Asia	2027	389	14314	0.0238	0.7381
South Asia	2028	400	14586	0.0239	0.7441
South Asia	2029	412	14865	0.0241	0.7501
South Asia	2030	423	15095	0.0242	0.7535
South Asia	2031	435	15327	0.0242	0.7569
South Asia	2032	446	15563	0.0243	0.7603
South Asia	2033	458	15804	0.0244	0.7637
South Asia	2034	470	16051	0.0245	0.7671
South Asia	2035	482	16241	0.0245	0.7677
South Asia	2036	493	16432	0.0245	0.7682
South Asia	2037	505	16624	0.0245	0.7688
South Asia	2038	516	16818	0.0244	0.7694
South Asia	2039	529	17016	0.0244	0.7699
South Asia	2040	542	17218	0.0244	0.7705
Southeast Asia	2022	136	3972	0.0236	0.6259
Southeast Asia	2023	141	4059	0.0237	0.6271
Southeast Asia	2024	146	4151	0.0237	0.6284
Southeast Asia	2025	151	4244	0.0237	0.6291
Southeast Asia	2026	156	4338	0.0238	0.6299
Southeast Asia	2027	162	4432	0.0238	0.6307
Southeast Asia	2028	167	4529	0.0238	0.6314
Southeast Asia	2029	173	4630	0.0238	0.6322
Southeast Asia	2030	180	4731	0.0238	0.6326
Southeast Asia	2031	186	4832	0.0238	0.6331
Southeast Asia	2032	193	4933	0.0238	0.6335
Southeast Asia	2033	200	5036	0.0239	0.6339
Southeast Asia	2034	207	5141	0.0239	0.6343

Southeast Asia	2035	214	5244	0.0239	0.6344
Southeast Asia	2036	222	5347	0.0239	0.6344
Southeast Asia	2037	229	5448	0.0238	0.6344
Southeast Asia	2038	237	5549	0.0238	0.6345
Southeast Asia	2039	245	5652	0.0238	0.6345
Southeast Asia	2040	253	5755	0.0238	0.6346
Southern Latin America	2022	106	2294	0.1181	2.8331
Southern Latin America	2023	110	2395	0.1203	2.9072
Southern Latin America	2024	115	2501	0.1226	2.9814
Southern Latin America	2025	120	2597	0.1244	3.0412
Southern Latin America	2026	124	2696	0.1262	3.1010
Southern Latin America	2027	129	2796	0.1280	3.1607
Southern Latin America	2028	134	2899	0.1298	3.2204
Southern Latin America	2029	139	3005	0.1316	3.2802
Southern Latin America	2030	144	3096	0.1327	3.3197
Southern Latin America	2031	149	3188	0.1338	3.3591
Southern Latin America	2032	154	3281	0.1349	3.3986
Southern Latin America	2033	159	3376	0.1360	3.4381
Southern Latin America	2034	164	3474	0.1371	3.4776
Southern Latin America	2035	169	3549	0.1374	3.4927
Southern Latin America	2036	174	3626	0.1377	3.5079
Southern Latin America	2037	178	3703	0.1381	3.5230
Southern Latin America	2038	183	3781	0.1384	3.5382
Southern Latin America	2039	188	3861	0.1387	3.5533
Southern Latin America	2040	193	3942	0.1391	3.5685
Southern Sub-Saharan Africa	2022	40	1262	0.0679	1.7648
Southern Sub-Saharan Africa	2023	41	1293	0.0681	1.7696

Southern Sub-Saharan Africa	2024	42	1325	0.0682	1.7744
Southern Sub-Saharan Africa	2025	44	1356	0.0684	1.7793
Southern Sub-Saharan Africa	2026	45	1387	0.0686	1.7843
Southern Sub-Saharan Africa	2027	46	1418	0.0688	1.7892
Southern Sub-Saharan Africa	2028	48	1449	0.0690	1.7941
Southern Sub-Saharan Africa	2029	49	1480	0.0692	1.7991
Southern Sub-Saharan Africa	2030	51	1511	0.0693	1.8019
Southern Sub-Saharan Africa	2031	52	1541	0.0694	1.8046
Southern Sub-Saharan Africa	2032	54	1572	0.0695	1.8074
Southern Sub-Saharan Africa	2033	55	1602	0.0696	1.8102
Southern Sub-Saharan Africa	2034	57	1634	0.0697	1.8131
Southern Sub-Saharan Africa	2035	58	1664	0.0697	1.8136
Southern Sub-Saharan Africa	2036	60	1694	0.0697	1.8141
Southern Sub-Saharan Africa	2037	62	1724	0.0697	1.8146
Southern Sub-Saharan Africa	2038	63	1755	0.0696	1.8151
Southern Sub-Saharan Africa	2039	65	1787	0.0696	1.8156
Southern Sub-Saharan Africa	2040	67	1820	0.0696	1.8162
Tropical Latin America	2022	32	1117	0.0134	0.5329
Tropical Latin America	2023	33	1143	0.0136	0.5384
Tropical Latin America	2024	34	1169	0.0138	0.5438
Tropical Latin America	2025	36	1194	0.0140	0.5490
Tropical Latin America	2026	37	1219	0.0141	0.5541
Tropical Latin America	2027	39	1244	0.0143	0.5593
Tropical Latin America	2028	40	1269	0.0144	0.5645
Tropical Latin America	2029	42	1294	0.0146	0.5697
Tropical Latin America	2030	43	1316	0.0147	0.5734
Tropical Latin America	2031	45	1338	0.0148	0.5772

Tropical Latin America	2032	46	1360	0.0149	0.5809
Tropical Latin America	2033	48	1382	0.0150	0.5847
Tropical Latin America	2034	49	1405	0.0151	0.5884
Tropical Latin America	2035	51	1423	0.0151	0.5900
Tropical Latin America	2036	52	1440	0.0151	0.5915
Tropical Latin America	2037	53	1458	0.0152	0.5931
Tropical Latin America	2038	55	1476	0.0152	0.5946
Tropical Latin America	2039	57	1494	0.0152	0.5962
Tropical Latin America	2040	58	1513	0.0152	0.5978
Western Europe	2022	3943	59662	0.3499	6.8204
Western Europe	2023	4070	61290	0.3548	6.8990
Western Europe	2024	4211	63022	0.3598	6.9776
Western Europe	2025	4343	64654	0.3633	7.0330
Western Europe	2026	4476	66301	0.3669	7.0882
Western Europe	2027	4606	67934	0.3704	7.1434
Western Europe	2028	4740	69617	0.3740	7.1985
Western Europe	2029	4884	71395	0.3775	7.2536
Western Europe	2030	5014	72973	0.3795	7.2831
Western Europe	2031	5146	74565	0.3814	7.3125
Western Europe	2032	5277	76139	0.3833	7.3418
Western Europe	2033	5411	77738	0.3853	7.3711
Western Europe	2034	5554	79400	0.3872	7.4004
Western Europe	2035	5679	80792	0.3875	7.4031
Western Europe	2036	5804	82170	0.3879	7.4057
Western Europe	2037	5926	83504	0.3882	7.4083
Western Europe	2038	6048	84825	0.3885	7.4108
Western Europe	2039	6175	86164	0.3888	7.4134

Western Europe	2040	6304	87499	0.3891	7.4160
Western Sub-Saharan Africa	2022	115	4748	0.0520	1.3243
Western Sub-Saharan Africa	2023	120	4935	0.0523	1.3346
Western Sub-Saharan Africa	2024	125	5131	0.0526	1.3449
Western Sub-Saharan Africa	2025	130	5325	0.0529	1.3526
Western Sub-Saharan Africa	2026	136	5525	0.0531	1.3602
Western Sub-Saharan Africa	2027	141	5731	0.0534	1.3679
Western Sub-Saharan Africa	2028	147	5946	0.0537	1.3757
Western Sub-Saharan Africa	2029	154	6169	0.0539	1.3834
Western Sub-Saharan Africa	2030	160	6386	0.0541	1.3882
Western Sub-Saharan Africa	2031	167	6610	0.0542	1.3929
Western Sub-Saharan Africa	2032	174	6838	0.0543	1.3977
Western Sub-Saharan Africa	2033	181	7073	0.0545	1.4025
Western Sub-Saharan Africa	2034	189	7316	0.0546	1.4074
Western Sub-Saharan Africa	2035	196	7547	0.0546	1.4091
Western Sub-Saharan Africa	2036	204	7783	0.0547	1.4108
Western Sub-Saharan Africa	2037	212	8022	0.0547	1.4125
Western Sub-Saharan Africa	2038	221	8266	0.0547	1.4142
Western Sub-Saharan Africa	2039	230	8517	0.0547	1.4160
Western Sub-Saharan Africa	2040	239	8773	0.0548	1.4177

Abbreviations: CDI, *Clostridioides difficile infections*; GBD, Global Burden of Disease; ASMRs, age-standardized mortality rates; ASDRs, age-standardized DALY rates; DALY, disability-adjusted life-year.

Table S11: The global trends and projections of deaths and DALYs of CDI between 2022 and 2040 across 194 countries and territories.

**Countries/territories**	**Year**	**Number of Deaths**	**Number of DALYs**	**ASMRs**	**ASDRs**
Afghanistan	2022	3	173	0.0172	0.5217
Afghanistan	2023	3	179	0.0174	0.5260

Afghanistan	2024	3	186	0.0176	0.5303
Afghanistan	2025	3	193	0.0178	0.5332
Afghanistan	2026	4	200	0.0179	0.5362
Afghanistan	2027	4	207	0.0180	0.5391
Afghanistan	2028	4	215	0.0181	0.5421
Afghanistan	2029	4	223	0.0182	0.5450
Afghanistan	2030	4	231	0.0183	0.5474
Afghanistan	2031	4	239	0.0184	0.5498
Afghanistan	2032	4	247	0.0185	0.5523
Afghanistan	2033	5	255	0.0186	0.5547
Afghanistan	2034	5	264	0.0187	0.5571
Afghanistan	2035	5	272	0.0188	0.5590
Afghanistan	2036	5	281	0.0189	0.5609
Afghanistan	2037	5	289	0.0190	0.5628
Afghanistan	2038	6	298	0.0191	0.5647
Afghanistan	2039	6	306	0.0192	0.5667
Afghanistan	2040	6	315	0.0193	0.5686
Albania	2022	9	169	0.2105	4.5055
Albania	2023	9	170	0.2076	4.4591
Albania	2024	10	172	0.2048	4.4134
Albania	2025	10	174	0.2028	4.3733
Albania	2026	10	177	0.2008	4.3332
Albania	2027	10	179	0.1988	4.2931
Albania	2028	10	180	0.1968	4.2533
Albania	2029	11	182	0.1948	4.2138
Albania	2030	11	185	0.1935	4.1846
Albania	2031	11	187	0.1921	4.1554
Albania	2032	11	189	0.1907	4.1262

Albania	2033	12	191	0.1893	4.0971
Albania	2034	12	193	0.1880	4.0681
Albania	2035	12	196	0.1871	4.0480
Albania	2036	13	198	0.1863	4.0279
Albania	2037	13	200	0.1854	4.0079
Albania	2038	13	202	0.1846	3.9879
Albania	2039	13	204	0.1837	3.9679
Albania	2040	14	206	0.1829	3.9481
Algeria	2022	9	267	0.0250	0.6511
Algeria	2023	9	276	0.0252	0.6563
Algeria	2024	10	286	0.0254	0.6616
Algeria	2025	10	294	0.0255	0.6652
Algeria	2026	11	303	0.0257	0.6689
Algeria	2027	11	312	0.0258	0.6725
Algeria	2028	11	320	0.0259	0.6762
Algeria	2029	12	329	0.0261	0.6799
Algeria	2030	12	337	0.0262	0.6819
Algeria	2031	13	345	0.0262	0.6839
Algeria	2032	13	354	0.0263	0.6858
Algeria	2033	14	363	0.0264	0.6878
Algeria	2034	14	372	0.0264	0.6898
Algeria	2035	15	380	0.0264	0.6899
Algeria	2036	16	389	0.0264	0.6900
Algeria	2037	16	398	0.0264	0.6901
Algeria	2038	17	407	0.0264	0.6902
Algeria	2039	18	417	0.0264	0.6903
Algeria	2040	19	427	0.0264	0.6903
American Samoa	2022	0	0	0.0359	0.8538

American Samoa	2023	0	0	0.0360	0.8555
American Samoa	2024	0	0	0.0360	0.8572
American Samoa	2025	0	0	0.0361	0.8584
American Samoa	2026	0	0	0.0361	0.8597
American Samoa	2027	0	0	0.0362	0.8610
American Samoa	2028	0	1	0.0362	0.8623
American Samoa	2029	0	1	0.0363	0.8636
American Samoa	2030	0	1	0.0363	0.8643
American Samoa	2031	0	1	0.0363	0.8650
American Samoa	2032	0	1	0.0363	0.8657
American Samoa	2033	0	1	0.0363	0.8664
American Samoa	2034	0	1	0.0364	0.8671
American Samoa	2035	0	1	0.0364	0.8672
American Samoa	2036	0	1	0.0364	0.8673
American Samoa	2037	0	1	0.0363	0.8674
American Samoa	2038	0	1	0.0363	0.8676
American Samoa	2039	0	1	0.0363	0.8677
American Samoa	2040	0	1	0.0363	0.8678
Andorra	2022	0	2	0.0860	1.3849
Andorra	2023	0	2	0.0855	1.3753
Andorra	2024	0	2	0.0850	1.3656
Andorra	2025	0	2	0.0846	1.3585
Andorra	2026	0	2	0.0841	1.3514
Andorra	2027	0	2	0.0837	1.3443
Andorra	2028	0	2	0.0833	1.3372
Andorra	2029	0	2	0.0829	1.3299
Andorra	2030	0	2	0.0826	1.3253
Andorra	2031	0	2	0.0824	1.3207

Andorra	2032	0	3	0.0821	1.3162
Andorra	2033	0	3	0.0818	1.3118
Andorra	2034	0	3	0.0816	1.3075
Andorra	2035	0	3	0.0815	1.3055
Andorra	2036	0	3	0.0814	1.3036
Andorra	2037	0	3	0.0812	1.3017
Andorra	2038	0	3	0.0811	1.2997
Andorra	2039	0	3	0.0810	1.2977
Andorra	2040	0	3	0.0809	1.2958
Angola	2022	7	314	0.0533	1.3582
Angola	2023	7	328	0.0538	1.3738
Angola	2024	8	342	0.0543	1.3894
Angola	2025	8	355	0.0547	1.4014
Angola	2026	8	369	0.0551	1.4134
Angola	2027	9	383	0.0555	1.4255
Angola	2028	9	398	0.0559	1.4376
Angola	2029	10	413	0.0563	1.4497
Angola	2030	10	427	0.0566	1.4577
Angola	2031	10	442	0.0568	1.4656
Angola	2032	11	457	0.0571	1.4736
Angola	2033	11	473	0.0573	1.4816
Angola	2034	12	488	0.0575	1.4896
Angola	2035	12	503	0.0576	1.4930
Angola	2036	13	518	0.0577	1.4965
Angola	2037	13	533	0.0578	1.4999
Angola	2038	14	548	0.0578	1.5033
Angola	2039	15	563	0.0579	1.5068
Angola	2040	15	579	0.0580	1.5102

Antigua and Barbuda	2022	0	0	0.0128	0.4803
Antigua and Barbuda	2023	0	0	0.0129	0.4839
Antigua and Barbuda	2024	0	0	0.0130	0.4875
Antigua and Barbuda	2025	0	0	0.0130	0.4896
Antigua and Barbuda	2026	0	0	0.0130	0.4917
Antigua and Barbuda	2027	0	0	0.0131	0.4937
Antigua and Barbuda	2028	0	0	0.0131	0.4958
Antigua and Barbuda	2029	0	0	0.0132	0.4978
Antigua and Barbuda	2030	0	0	0.0132	0.4990
Antigua and Barbuda	2031	0	0	0.0132	0.5002
Antigua and Barbuda	2032	0	0	0.0132	0.5013
Antigua and Barbuda	2033	0	0	0.0132	0.5025
Antigua and Barbuda	2034	0	0	0.0133	0.5036
Antigua and Barbuda	2035	0	0	0.0133	0.5040
Antigua and Barbuda	2036	0	0	0.0133	0.5043
Antigua and Barbuda	2037	0	0	0.0132	0.5047
Antigua and Barbuda	2038	0	0	0.0132	0.5050
Antigua and Barbuda	2039	0	1	0.0132	0.5053
Antigua and Barbuda	2040	0	1	0.0132	0.5057
Argentina	2022	32	619	0.0546	1.1479
Argentina	2023	33	634	0.0547	1.1584
Argentina	2024	34	650	0.0547	1.1690
Argentina	2025	34	666	0.0548	1.1776
Argentina	2026	35	682	0.0548	1.1861
Argentina	2027	36	698	0.0549	1.1946
Argentina	2028	37	714	0.0549	1.2031
Argentina	2029	38	731	0.0550	1.2117
Argentina	2030	39	746	0.0550	1.2168

Argentina	2031	39	762	0.0550	1.2219
Argentina	2032	40	778	0.0549	1.2269
Argentina	2033	41	793	0.0549	1.2320
Argentina	2034	42	810	0.0549	1.2371
Argentina	2035	43	825	0.0548	1.2384
Argentina	2036	44	839	0.0547	1.2397
Argentina	2037	45	854	0.0547	1.2409
Argentina	2038	46	869	0.0546	1.2422
Argentina	2039	47	885	0.0545	1.2434
Argentina	2040	48	901	0.0544	1.2447
Armenia	2022	2	32	0.0403	0.8594
Armenia	2023	2	32	0.0406	0.8678
Armenia	2024	2	33	0.0410	0.8761
Armenia	2025	2	34	0.0412	0.8818
Armenia	2026	2	34	0.0414	0.8875
Armenia	2027	2	35	0.0416	0.8932
Armenia	2028	2	36	0.0418	0.8989
Armenia	2029	2	36	0.0421	0.9046
Armenia	2030	2	37	0.0422	0.9075
Armenia	2031	2	38	0.0423	0.9103
Armenia	2032	2	38	0.0424	0.9132
Armenia	2033	2	39	0.0425	0.9160
Armenia	2034	2	40	0.0426	0.9188
Armenia	2035	2	40	0.0426	0.9189
Armenia	2036	2	41	0.0425	0.9190
Armenia	2037	2	42	0.0425	0.9191
Armenia	2038	2	42	0.0425	0.9192
Armenia	2039	2	43	0.0425	0.9193

Armenia	2040	3	43	0.0425	0.9193
Australia	2022	89	1413	0.1754	3.2171
Australia	2023	91	1432	0.1746	3.1664
Australia	2024	93	1452	0.1737	3.1158
Australia	2025	96	1476	0.1730	3.0788
Australia	2026	99	1500	0.1722	3.0418
Australia	2027	102	1522	0.1715	3.0048
Australia	2028	104	1545	0.1707	2.9677
Australia	2029	107	1568	0.1700	2.9307
Australia	2030	110	1596	0.1694	2.9047
Australia	2031	113	1623	0.1687	2.8787
Australia	2032	116	1648	0.1681	2.8526
Australia	2033	119	1673	0.1675	2.8266
Australia	2034	123	1697	0.1668	2.8005
Australia	2035	126	1726	0.1663	2.7848
Australia	2036	129	1755	0.1658	2.7691
Australia	2037	132	1781	0.1653	2.7533
Australia	2038	134	1806	0.1648	2.7374
Australia	2039	137	1831	0.1643	2.7216
Australia	2040	140	1855	0.1638	2.7057
Austria	2022	92	1255	0.3916	6.3643
Austria	2023	94	1285	0.3927	6.3574
Austria	2024	98	1318	0.3938	6.3508
Austria	2025	100	1347	0.3931	6.3244
Austria	2026	103	1376	0.3923	6.2977
Austria	2027	106	1404	0.3916	6.2710
Austria	2028	109	1433	0.3908	6.2443
Austria	2029	111	1464	0.3901	6.2177

Austria	2030	114	1490	0.3879	6.1750
Austria	2031	117	1516	0.3857	6.1323
Austria	2032	119	1541	0.3835	6.0895
Austria	2033	121	1566	0.3813	6.0468
Austria	2034	124	1592	0.3791	6.0042
Austria	2035	126	1613	0.3759	5.9502
Austria	2036	128	1635	0.3727	5.8961
Austria	2037	130	1656	0.3694	5.8419
Austria	2038	132	1678	0.3662	5.7878
Austria	2039	134	1702	0.3630	5.7338
Austria	2040	137	1726	0.3597	5.6797
Azerbaijan	2022	5	144	0.0639	1.5643
Azerbaijan	2023	5	148	0.0643	1.5748
Azerbaijan	2024	5	151	0.0646	1.5856
Azerbaijan	2025	5	155	0.0649	1.5935
Azerbaijan	2026	6	159	0.0651	1.6014
Azerbaijan	2027	6	163	0.0654	1.6092
Azerbaijan	2028	6	166	0.0656	1.6171
Azerbaijan	2029	6	171	0.0659	1.6250
Azerbaijan	2030	6	175	0.0660	1.6295
Azerbaijan	2031	7	179	0.0661	1.6340
Azerbaijan	2032	7	183	0.0662	1.6387
Azerbaijan	2033	7	187	0.0663	1.6433
Azerbaijan	2034	8	191	0.0664	1.6478
Azerbaijan	2035	8	195	0.0664	1.6487
Azerbaijan	2036	8	199	0.0663	1.6495
Azerbaijan	2037	8	203	0.0663	1.6503
Azerbaijan	2038	9	207	0.0662	1.6511

Azerbaijan	2039	9	211	0.0662	1.6520
Azerbaijan	2040	9	215	0.0661	1.6528
Bahamas	2022	0	2	0.0148	0.5882
Bahamas	2023	0	2	0.0149	0.5897
Bahamas	2024	0	2	0.0149	0.5912
Bahamas	2025	0	2	0.0149	0.5924
Bahamas	2026	0	2	0.0149	0.5935
Bahamas	2027	0	2	0.0150	0.5946
Bahamas	2028	0	2	0.0150	0.5957
Bahamas	2029	0	2	0.0150	0.5969
Bahamas	2030	0	2	0.0150	0.5976
Bahamas	2031	0	2	0.0151	0.5983
Bahamas	2032	0	2	0.0151	0.5990
Bahamas	2033	0	2	0.0151	0.5998
Bahamas	2034	0	2	0.0151	0.6005
Bahamas	2035	0	2	0.0151	0.6007
Bahamas	2036	0	2	0.0151	0.6009
Bahamas	2037	0	2	0.0151	0.6011
Bahamas	2038	0	2	0.0151	0.6013
Bahamas	2039	0	3	0.0151	0.6016
Bahamas	2040	0	3	0.0151	0.6018
Bahrain	2022	0	10	0.0306	0.7513
Bahrain	2023	0	11	0.0309	0.7574
Bahrain	2024	0	11	0.0312	0.7635
Bahrain	2025	0	12	0.0314	0.7677
Bahrain	2026	0	12	0.0316	0.7718
Bahrain	2027	0	13	0.0318	0.7760
Bahrain	2028	0	13	0.0320	0.7802

Bahrain	2029	1	14	0.0322	0.7845
Bahrain	2030	1	15	0.0323	0.7867
Bahrain	2031	1	15	0.0324	0.7889
Bahrain	2032	1	16	0.0326	0.7909
Bahrain	2033	1	17	0.0327	0.7931
Bahrain	2034	1	17	0.0328	0.7953
Bahrain	2035	1	18	0.0328	0.7953
Bahrain	2036	1	19	0.0329	0.7951
Bahrain	2037	1	20	0.0329	0.7949
Bahrain	2038	1	20	0.0329	0.7947
Bahrain	2039	1	21	0.0329	0.7945
Bahrain	2040	1	22	0.0329	0.7942
Bangladesh	2022	30	953	0.0237	0.6484
Bangladesh	2023	31	972	0.0238	0.6520
Bangladesh	2024	33	991	0.0240	0.6556
Bangladesh	2025	34	1008	0.0240	0.6578
Bangladesh	2026	35	1025	0.0241	0.6600
Bangladesh	2027	36	1042	0.0242	0.6622
Bangladesh	2028	37	1060	0.0243	0.6645
Bangladesh	2029	38	1079	0.0244	0.6668
Bangladesh	2030	40	1095	0.0244	0.6675
Bangladesh	2031	41	1112	0.0244	0.6682
Bangladesh	2032	42	1129	0.0244	0.6690
Bangladesh	2033	44	1146	0.0244	0.6697
Bangladesh	2034	45	1164	0.0244	0.6705
Bangladesh	2035	46	1180	0.0243	0.6696
Bangladesh	2036	48	1196	0.0242	0.6687
Bangladesh	2037	49	1212	0.0242	0.6678

Bangladesh	2038	50	1228	0.0241	0.6670
Bangladesh	2039	52	1244	0.0240	0.6661
Bangladesh	2040	53	1261	0.0239	0.6653
Barbados	2022	0	1	0.0126	0.4790
Barbados	2023	0	1	0.0127	0.4806
Barbados	2024	0	1	0.0127	0.4822
Barbados	2025	0	2	0.0127	0.4834
Barbados	2026	0	2	0.0128	0.4846
Barbados	2027	0	2	0.0128	0.4858
Barbados	2028	0	2	0.0128	0.4870
Barbados	2029	0	2	0.0128	0.4882
Barbados	2030	0	2	0.0128	0.4889
Barbados	2031	0	2	0.0128	0.4895
Barbados	2032	0	2	0.0128	0.4902
Barbados	2033	0	2	0.0129	0.4909
Barbados	2034	0	2	0.0129	0.4915
Barbados	2035	0	2	0.0129	0.4917
Barbados	2036	0	2	0.0129	0.4919
Barbados	2037	0	2	0.0128	0.4921
Barbados	2038	0	2	0.0128	0.4923
Barbados	2039	0	2	0.0128	0.4925
Barbados	2040	0	2	0.0128	0.4927
Belarus	2022	10	272	0.0674	2.2352
Belarus	2023	10	279	0.0676	2.2831
Belarus	2024	10	285	0.0678	2.3309
Belarus	2025	10	293	0.0684	2.3730
Belarus	2026	11	300	0.0690	2.4151
Belarus	2027	11	307	0.0696	2.4572

Belarus	2028	11	314	0.0702	2.4992
Belarus	2029	11	321	0.0708	2.5412
Belarus	2030	12	328	0.0718	2.5849
Belarus	2031	12	335	0.0728	2.6285
Belarus	2032	12	342	0.0739	2.6722
Belarus	2033	12	349	0.0749	2.7158
Belarus	2034	13	356	0.0759	2.7593
Belarus	2035	13	362	0.0773	2.8031
Belarus	2036	13	368	0.0787	2.8469
Belarus	2037	14	375	0.0801	2.8907
Belarus	2038	14	381	0.0814	2.9345
Belarus	2039	14	388	0.0828	2.9782
Belarus	2040	15	394	0.0842	3.0220
Belgium	2022	81	1305	0.2979	6.3832
Belgium	2023	83	1332	0.2999	6.4322
Belgium	2024	85	1361	0.3018	6.4813
Belgium	2025	87	1390	0.3032	6.5158
Belgium	2026	89	1420	0.3045	6.5503
Belgium	2027	91	1449	0.3059	6.5847
Belgium	2028	93	1480	0.3072	6.6191
Belgium	2029	96	1512	0.3085	6.6535
Belgium	2030	98	1543	0.3094	6.6767
Belgium	2031	101	1574	0.3102	6.6999
Belgium	2032	103	1605	0.3110	6.7229
Belgium	2033	106	1636	0.3118	6.7459
Belgium	2034	108	1668	0.3126	6.7689
Belgium	2035	111	1699	0.3130	6.7809
Belgium	2036	114	1728	0.3133	6.7929

Belgium	2037	116	1757	0.3136	6.8047
Belgium	2038	119	1785	0.3139	6.8165
Belgium	2039	122	1814	0.3142	6.8283
Belgium	2040	124	1841	0.3146	6.8400
Belize	2022	0	2	0.0113	0.4822
Belize	2023	0	2	0.0114	0.4859
Belize	2024	0	2	0.0115	0.4895
Belize	2025	0	2	0.0115	0.4921
Belize	2026	0	2	0.0116	0.4947
Belize	2027	0	2	0.0116	0.4973
Belize	2028	0	2	0.0116	0.4999
Belize	2029	0	2	0.0117	0.5025
Belize	2030	0	2	0.0117	0.5040
Belize	2031	0	2	0.0117	0.5056
Belize	2032	0	2	0.0117	0.5071
Belize	2033	0	2	0.0118	0.5087
Belize	2034	0	2	0.0118	0.5102
Belize	2035	0	2	0.0118	0.5107
Belize	2036	0	2	0.0118	0.5112
Belize	2037	0	2	0.0118	0.5117
Belize	2038	0	2	0.0117	0.5123
Belize	2039	0	2	0.0117	0.5128
Belize	2040	0	2	0.0117	0.5133
Benin	2022	2	98	0.0432	1.0598
Benin	2023	3	101	0.0434	1.0667
Benin	2024	3	105	0.0436	1.0736
Benin	2025	3	109	0.0438	1.0790
Benin	2026	3	112	0.0440	1.0843

Benin	2027	3	116	0.0442	1.0897
Benin	2028	3	120	0.0443	1.0951
Benin	2029	3	124	0.0445	1.1005
Benin	2030	3	128	0.0446	1.1037
Benin	2031	3	132	0.0447	1.1069
Benin	2032	4	136	0.0448	1.1101
Benin	2033	4	140	0.0449	1.1133
Benin	2034	4	145	0.0450	1.1164
Benin	2035	4	149	0.0450	1.1175
Benin	2036	4	153	0.0451	1.1185
Benin	2037	4	157	0.0451	1.1195
Benin	2038	4	162	0.0451	1.1205
Benin	2039	5	166	0.0451	1.1215
Benin	2040	5	171	0.0451	1.1226
Bermuda	2022	0	0	0.0131	0.4661
Bermuda	2023	0	0	0.0131	0.4687
Bermuda	2024	0	0	0.0132	0.4713
Bermuda	2025	0	0	0.0132	0.4729
Bermuda	2026	0	0	0.0133	0.4746
Bermuda	2027	0	0	0.0133	0.4763
Bermuda	2028	0	0	0.0134	0.4780
Bermuda	2029	0	0	0.0134	0.4797
Bermuda	2030	0	0	0.0134	0.4806
Bermuda	2031	0	0	0.0134	0.4816
Bermuda	2032	0	0	0.0134	0.4825
Bermuda	2033	0	0	0.0135	0.4835
Bermuda	2034	0	0	0.0135	0.4845
Bermuda	2035	0	0	0.0135	0.4847

Bermuda	2036	0	0	0.0135	0.4850
Bermuda	2037	0	0	0.0135	0.4852
Bermuda	2038	0	0	0.0134	0.4855
Bermuda	2039	0	0	0.0134	0.4858
Bermuda	2040	0	0	0.0134	0.4860
Bhutan	2022	0	6	0.0238	0.6497
Bhutan	2023	0	6	0.0241	0.6556
Bhutan	2024	0	6	0.0243	0.6615
Bhutan	2025	0	6	0.0244	0.6655
Bhutan	2026	0	6	0.0246	0.6695
Bhutan	2027	0	6	0.0247	0.6735
Bhutan	2028	0	7	0.0249	0.6775
Bhutan	2029	0	7	0.0250	0.6815
Bhutan	2030	0	7	0.0251	0.6833
Bhutan	2031	0	7	0.0251	0.6852
Bhutan	2032	0	7	0.0252	0.6870
Bhutan	2033	0	7	0.0252	0.6888
Bhutan	2034	0	7	0.0253	0.6907
Bhutan	2035	0	8	0.0252	0.6902
Bhutan	2036	0	8	0.0252	0.6897
Bhutan	2037	0	8	0.0252	0.6893
Bhutan	2038	0	8	0.0251	0.6888
Bhutan	2039	0	8	0.0251	0.6884
Bhutan	2040	0	8	0.0251	0.6879
Bolivia (Plurinational State of)	2022	2	71	0.0142	0.5591
Bolivia (Plurinational State of)	2023	2	73	0.0142	0.5622
Bolivia (Plurinational State of)	2024	2	74	0.0143	0.5653
Bolivia (Plurinational State of)	2025	2	76	0.0143	0.5674

Bolivia (Plurinational State of)	2026	2	77	0.0144	0.5696
Bolivia (Plurinational State of)	2027	2	79	0.0144	0.5718
Bolivia (Plurinational State of)	2028	2	80	0.0145	0.5740
Bolivia (Plurinational State of)	2029	2	82	0.0145	0.5762
Bolivia (Plurinational State of)	2030	2	83	0.0146	0.5775
Bolivia (Plurinational State of)	2031	2	84	0.0146	0.5788
Bolivia (Plurinational State of)	2032	2	85	0.0146	0.5801
Bolivia (Plurinational State of)	2033	2	86	0.0146	0.5814
Bolivia (Plurinational State of)	2034	2	88	0.0147	0.5827
Bolivia (Plurinational State of)	2035	2	89	0.0147	0.5832
Bolivia (Plurinational State of)	2036	2	90	0.0146	0.5836
Bolivia (Plurinational State of)	2037	2	91	0.0146	0.5841
Bolivia (Plurinational State of)	2038	2	92	0.0146	0.5845
Bolivia (Plurinational State of)	2039	2	94	0.0146	0.5850
Bolivia (Plurinational State of)	2040	2	95	0.0146	0.5854
Bosnia and Herzegovina	2022	16	310	0.2782	5.9723
Bosnia and Herzegovina	2023	17	317	0.2797	6.0103
Bosnia and Herzegovina	2024	17	323	0.2812	6.0488
Bosnia and Herzegovina	2025	18	330	0.2823	6.0770
Bosnia and Herzegovina	2026	18	336	0.2833	6.1051
Bosnia and Herzegovina	2027	18	342	0.2844	6.1330
Bosnia and Herzegovina	2028	19	348	0.2854	6.1607
Bosnia and Herzegovina	2029	19	354	0.2864	6.1886
Bosnia and Herzegovina	2030	20	360	0.2870	6.2065
Bosnia and Herzegovina	2031	20	366	0.2877	6.2246
Bosnia and Herzegovina	2032	21	371	0.2883	6.2425
Bosnia and Herzegovina	2033	21	376	0.2889	6.2603
Bosnia and Herzegovina	2034	22	382	0.2895	6.2781

Bosnia and Herzegovina	2035	22	386	0.2897	6.2857
Bosnia and Herzegovina	2036	23	390	0.2900	6.2932
Bosnia and Herzegovina	2037	23	394	0.2902	6.3007
Bosnia and Herzegovina	2038	24	397	0.2904	6.3081
Bosnia and Herzegovina	2039	24	401	0.2906	6.3156
Bosnia and Herzegovina	2040	24	404	0.2908	6.3229
Botswana	2022	1	35	0.0685	1.7578
Botswana	2023	1	36	0.0691	1.7765
Botswana	2024	1	37	0.0698	1.7953
Botswana	2025	1	38	0.0702	1.8103
Botswana	2026	1	39	0.0707	1.8254
Botswana	2027	1	41	0.0712	1.8405
Botswana	2028	1	42	0.0716	1.8557
Botswana	2029	1	43	0.0721	1.8708
Botswana	2030	1	44	0.0724	1.8809
Botswana	2031	1	46	0.0727	1.8911
Botswana	2032	1	47	0.0730	1.9012
Botswana	2033	2	48	0.0733	1.9114
Botswana	2034	2	50	0.0736	1.9217
Botswana	2035	2	51	0.0737	1.9265
Botswana	2036	2	52	0.0738	1.9313
Botswana	2037	2	53	0.0739	1.9360
Botswana	2038	2	55	0.0740	1.9408
Botswana	2039	2	56	0.0741	1.9456
Botswana	2040	2	58	0.0742	1.9504
Brazil	2022	31	1076	0.0134	0.5313
Brazil	2023	32	1102	0.0136	0.5367
Brazil	2024	33	1128	0.0138	0.5421

Brazil	2025	35	1152	0.0140	0.5473
Brazil	2026	36	1176	0.0141	0.5525
Brazil	2027	38	1200	0.0143	0.5577
Brazil	2028	39	1224	0.0144	0.5629
Brazil	2029	41	1249	0.0146	0.5681
Brazil	2030	42	1271	0.0147	0.5719
Brazil	2031	43	1292	0.0148	0.5757
Brazil	2032	45	1314	0.0149	0.5794
Brazil	2033	46	1335	0.0150	0.5832
Brazil	2034	48	1358	0.0151	0.5870
Brazil	2035	49	1375	0.0151	0.5886
Brazil	2036	51	1392	0.0151	0.5901
Brazil	2037	52	1409	0.0152	0.5917
Brazil	2038	54	1426	0.0152	0.5932
Brazil	2039	55	1444	0.0152	0.5948
Brazil	2040	57	1463	0.0152	0.5963
Brunei Darussalam	2022	1	27	0.2682	6.9300
Brunei Darussalam	2023	1	28	0.2694	6.9513
Brunei Darussalam	2024	1	29	0.2706	6.9735
Brunei Darussalam	2025	1	29	0.2713	6.9836
Brunei Darussalam	2026	1	30	0.2721	6.9935
Brunei Darussalam	2027	1	31	0.2728	7.0029
Brunei Darussalam	2028	1	32	0.2735	7.0122
Brunei Darussalam	2029	1	32	0.2743	7.0219
Brunei Darussalam	2030	1	33	0.2747	7.0240
Brunei Darussalam	2031	1	34	0.2751	7.0258
Brunei Darussalam	2032	1	35	0.2754	7.0270
Brunei Darussalam	2033	1	35	0.2758	7.0278

Brunei Darussalam	2034	1	36	0.2761	7.0285
Brunei Darussalam	2035	1	37	0.2760	7.0201
Brunei Darussalam	2036	1	38	0.2759	7.0117
Brunei Darussalam	2037	2	39	0.2758	7.0032
Brunei Darussalam	2038	2	40	0.2757	6.9946
Brunei Darussalam	2039	2	40	0.2755	6.9861
Brunei Darussalam	2040	2	41	0.2754	6.9777
Bulgaria	2022	65	1138	0.4261	9.2456
Bulgaria	2023	68	1172	0.4387	9.4672
Bulgaria	2024	71	1208	0.4513	9.6895
Bulgaria	2025	73	1236	0.4601	9.8502
Bulgaria	2026	75	1264	0.4689	10.0110
Bulgaria	2027	78	1290	0.4777	10.1719
Bulgaria	2028	80	1318	0.4865	10.3330
Bulgaria	2029	82	1346	0.4954	10.4947
Bulgaria	2030	84	1364	0.4997	10.5775
Bulgaria	2031	86	1382	0.5041	10.6605
Bulgaria	2032	88	1400	0.5085	10.7436
Bulgaria	2033	89	1418	0.5128	10.8269
Bulgaria	2034	91	1437	0.5173	10.9107
Bulgaria	2035	93	1445	0.5168	10.9085
Bulgaria	2036	94	1452	0.5163	10.9062
Bulgaria	2037	95	1459	0.5158	10.9039
Bulgaria	2038	95	1465	0.5154	10.9016
Bulgaria	2039	96	1473	0.5149	10.8996
Bulgaria	2040	97	1480	0.5145	10.8977
Burkina Faso	2022	4	170	0.0397	0.9788
Burkina Faso	2023	4	176	0.0399	0.9848

Burkina Faso	2024	4	183	0.0401	0.9907
Burkina Faso	2025	5	189	0.0402	0.9955
Burkina Faso	2026	5	196	0.0404	1.0004
Burkina Faso	2027	5	203	0.0406	1.0052
Burkina Faso	2028	5	210	0.0407	1.0101
Burkina Faso	2029	5	217	0.0409	1.0149
Burkina Faso	2030	5	225	0.0410	1.0178
Burkina Faso	2031	6	232	0.0411	1.0208
Burkina Faso	2032	6	240	0.0412	1.0237
Burkina Faso	2033	6	248	0.0412	1.0267
Burkina Faso	2034	6	256	0.0413	1.0296
Burkina Faso	2035	7	264	0.0413	1.0306
Burkina Faso	2036	7	272	0.0414	1.0315
Burkina Faso	2037	7	280	0.0414	1.0325
Burkina Faso	2038	7	288	0.0414	1.0335
Burkina Faso	2039	8	296	0.0414	1.0345
Burkina Faso	2040	8	305	0.0414	1.0355
Burundi	2022	2	87	0.0401	0.9974
Burundi	2023	2	90	0.0402	0.9995
Burundi	2024	2	93	0.0403	1.0016
Burundi	2025	2	97	0.0403	1.0032
Burundi	2026	2	100	0.0404	1.0049
Burundi	2027	2	103	0.0405	1.0065
Burundi	2028	2	107	0.0405	1.0081
Burundi	2029	3	111	0.0406	1.0098
Burundi	2030	3	114	0.0407	1.0105
Burundi	2031	3	118	0.0407	1.0113
Burundi	2032	3	122	0.0407	1.0121

Burundi	2033	3	126	0.0407	1.0128
Burundi	2034	3	130	0.0408	1.0136
Burundi	2035	3	134	0.0408	1.0134
Burundi	2036	3	138	0.0408	1.0132
Burundi	2037	3	142	0.0407	1.0130
Burundi	2038	3	146	0.0407	1.0129
Burundi	2039	4	150	0.0407	1.0127
Burundi	2040	4	154	0.0407	1.0126
Cabo Verde	2022	0	6	0.0519	1.2435
Cabo Verde	2023	0	7	0.0528	1.2676
Cabo Verde	2024	0	7	0.0536	1.2917
Cabo Verde	2025	0	7	0.0542	1.3112
Cabo Verde	2026	0	7	0.0548	1.3307
Cabo Verde	2027	0	8	0.0554	1.3501
Cabo Verde	2028	0	8	0.0560	1.3696
Cabo Verde	2029	0	8	0.0566	1.3890
Cabo Verde	2030	0	9	0.0570	1.4021
Cabo Verde	2031	0	9	0.0573	1.4151
Cabo Verde	2032	0	9	0.0577	1.4281
Cabo Verde	2033	0	9	0.0580	1.4411
Cabo Verde	2034	0	10	0.0584	1.4541
Cabo Verde	2035	0	10	0.0585	1.4598
Cabo Verde	2036	0	10	0.0585	1.4655
Cabo Verde	2037	0	11	0.0586	1.4711
Cabo Verde	2038	0	11	0.0587	1.4767
Cabo Verde	2039	0	11	0.0588	1.4823
Cabo Verde	2040	0	11	0.0589	1.4879
Cambodia	2022	2	72	0.0187	0.4973

Cambodia	2023	2	74	0.0187	0.4983
Cambodia	2024	2	76	0.0188	0.4994
Cambodia	2025	2	77	0.0188	0.5002
Cambodia	2026	2	79	0.0188	0.5010
Cambodia	2027	2	80	0.0188	0.5018
Cambodia	2028	3	81	0.0189	0.5026
Cambodia	2029	3	83	0.0189	0.5034
Cambodia	2030	3	84	0.0189	0.5039
Cambodia	2031	3	86	0.0189	0.5043
Cambodia	2032	3	87	0.0189	0.5047
Cambodia	2033	3	89	0.0189	0.5051
Cambodia	2034	3	90	0.0189	0.5055
Cambodia	2035	3	92	0.0189	0.5055
Cambodia	2036	3	93	0.0189	0.5055
Cambodia	2037	3	95	0.0189	0.5055
Cambodia	2038	4	96	0.0189	0.5055
Cambodia	2039	4	98	0.0189	0.5055
Cambodia	2040	4	99	0.0189	0.5055
Cameroon	2022	7	289	0.0528	1.3376
Cameroon	2023	7	297	0.0531	1.3463
Cameroon	2024	8	307	0.0534	1.3550
Cameroon	2025	8	315	0.0536	1.3615
Cameroon	2026	8	324	0.0538	1.3681
Cameroon	2027	8	333	0.0540	1.3746
Cameroon	2028	9	343	0.0542	1.3812
Cameroon	2029	9	352	0.0544	1.3878
Cameroon	2030	9	362	0.0546	1.3917
Cameroon	2031	10	371	0.0547	1.3956

Cameroon	2032	10	380	0.0548	1.3995
Cameroon	2033	11	390	0.0549	1.4035
Cameroon	2034	11	400	0.0550	1.4074
Cameroon	2035	11	410	0.0550	1.4086
Cameroon	2036	12	419	0.0550	1.4099
Cameroon	2037	12	429	0.0551	1.4111
Cameroon	2038	13	439	0.0551	1.4124
Cameroon	2039	13	449	0.0551	1.4136
Cameroon	2040	13	460	0.0551	1.4149
Canada	2022	570	9501	0.6987	13.6453
Canada	2023	591	9745	0.7018	13.6478
Canada	2024	613	10000	0.7049	13.6501
Canada	2025	636	10266	0.7073	13.6386
Canada	2026	659	10531	0.7097	13.6270
Canada	2027	683	10791	0.7121	13.6154
Canada	2028	707	11051	0.7145	13.6037
Canada	2029	733	11319	0.7169	13.5920
Canada	2030	759	11574	0.7188	13.5690
Canada	2031	785	11823	0.7206	13.5460
Canada	2032	811	12061	0.7225	13.5230
Canada	2033	837	12291	0.7243	13.5000
Canada	2034	864	12520	0.7262	13.4771
Canada	2035	891	12727	0.7277	13.4422
Canada	2036	917	12923	0.7292	13.4074
Canada	2037	941	13103	0.7307	13.3726
Canada	2038	966	13272	0.7322	13.3380
Canada	2039	990	13432	0.7337	13.3035
Canada	2040	1013	13582	0.7353	13.2690

Central African Republic	2022	1	37	0.0433	1.1046
Central African Republic	2023	1	37	0.0434	1.1076
Central African Republic	2024	1	38	0.0436	1.1106
Central African Republic	2025	1	38	0.0436	1.1127
Central African Republic	2026	1	39	0.0437	1.1148
Central African Republic	2027	1	39	0.0438	1.1169
Central African Republic	2028	1	39	0.0439	1.1191
Central African Republic	2029	1	40	0.0440	1.1212
Central African Republic	2030	1	40	0.0440	1.1221
Central African Republic	2031	1	41	0.0440	1.1231
Central African Republic	2032	1	41	0.0441	1.1240
Central African Republic	2033	1	42	0.0441	1.1249
Central African Republic	2034	1	42	0.0441	1.1258
Central African Republic	2035	1	43	0.0441	1.1255
Central African Republic	2036	1	43	0.0441	1.1251
Central African Republic	2037	1	44	0.0441	1.1248
Central African Republic	2038	1	44	0.0441	1.1245
Central African Republic	2039	1	45	0.0441	1.1241
Central African Republic	2040	1	45	0.0440	1.1237
Chad	2022	3	118	0.0383	0.9414
Chad	2023	3	123	0.0385	0.9452
Chad	2024	3	128	0.0386	0.9491
Chad	2025	3	133	0.0387	0.9520
Chad	2026	3	139	0.0388	0.9550
Chad	2027	3	144	0.0389	0.9581
Chad	2028	3	150	0.0390	0.9611
Chad	2029	3	156	0.0391	0.9641
Chad	2030	4	163	0.0392	0.9656

Chad	2031	4	169	0.0392	0.9671
Chad	2032	4	176	0.0393	0.9686
Chad	2033	4	183	0.0393	0.9701
Chad	2034	4	190	0.0394	0.9716
Chad	2035	4	198	0.0394	0.9716
Chad	2036	4	205	0.0393	0.9715
Chad	2037	5	213	0.0393	0.9715
Chad	2038	5	221	0.0393	0.9714
Chad	2039	5	229	0.0393	0.9714
Chad	2040	5	237	0.0393	0.9714
Chile	2022	68	1545	0.2680	6.8588
Chile	2023	72	1629	0.2757	7.0722
Chile	2024	76	1717	0.2834	7.2857
Chile	2025	81	1797	0.2892	7.4555
Chile	2026	85	1879	0.2950	7.6253
Chile	2027	89	1962	0.3007	7.7951
Chile	2028	94	2047	0.3065	7.9650
Chile	2029	99	2136	0.3123	8.1348
Chile	2030	103	2212	0.3157	8.2469
Chile	2031	107	2288	0.3191	8.3589
Chile	2032	112	2364	0.3225	8.4709
Chile	2033	116	2441	0.3259	8.5829
Chile	2034	121	2520	0.3293	8.6950
Chile	2035	125	2580	0.3301	8.7390
Chile	2036	129	2640	0.3310	8.7831
Chile	2037	133	2698	0.3318	8.8271
Chile	2038	137	2756	0.3327	8.8711
Chile	2039	141	2815	0.3335	8.9152

Chile	2040	146	2873	0.3343	8.9592
China	2022	365	8307	0.0202	0.5163
China	2023	380	8560	0.0205	0.5229
China	2024	397	8828	0.0207	0.5296
China	2025	415	9082	0.0209	0.5347
China	2026	433	9338	0.0211	0.5399
China	2027	451	9591	0.0214	0.5451
China	2028	470	9860	0.0216	0.5502
China	2029	490	10149	0.0218	0.5554
China	2030	511	10415	0.0219	0.5588
China	2031	532	10683	0.0221	0.5622
China	2032	553	10945	0.0222	0.5656
China	2033	575	11220	0.0224	0.5690
China	2034	599	11515	0.0225	0.5725
China	2035	622	11775	0.0226	0.5739
China	2036	646	12031	0.0227	0.5754
China	2037	668	12272	0.0228	0.5769
China	2038	690	12507	0.0228	0.5784
China	2039	714	12745	0.0229	0.5799
China	2040	737	12983	0.0230	0.5814
Colombia	2022	20	652	0.0343	1.2475
Colombia	2023	21	666	0.0345	1.2543
Colombia	2024	22	680	0.0347	1.2611
Colombia	2025	23	694	0.0348	1.2663
Colombia	2026	24	707	0.0349	1.2716
Colombia	2027	25	721	0.0351	1.2769
Colombia	2028	25	735	0.0352	1.2821
Colombia	2029	26	749	0.0354	1.2874

Colombia	2030	27	763	0.0355	1.2912
Colombia	2031	28	777	0.0356	1.2951
Colombia	2032	29	791	0.0357	1.2989
Colombia	2033	30	804	0.0358	1.3028
Colombia	2034	31	818	0.0359	1.3066
Colombia	2035	32	831	0.0360	1.3088
Colombia	2036	33	844	0.0360	1.3111
Colombia	2037	34	856	0.0361	1.3133
Colombia	2038	35	869	0.0361	1.3156
Colombia	2039	36	881	0.0362	1.3178
Colombia	2040	37	894	0.0363	1.3201
Comoros	2022	0	7	0.0491	1.1999
Comoros	2023	0	7	0.0494	1.2069
Comoros	2024	0	8	0.0496	1.2139
Comoros	2025	0	8	0.0499	1.2206
Comoros	2026	0	8	0.0501	1.2273
Comoros	2027	0	8	0.0504	1.2340
Comoros	2028	0	8	0.0506	1.2407
Comoros	2029	0	9	0.0508	1.2474
Comoros	2030	0	9	0.0510	1.2516
Comoros	2031	0	9	0.0511	1.2558
Comoros	2032	0	9	0.0513	1.2601
Comoros	2033	0	9	0.0514	1.2643
Comoros	2034	0	10	0.0515	1.2685
Comoros	2035	0	10	0.0516	1.2702
Comoros	2036	0	10	0.0516	1.2719
Comoros	2037	0	10	0.0517	1.2736
Comoros	2038	0	11	0.0517	1.2753

Comoros	2039	0	11	0.0517	1.2770
Comoros	2040	0	11	0.0518	1.2786
Congo	2022	2	59	0.0603	1.5239
Congo	2023	2	60	0.0606	1.5333
Congo	2024	2	61	0.0610	1.5427
Congo	2025	2	63	0.0613	1.5508
Congo	2026	2	64	0.0616	1.5588
Congo	2027	2	66	0.0619	1.5668
Congo	2028	2	67	0.0622	1.5748
Congo	2029	2	69	0.0625	1.5828
Congo	2030	2	70	0.0626	1.5881
Congo	2031	2	72	0.0628	1.5933
Congo	2032	2	74	0.0630	1.5986
Congo	2033	2	75	0.0632	1.6038
Congo	2034	2	77	0.0634	1.6090
Congo	2035	2	79	0.0634	1.6110
Congo	2036	2	81	0.0635	1.6130
Congo	2037	3	83	0.0635	1.6150
Congo	2038	3	85	0.0636	1.6169
Congo	2039	3	87	0.0636	1.6188
Congo	2040	3	89	0.0637	1.6207
Costa Rica	2022	2	56	0.0350	1.1518
Costa Rica	2023	2	57	0.0352	1.1587
Costa Rica	2024	2	58	0.0354	1.1657
Costa Rica	2025	2	60	0.0356	1.1714
Costa Rica	2026	2	61	0.0357	1.1771
Costa Rica	2027	2	63	0.0359	1.1829
Costa Rica	2028	2	64	0.0361	1.1887

Costa Rica	2029	2	66	0.0362	1.1945
Costa Rica	2030	3	67	0.0363	1.1983
Costa Rica	2031	3	68	0.0364	1.2020
Costa Rica	2032	3	70	0.0365	1.2058
Costa Rica	2033	3	71	0.0366	1.2096
Costa Rica	2034	3	72	0.0367	1.2134
Costa Rica	2035	3	74	0.0367	1.2151
Costa Rica	2036	3	75	0.0367	1.2168
Costa Rica	2037	3	76	0.0368	1.2186
Costa Rica	2038	3	77	0.0368	1.2203
Costa Rica	2039	3	78	0.0368	1.2220
Costa Rica	2040	3	79	0.0368	1.2238
Croatia	2022	38	639	0.3980	8.2248
Croatia	2023	39	658	0.4064	8.3670
Croatia	2024	40	678	0.4149	8.5104
Croatia	2025	42	695	0.4212	8.6214
Croatia	2026	43	712	0.4274	8.7322
Croatia	2027	44	728	0.4337	8.8427
Croatia	2028	45	746	0.4400	8.9538
Croatia	2029	47	764	0.4463	9.0657
Croatia	2030	48	778	0.4498	9.1292
Croatia	2031	49	793	0.4533	9.1927
Croatia	2032	50	807	0.4568	9.2559
Croatia	2033	52	821	0.4603	9.3194
Croatia	2034	53	836	0.4638	9.3834
Croatia	2035	54	847	0.4643	9.3960
Croatia	2036	55	857	0.4647	9.4084
Croatia	2037	56	866	0.4652	9.4204

Croatia	2038	57	875	0.4656	9.4325
Croatia	2039	58	884	0.4661	9.4448
Croatia	2040	59	893	0.4665	9.4570
Cuba	2022	2	46	0.0106	0.3978
Cuba	2023	2	47	0.0106	0.3990
Cuba	2024	2	47	0.0106	0.4001
Cuba	2025	2	47	0.0107	0.4007
Cuba	2026	2	48	0.0107	0.4013
Cuba	2027	2	48	0.0107	0.4019
Cuba	2028	2	49	0.0107	0.4025
Cuba	2029	2	49	0.0107	0.4032
Cuba	2030	2	49	0.0107	0.4036
Cuba	2031	2	50	0.0107	0.4040
Cuba	2032	2	50	0.0107	0.4044
Cuba	2033	2	51	0.0107	0.4048
Cuba	2034	2	51	0.0108	0.4052
Cuba	2035	2	52	0.0108	0.4053
Cuba	2036	2	52	0.0107	0.4055
Cuba	2037	2	52	0.0107	0.4056
Cuba	2038	3	52	0.0107	0.4057
Cuba	2039	3	53	0.0107	0.4059
Cuba	2040	3	53	0.0107	0.4060
Cyprus	2022	7	116	0.2973	6.0872
Cyprus	2023	7	120	0.2992	6.1348
Cyprus	2024	7	124	0.3010	6.1825
Cyprus	2025	8	129	0.3024	6.2171
Cyprus	2026	8	133	0.3038	6.2516
Cyprus	2027	8	137	0.3051	6.2857

Cyprus	2028	9	141	0.3065	6.3199
Cyprus	2029	9	146	0.3078	6.3540
Cyprus	2030	9	150	0.3087	6.3757
Cyprus	2031	10	154	0.3095	6.3972
Cyprus	2032	10	158	0.3103	6.4187
Cyprus	2033	10	163	0.3111	6.4401
Cyprus	2034	11	167	0.3119	6.4616
Cyprus	2035	11	171	0.3122	6.4705
Cyprus	2036	12	175	0.3125	6.4795
Cyprus	2037	12	179	0.3128	6.4885
Cyprus	2038	12	184	0.3131	6.4977
Cyprus	2039	13	188	0.3134	6.5069
Cyprus	2040	13	192	0.3137	6.5163
Czechia	2022	88	1466	0.3852	7.7194
Czechia	2023	91	1471	0.3884	7.5487
Czechia	2024	95	1478	0.3916	7.3786
Czechia	2025	98	1484	0.3930	7.2652
Czechia	2026	101	1489	0.3943	7.1515
Czechia	2027	104	1493	0.3956	7.0373
Czechia	2028	107	1495	0.3969	6.9231
Czechia	2029	110	1499	0.3983	6.8090
Czechia	2030	113	1506	0.3982	6.7300
Czechia	2031	116	1512	0.3981	6.6508
Czechia	2032	118	1516	0.3980	6.5711
Czechia	2033	121	1520	0.3979	6.4913
Czechia	2034	124	1524	0.3978	6.4115
Czechia	2035	126	1534	0.3966	6.3673
Czechia	2036	128	1542	0.3954	6.3228

Czechia	2037	130	1548	0.3941	6.2781
Czechia	2038	131	1554	0.3929	6.2332
Czechia	2039	133	1560	0.3916	6.1882
Czechia	2040	135	1566	0.3904	6.1432
Democratic People's Republic of Korea	2022	11	255	0.0391	0.9055
Democratic People's Republic of Korea	2023	12	261	0.0393	0.9111
Democratic People's Republic of Korea	2024	12	267	0.0396	0.9168
Democratic People's Republic of Korea	2025	12	273	0.0398	0.9210
Democratic People's Republic of Korea	2026	13	279	0.0399	0.9251
Democratic People's Republic of Korea	2027	13	285	0.0401	0.9291
Democratic People's Republic of Korea	2028	14	291	0.0403	0.9330
Democratic People's Republic of Korea	2029	14	297	0.0405	0.9371
Democratic People's Republic of Korea	2030	14	303	0.0406	0.9394
Democratic People's Republic of Korea	2031	15	309	0.0407	0.9416
Democratic People's Republic of Korea	2032	15	315	0.0408	0.9438
Democratic People's Republic of Korea	2033	15	321	0.0408	0.9460
Democratic People's Republic of Korea	2034	16	327	0.0409	0.9482
Democratic People's Republic of Korea	2035	16	332	0.0409	0.9486
Democratic People's Republic of Korea	2036	17	337	0.0410	0.9490
Democratic People's Republic of Korea	2037	17	342	0.0410	0.9494
Democratic People's Republic of Korea	2038	17	347	0.0410	0.9498
Democratic People's Republic of Korea	2039	18	352	0.0410	0.9501
Democratic People's Republic of Korea	2040	18	357	0.0410	0.9505
Democratic Republic of the Congo	2022	17	728	0.0440	1.1160
Democratic Republic of the Congo	2023	17	758	0.0441	1.1272
Democratic Republic of the Congo	2024	18	788	0.0442	1.1385
Democratic Republic of the Congo	2025	19	818	0.0443	1.1476
Democratic Republic of the Congo	2026	19	849	0.0444	1.1566

Democratic Republic of the Congo	2027	20	880	0.0445	1.1656
Democratic Republic of the Congo	2028	21	913	0.0447	1.1746
Democratic Republic of the Congo	2029	22	946	0.0448	1.1837
Democratic Republic of the Congo	2030	22	977	0.0448	1.1895
Democratic Republic of the Congo	2031	23	1009	0.0449	1.1953
Democratic Republic of the Congo	2032	24	1041	0.0450	1.2011
Democratic Republic of the Congo	2033	25	1074	0.0450	1.2069
Democratic Republic of the Congo	2034	26	1108	0.0451	1.2127
Democratic Republic of the Congo	2035	27	1139	0.0451	1.2151
Democratic Republic of the Congo	2036	28	1171	0.0451	1.2174
Democratic Republic of the Congo	2037	29	1203	0.0451	1.2198
Democratic Republic of the Congo	2038	30	1236	0.0451	1.2221
Democratic Republic of the Congo	2039	31	1269	0.0452	1.2245
Democratic Republic of the Congo	2040	32	1303	0.0452	1.2268
Denmark	2022	39	680	0.3146	7.2218
Denmark	2023	40	696	0.3170	7.3067
Denmark	2024	42	714	0.3194	7.3918
Denmark	2025	43	732	0.3211	7.4519
Denmark	2026	44	750	0.3228	7.5119
Denmark	2027	46	767	0.3244	7.5718
Denmark	2028	47	784	0.3261	7.6317
Denmark	2029	49	803	0.3277	7.6917
Denmark	2030	50	820	0.3287	7.7330
Denmark	2031	51	836	0.3296	7.7743
Denmark	2032	53	851	0.3306	7.8155
Denmark	2033	54	865	0.3315	7.8566
Denmark	2034	55	880	0.3324	7.8978
Denmark	2035	56	892	0.3326	7.9189

Denmark	2036	57	903	0.3328	7.9399
Denmark	2037	58	913	0.3330	7.9609
Denmark	2038	59	921	0.3331	7.9819
Denmark	2039	60	930	0.3333	8.0029
Denmark	2040	60	938	0.3335	8.0239
Djibouti	2022	0	11	0.0481	1.1866
Djibouti	2023	0	11	0.0483	1.1948
Djibouti	2024	0	11	0.0486	1.2029
Djibouti	2025	0	12	0.0488	1.2091
Djibouti	2026	0	12	0.0490	1.2153
Djibouti	2027	0	12	0.0492	1.2215
Djibouti	2028	0	13	0.0494	1.2275
Djibouti	2029	0	13	0.0497	1.2335
Djibouti	2030	0	13	0.0498	1.2372
Djibouti	2031	0	14	0.0499	1.2408
Djibouti	2032	0	14	0.0500	1.2443
Djibouti	2033	0	14	0.0501	1.2477
Djibouti	2034	0	15	0.0502	1.2511
Djibouti	2035	0	15	0.0502	1.2522
Djibouti	2036	1	15	0.0503	1.2531
Djibouti	2037	1	16	0.0503	1.2541
Djibouti	2038	1	16	0.0503	1.2550
Djibouti	2039	1	16	0.0503	1.2559
Djibouti	2040	1	17	0.0503	1.2569
Dominica	2022	0	0	0.0142	0.5675
Dominica	2023	0	0	0.0143	0.5725
Dominica	2024	0	0	0.0144	0.5774
Dominica	2025	0	0	0.0145	0.5808

Dominica	2026	0	0	0.0145	0.5842
Dominica	2027	0	0	0.0146	0.5876
Dominica	2028	0	0	0.0147	0.5909
Dominica	2029	0	0	0.0148	0.5943
Dominica	2030	0	0	0.0148	0.5964
Dominica	2031	0	0	0.0148	0.5984
Dominica	2032	0	0	0.0149	0.6005
Dominica	2033	0	0	0.0149	0.6025
Dominica	2034	0	0	0.0149	0.6045
Dominica	2035	0	0	0.0149	0.6052
Dominica	2036	0	0	0.0149	0.6059
Dominica	2037	0	0	0.0149	0.6066
Dominica	2038	0	0	0.0149	0.6073
Dominica	2039	0	0	0.0149	0.6080
Dominica	2040	0	0	0.0149	0.6087
Dominican Republic	2022	1	52	0.0114	0.5052
Dominican Republic	2023	1	53	0.0115	0.5091
Dominican Republic	2024	1	54	0.0115	0.5131
Dominican Republic	2025	1	54	0.0116	0.5161
Dominican Republic	2026	1	55	0.0117	0.5191
Dominican Republic	2027	1	55	0.0117	0.5221
Dominican Republic	2028	1	56	0.0118	0.5251
Dominican Republic	2029	1	56	0.0118	0.5281
Dominican Republic	2030	1	56	0.0119	0.5300
Dominican Republic	2031	1	57	0.0119	0.5319
Dominican Republic	2032	1	57	0.0119	0.5339
Dominican Republic	2033	1	57	0.0119	0.5358
Dominican Republic	2034	1	57	0.0120	0.5377

Dominican Republic	2035	1	58	0.0120	0.5386
Dominican Republic	2036	2	58	0.0120	0.5394
Dominican Republic	2037	2	58	0.0120	0.5403
Dominican Republic	2038	2	58	0.0120	0.5411
Dominican Republic	2039	2	58	0.0120	0.5420
Dominican Republic	2040	2	58	0.0120	0.5428
Ecuador	2022	4	131	0.0236	0.7727
Ecuador	2023	4	136	0.0242	0.7901
Ecuador	2024	4	142	0.0247	0.8074
Ecuador	2025	5	147	0.0251	0.8207
Ecuador	2026	5	152	0.0255	0.8340
Ecuador	2027	5	157	0.0259	0.8473
Ecuador	2028	5	162	0.0263	0.8607
Ecuador	2029	5	167	0.0267	0.8740
Ecuador	2030	6	171	0.0269	0.8820
Ecuador	2031	6	176	0.0272	0.8900
Ecuador	2032	6	180	0.0274	0.8980
Ecuador	2033	6	185	0.0276	0.9060
Ecuador	2034	6	189	0.0278	0.9140
Ecuador	2035	7	192	0.0278	0.9158
Ecuador	2036	7	196	0.0278	0.9175
Ecuador	2037	7	199	0.0278	0.9192
Ecuador	2038	7	202	0.0278	0.9209
Ecuador	2039	7	205	0.0278	0.9227
Ecuador	2040	7	209	0.0278	0.9244
Egypt	2022	15	590	0.0254	0.6851
Egypt	2023	15	608	0.0256	0.6908
Egypt	2024	16	626	0.0258	0.6965

Egypt	2025	16	643	0.0259	0.7003
Egypt	2026	17	660	0.0261	0.7041
Egypt	2027	18	678	0.0262	0.7078
Egypt	2028	18	696	0.0263	0.7116
Egypt	2029	19	714	0.0264	0.7154
Egypt	2030	20	731	0.0265	0.7173
Egypt	2031	21	748	0.0265	0.7192
Egypt	2032	22	766	0.0266	0.7210
Egypt	2033	22	784	0.0266	0.7229
Egypt	2034	23	802	0.0266	0.7249
Egypt	2035	24	819	0.0266	0.7247
Egypt	2036	25	835	0.0266	0.7245
Egypt	2037	26	852	0.0265	0.7244
Egypt	2038	27	869	0.0265	0.7242
Egypt	2039	28	886	0.0264	0.7241
Egypt	2040	29	904	0.0264	0.7240
El Salvador	2022	2	71	0.0324	1.2246
El Salvador	2023	2	72	0.0326	1.2359
El Salvador	2024	2	73	0.0329	1.2472
El Salvador	2025	2	74	0.0330	1.2553
El Salvador	2026	2	75	0.0332	1.2635
El Salvador	2027	2	75	0.0334	1.2717
El Salvador	2028	2	76	0.0336	1.2799
El Salvador	2029	2	77	0.0337	1.2882
El Salvador	2030	2	77	0.0338	1.2937
El Salvador	2031	2	78	0.0339	1.2992
El Salvador	2032	2	78	0.0340	1.3047
El Salvador	2033	2	78	0.0341	1.3102

El Salvador	2034	2	79	0.0342	1.3158
El Salvador	2035	3	79	0.0343	1.3186
El Salvador	2036	3	79	0.0343	1.3215
El Salvador	2037	3	80	0.0343	1.3244
El Salvador	2038	3	80	0.0343	1.3274
El Salvador	2039	3	80	0.0344	1.3303
El Salvador	2040	3	80	0.0344	1.3333
Equatorial Guinea	2022	0	21	0.0714	1.9045
Equatorial Guinea	2023	0	22	0.0728	1.9481
Equatorial Guinea	2024	1	23	0.0741	1.9918
Equatorial Guinea	2025	1	24	0.0751	2.0262
Equatorial Guinea	2026	1	25	0.0761	2.0606
Equatorial Guinea	2027	1	26	0.0771	2.0950
Equatorial Guinea	2028	1	27	0.0781	2.1293
Equatorial Guinea	2029	1	29	0.0792	2.1637
Equatorial Guinea	2030	1	30	0.0798	2.1874
Equatorial Guinea	2031	1	31	0.0804	2.2110
Equatorial Guinea	2032	1	32	0.0811	2.2345
Equatorial Guinea	2033	1	33	0.0817	2.2578
Equatorial Guinea	2034	1	35	0.0823	2.2812
Equatorial Guinea	2035	1	36	0.0826	2.2928
Equatorial Guinea	2036	1	37	0.0828	2.3043
Equatorial Guinea	2037	1	38	0.0830	2.3157
Equatorial Guinea	2038	1	39	0.0833	2.3271
Equatorial Guinea	2039	1	41	0.0835	2.3385
Equatorial Guinea	2040	1	42	0.0837	2.3499
Eritrea	2022	1	51	0.0478	1.1899
Eritrea	2023	1	52	0.0482	1.1987

Eritrea	2024	1	54	0.0485	1.2074
Eritrea	2025	1	55	0.0487	1.2139
Eritrea	2026	1	57	0.0489	1.2203
Eritrea	2027	1	58	0.0491	1.2268
Eritrea	2028	1	60	0.0493	1.2333
Eritrea	2029	1	61	0.0496	1.2398
Eritrea	2030	2	63	0.0497	1.2439
Eritrea	2031	2	64	0.0498	1.2481
Eritrea	2032	2	66	0.0500	1.2522
Eritrea	2033	2	67	0.0501	1.2562
Eritrea	2034	2	69	0.0502	1.2603
Eritrea	2035	2	70	0.0503	1.2619
Eritrea	2036	2	72	0.0503	1.2634
Eritrea	2037	2	74	0.0503	1.2649
Eritrea	2038	2	75	0.0504	1.2663
Eritrea	2039	2	77	0.0504	1.2678
Eritrea	2040	2	78	0.0504	1.2693
Estonia	2022	1	34	0.0577	1.8406
Estonia	2023	1	35	0.0575	1.8978
Estonia	2024	1	36	0.0574	1.9545
Estonia	2025	1	37	0.0574	1.9996
Estonia	2026	1	38	0.0575	2.0445
Estonia	2027	2	39	0.0575	2.0891
Estonia	2028	2	40	0.0575	2.1336
Estonia	2029	2	41	0.0575	2.1778
Estonia	2030	2	42	0.0579	2.2191
Estonia	2031	2	43	0.0583	2.2602
Estonia	2032	2	44	0.0587	2.3013

Estonia	2033	2	45	0.0590	2.3421
Estonia	2034	2	46	0.0594	2.3828
Estonia	2035	2	47	0.0601	2.4161
Estonia	2036	2	48	0.0608	2.4494
Estonia	2037	2	48	0.0616	2.4826
Estonia	2038	2	49	0.0623	2.5158
Estonia	2039	2	50	0.0630	2.5489
Estonia	2040	2	51	0.0637	2.5820
Eswatini	2022	0	15	0.0639	1.6434
Eswatini	2023	0	15	0.0642	1.6536
Eswatini	2024	0	16	0.0646	1.6639
Eswatini	2025	0	16	0.0649	1.6725
Eswatini	2026	0	16	0.0651	1.6811
Eswatini	2027	0	17	0.0654	1.6898
Eswatini	2028	0	17	0.0657	1.6984
Eswatini	2029	0	17	0.0660	1.7071
Eswatini	2030	0	18	0.0661	1.7126
Eswatini	2031	0	18	0.0663	1.7180
Eswatini	2032	1	18	0.0664	1.7235
Eswatini	2033	1	19	0.0666	1.7290
Eswatini	2034	1	19	0.0668	1.7344
Eswatini	2035	1	20	0.0668	1.7365
Eswatini	2036	1	20	0.0668	1.7386
Eswatini	2037	1	20	0.0669	1.7407
Eswatini	2038	1	21	0.0669	1.7428
Eswatini	2039	1	21	0.0669	1.7449
Eswatini	2040	1	21	0.0670	1.7470
Ethiopia	2022	23	979	0.0445	1.1422

Ethiopia	2023	24	1018	0.0448	1.1530
Ethiopia	2024	25	1059	0.0452	1.1639
Ethiopia	2025	27	1099	0.0455	1.1725
Ethiopia	2026	28	1139	0.0458	1.1812
Ethiopia	2027	29	1180	0.0461	1.1898
Ethiopia	2028	30	1222	0.0464	1.1985
Ethiopia	2029	32	1266	0.0467	1.2072
Ethiopia	2030	33	1307	0.0469	1.2127
Ethiopia	2031	35	1349	0.0471	1.2182
Ethiopia	2032	36	1391	0.0472	1.2237
Ethiopia	2033	38	1435	0.0474	1.2291
Ethiopia	2034	39	1479	0.0476	1.2346
Ethiopia	2035	41	1520	0.0476	1.2366
Ethiopia	2036	42	1562	0.0477	1.2385
Ethiopia	2037	44	1605	0.0477	1.2404
Ethiopia	2038	46	1649	0.0478	1.2423
Ethiopia	2039	48	1694	0.0478	1.2443
Ethiopia	2040	50	1740	0.0479	1.2462
Fiji	2022	0	7	0.0337	0.8197
Fiji	2023	0	7	0.0338	0.8222
Fiji	2024	0	7	0.0339	0.8248
Fiji	2025	0	7	0.0339	0.8265
Fiji	2026	0	7	0.0340	0.8282
Fiji	2027	0	7	0.0340	0.8299
Fiji	2028	0	7	0.0341	0.8316
Fiji	2029	0	7	0.0342	0.8333
Fiji	2030	0	8	0.0342	0.8342
Fiji	2031	0	8	0.0342	0.8351

Fiji	2032	0	8	0.0343	0.8359
Fiji	2033	0	8	0.0343	0.8368
Fiji	2034	0	8	0.0343	0.8377
Fiji	2035	0	8	0.0343	0.8376
Fiji	2036	0	8	0.0343	0.8376
Fiji	2037	0	8	0.0343	0.8375
Fiji	2038	0	9	0.0343	0.8374
Fiji	2039	0	9	0.0343	0.8374
Fiji	2040	0	9	0.0343	0.8374
Finland	2022	41	628	0.2765	5.4005
Finland	2023	41	632	0.2732	5.3284
Finland	2024	42	638	0.2698	5.2561
Finland	2025	42	644	0.2675	5.2093
Finland	2026	43	651	0.2652	5.1624
Finland	2027	44	656	0.2629	5.1155
Finland	2028	45	662	0.2606	5.0685
Finland	2029	45	667	0.2583	5.0215
Finland	2030	46	675	0.2569	4.9937
Finland	2031	47	682	0.2554	4.9660
Finland	2032	48	688	0.2540	4.9382
Finland	2033	49	693	0.2525	4.9103
Finland	2034	50	698	0.2511	4.8824
Finland	2035	51	704	0.2503	4.8713
Finland	2036	51	709	0.2496	4.8602
Finland	2037	52	713	0.2488	4.8491
Finland	2038	53	716	0.2481	4.8380
Finland	2039	53	719	0.2474	4.8269
Finland	2040	54	720	0.2466	4.8157

France	2022	485	7717	0.2782	6.1803
France	2023	495	7970	0.2796	6.3245
France	2024	506	8235	0.2810	6.4691
France	2025	518	8490	0.2819	6.5838
France	2026	530	8750	0.2829	6.6987
France	2027	542	9011	0.2838	6.8137
France	2028	555	9282	0.2847	6.9287
France	2029	568	9567	0.2856	7.0439
France	2030	583	9821	0.2861	7.1252
France	2031	597	10079	0.2866	7.2066
France	2032	612	10337	0.2871	7.2879
France	2033	627	10602	0.2876	7.3692
France	2034	644	10878	0.2882	7.4507
France	2035	661	11106	0.2883	7.4909
France	2036	678	11330	0.2884	7.5310
France	2037	693	11544	0.2884	7.5710
France	2038	709	11751	0.2885	7.6111
France	2039	724	11957	0.2886	7.6512
France	2040	739	12155	0.2887	7.6913
Gabon	2022	1	23	0.0627	1.5816
Gabon	2023	1	23	0.0631	1.5945
Gabon	2024	1	24	0.0635	1.6074
Gabon	2025	1	25	0.0639	1.6175
Gabon	2026	1	26	0.0642	1.6275
Gabon	2027	1	26	0.0645	1.6375
Gabon	2028	1	27	0.0648	1.6475
Gabon	2029	1	28	0.0651	1.6575
Gabon	2030	1	29	0.0653	1.6637

Gabon	2031	1	29	0.0655	1.6700
Gabon	2032	1	30	0.0657	1.6762
Gabon	2033	1	31	0.0658	1.6825
Gabon	2034	1	32	0.0660	1.6888
Gabon	2035	1	32	0.0660	1.6913
Gabon	2036	1	33	0.0661	1.6938
Gabon	2037	1	34	0.0661	1.6963
Gabon	2038	1	34	0.0662	1.6989
Gabon	2039	1	35	0.0662	1.7015
Gabon	2040	1	36	0.0662	1.7042
Gambia	2022	0	18	0.0468	1.1390
Gambia	2023	1	19	0.0471	1.1483
Gambia	2024	1	20	0.0475	1.1576
Gambia	2025	1	20	0.0477	1.1645
Gambia	2026	1	21	0.0480	1.1715
Gambia	2027	1	21	0.0482	1.1784
Gambia	2028	1	22	0.0484	1.1853
Gambia	2029	1	23	0.0487	1.1922
Gambia	2030	1	23	0.0488	1.1965
Gambia	2031	1	24	0.0489	1.2008
Gambia	2032	1	24	0.0491	1.2051
Gambia	2033	1	25	0.0492	1.2094
Gambia	2034	1	26	0.0494	1.2138
Gambia	2035	1	26	0.0494	1.2152
Gambia	2036	1	27	0.0494	1.2168
Gambia	2037	1	28	0.0495	1.2183
Gambia	2038	1	28	0.0495	1.2198
Gambia	2039	1	29	0.0495	1.2213

Gambia	2040	1	30	0.0495	1.2229
Georgia	2022	3	85	0.0620	2.0302
Georgia	2023	3	85	0.0621	2.0431
Georgia	2024	3	85	0.0623	2.0561
Georgia	2025	3	86	0.0627	2.0686
Georgia	2026	3	86	0.0631	2.0811
Georgia	2027	3	87	0.0634	2.0934
Georgia	2028	3	87	0.0638	2.1057
Georgia	2029	3	88	0.0641	2.1180
Georgia	2030	3	89	0.0647	2.1341
Georgia	2031	3	90	0.0653	2.1503
Georgia	2032	4	91	0.0658	2.1664
Georgia	2033	4	92	0.0664	2.1824
Georgia	2034	4	93	0.0670	2.1985
Georgia	2035	4	94	0.0678	2.2194
Georgia	2036	4	95	0.0686	2.2404
Georgia	2037	4	97	0.0694	2.2613
Georgia	2038	4	98	0.0702	2.2822
Georgia	2039	4	99	0.0711	2.3031
Georgia	2040	4	100	0.0719	2.3241
Germany	2022	1069	16710	0.4772	9.7864
Germany	2023	1097	17055	0.4822	9.8728
Germany	2024	1128	17424	0.4872	9.9592
Germany	2025	1159	17787	0.4912	10.0181
Germany	2026	1188	18143	0.4953	10.0769
Germany	2027	1214	18484	0.4993	10.1356
Germany	2028	1240	18830	0.5032	10.1942
Germany	2029	1268	19196	0.5072	10.2528

Germany	2030	1292	19524	0.5097	10.2855
Germany	2031	1317	19850	0.5122	10.3182
Germany	2032	1341	20164	0.5146	10.3508
Germany	2033	1366	20479	0.5171	10.3833
Germany	2034	1393	20807	0.5195	10.4158
Germany	2035	1417	21082	0.5204	10.4197
Germany	2036	1442	21355	0.5213	10.4236
Germany	2037	1467	21618	0.5222	10.4274
Germany	2038	1494	21880	0.5232	10.4312
Germany	2039	1523	22148	0.5241	10.4350
Germany	2040	1553	22420	0.5250	10.4388
Ghana	2022	10	360	0.0582	1.4686
Ghana	2023	10	372	0.0586	1.4811
Ghana	2024	10	384	0.0590	1.4936
Ghana	2025	11	396	0.0593	1.5036
Ghana	2026	11	408	0.0596	1.5136
Ghana	2027	12	420	0.0599	1.5236
Ghana	2028	12	433	0.0602	1.5337
Ghana	2029	12	445	0.0606	1.5438
Ghana	2030	13	457	0.0607	1.5501
Ghana	2031	13	470	0.0609	1.5564
Ghana	2032	14	482	0.0611	1.5627
Ghana	2033	14	495	0.0613	1.5691
Ghana	2034	15	508	0.0615	1.5755
Ghana	2035	15	520	0.0615	1.5781
Ghana	2036	16	532	0.0616	1.5806
Ghana	2037	16	545	0.0616	1.5831
Ghana	2038	17	558	0.0617	1.5857

Ghana	2039	18	572	0.0617	1.5883
Ghana	2040	18	586	0.0618	1.5908
Greece	2022	82	1011	0.2391	3.7594
Greece	2023	85	1052	0.2460	3.8871
Greece	2024	89	1096	0.2528	4.0149
Greece	2025	91	1131	0.2572	4.1078
Greece	2026	94	1164	0.2616	4.2004
Greece	2027	96	1195	0.2659	4.2928
Greece	2028	98	1227	0.2703	4.3851
Greece	2029	100	1260	0.2746	4.4774
Greece	2030	102	1285	0.2768	4.5302
Greece	2031	104	1311	0.2790	4.5830
Greece	2032	106	1338	0.2812	4.6358
Greece	2033	108	1366	0.2833	4.6885
Greece	2034	110	1396	0.2855	4.7413
Greece	2035	111	1417	0.2854	4.7482
Greece	2036	113	1439	0.2853	4.7552
Greece	2037	115	1462	0.2852	4.7624
Greece	2038	117	1486	0.2851	4.7697
Greece	2039	119	1512	0.2851	4.7771
Greece	2040	121	1540	0.2850	4.7846
Greenland	2022	1	13	0.8713	18.4801
Greenland	2023	1	13	0.8743	18.5578
Greenland	2024	1	13	0.8772	18.6360
Greenland	2025	1	14	0.8787	18.6794
Greenland	2026	1	14	0.8803	18.7230
Greenland	2027	1	15	0.8818	18.7665
Greenland	2028	1	15	0.8834	18.8103

Greenland	2029	1	16	0.8850	18.8544
Greenland	2030	1	16	0.8852	18.8716
Greenland	2031	1	17	0.8855	18.8886
Greenland	2032	1	17	0.8858	18.9051
Greenland	2033	1	18	0.8861	18.9211
Greenland	2034	1	18	0.8864	18.9367
Greenland	2035	1	18	0.8855	18.9256
Greenland	2036	1	19	0.8845	18.9142
Greenland	2037	1	19	0.8836	18.9026
Greenland	2038	1	19	0.8827	18.8909
Greenland	2039	1	20	0.8817	18.8791
Greenland	2040	1	20	0.8807	18.8671
Grenada	2022	0	1	0.0128	0.5038
Grenada	2023	0	1	0.0128	0.5084
Grenada	2024	0	1	0.0129	0.5130
Grenada	2025	0	1	0.0130	0.5167
Grenada	2026	0	1	0.0131	0.5204
Grenada	2027	0	1	0.0132	0.5241
Grenada	2028	0	1	0.0132	0.5278
Grenada	2029	0	1	0.0133	0.5314
Grenada	2030	0	1	0.0134	0.5338
Grenada	2031	0	1	0.0134	0.5362
Grenada	2032	0	1	0.0135	0.5386
Grenada	2033	0	1	0.0135	0.5410
Grenada	2034	0	1	0.0135	0.5434
Grenada	2035	0	1	0.0136	0.5446
Grenada	2036	0	1	0.0136	0.5457
Grenada	2037	0	1	0.0136	0.5469

Grenada	2038	0	1	0.0136	0.5481
Grenada	2039	0	1	0.0136	0.5493
Grenada	2040	0	1	0.0136	0.5504
Guam	2022	0	2	0.0392	0.9367
Guam	2023	0	2	0.0394	0.9401
Guam	2024	0	2	0.0395	0.9434
Guam	2025	0	2	0.0396	0.9459
Guam	2026	0	2	0.0397	0.9484
Guam	2027	0	2	0.0398	0.9508
Guam	2028	0	2	0.0399	0.9533
Guam	2029	0	2	0.0399	0.9557
Guam	2030	0	2	0.0400	0.9572
Guam	2031	0	2	0.0400	0.9586
Guam	2032	0	2	0.0400	0.9600
Guam	2033	0	2	0.0401	0.9614
Guam	2034	0	2	0.0401	0.9628
Guam	2035	0	2	0.0401	0.9633
Guam	2036	0	2	0.0401	0.9637
Guam	2037	0	2	0.0400	0.9642
Guam	2038	0	2	0.0400	0.9647
Guam	2039	0	2	0.0400	0.9652
Guam	2040	0	3	0.0400	0.9657
Guatemala	2022	5	221	0.0330	1.2849
Guatemala	2023	5	225	0.0331	1.2940
Guatemala	2024	5	228	0.0333	1.3031
Guatemala	2025	5	232	0.0334	1.3100
Guatemala	2026	5	236	0.0336	1.3169
Guatemala	2027	5	239	0.0337	1.3238

Guatemala	2028	6	242	0.0338	1.3307
Guatemala	2029	6	246	0.0340	1.3377
Guatemala	2030	6	249	0.0340	1.3419
Guatemala	2031	6	252	0.0341	1.3460
Guatemala	2032	6	255	0.0342	1.3502
Guatemala	2033	6	259	0.0342	1.3544
Guatemala	2034	6	262	0.0343	1.3586
Guatemala	2035	7	265	0.0343	1.3603
Guatemala	2036	7	267	0.0343	1.3621
Guatemala	2037	7	270	0.0343	1.3639
Guatemala	2038	7	273	0.0343	1.3656
Guatemala	2039	7	276	0.0343	1.3675
Guatemala	2040	7	279	0.0342	1.3693
Guinea	2022	3	104	0.0437	1.0847
Guinea	2023	3	107	0.0439	1.0917
Guinea	2024	3	111	0.0441	1.0987
Guinea	2025	3	114	0.0443	1.1046
Guinea	2026	3	117	0.0445	1.1105
Guinea	2027	3	120	0.0447	1.1164
Guinea	2028	3	123	0.0449	1.1224
Guinea	2029	3	127	0.0451	1.1283
Guinea	2030	3	130	0.0452	1.1319
Guinea	2031	3	133	0.0453	1.1354
Guinea	2032	3	137	0.0454	1.1390
Guinea	2033	3	140	0.0455	1.1427
Guinea	2034	4	144	0.0456	1.1463
Guinea	2035	4	147	0.0456	1.1475
Guinea	2036	4	151	0.0456	1.1487

Guinea	2037	4	154	0.0456	1.1499
Guinea	2038	4	158	0.0456	1.1511
Guinea	2039	4	161	0.0456	1.1524
Guinea	2040	4	165	0.0457	1.1536
Guinea-Bissau	2022	0	16	0.0456	1.1513
Guinea-Bissau	2023	0	17	0.0457	1.1566
Guinea-Bissau	2024	0	17	0.0459	1.1618
Guinea-Bissau	2025	0	18	0.0460	1.1646
Guinea-Bissau	2026	0	18	0.0461	1.1675
Guinea-Bissau	2027	0	19	0.0463	1.1704
Guinea-Bissau	2028	0	19	0.0464	1.1732
Guinea-Bissau	2029	0	20	0.0465	1.1761
Guinea-Bissau	2030	0	20	0.0466	1.1776
Guinea-Bissau	2031	0	21	0.0466	1.1790
Guinea-Bissau	2032	1	21	0.0467	1.1805
Guinea-Bissau	2033	1	22	0.0467	1.1819
Guinea-Bissau	2034	1	22	0.0468	1.1834
Guinea-Bissau	2035	1	23	0.0468	1.1834
Guinea-Bissau	2036	1	23	0.0468	1.1834
Guinea-Bissau	2037	1	24	0.0468	1.1834
Guinea-Bissau	2038	1	24	0.0468	1.1835
Guinea-Bissau	2039	1	25	0.0468	1.1835
Guinea-Bissau	2040	1	25	0.0468	1.1836
Guyana	2022	0	4	0.0133	0.5650
Guyana	2023	0	4	0.0134	0.5699
Guyana	2024	0	4	0.0135	0.5748
Guyana	2025	0	4	0.0135	0.5782
Guyana	2026	0	4	0.0136	0.5816

Guyana	2027	0	4	0.0137	0.5850
Guyana	2028	0	4	0.0137	0.5884
Guyana	2029	0	4	0.0138	0.5918
Guyana	2030	0	5	0.0138	0.5938
Guyana	2031	0	5	0.0139	0.5958
Guyana	2032	0	5	0.0139	0.5979
Guyana	2033	0	5	0.0139	0.5999
Guyana	2034	0	5	0.0140	0.6020
Guyana	2035	0	5	0.0139	0.6028
Guyana	2036	0	5	0.0139	0.6035
Guyana	2037	0	5	0.0139	0.6043
Guyana	2038	0	5	0.0139	0.6050
Guyana	2039	0	5	0.0139	0.6058
Guyana	2040	0	5	0.0139	0.6065
Haiti	2022	1	75	0.0113	0.5627
Haiti	2023	1	76	0.0113	0.5661
Haiti	2024	1	77	0.0114	0.5696
Haiti	2025	1	78	0.0114	0.5722
Haiti	2026	1	79	0.0115	0.5748
Haiti	2027	1	79	0.0115	0.5773
Haiti	2028	1	80	0.0116	0.5799
Haiti	2029	1	81	0.0116	0.5825
Haiti	2030	1	81	0.0116	0.5841
Haiti	2031	1	81	0.0116	0.5857
Haiti	2032	1	82	0.0117	0.5874
Haiti	2033	1	82	0.0117	0.5890
Haiti	2034	1	82	0.0117	0.5906
Haiti	2035	1	82	0.0117	0.5914

Haiti	2036	1	82	0.0117	0.5921
Haiti	2037	1	83	0.0117	0.5929
Haiti	2038	1	83	0.0117	0.5936
Haiti	2039	2	83	0.0117	0.5944
Haiti	2040	2	83	0.0117	0.5952
Honduras	2022	3	117	0.0324	1.1988
Honduras	2023	3	120	0.0327	1.2108
Honduras	2024	3	123	0.0330	1.2228
Honduras	2025	3	126	0.0332	1.2310
Honduras	2026	3	128	0.0334	1.2391
Honduras	2027	3	131	0.0336	1.2473
Honduras	2028	3	133	0.0338	1.2555
Honduras	2029	3	136	0.0340	1.2636
Honduras	2030	3	138	0.0341	1.2688
Honduras	2031	3	141	0.0342	1.2739
Honduras	2032	3	143	0.0343	1.2791
Honduras	2033	4	145	0.0344	1.2842
Honduras	2034	4	147	0.0345	1.2894
Honduras	2035	4	149	0.0345	1.2917
Honduras	2036	4	151	0.0346	1.2940
Honduras	2037	4	153	0.0346	1.2962
Honduras	2038	4	155	0.0346	1.2985
Honduras	2039	4	157	0.0346	1.3008
Honduras	2040	4	159	0.0346	1.3031
Hungary	2022	82	1332	0.3853	7.7140
Hungary	2023	84	1328	0.3896	7.5342
Hungary	2024	87	1327	0.3941	7.3552
Hungary	2025	89	1322	0.3961	7.2292

Hungary	2026	91	1317	0.3981	7.1031
Hungary	2027	93	1310	0.4002	6.9766
Hungary	2028	96	1303	0.4022	6.8501
Hungary	2029	98	1296	0.4043	6.7242
Hungary	2030	100	1293	0.4042	6.6251
Hungary	2031	102	1288	0.4042	6.5257
Hungary	2032	104	1283	0.4041	6.4258
Hungary	2033	106	1278	0.4040	6.3259
Hungary	2034	108	1274	0.4040	6.2260
Hungary	2035	109	1273	0.4022	6.1531
Hungary	2036	111	1271	0.4004	6.0798
Hungary	2037	112	1268	0.3985	6.0061
Hungary	2038	113	1265	0.3967	5.9320
Hungary	2039	115	1262	0.3949	5.8578
Hungary	2040	116	1259	0.3930	5.7834
Iceland	2022	1	23	0.2215	4.1543
Iceland	2023	2	24	0.2241	4.2220
Iceland	2024	2	25	0.2268	4.2898
Iceland	2025	2	26	0.2288	4.3409
Iceland	2026	2	27	0.2309	4.3919
Iceland	2027	2	28	0.2329	4.4429
Iceland	2028	2	29	0.2350	4.4939
Iceland	2029	2	30	0.2370	4.5450
Iceland	2030	2	31	0.2383	4.5807
Iceland	2031	2	32	0.2397	4.6165
Iceland	2032	2	33	0.2410	4.6522
Iceland	2033	2	34	0.2423	4.6880
Iceland	2034	2	35	0.2436	4.7239

Iceland	2035	2	36	0.2442	4.7435
Iceland	2036	3	37	0.2448	4.7630
Iceland	2037	3	38	0.2454	4.7825
Iceland	2038	3	39	0.2460	4.8020
Iceland	2039	3	40	0.2466	4.8217
Iceland	2040	3	41	0.2473	4.8415
India	2022	250	9170	0.0221	0.6897
India	2023	259	9389	0.0224	0.6973
India	2024	268	9614	0.0226	0.7049
India	2025	276	9807	0.0228	0.7104
India	2026	284	10001	0.0229	0.7158
India	2027	293	10196	0.0231	0.7213
India	2028	302	10395	0.0233	0.7268
India	2029	311	10598	0.0234	0.7322
India	2030	319	10764	0.0235	0.7351
India	2031	328	10933	0.0236	0.7380
India	2032	336	11105	0.0237	0.7408
India	2033	345	11280	0.0238	0.7437
India	2034	354	11459	0.0238	0.7466
India	2035	363	11596	0.0238	0.7467
India	2036	371	11733	0.0238	0.7467
India	2037	379	11871	0.0238	0.7468
India	2038	388	12009	0.0238	0.7468
India	2039	397	12152	0.0238	0.7469
India	2040	406	12297	0.0238	0.7469
Indonesia	2022	49	1591	0.0249	0.6776
Indonesia	2023	51	1621	0.0249	0.6788
Indonesia	2024	53	1652	0.0250	0.6800

Indonesia	2025	54	1685	0.0250	0.6808
Indonesia	2026	56	1718	0.0250	0.6817
Indonesia	2027	58	1751	0.0251	0.6825
Indonesia	2028	60	1787	0.0251	0.6834
Indonesia	2029	62	1823	0.0251	0.6843
Indonesia	2030	64	1860	0.0251	0.6847
Indonesia	2031	66	1897	0.0251	0.6852
Indonesia	2032	68	1935	0.0251	0.6856
Indonesia	2033	70	1973	0.0252	0.6860
Indonesia	2034	73	2012	0.0252	0.6865
Indonesia	2035	75	2050	0.0252	0.6864
Indonesia	2036	78	2087	0.0251	0.6864
Indonesia	2037	80	2124	0.0251	0.6864
Indonesia	2038	83	2162	0.0251	0.6863
Indonesia	2039	86	2199	0.0251	0.6863
Indonesia	2040	88	2237	0.0251	0.6862
Iran (Islamic Republic of)	2022	21	634	0.0285	0.7946
Iran (Islamic Republic of)	2023	21	650	0.0287	0.8019
Iran (Islamic Republic of)	2024	22	667	0.0290	0.8093
Iran (Islamic Republic of)	2025	23	682	0.0292	0.8145
Iran (Islamic Republic of)	2026	24	697	0.0293	0.8198
Iran (Islamic Republic of)	2027	25	713	0.0295	0.8251
Iran (Islamic Republic of)	2028	25	729	0.0297	0.8304
Iran (Islamic Republic of)	2029	26	745	0.0298	0.8357
Iran (Islamic Republic of)	2030	27	760	0.0299	0.8386
Iran (Islamic Republic of)	2031	28	776	0.0300	0.8416
Iran (Islamic Republic of)	2032	29	792	0.0301	0.8446
Iran (Islamic Republic of)	2033	31	808	0.0301	0.8476

Iran (Islamic Republic of)	2034	32	826	0.0302	0.8506
Iran (Islamic Republic of)	2035	33	842	0.0302	0.8511
Iran (Islamic Republic of)	2036	34	858	0.0302	0.8515
Iran (Islamic Republic of)	2037	36	875	0.0302	0.8519
Iran (Islamic Republic of)	2038	37	893	0.0301	0.8524
Iran (Islamic Republic of)	2039	38	912	0.0301	0.8529
Iran (Islamic Republic of)	2040	40	932	0.0301	0.8534
Iraq	2022	8	287	0.0259	0.6973
Iraq	2023	8	299	0.0262	0.7056
Iraq	2024	9	312	0.0265	0.7140
Iraq	2025	9	325	0.0267	0.7199
Iraq	2026	10	337	0.0269	0.7258
Iraq	2027	11	350	0.0271	0.7318
Iraq	2028	11	364	0.0273	0.7378
Iraq	2029	12	378	0.0275	0.7437
Iraq	2030	13	392	0.0276	0.7471
Iraq	2031	13	406	0.0277	0.7504
Iraq	2032	14	420	0.0279	0.7538
Iraq	2033	15	435	0.0280	0.7572
Iraq	2034	16	450	0.0281	0.7606
Iraq	2035	17	465	0.0281	0.7610
Iraq	2036	18	480	0.0280	0.7614
Iraq	2037	18	495	0.0280	0.7618
Iraq	2038	19	511	0.0280	0.7623
Iraq	2039	20	527	0.0280	0.7627
Iraq	2040	22	543	0.0280	0.7632
Ireland	2022	23	363	0.2711	4.6779
Ireland	2023	24	373	0.2739	4.6748

Ireland	2024	26	384	0.2767	4.6716
Ireland	2025	27	396	0.2792	4.6754
Ireland	2026	28	409	0.2817	4.6791
Ireland	2027	29	421	0.2841	4.6826
Ireland	2028	31	434	0.2866	4.6861
Ireland	2029	32	448	0.2891	4.6896
Ireland	2030	34	463	0.2907	4.6939
Ireland	2031	35	477	0.2924	4.6982
Ireland	2032	36	491	0.2941	4.7023
Ireland	2033	38	506	0.2957	4.7063
Ireland	2034	40	521	0.2974	4.7104
Ireland	2035	41	537	0.2983	4.7153
Ireland	2036	43	552	0.2992	4.7202
Ireland	2037	44	568	0.3001	4.7250
Ireland	2038	46	583	0.3009	4.7298
Ireland	2039	47	599	0.3018	4.7346
Ireland	2040	49	615	0.3027	4.7394
Israel	2022	38	695	0.2757	5.7555
Israel	2023	39	717	0.2773	5.7938
Israel	2024	41	741	0.2789	5.8321
Israel	2025	42	763	0.2798	5.8582
Israel	2026	44	786	0.2807	5.8843
Israel	2027	45	809	0.2816	5.9104
Israel	2028	47	834	0.2825	5.9365
Israel	2029	49	859	0.2834	5.9626
Israel	2030	51	884	0.2837	5.9770
Israel	2031	53	909	0.2841	5.9914
Israel	2032	54	935	0.2845	6.0057

Israel	2033	56	961	0.2848	6.0199
Israel	2034	58	988	0.2852	6.0342
Israel	2035	60	1014	0.2850	6.0367
Israel	2036	63	1041	0.2849	6.0393
Israel	2037	64	1066	0.2847	6.0418
Israel	2038	66	1092	0.2846	6.0443
Israel	2039	68	1118	0.2844	6.0469
Israel	2040	70	1144	0.2843	6.0494
Italy	2022	691	9612	0.3584	6.7759
Italy	2023	725	10063	0.3688	6.9874
Italy	2024	762	10535	0.3793	7.1991
Italy	2025	794	10968	0.3871	7.3738
Italy	2026	826	11403	0.3950	7.5485
Italy	2027	857	11838	0.4028	7.7230
Italy	2028	889	12284	0.4107	7.8975
Italy	2029	923	12749	0.4185	8.0722
Italy	2030	950	13136	0.4225	8.1831
Italy	2031	978	13530	0.4265	8.2940
Italy	2032	1005	13927	0.4305	8.4049
Italy	2033	1033	14336	0.4345	8.5159
Italy	2034	1063	14764	0.4385	8.6269
Italy	2035	1083	15065	0.4381	8.6603
Italy	2036	1104	15370	0.4376	8.6937
Italy	2037	1124	15673	0.4372	8.7271
Italy	2038	1145	15980	0.4367	8.7605
Italy	2039	1168	16298	0.4362	8.7940
Italy	2040	1191	16621	0.4358	8.8275
Jamaica	2022	0	11	0.0114	0.4481

Jamaica	2023	0	11	0.0115	0.4517
Jamaica	2024	0	12	0.0116	0.4553
Jamaica	2025	0	12	0.0116	0.4577
Jamaica	2026	0	12	0.0117	0.4602
Jamaica	2027	0	12	0.0117	0.4626
Jamaica	2028	0	12	0.0118	0.4650
Jamaica	2029	0	12	0.0119	0.4674
Jamaica	2030	0	12	0.0119	0.4688
Jamaica	2031	0	12	0.0119	0.4703
Jamaica	2032	0	12	0.0119	0.4717
Jamaica	2033	0	12	0.0119	0.4732
Jamaica	2034	0	12	0.0120	0.4746
Jamaica	2035	0	12	0.0119	0.4750
Jamaica	2036	0	12	0.0119	0.4755
Jamaica	2037	0	12	0.0119	0.4759
Jamaica	2038	0	12	0.0119	0.4763
Jamaica	2039	0	12	0.0119	0.4768
Jamaica	2040	0	12	0.0119	0.4772
Japan	2022	986	13299	0.2063	4.6222
Japan	2023	1011	13396	0.2067	4.5771
Japan	2024	1038	13505	0.2070	4.5322
Japan	2025	1064	13634	0.2070	4.4941
Japan	2026	1088	13751	0.2070	4.4557
Japan	2027	1109	13850	0.2069	4.4171
Japan	2028	1131	13954	0.2068	4.3784
Japan	2029	1155	14076	0.2068	4.3398
Japan	2030	1177	14218	0.2065	4.3123
Japan	2031	1198	14348	0.2062	4.2846

Japan	2032	1216	14454	0.2058	4.2568
Japan	2033	1234	14555	0.2055	4.2291
Japan	2034	1254	14668	0.2052	4.2014
Japan	2035	1271	14790	0.2046	4.1833
Japan	2036	1286	14890	0.2040	4.1652
Japan	2037	1296	14954	0.2034	4.1469
Japan	2038	1304	14995	0.2028	4.1286
Japan	2039	1312	15033	0.2022	4.1104
Japan	2040	1317	15057	0.2016	4.0921
Jordan	2022	2	65	0.0269	0.6897
Jordan	2023	2	67	0.0272	0.6960
Jordan	2024	2	70	0.0274	0.7023
Jordan	2025	2	73	0.0276	0.7063
Jordan	2026	2	75	0.0277	0.7104
Jordan	2027	2	78	0.0279	0.7144
Jordan	2028	3	80	0.0280	0.7185
Jordan	2029	3	83	0.0282	0.7226
Jordan	2030	3	86	0.0283	0.7248
Jordan	2031	3	89	0.0283	0.7271
Jordan	2032	3	92	0.0284	0.7293
Jordan	2033	3	95	0.0285	0.7316
Jordan	2034	3	98	0.0286	0.7339
Jordan	2035	4	101	0.0285	0.7341
Jordan	2036	4	104	0.0285	0.7343
Jordan	2037	4	107	0.0285	0.7345
Jordan	2038	4	110	0.0285	0.7348
Jordan	2039	4	114	0.0285	0.7350
Jordan	2040	4	117	0.0285	0.7353

Kazakhstan	2022	9	276	0.0591	1.5373
Kazakhstan	2023	10	284	0.0595	1.5556
Kazakhstan	2024	10	292	0.0600	1.5739
Kazakhstan	2025	10	300	0.0602	1.5869
Kazakhstan	2026	11	308	0.0605	1.6000
Kazakhstan	2027	11	315	0.0607	1.6130
Kazakhstan	2028	11	323	0.0610	1.6260
Kazakhstan	2029	12	332	0.0613	1.6391
Kazakhstan	2030	12	339	0.0614	1.6477
Kazakhstan	2031	12	347	0.0616	1.6562
Kazakhstan	2032	13	355	0.0618	1.6648
Kazakhstan	2033	13	363	0.0620	1.6733
Kazakhstan	2034	14	372	0.0621	1.6818
Kazakhstan	2035	14	380	0.0622	1.6857
Kazakhstan	2036	15	388	0.0623	1.6896
Kazakhstan	2037	15	396	0.0623	1.6934
Kazakhstan	2038	16	404	0.0624	1.6973
Kazakhstan	2039	16	413	0.0625	1.7012
Kazakhstan	2040	17	423	0.0625	1.7051
Kenya	2022	14	572	0.0558	1.4697
Kenya	2023	14	589	0.0561	1.4790
Kenya	2024	15	607	0.0564	1.4883
Kenya	2025	16	624	0.0566	1.4954
Kenya	2026	16	642	0.0569	1.5025
Kenya	2027	17	660	0.0571	1.5095
Kenya	2028	17	678	0.0574	1.5165
Kenya	2029	18	697	0.0576	1.5236
Kenya	2030	19	715	0.0577	1.5276

Kenya	2031	19	734	0.0578	1.5317
Kenya	2032	20	752	0.0580	1.5358
Kenya	2033	21	771	0.0581	1.5399
Kenya	2034	22	790	0.0582	1.5441
Kenya	2035	22	809	0.0582	1.5452
Kenya	2036	23	827	0.0582	1.5463
Kenya	2037	24	846	0.0583	1.5475
Kenya	2038	25	864	0.0583	1.5487
Kenya	2039	25	884	0.0583	1.5499
Kenya	2040	26	903	0.0583	1.5511
Kiribati	2022	0	1	0.0286	0.7311
Kiribati	2023	0	1	0.0287	0.7327
Kiribati	2024	0	1	0.0288	0.7344
Kiribati	2025	0	1	0.0288	0.7356
Kiribati	2026	0	1	0.0288	0.7368
Kiribati	2027	0	1	0.0289	0.7380
Kiribati	2028	0	1	0.0289	0.7391
Kiribati	2029	0	1	0.0290	0.7403
Kiribati	2030	0	1	0.0290	0.7409
Kiribati	2031	0	1	0.0290	0.7414
Kiribati	2032	0	1	0.0290	0.7420
Kiribati	2033	0	1	0.0290	0.7426
Kiribati	2034	0	1	0.0291	0.7432
Kiribati	2035	0	1	0.0290	0.7431
Kiribati	2036	0	1	0.0290	0.7431
Kiribati	2037	0	1	0.0290	0.7430
Kiribati	2038	0	1	0.0290	0.7430
Kiribati	2039	0	1	0.0290	0.7430

Kiribati	2040	0	1	0.0290	0.7430
Kuwait	2022	1	29	0.0295	0.7339
Kuwait	2023	1	29	0.0295	0.7320
Kuwait	2024	1	30	0.0295	0.7301
Kuwait	2025	1	31	0.0294	0.7281
Kuwait	2026	1	32	0.0294	0.7261
Kuwait	2027	1	33	0.0294	0.7240
Kuwait	2028	1	34	0.0294	0.7220
Kuwait	2029	1	35	0.0293	0.7200
Kuwait	2030	1	36	0.0293	0.7183
Kuwait	2031	1	37	0.0293	0.7165
Kuwait	2032	2	38	0.0293	0.7148
Kuwait	2033	2	39	0.0292	0.7132
Kuwait	2034	2	40	0.0292	0.7115
Kuwait	2035	2	42	0.0292	0.7099
Kuwait	2036	2	43	0.0291	0.7083
Kuwait	2037	2	45	0.0291	0.7067
Kuwait	2038	2	46	0.0291	0.7051
Kuwait	2039	2	48	0.0290	0.7035
Kuwait	2040	3	50	0.0290	0.7019
Kyrgyzstan	2022	2	73	0.0522	1.2705
Kyrgyzstan	2023	2	75	0.0526	1.2796
Kyrgyzstan	2024	3	77	0.0530	1.2888
Kyrgyzstan	2025	3	79	0.0532	1.2943
Kyrgyzstan	2026	3	81	0.0534	1.2998
Kyrgyzstan	2027	3	83	0.0536	1.3053
Kyrgyzstan	2028	3	86	0.0538	1.3107
Kyrgyzstan	2029	3	88	0.0541	1.3161

Kyrgyzstan	2030	3	90	0.0542	1.3190
Kyrgyzstan	2031	3	92	0.0543	1.3219
Kyrgyzstan	2032	3	94	0.0544	1.3248
Kyrgyzstan	2033	4	97	0.0545	1.3277
Kyrgyzstan	2034	4	99	0.0546	1.3305
Kyrgyzstan	2035	4	102	0.0546	1.3306
Kyrgyzstan	2036	4	104	0.0546	1.3306
Kyrgyzstan	2037	4	107	0.0546	1.3307
Kyrgyzstan	2038	4	109	0.0546	1.3308
Kyrgyzstan	2039	4	112	0.0546	1.3309
Kyrgyzstan	2040	5	115	0.0546	1.3310
Lao People's Democratic Republic	2022	1	33	0.0201	0.5433
Lao People's Democratic Republic	2023	1	34	0.0202	0.5451
Lao People's Democratic Republic	2024	1	34	0.0202	0.5470
Lao People's Democratic Republic	2025	1	35	0.0203	0.5480
Lao People's Democratic Republic	2026	1	36	0.0203	0.5491
Lao People's Democratic Republic	2027	1	36	0.0203	0.5501
Lao People's Democratic Republic	2028	1	37	0.0204	0.5512
Lao People's Democratic Republic	2029	1	38	0.0204	0.5522
Lao People's Democratic Republic	2030	1	38	0.0204	0.5527
Lao People's Democratic Republic	2031	1	39	0.0204	0.5532
Lao People's Democratic Republic	2032	1	40	0.0204	0.5537
Lao People's Democratic Republic	2033	1	41	0.0204	0.5542
Lao People's Democratic Republic	2034	1	41	0.0204	0.5548
Lao People's Democratic Republic	2035	1	42	0.0204	0.5547
Lao People's Democratic Republic	2036	1	43	0.0204	0.5546
Lao People's Democratic Republic	2037	1	43	0.0204	0.5545
Lao People's Democratic Republic	2038	2	44	0.0204	0.5545

Lao People's Democratic Republic	2039	2	45	0.0204	0.5544
Lao People's Democratic Republic	2040	2	46	0.0204	0.5543
Latvia	2022	2	50	0.0568	1.8434
Latvia	2023	2	50	0.0568	1.8858
Latvia	2024	2	51	0.0567	1.9279
Latvia	2025	2	52	0.0568	1.9578
Latvia	2026	2	52	0.0569	1.9876
Latvia	2027	2	53	0.0569	2.0172
Latvia	2028	2	54	0.0570	2.0466
Latvia	2029	2	55	0.0571	2.0758
Latvia	2030	2	56	0.0575	2.1055
Latvia	2031	2	56	0.0580	2.1350
Latvia	2032	2	57	0.0585	2.1645
Latvia	2033	2	58	0.0589	2.1939
Latvia	2034	2	58	0.0594	2.2231
Latvia	2035	2	59	0.0602	2.2500
Latvia	2036	2	60	0.0610	2.2769
Latvia	2037	2	60	0.0618	2.3038
Latvia	2038	2	61	0.0627	2.3306
Latvia	2039	2	61	0.0635	2.3573
Latvia	2040	2	62	0.0643	2.3840
Lebanon	2022	2	53	0.0260	0.6682
Lebanon	2023	2	55	0.0263	0.6754
Lebanon	2024	2	56	0.0267	0.6828
Lebanon	2025	2	58	0.0269	0.6879
Lebanon	2026	2	59	0.0271	0.6931
Lebanon	2027	2	61	0.0273	0.6982
Lebanon	2028	2	63	0.0275	0.7032

Lebanon	2029	2	64	0.0277	0.7082
Lebanon	2030	2	66	0.0278	0.7111
Lebanon	2031	2	68	0.0279	0.7139
Lebanon	2032	3	69	0.0280	0.7168
Lebanon	2033	3	71	0.0281	0.7196
Lebanon	2034	3	73	0.0282	0.7225
Lebanon	2035	3	74	0.0282	0.7230
Lebanon	2036	3	76	0.0282	0.7236
Lebanon	2037	3	77	0.0282	0.7242
Lebanon	2038	3	79	0.0282	0.7247
Lebanon	2039	3	81	0.0282	0.7252
Lebanon	2040	3	83	0.0282	0.7257
Lesotho	2022	1	24	0.0587	1.5024
Lesotho	2023	1	24	0.0590	1.5130
Lesotho	2024	1	25	0.0593	1.5236
Lesotho	2025	1	25	0.0596	1.5318
Lesotho	2026	1	26	0.0599	1.5401
Lesotho	2027	1	26	0.0601	1.5484
Lesotho	2028	1	26	0.0604	1.5567
Lesotho	2029	1	27	0.0606	1.5650
Lesotho	2030	1	27	0.0608	1.5701
Lesotho	2031	1	28	0.0609	1.5752
Lesotho	2032	1	28	0.0611	1.5804
Lesotho	2033	1	28	0.0612	1.5856
Lesotho	2034	1	29	0.0614	1.5908
Lesotho	2035	1	29	0.0614	1.5926
Lesotho	2036	1	29	0.0615	1.5945
Lesotho	2037	1	30	0.0615	1.5964

Lesotho	2038	1	30	0.0616	1.5983
Lesotho	2039	1	30	0.0616	1.6002
Lesotho	2040	1	31	0.0616	1.6021
Liberia	2022	1	39	0.0436	1.0723
Liberia	2023	1	40	0.0438	1.0779
Liberia	2024	1	41	0.0440	1.0834
Liberia	2025	1	43	0.0442	1.0879
Liberia	2026	1	44	0.0444	1.0925
Liberia	2027	1	45	0.0446	1.0970
Liberia	2028	1	46	0.0448	1.1014
Liberia	2029	1	48	0.0450	1.1059
Liberia	2030	1	49	0.0451	1.1084
Liberia	2031	1	50	0.0452	1.1109
Liberia	2032	1	51	0.0453	1.1134
Liberia	2033	1	53	0.0453	1.1159
Liberia	2034	1	54	0.0454	1.1183
Liberia	2035	2	56	0.0455	1.1187
Liberia	2036	2	57	0.0455	1.1191
Liberia	2037	2	58	0.0455	1.1194
Liberia	2038	2	60	0.0455	1.1197
Liberia	2039	2	61	0.0455	1.1201
Liberia	2040	2	63	0.0455	1.1204
Libya	2022	1	46	0.0273	0.7327
Libya	2023	1	47	0.0275	0.7393
Libya	2024	1	48	0.0278	0.7459
Libya	2025	1	49	0.0279	0.7504
Libya	2026	2	50	0.0281	0.7548
Libya	2027	2	51	0.0282	0.7591

Libya	2028	2	53	0.0284	0.7635
Libya	2029	2	54	0.0285	0.7678
Libya	2030	2	55	0.0286	0.7701
Libya	2031	2	56	0.0287	0.7724
Libya	2032	2	58	0.0287	0.7747
Libya	2033	2	59	0.0288	0.7770
Libya	2034	2	60	0.0288	0.7793
Libya	2035	2	62	0.0288	0.7794
Libya	2036	2	63	0.0288	0.7796
Libya	2037	2	64	0.0288	0.7798
Libya	2038	2	66	0.0288	0.7800
Libya	2039	2	67	0.0288	0.7803
Libya	2040	3	69	0.0288	0.7805
Lithuania	2022	9	162	0.1537	3.6241
Lithuania	2023	10	170	0.1632	3.8468
Lithuania	2024	10	178	0.1726	4.0691
Lithuania	2025	11	186	0.1820	4.2554
Lithuania	2026	11	195	0.1915	4.4415
Lithuania	2027	12	203	0.2009	4.6274
Lithuania	2028	12	212	0.2103	4.8129
Lithuania	2029	13	220	0.2196	4.9980
Lithuania	2030	13	228	0.2276	5.1489
Lithuania	2031	14	235	0.2355	5.2997
Lithuania	2032	14	242	0.2435	5.4504
Lithuania	2033	15	249	0.2514	5.6008
Lithuania	2034	16	256	0.2593	5.7509
Lithuania	2035	16	261	0.2643	5.8449
Lithuania	2036	16	266	0.2692	5.9388

Lithuania	2037	17	270	0.2742	6.0327
Lithuania	2038	17	275	0.2791	6.1265
Lithuania	2039	18	280	0.2840	6.2202
Lithuania	2040	18	285	0.2889	6.3137
Luxembourg	2022	3	57	0.2995	6.0658
Luxembourg	2023	3	59	0.3008	6.0987
Luxembourg	2024	4	61	0.3021	6.1317
Luxembourg	2025	4	62	0.3031	6.1536
Luxembourg	2026	4	64	0.3041	6.1754
Luxembourg	2027	4	66	0.3051	6.1970
Luxembourg	2028	4	68	0.3061	6.2185
Luxembourg	2029	4	70	0.3071	6.2400
Luxembourg	2030	4	72	0.3077	6.2539
Luxembourg	2031	4	74	0.3083	6.2676
Luxembourg	2032	5	76	0.3088	6.2810
Luxembourg	2033	5	78	0.3094	6.2942
Luxembourg	2034	5	80	0.3100	6.3073
Luxembourg	2035	5	82	0.3101	6.3112
Luxembourg	2036	5	84	0.3103	6.3149
Luxembourg	2037	5	87	0.3104	6.3184
Luxembourg	2038	6	89	0.3106	6.3219
Luxembourg	2039	6	91	0.3107	6.3252
Luxembourg	2040	6	93	0.3108	6.3284
Madagascar	2022	5	239	0.0449	1.1227
Madagascar	2023	6	249	0.0451	1.1289
Madagascar	2024	6	259	0.0452	1.1351
Madagascar	2025	6	269	0.0454	1.1409
Madagascar	2026	6	279	0.0456	1.1466

Madagascar	2027	7	290	0.0458	1.1522
Madagascar	2028	7	300	0.0460	1.1579
Madagascar	2029	7	311	0.0461	1.1636
Madagascar	2030	7	322	0.0462	1.1674
Madagascar	2031	8	333	0.0464	1.1711
Madagascar	2032	8	344	0.0465	1.1749
Madagascar	2033	8	356	0.0466	1.1787
Madagascar	2034	9	368	0.0467	1.1824
Madagascar	2035	9	379	0.0467	1.1841
Madagascar	2036	9	390	0.0468	1.1859
Madagascar	2037	10	402	0.0468	1.1876
Madagascar	2038	10	414	0.0468	1.1893
Madagascar	2039	11	426	0.0469	1.1910
Madagascar	2040	11	439	0.0469	1.1928
Malawi	2022	4	172	0.0469	1.1955
Malawi	2023	5	178	0.0472	1.2048
Malawi	2024	5	184	0.0475	1.2141
Malawi	2025	5	190	0.0478	1.2216
Malawi	2026	5	196	0.0481	1.2292
Malawi	2027	5	202	0.0483	1.2368
Malawi	2028	5	208	0.0486	1.2444
Malawi	2029	6	215	0.0489	1.2521
Malawi	2030	6	221	0.0490	1.2569
Malawi	2031	6	227	0.0492	1.2617
Malawi	2032	6	233	0.0493	1.2665
Malawi	2033	6	239	0.0495	1.2714
Malawi	2034	7	245	0.0497	1.2763
Malawi	2035	7	251	0.0497	1.2781

Malawi	2036	7	257	0.0498	1.2799
Malawi	2037	7	263	0.0498	1.2817
Malawi	2038	7	270	0.0499	1.2836
Malawi	2039	8	276	0.0499	1.2854
Malawi	2040	8	283	0.0500	1.2872
Malaysia	2022	7	180	0.0262	0.6198
Malaysia	2023	7	186	0.0263	0.6219
Malaysia	2024	7	192	0.0264	0.6240
Malaysia	2025	8	198	0.0265	0.6252
Malaysia	2026	8	204	0.0265	0.6263
Malaysia	2027	8	209	0.0265	0.6275
Malaysia	2028	8	215	0.0266	0.6287
Malaysia	2029	9	222	0.0266	0.6298
Malaysia	2030	9	227	0.0266	0.6302
Malaysia	2031	10	233	0.0266	0.6307
Malaysia	2032	10	239	0.0266	0.6311
Malaysia	2033	10	245	0.0266	0.6315
Malaysia	2034	11	251	0.0266	0.6319
Malaysia	2035	11	257	0.0266	0.6314
Malaysia	2036	11	262	0.0266	0.6310
Malaysia	2037	12	268	0.0265	0.6306
Malaysia	2038	12	274	0.0265	0.6302
Malaysia	2039	13	280	0.0265	0.6297
Malaysia	2040	13	286	0.0265	0.6293
Maldives	2022	0	2	0.0232	0.5647
Maldives	2023	0	2	0.0234	0.5684
Maldives	2024	0	2	0.0236	0.5721
Maldives	2025	0	3	0.0236	0.5744

Maldives	2026	0	3	0.0237	0.5767
Maldives	2027	0	3	0.0238	0.5790
Maldives	2028	0	3	0.0239	0.5813
Maldives	2029	0	3	0.0240	0.5836
Maldives	2030	0	3	0.0240	0.5849
Maldives	2031	0	3	0.0241	0.5861
Maldives	2032	0	3	0.0241	0.5874
Maldives	2033	0	3	0.0241	0.5887
Maldives	2034	0	3	0.0242	0.5900
Maldives	2035	0	4	0.0242	0.5901
Maldives	2036	0	4	0.0242	0.5902
Maldives	2037	0	4	0.0242	0.5903
Maldives	2038	0	4	0.0242	0.5905
Maldives	2039	0	4	0.0242	0.5906
Maldives	2040	0	4	0.0242	0.5908
Mali	2022	4	165	0.0391	0.9668
Mali	2023	4	172	0.0393	0.9723
Mali	2024	4	179	0.0395	0.9778
Mali	2025	4	186	0.0396	0.9826
Mali	2026	5	194	0.0398	0.9873
Mali	2027	5	201	0.0400	0.9921
Mali	2028	5	209	0.0401	0.9968
Mali	2029	5	218	0.0403	1.0016
Mali	2030	5	226	0.0404	1.0045
Mali	2031	6	234	0.0405	1.0074
Mali	2032	6	242	0.0406	1.0103
Mali	2033	6	251	0.0406	1.0132
Mali	2034	6	260	0.0407	1.0161

Mali	2035	7	268	0.0407	1.0171
Mali	2036	7	277	0.0408	1.0180
Mali	2037	7	286	0.0408	1.0190
Mali	2038	8	295	0.0408	1.0199
Mali	2039	8	304	0.0408	1.0208
Mali	2040	8	313	0.0408	1.0218
Malta	2022	2	35	0.2319	3.9367
Malta	2023	3	37	0.2354	3.9782
Malta	2024	3	38	0.2391	4.0198
Malta	2025	3	39	0.2417	4.0546
Malta	2026	3	41	0.2444	4.0892
Malta	2027	3	42	0.2470	4.1235
Malta	2028	3	43	0.2497	4.1578
Malta	2029	3	45	0.2523	4.1921
Malta	2030	3	46	0.2539	4.2138
Malta	2031	4	47	0.2555	4.2354
Malta	2032	4	48	0.2570	4.2569
Malta	2033	4	49	0.2586	4.2783
Malta	2034	4	50	0.2602	4.2997
Malta	2035	4	51	0.2606	4.3071
Malta	2036	4	52	0.2610	4.3143
Malta	2037	4	52	0.2614	4.3216
Malta	2038	4	53	0.2618	4.3288
Malta	2039	4	53	0.2623	4.3360
Malta	2040	4	54	0.2627	4.3432
Marshall Islands	2022	0	0	0.0300	0.7369
Marshall Islands	2023	0	0	0.0301	0.7375
Marshall Islands	2024	0	0	0.0301	0.7382

Marshall Islands	2025	0	0	0.0301	0.7387
Marshall Islands	2026	0	0	0.0301	0.7391
Marshall Islands	2027	0	0	0.0301	0.7396
Marshall Islands	2028	0	0	0.0301	0.7401
Marshall Islands	2029	0	0	0.0301	0.7407
Marshall Islands	2030	0	0	0.0301	0.7407
Marshall Islands	2031	0	0	0.0301	0.7407
Marshall Islands	2032	0	0	0.0301	0.7407
Marshall Islands	2033	0	0	0.0301	0.7407
Marshall Islands	2034	0	0	0.0301	0.7408
Marshall Islands	2035	0	0	0.0300	0.7404
Marshall Islands	2036	0	0	0.0300	0.7399
Marshall Islands	2037	0	0	0.0300	0.7395
Marshall Islands	2038	0	0	0.0299	0.7391
Marshall Islands	2039	0	0	0.0299	0.7387
Marshall Islands	2040	0	0	0.0299	0.7383
Mauritania	2022	1	39	0.0506	1.2230
Mauritania	2023	1	41	0.0508	1.2290
Mauritania	2024	1	42	0.0511	1.2349
Mauritania	2025	1	43	0.0513	1.2393
Mauritania	2026	1	45	0.0514	1.2437
Mauritania	2027	1	46	0.0516	1.2482
Mauritania	2028	1	47	0.0518	1.2526
Mauritania	2029	1	49	0.0520	1.2571
Mauritania	2030	2	50	0.0521	1.2597
Mauritania	2031	2	52	0.0521	1.2623
Mauritania	2032	2	53	0.0522	1.2649
Mauritania	2033	2	55	0.0523	1.2675

Mauritania	2034	2	57	0.0524	1.2702
Mauritania	2035	2	58	0.0524	1.2708
Mauritania	2036	2	60	0.0524	1.2714
Mauritania	2037	2	62	0.0525	1.2720
Mauritania	2038	2	64	0.0525	1.2726
Mauritania	2039	2	65	0.0525	1.2732
Mauritania	2040	2	67	0.0525	1.2739
Mauritius	2022	0	9	0.0250	0.5879
Mauritius	2023	0	9	0.0250	0.5892
Mauritius	2024	0	10	0.0251	0.5906
Mauritius	2025	0	10	0.0251	0.5913
Mauritius	2026	0	10	0.0251	0.5920
Mauritius	2027	0	10	0.0252	0.5928
Mauritius	2028	1	11	0.0252	0.5935
Mauritius	2029	1	11	0.0252	0.5942
Mauritius	2030	1	11	0.0252	0.5945
Mauritius	2031	1	11	0.0252	0.5949
Mauritius	2032	1	12	0.0252	0.5952
Mauritius	2033	1	12	0.0252	0.5955
Mauritius	2034	1	12	0.0252	0.5959
Mauritius	2035	1	12	0.0252	0.5957
Mauritius	2036	1	13	0.0252	0.5955
Mauritius	2037	1	13	0.0252	0.5954
Mauritius	2038	1	13	0.0251	0.5952
Mauritius	2039	1	13	0.0251	0.5951
Mauritius	2040	1	13	0.0251	0.5949
Mexico	2022	41	1364	0.0329	1.0547
Mexico	2023	44	1406	0.0335	1.0682

Mexico	2024	46	1449	0.0342	1.0817
Mexico	2025	48	1490	0.0346	1.0927
Mexico	2026	50	1531	0.0351	1.1037
Mexico	2027	52	1572	0.0355	1.1148
Mexico	2028	54	1614	0.0359	1.1258
Mexico	2029	56	1658	0.0363	1.1369
Mexico	2030	58	1696	0.0365	1.1436
Mexico	2031	60	1734	0.0366	1.1502
Mexico	2032	62	1772	0.0368	1.1569
Mexico	2033	64	1811	0.0370	1.1636
Mexico	2034	67	1851	0.0371	1.1703
Mexico	2035	69	1883	0.0371	1.1718
Mexico	2036	71	1915	0.0370	1.1734
Mexico	2037	73	1948	0.0369	1.1749
Mexico	2038	75	1980	0.0368	1.1764
Mexico	2039	77	2014	0.0367	1.1780
Mexico	2040	79	2047	0.0367	1.1796
Micronesia (Federated States of)	2022	0	1	0.0309	0.7551
Micronesia (Federated States of)	2023	0	1	0.0309	0.7550
Micronesia (Federated States of)	2024	0	1	0.0309	0.7548
Micronesia (Federated States of)	2025	0	1	0.0309	0.7549
Micronesia (Federated States of)	2026	0	1	0.0309	0.7550
Micronesia (Federated States of)	2027	0	1	0.0309	0.7551
Micronesia (Federated States of)	2028	0	1	0.0310	0.7552
Micronesia (Federated States of)	2029	0	1	0.0310	0.7553
Micronesia (Federated States of)	2030	0	1	0.0310	0.7552
Micronesia (Federated States of)	2031	0	1	0.0310	0.7551
Micronesia (Federated States of)	2032	0	1	0.0310	0.7550

Micronesia (Federated States of)	2033	0	1	0.0310	0.7549
Micronesia (Federated States of)	2034	0	1	0.0310	0.7548
Micronesia (Federated States of)	2035	0	1	0.0310	0.7545
Micronesia (Federated States of)	2036	0	1	0.0309	0.7542
Micronesia (Federated States of)	2037	0	1	0.0309	0.7539
Micronesia (Federated States of)	2038	0	1	0.0309	0.7536
Micronesia (Federated States of)	2039	0	1	0.0309	0.7534
Micronesia (Federated States of)	2040	0	1	0.0309	0.7531
Mongolia	2022	1	32	0.0281	0.9903
Mongolia	2023	1	33	0.0281	0.9904
Mongolia	2024	1	33	0.0281	0.9906
Mongolia	2025	1	34	0.0282	0.9912
Mongolia	2026	1	34	0.0282	0.9918
Mongolia	2027	1	35	0.0282	0.9924
Mongolia	2028	1	35	0.0283	0.9930
Mongolia	2029	1	36	0.0283	0.9937
Mongolia	2030	1	37	0.0285	0.9984
Mongolia	2031	1	37	0.0288	1.0031
Mongolia	2032	1	38	0.0290	1.0078
Mongolia	2033	1	39	0.0292	1.0126
Mongolia	2034	1	40	0.0295	1.0174
Mongolia	2035	1	41	0.0299	1.0271
Mongolia	2036	1	42	0.0304	1.0369
Mongolia	2037	1	43	0.0308	1.0467
Mongolia	2038	1	44	0.0312	1.0565
Mongolia	2039	1	45	0.0317	1.0662
Mongolia	2040	1	46	0.0321	1.0760
Montenegro	2022	0	9	0.0443	1.1409

Montenegro	2023	0	9	0.0438	1.1311
Montenegro	2024	0	9	0.0432	1.1214
Montenegro	2025	0	9	0.0431	1.1161
Montenegro	2026	0	9	0.0429	1.1107
Montenegro	2027	0	9	0.0428	1.1053
Montenegro	2028	0	9	0.0427	1.0999
Montenegro	2029	0	9	0.0425	1.0947
Montenegro	2030	0	9	0.0424	1.0913
Montenegro	2031	0	9	0.0424	1.0879
Montenegro	2032	0	9	0.0423	1.0844
Montenegro	2033	0	9	0.0422	1.0810
Montenegro	2034	0	9	0.0421	1.0777
Montenegro	2035	1	10	0.0421	1.0762
Montenegro	2036	1	10	0.0421	1.0746
Montenegro	2037	1	10	0.0421	1.0730
Montenegro	2038	1	10	0.0421	1.0714
Montenegro	2039	1	10	0.0421	1.0699
Montenegro	2040	1	10	0.0420	1.0683
Morocco	2022	7	203	0.0222	0.5892
Morocco	2023	7	208	0.0224	0.5951
Morocco	2024	7	214	0.0226	0.6010
Morocco	2025	8	219	0.0228	0.6053
Morocco	2026	8	224	0.0229	0.6095
Morocco	2027	8	230	0.0230	0.6137
Morocco	2028	8	235	0.0232	0.6180
Morocco	2029	9	241	0.0234	0.6222
Morocco	2030	9	246	0.0234	0.6245
Morocco	2031	9	251	0.0235	0.6268

Morocco	2032	10	256	0.0236	0.6291
Morocco	2033	10	261	0.0236	0.6314
Morocco	2034	10	267	0.0237	0.6337
Morocco	2035	11	272	0.0237	0.6339
Morocco	2036	11	277	0.0237	0.6340
Morocco	2037	11	282	0.0237	0.6342
Morocco	2038	12	287	0.0237	0.6343
Morocco	2039	12	293	0.0237	0.6345
Morocco	2040	13	299	0.0237	0.6346
Mozambique	2022	6	245	0.0430	1.0767
Mozambique	2023	6	253	0.0432	1.0849
Mozambique	2024	6	260	0.0435	1.0931
Mozambique	2025	6	267	0.0438	1.0998
Mozambique	2026	6	275	0.0440	1.1065
Mozambique	2027	7	282	0.0443	1.1132
Mozambique	2028	7	290	0.0445	1.1199
Mozambique	2029	7	298	0.0447	1.1267
Mozambique	2030	7	306	0.0449	1.1308
Mozambique	2031	8	313	0.0450	1.1349
Mozambique	2032	8	321	0.0451	1.1391
Mozambique	2033	8	329	0.0453	1.1432
Mozambique	2034	8	337	0.0454	1.1475
Mozambique	2035	9	345	0.0454	1.1488
Mozambique	2036	9	352	0.0455	1.1501
Mozambique	2037	9	360	0.0455	1.1514
Mozambique	2038	10	367	0.0455	1.1528
Mozambique	2039	10	375	0.0456	1.1542
Mozambique	2040	10	383	0.0456	1.1556

Myanmar	2022	9	281	0.0209	0.5770
Myanmar	2023	9	287	0.0210	0.5795
Myanmar	2024	9	293	0.0211	0.5821
Myanmar	2025	10	299	0.0211	0.5838
Myanmar	2026	10	306	0.0212	0.5855
Myanmar	2027	10	312	0.0212	0.5873
Myanmar	2028	11	319	0.0213	0.5890
Myanmar	2029	11	326	0.0213	0.5907
Myanmar	2030	12	333	0.0213	0.5918
Myanmar	2031	12	340	0.0213	0.5928
Myanmar	2032	12	346	0.0214	0.5938
Myanmar	2033	13	353	0.0214	0.5948
Myanmar	2034	13	360	0.0214	0.5959
Myanmar	2035	14	367	0.0214	0.5962
Myanmar	2036	14	374	0.0214	0.5965
Myanmar	2037	15	380	0.0214	0.5968
Myanmar	2038	15	386	0.0214	0.5971
Myanmar	2039	15	393	0.0214	0.5974
Myanmar	2040	16	399	0.0214	0.5977
Namibia	2022	1	32	0.0623	1.5564
Namibia	2023	1	32	0.0626	1.5661
Namibia	2024	1	33	0.0630	1.5759
Namibia	2025	1	34	0.0632	1.5835
Namibia	2026	1	35	0.0635	1.5912
Namibia	2027	1	36	0.0637	1.5989
Namibia	2028	1	37	0.0640	1.6067
Namibia	2029	1	38	0.0642	1.6146
Namibia	2030	1	39	0.0644	1.6196

Namibia	2031	1	40	0.0646	1.6247
Namibia	2032	1	41	0.0647	1.6297
Namibia	2033	1	43	0.0649	1.6348
Namibia	2034	1	44	0.0650	1.6400
Namibia	2035	2	45	0.0651	1.6420
Namibia	2036	2	46	0.0651	1.6441
Namibia	2037	2	47	0.0651	1.6462
Namibia	2038	2	48	0.0652	1.6483
Namibia	2039	2	49	0.0652	1.6505
Namibia	2040	2	51	0.0653	1.6526
Nepal	2022	7	227	0.0317	0.8446
Nepal	2023	7	231	0.0318	0.8457
Nepal	2024	7	235	0.0319	0.8468
Nepal	2025	8	238	0.0319	0.8470
Nepal	2026	8	242	0.0319	0.8472
Nepal	2027	8	246	0.0319	0.8475
Nepal	2028	8	250	0.0320	0.8478
Nepal	2029	8	253	0.0320	0.8481
Nepal	2030	9	257	0.0320	0.8471
Nepal	2031	9	261	0.0320	0.8462
Nepal	2032	9	264	0.0319	0.8454
Nepal	2033	9	268	0.0319	0.8445
Nepal	2034	10	272	0.0319	0.8437
Nepal	2035	10	275	0.0318	0.8415
Nepal	2036	10	278	0.0317	0.8394
Nepal	2037	10	281	0.0316	0.8373
Nepal	2038	11	285	0.0315	0.8352
Nepal	2039	11	288	0.0315	0.8331

Nepal	2040	11	292	0.0314	0.8310
Netherlands	2022	75	1155	0.1903	3.5222
Netherlands	2023	77	1186	0.1913	3.5463
Netherlands	2024	80	1220	0.1923	3.5704
Netherlands	2025	82	1254	0.1930	3.5879
Netherlands	2026	85	1288	0.1936	3.6052
Netherlands	2027	88	1321	0.1943	3.6225
Netherlands	2028	90	1356	0.1949	3.6397
Netherlands	2029	94	1392	0.1956	3.6570
Netherlands	2030	97	1427	0.1960	3.6683
Netherlands	2031	100	1462	0.1963	3.6795
Netherlands	2032	102	1495	0.1967	3.6906
Netherlands	2033	105	1527	0.1970	3.7017
Netherlands	2034	108	1559	0.1974	3.7127
Netherlands	2035	111	1589	0.1975	3.7183
Netherlands	2036	114	1617	0.1975	3.7238
Netherlands	2037	116	1642	0.1976	3.7292
Netherlands	2038	119	1665	0.1977	3.7346
Netherlands	2039	121	1687	0.1978	3.7400
Netherlands	2040	123	1707	0.1978	3.7454
New Zealand	2022	43	714	0.4674	8.9458
New Zealand	2023	45	746	0.4756	9.0822
New Zealand	2024	48	779	0.4838	9.2187
New Zealand	2025	50	809	0.4890	9.3169
New Zealand	2026	52	840	0.4941	9.4150
New Zealand	2027	54	870	0.4993	9.5129
New Zealand	2028	56	902	0.5044	9.6108
New Zealand	2029	59	935	0.5095	9.7089

New Zealand	2030	61	964	0.5122	9.7646
New Zealand	2031	63	993	0.5148	9.8201
New Zealand	2032	66	1022	0.5174	9.8752
New Zealand	2033	68	1051	0.5200	9.9300
New Zealand	2034	70	1080	0.5226	9.9847
New Zealand	2035	72	1105	0.5229	9.9950
New Zealand	2036	74	1129	0.5231	10.0049
New Zealand	2037	76	1151	0.5232	10.0145
New Zealand	2038	78	1173	0.5234	10.0239
New Zealand	2039	80	1195	0.5236	10.0330
New Zealand	2040	82	1216	0.5237	10.0421
Nicaragua	2022	2	67	0.0288	1.0686
Nicaragua	2023	2	68	0.0290	1.0777
Nicaragua	2024	2	69	0.0293	1.0868
Nicaragua	2025	2	70	0.0294	1.0931
Nicaragua	2026	2	72	0.0296	1.0995
Nicaragua	2027	2	73	0.0297	1.1058
Nicaragua	2028	2	74	0.0298	1.1122
Nicaragua	2029	2	75	0.0300	1.1185
Nicaragua	2030	2	76	0.0301	1.1225
Nicaragua	2031	2	77	0.0302	1.1264
Nicaragua	2032	2	78	0.0302	1.1304
Nicaragua	2033	2	79	0.0303	1.1343
Nicaragua	2034	2	80	0.0304	1.1383
Nicaragua	2035	3	81	0.0304	1.1400
Nicaragua	2036	3	82	0.0304	1.1417
Nicaragua	2037	3	83	0.0304	1.1435
Nicaragua	2038	3	84	0.0305	1.1452

Nicaragua	2039	3	85	0.0305	1.1469
Nicaragua	2040	3	86	0.0305	1.1486
Niger	2022	3	151	0.0334	0.8195
Niger	2023	3	158	0.0335	0.8216
Niger	2024	4	165	0.0335	0.8238
Niger	2025	4	173	0.0336	0.8258
Niger	2026	4	181	0.0337	0.8278
Niger	2027	4	189	0.0338	0.8298
Niger	2028	4	197	0.0338	0.8318
Niger	2029	4	206	0.0339	0.8338
Niger	2030	5	216	0.0339	0.8348
Niger	2031	5	225	0.0340	0.8358
Niger	2032	5	235	0.0340	0.8368
Niger	2033	5	244	0.0340	0.8378
Niger	2034	6	255	0.0341	0.8388
Niger	2035	6	265	0.0340	0.8388
Niger	2036	6	276	0.0340	0.8389
Niger	2037	6	286	0.0340	0.8389
Niger	2038	7	298	0.0340	0.8390
Niger	2039	7	309	0.0340	0.8390
Niger	2040	7	321	0.0340	0.8391
Nigeria	2022	63	2667	0.0585	1.5248
Nigeria	2023	66	2783	0.0590	1.5390
Nigeria	2024	69	2905	0.0594	1.5534
Nigeria	2025	73	3025	0.0597	1.5634
Nigeria	2026	76	3151	0.0601	1.5734
Nigeria	2027	80	3281	0.0604	1.5835
Nigeria	2028	83	3416	0.0607	1.5936

Nigeria	2029	87	3558	0.0610	1.6038
Nigeria	2030	91	3695	0.0612	1.6100
Nigeria	2031	95	3837	0.0614	1.6162
Nigeria	2032	100	3982	0.0616	1.6225
Nigeria	2033	104	4131	0.0618	1.6288
Nigeria	2034	109	4284	0.0619	1.6352
Nigeria	2035	114	4429	0.0620	1.6374
Nigeria	2036	119	4576	0.0620	1.6397
Nigeria	2037	124	4725	0.0620	1.6419
Nigeria	2038	129	4876	0.0620	1.6442
Nigeria	2039	134	5030	0.0621	1.6466
Nigeria	2040	140	5186	0.0621	1.6489
North Macedonia	2022	14	260	0.3804	8.2933
North Macedonia	2023	14	267	0.3828	8.3486
North Macedonia	2024	15	275	0.3852	8.4053
North Macedonia	2025	15	282	0.3863	8.4393
North Macedonia	2026	16	289	0.3874	8.4735
North Macedonia	2027	16	296	0.3885	8.5075
North Macedonia	2028	17	303	0.3896	8.5418
North Macedonia	2029	18	310	0.3908	8.5768
North Macedonia	2030	18	316	0.3910	8.5904
North Macedonia	2031	19	322	0.3913	8.6040
North Macedonia	2032	19	328	0.3915	8.6176
North Macedonia	2033	20	334	0.3917	8.6313
North Macedonia	2034	20	340	0.3920	8.6453
North Macedonia	2035	21	345	0.3913	8.6374
North Macedonia	2036	21	350	0.3906	8.6295
North Macedonia	2037	22	354	0.3899	8.6214

North Macedonia	2038	22	359	0.3892	8.6135
North Macedonia	2039	23	364	0.3885	8.6058
North Macedonia	2040	23	369	0.3879	8.5981
Northern Mariana Islands	2022	0	0	0.0367	0.8435
Northern Mariana Islands	2023	0	0	0.0367	0.8415
Northern Mariana Islands	2024	0	0	0.0367	0.8398
Northern Mariana Islands	2025	0	0	0.0366	0.8386
Northern Mariana Islands	2026	0	0	0.0366	0.8374
Northern Mariana Islands	2027	0	0	0.0365	0.8361
Northern Mariana Islands	2028	0	1	0.0365	0.8349
Northern Mariana Islands	2029	0	1	0.0365	0.8340
Northern Mariana Islands	2030	0	1	0.0365	0.8333
Northern Mariana Islands	2031	0	1	0.0365	0.8328
Northern Mariana Islands	2032	0	1	0.0364	0.8318
Northern Mariana Islands	2033	0	1	0.0364	0.8303
Northern Mariana Islands	2034	0	1	0.0364	0.8290
Northern Mariana Islands	2035	0	1	0.0364	0.8280
Northern Mariana Islands	2036	0	1	0.0363	0.8275
Northern Mariana Islands	2037	0	1	0.0363	0.8266
Northern Mariana Islands	2038	0	1	0.0362	0.8250
Northern Mariana Islands	2039	0	1	0.0362	0.8237
Northern Mariana Islands	2040	0	1	0.0362	0.8234
Norway	2022	103	1650	0.9004	15.9658
Norway	2023	106	1679	0.8954	15.7804
Norway	2024	109	1709	0.8905	15.5944
Norway	2025	112	1742	0.8855	15.4557
Norway	2026	115	1774	0.8805	15.3163
Norway	2027	118	1805	0.8754	15.1762

Norway	2028	121	1836	0.8703	15.0355
Norway	2029	124	1867	0.8652	14.8943
Norway	2030	127	1901	0.8610	14.8012
Norway	2031	130	1935	0.8568	14.7080
Norway	2032	133	1967	0.8525	14.6147
Norway	2033	136	1998	0.8483	14.5213
Norway	2034	139	2029	0.8441	14.4279
Norway	2035	143	2065	0.8413	14.3820
Norway	2036	146	2099	0.8386	14.3360
Norway	2037	149	2132	0.8359	14.2900
Norway	2038	151	2163	0.8331	14.2439
Norway	2039	154	2194	0.8304	14.1977
Norway	2040	157	2225	0.8277	14.1515
Oman	2022	1	36	0.0339	0.8838
Oman	2023	1	38	0.0344	0.8968
Oman	2024	1	40	0.0349	0.9096
Oman	2025	1	42	0.0353	0.9190
Oman	2026	1	43	0.0356	0.9284
Oman	2027	1	45	0.0360	0.9377
Oman	2028	1	47	0.0363	0.9470
Oman	2029	1	49	0.0366	0.9563
Oman	2030	2	51	0.0368	0.9617
Oman	2031	2	53	0.0370	0.9670
Oman	2032	2	54	0.0372	0.9725
Oman	2033	2	56	0.0374	0.9780
Oman	2034	2	58	0.0376	0.9835
Oman	2035	2	60	0.0377	0.9847
Oman	2036	2	62	0.0377	0.9859

Oman	2037	2	64	0.0378	0.9871
Oman	2038	2	66	0.0378	0.9884
Oman	2039	2	69	0.0379	0.9898
Oman	2040	3	71	0.0379	0.9910
Pakistan	2022	35	1521	0.0274	0.7999
Pakistan	2023	37	1557	0.0275	0.8047
Pakistan	2024	38	1594	0.0277	0.8095
Pakistan	2025	39	1629	0.0278	0.8129
Pakistan	2026	40	1664	0.0279	0.8163
Pakistan	2027	41	1698	0.0281	0.8197
Pakistan	2028	42	1734	0.0282	0.8231
Pakistan	2029	44	1770	0.0283	0.8265
Pakistan	2030	45	1803	0.0283	0.8280
Pakistan	2031	46	1836	0.0284	0.8294
Pakistan	2032	48	1869	0.0284	0.8308
Pakistan	2033	49	1902	0.0285	0.8323
Pakistan	2034	50	1937	0.0285	0.8337
Pakistan	2035	52	1967	0.0285	0.8331
Pakistan	2036	53	1997	0.0284	0.8325
Pakistan	2037	55	2027	0.0284	0.8318
Pakistan	2038	56	2057	0.0284	0.8312
Pakistan	2039	58	2088	0.0283	0.8306
Pakistan	2040	59	2119	0.0283	0.8300
Palestine	2022	1	24	0.0234	0.5784
Palestine	2023	1	25	0.0237	0.5852
Palestine	2024	1	26	0.0240	0.5919
Palestine	2025	1	27	0.0242	0.5976
Palestine	2026	1	28	0.0244	0.6032

Palestine	2027	1	29	0.0247	0.6089
Palestine	2028	1	30	0.0249	0.6145
Palestine	2029	1	31	0.0251	0.6201
Palestine	2030	1	32	0.0253	0.6233
Palestine	2031	1	33	0.0254	0.6265
Palestine	2032	1	34	0.0255	0.6297
Palestine	2033	1	35	0.0257	0.6328
Palestine	2034	1	36	0.0258	0.6360
Palestine	2035	1	37	0.0258	0.6365
Palestine	2036	1	38	0.0258	0.6370
Palestine	2037	1	39	0.0258	0.6375
Palestine	2038	1	40	0.0258	0.6379
Palestine	2039	1	41	0.0258	0.6383
Palestine	2040	2	43	0.0258	0.6387
Panama	2022	2	56	0.0376	1.3627
Panama	2023	2	58	0.0378	1.3723
Panama	2024	2	59	0.0380	1.3819
Panama	2025	2	60	0.0382	1.3882
Panama	2026	2	62	0.0383	1.3945
Panama	2027	2	63	0.0385	1.4008
Panama	2028	2	65	0.0386	1.4070
Panama	2029	2	66	0.0387	1.4133
Panama	2030	2	68	0.0388	1.4171
Panama	2031	2	69	0.0389	1.4209
Panama	2032	2	70	0.0389	1.4247
Panama	2033	2	72	0.0390	1.4284
Panama	2034	2	73	0.0391	1.4322
Panama	2035	3	74	0.0391	1.4339

Panama	2036	3	76	0.0391	1.4357
Panama	2037	3	77	0.0391	1.4374
Panama	2038	3	78	0.0391	1.4391
Panama	2039	3	79	0.0391	1.4409
Panama	2040	3	81	0.0391	1.4426
Papua New Guinea	2022	1	47	0.0237	0.6027
Papua New Guinea	2023	1	48	0.0237	0.6033
Papua New Guinea	2024	1	49	0.0238	0.6039
Papua New Guinea	2025	1	51	0.0238	0.6040
Papua New Guinea	2026	1	52	0.0238	0.6041
Papua New Guinea	2027	1	53	0.0238	0.6042
Papua New Guinea	2028	1	54	0.0238	0.6042
Papua New Guinea	2029	1	55	0.0238	0.6043
Papua New Guinea	2030	1	56	0.0238	0.6040
Papua New Guinea	2031	1	57	0.0238	0.6037
Papua New Guinea	2032	1	59	0.0238	0.6035
Papua New Guinea	2033	1	60	0.0238	0.6032
Papua New Guinea	2034	1	61	0.0237	0.6029
Papua New Guinea	2035	1	62	0.0237	0.6023
Papua New Guinea	2036	2	63	0.0237	0.6017
Papua New Guinea	2037	2	65	0.0237	0.6012
Papua New Guinea	2038	2	66	0.0236	0.6006
Papua New Guinea	2039	2	67	0.0236	0.6000
Papua New Guinea	2040	2	69	0.0236	0.5995
Paraguay	2022	1	41	0.0140	0.5748
Paraguay	2023	1	42	0.0141	0.5799
Paraguay	2024	1	43	0.0142	0.5850
Paraguay	2025	1	43	0.0143	0.5888

Paraguay	2026	1	44	0.0144	0.5927
Paraguay	2027	1	45	0.0145	0.5965
Paraguay	2028	1	45	0.0145	0.6003
Paraguay	2029	1	46	0.0146	0.6041
Paraguay	2030	1	47	0.0147	0.6065
Paraguay	2031	1	47	0.0147	0.6089
Paraguay	2032	1	48	0.0148	0.6113
Paraguay	2033	1	48	0.0148	0.6136
Paraguay	2034	1	49	0.0148	0.6160
Paraguay	2035	1	49	0.0148	0.6170
Paraguay	2036	1	49	0.0148	0.6180
Paraguay	2037	1	50	0.0148	0.6190
Paraguay	2038	1	50	0.0148	0.6200
Paraguay	2039	1	51	0.0149	0.6209
Paraguay	2040	1	51	0.0149	0.6219
Peru	2022	5	193	0.0134	0.5264
Peru	2023	5	197	0.0135	0.5289
Peru	2024	5	202	0.0135	0.5314
Peru	2025	5	205	0.0135	0.5329
Peru	2026	6	209	0.0136	0.5345
Peru	2027	6	213	0.0136	0.5361
Peru	2028	6	216	0.0136	0.5377
Peru	2029	6	220	0.0137	0.5392
Peru	2030	6	223	0.0137	0.5402
Peru	2031	6	226	0.0137	0.5411
Peru	2032	7	230	0.0137	0.5420
Peru	2033	7	233	0.0137	0.5429
Peru	2034	7	236	0.0137	0.5439

Peru	2035	7	239	0.0137	0.5442
Peru	2036	7	242	0.0137	0.5446
Peru	2037	8	246	0.0137	0.5449
Peru	2038	8	249	0.0137	0.5452
Peru	2039	8	252	0.0137	0.5456
Peru	2040	8	256	0.0137	0.5459
Philippines	2022	17	630	0.0238	0.6580
Philippines	2023	18	642	0.0238	0.6564
Philippines	2024	18	653	0.0237	0.6549
Philippines	2025	19	666	0.0237	0.6537
Philippines	2026	19	678	0.0237	0.6525
Philippines	2027	20	689	0.0237	0.6513
Philippines	2028	20	701	0.0236	0.6501
Philippines	2029	21	713	0.0236	0.6490
Philippines	2030	22	726	0.0236	0.6482
Philippines	2031	22	738	0.0236	0.6474
Philippines	2032	23	750	0.0236	0.6466
Philippines	2033	23	762	0.0236	0.6458
Philippines	2034	24	774	0.0236	0.6450
Philippines	2035	25	787	0.0236	0.6447
Philippines	2036	26	800	0.0236	0.6444
Philippines	2037	26	813	0.0236	0.6440
Philippines	2038	27	826	0.0236	0.6437
Philippines	2039	28	839	0.0236	0.6433
Philippines	2040	29	853	0.0236	0.6430
Poland	2022	357	6092	0.4538	9.1152
Poland	2023	379	6417	0.4711	9.3729
Poland	2024	404	6774	0.4885	9.6330

Poland	2025	426	7058	0.5012	9.8071
Poland	2026	448	7348	0.5138	9.9818
Poland	2027	471	7639	0.5265	10.1568
Poland	2028	495	7943	0.5392	10.3329
Poland	2029	522	8274	0.5521	10.5109
Poland	2030	545	8516	0.5590	10.5762
Poland	2031	568	8760	0.5659	10.6417
Poland	2032	591	9001	0.5727	10.7070
Poland	2033	616	9251	0.5796	10.7729
Poland	2034	644	9522	0.5866	10.8401
Poland	2035	666	9683	0.5871	10.7953
Poland	2036	687	9839	0.5876	10.7500
Poland	2037	707	9981	0.5880	10.7040
Poland	2038	727	10122	0.5884	10.6581
Poland	2039	748	10274	0.5889	10.6127
Poland	2040	770	10430	0.5894	10.5674
Portugal	2022	55	769	0.1787	3.1876
Portugal	2023	57	788	0.1798	3.2066
Portugal	2024	59	808	0.1808	3.2256
Portugal	2025	60	825	0.1814	3.2378
Portugal	2026	62	843	0.1819	3.2499
Portugal	2027	63	860	0.1825	3.2621
Portugal	2028	65	878	0.1831	3.2742
Portugal	2029	67	896	0.1836	3.2864
Portugal	2030	68	914	0.1838	3.2912
Portugal	2031	70	931	0.1839	3.2960
Portugal	2032	72	947	0.1841	3.3007
Portugal	2033	73	964	0.1842	3.3055

Portugal	2034	75	982	0.1844	3.3103
Portugal	2035	77	998	0.1842	3.3084
Portugal	2036	78	1014	0.1840	3.3064
Portugal	2037	80	1029	0.1837	3.3045
Portugal	2038	81	1044	0.1835	3.3025
Portugal	2039	83	1059	0.1833	3.3006
Portugal	2040	84	1074	0.1831	3.2987
Puerto Rico	2022	1	20	0.0134	0.4882
Puerto Rico	2023	1	20	0.0135	0.4918
Puerto Rico	2024	1	21	0.0136	0.4954
Puerto Rico	2025	1	21	0.0136	0.4975
Puerto Rico	2026	1	21	0.0137	0.4996
Puerto Rico	2027	1	21	0.0137	0.5017
Puerto Rico	2028	1	22	0.0138	0.5038
Puerto Rico	2029	1	22	0.0138	0.5060
Puerto Rico	2030	1	22	0.0138	0.5073
Puerto Rico	2031	1	22	0.0139	0.5087
Puerto Rico	2032	1	22	0.0139	0.5100
Puerto Rico	2033	1	23	0.0139	0.5114
Puerto Rico	2034	1	23	0.0139	0.5127
Puerto Rico	2035	1	23	0.0139	0.5133
Puerto Rico	2036	1	23	0.0139	0.5138
Puerto Rico	2037	1	23	0.0139	0.5143
Puerto Rico	2038	1	23	0.0139	0.5149
Puerto Rico	2039	1	23	0.0138	0.5154
Puerto Rico	2040	1	23	0.0138	0.5159
Qatar	2022	0	19	0.0355	0.8740
Qatar	2023	0	20	0.0358	0.8811

Qatar	2024	1	21	0.0361	0.8882
Qatar	2025	1	21	0.0363	0.8927
Qatar	2026	1	22	0.0365	0.8969
Qatar	2027	1	23	0.0367	0.9013
Qatar	2028	1	24	0.0370	0.9062
Qatar	2029	1	25	0.0372	0.9111
Qatar	2030	1	26	0.0373	0.9128
Qatar	2031	1	26	0.0374	0.9142
Qatar	2032	1	27	0.0374	0.9158
Qatar	2033	1	28	0.0376	0.9178
Qatar	2034	1	29	0.0377	0.9199
Qatar	2035	1	31	0.0376	0.9185
Qatar	2036	1	32	0.0376	0.9168
Qatar	2037	1	33	0.0375	0.9153
Qatar	2038	1	34	0.0375	0.9143
Qatar	2039	1	35	0.0375	0.9132
Qatar	2040	1	37	0.0374	0.9120
Republic of Korea	2022	231	4756	0.2574	6.7652
Republic of Korea	2023	242	4893	0.2584	6.7688
Republic of Korea	2024	256	5043	0.2596	6.7732
Republic of Korea	2025	269	5194	0.2603	6.7710
Republic of Korea	2026	282	5345	0.2611	6.7684
Republic of Korea	2027	295	5493	0.2618	6.7651
Republic of Korea	2028	308	5643	0.2625	6.7618
Republic of Korea	2029	322	5800	0.2632	6.7589
Republic of Korea	2030	336	5955	0.2637	6.7508
Republic of Korea	2031	351	6110	0.2641	6.7424
Republic of Korea	2032	365	6264	0.2644	6.7334

Republic of Korea	2033	380	6422	0.2648	6.7242
Republic of Korea	2034	396	6588	0.2651	6.7149
Republic of Korea	2035	412	6750	0.2651	6.7009
Republic of Korea	2036	429	6915	0.2651	6.6866
Republic of Korea	2037	446	7082	0.2651	6.6720
Republic of Korea	2038	464	7255	0.2650	6.6571
Republic of Korea	2039	483	7441	0.2649	6.6421
Republic of Korea	2040	504	7632	0.2649	6.6270
Republic of Moldova	2022	6	137	0.1025	2.8842
Republic of Moldova	2023	6	140	0.1035	2.9104
Republic of Moldova	2024	6	143	0.1045	2.9367
Republic of Moldova	2025	6	147	0.1058	2.9643
Republic of Moldova	2026	7	150	0.1070	2.9919
Republic of Moldova	2027	7	154	0.1083	3.0196
Republic of Moldova	2028	7	157	0.1095	3.0473
Republic of Moldova	2029	7	160	0.1108	3.0749
Republic of Moldova	2030	8	164	0.1124	3.1095
Republic of Moldova	2031	8	167	0.1139	3.1442
Republic of Moldova	2032	8	171	0.1155	3.1792
Republic of Moldova	2033	8	174	0.1171	3.2141
Republic of Moldova	2034	9	177	0.1187	3.2492
Republic of Moldova	2035	9	181	0.1207	3.2930
Republic of Moldova	2036	9	184	0.1228	3.3368
Republic of Moldova	2037	9	187	0.1248	3.3807
Republic of Moldova	2038	10	191	0.1268	3.4245
Republic of Moldova	2039	10	194	0.1288	3.4683
Republic of Moldova	2040	10	198	0.1308	3.5121
Romania	2022	155	2853	0.3990	9.0579

Romania	2023	158	2916	0.4030	9.1273
Romania	2024	162	2987	0.4070	9.1973
Romania	2025	165	3034	0.4093	9.2367
Romania	2026	169	3081	0.4116	9.2765
Romania	2027	172	3126	0.4139	9.3165
Romania	2028	175	3175	0.4163	9.3568
Romania	2029	180	3232	0.4187	9.3978
Romania	2030	183	3267	0.4190	9.3842
Romania	2031	186	3302	0.4194	9.3709
Romania	2032	190	3336	0.4198	9.3576
Romania	2033	193	3374	0.4201	9.3444
Romania	2034	197	3420	0.4206	9.3318
Romania	2035	201	3444	0.4191	9.2689
Romania	2036	204	3467	0.4176	9.2061
Romania	2037	207	3486	0.4161	9.1430
Romania	2038	211	3505	0.4146	9.0798
Romania	2039	214	3529	0.4131	9.0168
Romania	2040	218	3556	0.4116	8.9540
Russian Federation	2022	279	6369	0.1226	3.3750
Russian Federation	2023	286	6454	0.1238	3.3956
Russian Federation	2024	293	6540	0.1250	3.4161
Russian Federation	2025	301	6625	0.1263	3.4310
Russian Federation	2026	310	6710	0.1277	3.4458
Russian Federation	2027	318	6793	0.1290	3.4606
Russian Federation	2028	327	6878	0.1304	3.4756
Russian Federation	2029	336	6967	0.1317	3.4907
Russian Federation	2030	346	7049	0.1331	3.5036
Russian Federation	2031	356	7128	0.1345	3.5165

Russian Federation	2032	365	7200	0.1359	3.5295
Russian Federation	2033	374	7268	0.1372	3.5424
Russian Federation	2034	383	7334	0.1386	3.5553
Russian Federation	2035	392	7393	0.1399	3.5660
Russian Federation	2036	401	7454	0.1412	3.5768
Russian Federation	2037	410	7517	0.1425	3.5877
Russian Federation	2038	420	7584	0.1438	3.5986
Russian Federation	2039	429	7657	0.1451	3.6095
Russian Federation	2040	439	7732	0.1464	3.6205
Rwanda	2022	3	126	0.0485	1.2148
Rwanda	2023	3	131	0.0487	1.2208
Rwanda	2024	3	135	0.0490	1.2268
Rwanda	2025	4	140	0.0491	1.2300
Rwanda	2026	4	145	0.0493	1.2332
Rwanda	2027	4	149	0.0495	1.2364
Rwanda	2028	4	154	0.0496	1.2397
Rwanda	2029	4	159	0.0498	1.2429
Rwanda	2030	5	164	0.0499	1.2444
Rwanda	2031	5	169	0.0499	1.2459
Rwanda	2032	5	174	0.0500	1.2474
Rwanda	2033	5	179	0.0501	1.2489
Rwanda	2034	5	184	0.0502	1.2504
Rwanda	2035	6	189	0.0502	1.2501
Rwanda	2036	6	194	0.0502	1.2498
Rwanda	2037	6	200	0.0502	1.2495
Rwanda	2038	6	205	0.0502	1.2493
Rwanda	2039	7	211	0.0503	1.2490
Rwanda	2040	7	217	0.0503	1.2488

Saint Lucia	2022	0	1	0.0119	0.4667
Saint Lucia	2023	0	1	0.0120	0.4704
Saint Lucia	2024	0	1	0.0121	0.4742
Saint Lucia	2025	0	1	0.0121	0.4768
Saint Lucia	2026	0	1	0.0122	0.4794
Saint Lucia	2027	0	1	0.0123	0.4821
Saint Lucia	2028	0	1	0.0123	0.4847
Saint Lucia	2029	0	1	0.0124	0.4873
Saint Lucia	2030	0	1	0.0124	0.4889
Saint Lucia	2031	0	1	0.0124	0.4905
Saint Lucia	2032	0	1	0.0124	0.4921
Saint Lucia	2033	0	1	0.0125	0.4936
Saint Lucia	2034	0	1	0.0125	0.4952
Saint Lucia	2035	0	1	0.0125	0.4957
Saint Lucia	2036	0	1	0.0125	0.4962
Saint Lucia	2037	0	1	0.0125	0.4967
Saint Lucia	2038	0	1	0.0125	0.4972
Saint Lucia	2039	0	1	0.0125	0.4977
Saint Lucia	2040	0	1	0.0125	0.4982
Saint Vincent and the Grenadines	2022	0	1	0.0115	0.4673
Saint Vincent and the Grenadines	2023	0	1	0.0116	0.4709
Saint Vincent and the Grenadines	2024	0	1	0.0117	0.4744
Saint Vincent and the Grenadines	2025	0	1	0.0117	0.4768
Saint Vincent and the Grenadines	2026	0	1	0.0118	0.4792
Saint Vincent and the Grenadines	2027	0	1	0.0118	0.4816
Saint Vincent and the Grenadines	2028	0	1	0.0119	0.4839
Saint Vincent and the Grenadines	2029	0	1	0.0119	0.4863
Saint Vincent and the Grenadines	2030	0	1	0.0119	0.4877

Saint Vincent and the Grenadines	2031	0	1	0.0120	0.4891
Saint Vincent and the Grenadines	2032	0	1	0.0120	0.4905
Saint Vincent and the Grenadines	2033	0	1	0.0120	0.4919
Saint Vincent and the Grenadines	2034	0	1	0.0120	0.4933
Saint Vincent and the Grenadines	2035	0	1	0.0120	0.4937
Saint Vincent and the Grenadines	2036	0	1	0.0120	0.4942
Saint Vincent and the Grenadines	2037	0	1	0.0120	0.4947
Saint Vincent and the Grenadines	2038	0	1	0.0120	0.4951
Saint Vincent and the Grenadines	2039	0	1	0.0120	0.4956
Saint Vincent and the Grenadines	2040	0	1	0.0120	0.4960
Samoa	2022	0	1	0.0297	0.7166
Samoa	2023	0	1	0.0297	0.7177
Samoa	2024	0	1	0.0298	0.7188
Samoa	2025	0	1	0.0298	0.7197
Samoa	2026	0	1	0.0299	0.7206
Samoa	2027	0	1	0.0299	0.7215
Samoa	2028	0	1	0.0299	0.7223
Samoa	2029	0	2	0.0300	0.7232
Samoa	2030	0	2	0.0300	0.7236
Samoa	2031	0	2	0.0300	0.7241
Samoa	2032	0	2	0.0301	0.7245
Samoa	2033	0	2	0.0301	0.7250
Samoa	2034	0	2	0.0301	0.7255
Samoa	2035	0	2	0.0301	0.7255
Samoa	2036	0	2	0.0301	0.7255
Samoa	2037	0	2	0.0301	0.7255
Samoa	2038	0	2	0.0301	0.7255
Samoa	2039	0	2	0.0301	0.7255

Samoa	2040	0	2	0.0301	0.7255
Sao Tome and Principe	2022	0	2	0.0519	1.2439
Sao Tome and Principe	2023	0	2	0.0523	1.2567
Sao Tome and Principe	2024	0	2	0.0528	1.2695
Sao Tome and Principe	2025	0	2	0.0531	1.2777
Sao Tome and Principe	2026	0	2	0.0534	1.2859
Sao Tome and Principe	2027	0	2	0.0537	1.2941
Sao Tome and Principe	2028	0	2	0.0540	1.3023
Sao Tome and Principe	2029	0	2	0.0543	1.3105
Sao Tome and Principe	2030	0	2	0.0545	1.3153
Sao Tome and Principe	2031	0	2	0.0547	1.3202
Sao Tome and Principe	2032	0	2	0.0549	1.3250
Sao Tome and Principe	2033	0	3	0.0550	1.3299
Sao Tome and Principe	2034	0	3	0.0552	1.3348
Sao Tome and Principe	2035	0	3	0.0552	1.3361
Sao Tome and Principe	2036	0	3	0.0553	1.3374
Sao Tome and Principe	2037	0	3	0.0553	1.3388
Sao Tome and Principe	2038	0	3	0.0554	1.3401
Sao Tome and Principe	2039	0	3	0.0554	1.3414
Sao Tome and Principe	2040	0	3	0.0554	1.3428
Saudi Arabia	2022	7	269	0.0336	0.9053
Saudi Arabia	2023	7	280	0.0341	0.9181
Saudi Arabia	2024	8	292	0.0346	0.9311
Saudi Arabia	2025	8	302	0.0349	0.9400
Saudi Arabia	2026	8	313	0.0352	0.9489
Saudi Arabia	2027	9	323	0.0355	0.9576
Saudi Arabia	2028	9	334	0.0358	0.9663
Saudi Arabia	2029	10	346	0.0361	0.9750

Saudi Arabia	2030	10	356	0.0363	0.9799
Saudi Arabia	2031	11	367	0.0364	0.9847
Saudi Arabia	2032	12	378	0.0366	0.9894
Saudi Arabia	2033	12	390	0.0367	0.9941
Saudi Arabia	2034	13	402	0.0369	0.9988
Saudi Arabia	2035	13	412	0.0369	0.9994
Saudi Arabia	2036	14	424	0.0368	0.9999
Saudi Arabia	2037	15	435	0.0368	1.0004
Saudi Arabia	2038	16	447	0.0368	1.0010
Saudi Arabia	2039	16	460	0.0368	1.0016
Saudi Arabia	2040	17	474	0.0368	1.0022
Senegal	2022	4	134	0.0463	1.1209
Senegal	2023	4	138	0.0466	1.1291
Senegal	2024	4	143	0.0469	1.1372
Senegal	2025	4	148	0.0471	1.1432
Senegal	2026	4	153	0.0473	1.1492
Senegal	2027	4	158	0.0475	1.1552
Senegal	2028	5	163	0.0477	1.1612
Senegal	2029	5	168	0.0479	1.1672
Senegal	2030	5	173	0.0480	1.1707
Senegal	2031	5	178	0.0481	1.1742
Senegal	2032	5	183	0.0482	1.1777
Senegal	2033	5	188	0.0483	1.1812
Senegal	2034	6	193	0.0484	1.1847
Senegal	2035	6	198	0.0484	1.1856
Senegal	2036	6	203	0.0484	1.1865
Senegal	2037	6	208	0.0484	1.1875
Senegal	2038	6	213	0.0484	1.1884

Senegal	2039	7	219	0.0484	1.1894
Senegal	2040	7	224	0.0484	1.1904
Serbia	2022	37	666	0.2497	5.0312
Serbia	2023	37	672	0.2516	5.0602
Serbia	2024	37	680	0.2535	5.0891
Serbia	2025	38	688	0.2548	5.1075
Serbia	2026	39	696	0.2561	5.1254
Serbia	2027	39	704	0.2573	5.1428
Serbia	2028	40	712	0.2584	5.1599
Serbia	2029	41	722	0.2595	5.1766
Serbia	2030	41	730	0.2600	5.1815
Serbia	2031	42	739	0.2605	5.1859
Serbia	2032	43	746	0.2609	5.1901
Serbia	2033	44	753	0.2612	5.1940
Serbia	2034	45	761	0.2616	5.1979
Serbia	2035	45	766	0.2614	5.1912
Serbia	2036	46	770	0.2612	5.1844
Serbia	2037	46	773	0.2609	5.1775
Serbia	2038	47	775	0.2606	5.1705
Serbia	2039	47	777	0.2604	5.1636
Serbia	2040	48	778	0.2601	5.1567
Seychelles	2022	0	1	0.0253	0.6071
Seychelles	2023	0	1	0.0253	0.6064
Seychelles	2024	0	1	0.0253	0.6058
Seychelles	2025	0	1	0.0253	0.6055
Seychelles	2026	0	1	0.0253	0.6051
Seychelles	2027	0	1	0.0253	0.6048
Seychelles	2028	0	1	0.0253	0.6044

Seychelles	2029	0	1	0.0253	0.6040
Seychelles	2030	0	1	0.0253	0.6038
Seychelles	2031	0	1	0.0253	0.6035
Seychelles	2032	0	1	0.0253	0.6032
Seychelles	2033	0	1	0.0253	0.6029
Seychelles	2034	0	1	0.0253	0.6027
Seychelles	2035	0	1	0.0253	0.6024
Seychelles	2036	0	1	0.0253	0.6021
Seychelles	2037	0	1	0.0253	0.6018
Seychelles	2038	0	1	0.0253	0.6015
Seychelles	2039	0	1	0.0253	0.6012
Seychelles	2040	0	1	0.0253	0.6008
Sierra Leone	2022	2	68	0.0439	1.0949
Sierra Leone	2023	2	70	0.0441	1.1005
Sierra Leone	2024	2	72	0.0442	1.1062
Sierra Leone	2025	2	74	0.0444	1.1110
Sierra Leone	2026	2	76	0.0446	1.1158
Sierra Leone	2027	2	78	0.0448	1.1205
Sierra Leone	2028	2	80	0.0449	1.1253
Sierra Leone	2029	2	82	0.0451	1.1301
Sierra Leone	2030	2	83	0.0452	1.1330
Sierra Leone	2031	2	85	0.0453	1.1359
Sierra Leone	2032	2	87	0.0454	1.1388
Sierra Leone	2033	2	89	0.0455	1.1417
Sierra Leone	2034	2	91	0.0456	1.1446
Sierra Leone	2035	2	93	0.0456	1.1456
Sierra Leone	2036	3	95	0.0456	1.1465
Sierra Leone	2037	3	97	0.0456	1.1474

Sierra Leone	2038	3	100	0.0456	1.1484
Sierra Leone	2039	3	102	0.0456	1.1493
Sierra Leone	2040	3	104	0.0457	1.1502
Singapore	2022	19	381	0.2300	5.3889
Singapore	2023	20	396	0.2306	5.3775
Singapore	2024	21	411	0.2311	5.3664
Singapore	2025	23	427	0.2314	5.3537
Singapore	2026	24	444	0.2316	5.3409
Singapore	2027	25	461	0.2319	5.3275
Singapore	2028	27	478	0.2321	5.3133
Singapore	2029	28	496	0.2324	5.2989
Singapore	2030	30	515	0.2323	5.2810
Singapore	2031	32	534	0.2322	5.2632
Singapore	2032	33	553	0.2321	5.2455
Singapore	2033	35	573	0.2320	5.2280
Singapore	2034	37	594	0.2319	5.2103
Singapore	2035	39	615	0.2314	5.1879
Singapore	2036	41	636	0.2309	5.1656
Singapore	2037	44	658	0.2303	5.1430
Singapore	2038	46	679	0.2298	5.1205
Singapore	2039	48	702	0.2293	5.0978
Singapore	2040	51	726	0.2287	5.0751
Slovakia	2022	13	247	0.1465	3.2213
Slovakia	2023	14	251	0.1462	3.2183
Slovakia	2024	14	256	0.1460	3.2153
Slovakia	2025	14	260	0.1458	3.2111
Slovakia	2026	15	265	0.1455	3.2070
Slovakia	2027	15	269	0.1452	3.2027

Slovakia	2028	15	274	0.1449	3.1985
Slovakia	2029	16	279	0.1447	3.1942
Slovakia	2030	16	283	0.1444	3.1889
Slovakia	2031	17	288	0.1441	3.1835
Slovakia	2032	17	293	0.1437	3.1780
Slovakia	2033	18	297	0.1434	3.1726
Slovakia	2034	18	302	0.1431	3.1671
Slovakia	2035	19	306	0.1427	3.1609
Slovakia	2036	19	310	0.1424	3.1546
Slovakia	2037	20	314	0.1420	3.1483
Slovakia	2038	20	317	0.1417	3.1421
Slovakia	2039	20	321	0.1413	3.1358
Slovakia	2040	21	324	0.1409	3.1295
Slovenia	2022	16	256	0.3019	6.2284
Slovenia	2023	16	273	0.3095	6.5517
Slovenia	2024	17	291	0.3171	6.8757
Slovenia	2025	18	307	0.3219	7.1346
Slovenia	2026	19	324	0.3267	7.3937
Slovenia	2027	19	341	0.3314	7.6528
Slovenia	2028	20	358	0.3361	7.9123
Slovenia	2029	21	377	0.3409	8.1725
Slovenia	2030	22	391	0.3422	8.3200
Slovenia	2031	22	406	0.3435	8.4674
Slovenia	2032	23	421	0.3448	8.6146
Slovenia	2033	24	436	0.3461	8.7621
Slovenia	2034	25	452	0.3474	8.9098
Slovenia	2035	25	462	0.3453	8.9207
Slovenia	2036	26	471	0.3431	8.9314

Slovenia	2037	26	481	0.3410	8.9417
Slovenia	2038	27	490	0.3388	8.9519
Slovenia	2039	28	499	0.3366	8.9623
Slovenia	2040	28	509	0.3345	8.9724
Solomon Islands	2022	0	3	0.0242	0.5910
Solomon Islands	2023	0	3	0.0242	0.5922
Solomon Islands	2024	0	3	0.0243	0.5934
Solomon Islands	2025	0	3	0.0243	0.5941
Solomon Islands	2026	0	3	0.0243	0.5948
Solomon Islands	2027	0	3	0.0243	0.5954
Solomon Islands	2028	0	3	0.0244	0.5961
Solomon Islands	2029	0	4	0.0244	0.5968
Solomon Islands	2030	0	4	0.0244	0.5969
Solomon Islands	2031	0	4	0.0244	0.5970
Solomon Islands	2032	0	4	0.0244	0.5971
Solomon Islands	2033	0	4	0.0244	0.5973
Solomon Islands	2034	0	4	0.0244	0.5974
Solomon Islands	2035	0	4	0.0243	0.5970
Solomon Islands	2036	0	4	0.0243	0.5966
Solomon Islands	2037	0	4	0.0243	0.5962
Solomon Islands	2038	0	4	0.0243	0.5959
Solomon Islands	2039	0	4	0.0243	0.5955
Solomon Islands	2040	0	4	0.0242	0.5951
Somalia	2022	2	98	0.0295	0.7211
Somalia	2023	2	100	0.0294	0.7194
Somalia	2024	2	103	0.0294	0.7178
Somalia	2025	2	106	0.0294	0.7174
Somalia	2026	2	109	0.0294	0.7170

Somalia	2027	2	111	0.0294	0.7166
Somalia	2028	3	114	0.0294	0.7162
Somalia	2029	3	117	0.0294	0.7157
Somalia	2030	3	120	0.0294	0.7154
Somalia	2031	3	123	0.0294	0.7151
Somalia	2032	3	126	0.0294	0.7147
Somalia	2033	3	129	0.0294	0.7144
Somalia	2034	3	132	0.0294	0.7140
Somalia	2035	3	136	0.0294	0.7136
Somalia	2036	3	139	0.0294	0.7132
Somalia	2037	3	142	0.0294	0.7128
Somalia	2038	3	146	0.0294	0.7124
Somalia	2039	3	150	0.0294	0.7120
Somalia	2040	3	153	0.0294	0.7116
South Africa	2022	33	1006	0.0709	1.8954
South Africa	2023	34	1030	0.0711	1.9023
South Africa	2024	35	1055	0.0713	1.9092
South Africa	2025	36	1079	0.0715	1.9154
South Africa	2026	37	1103	0.0718	1.9216
South Africa	2027	38	1127	0.0720	1.9278
South Africa	2028	40	1151	0.0722	1.9340
South Africa	2029	41	1175	0.0724	1.9402
South Africa	2030	42	1198	0.0725	1.9438
South Africa	2031	43	1221	0.0726	1.9474
South Africa	2032	44	1244	0.0727	1.9510
South Africa	2033	46	1267	0.0728	1.9547
South Africa	2034	47	1291	0.0729	1.9583
South Africa	2035	48	1313	0.0729	1.9594

South Africa	2036	50	1335	0.0729	1.9606
South Africa	2037	51	1358	0.0729	1.9617
South Africa	2038	52	1380	0.0729	1.9628
South Africa	2039	54	1403	0.0729	1.9639
South Africa	2040	55	1427	0.0729	1.9651
South Sudan	2022	2	79	0.0386	0.9603
South Sudan	2023	2	82	0.0387	0.9631
South Sudan	2024	2	85	0.0388	0.9660
South Sudan	2025	2	89	0.0389	0.9683
South Sudan	2026	2	92	0.0389	0.9706
South Sudan	2027	2	95	0.0390	0.9729
South Sudan	2028	2	99	0.0391	0.9752
South Sudan	2029	2	103	0.0391	0.9776
South Sudan	2030	2	107	0.0392	0.9789
South Sudan	2031	2	111	0.0392	0.9803
South Sudan	2032	3	115	0.0392	0.9816
South Sudan	2033	3	119	0.0392	0.9829
South Sudan	2034	3	124	0.0393	0.9842
South Sudan	2035	3	128	0.0393	0.9845
South Sudan	2036	3	133	0.0393	0.9849
South Sudan	2037	3	138	0.0393	0.9853
South Sudan	2038	3	143	0.0392	0.9856
South Sudan	2039	3	148	0.0392	0.9860
South Sudan	2040	4	153	0.0392	0.9864
Spain	2022	376	5766	0.3170	6.3245
Spain	2023	387	5878	0.3219	6.3396
Spain	2024	399	6000	0.3268	6.3549
Spain	2025	412	6115	0.3305	6.3536

Spain	2026	424	6233	0.3341	6.3523
Spain	2027	437	6349	0.3378	6.3510
Spain	2028	450	6472	0.3414	6.3498
Spain	2029	464	6603	0.3451	6.3486
Spain	2030	477	6721	0.3474	6.3339
Spain	2031	491	6842	0.3496	6.3193
Spain	2032	505	6965	0.3519	6.3046
Spain	2033	520	7093	0.3542	6.2899
Spain	2034	536	7228	0.3565	6.2754
Spain	2035	550	7346	0.3572	6.2476
Spain	2036	565	7465	0.3579	6.2197
Spain	2037	580	7583	0.3586	6.1917
Spain	2038	595	7703	0.3593	6.1638
Spain	2039	611	7827	0.3600	6.1360
Spain	2040	627	7951	0.3607	6.1082
Sri Lanka	2022	6	138	0.0237	0.5696
Sri Lanka	2023	6	142	0.0238	0.5706
Sri Lanka	2024	6	146	0.0238	0.5717
Sri Lanka	2025	7	150	0.0238	0.5723
Sri Lanka	2026	7	153	0.0238	0.5729
Sri Lanka	2027	7	157	0.0238	0.5735
Sri Lanka	2028	8	161	0.0239	0.5741
Sri Lanka	2029	8	165	0.0239	0.5748
Sri Lanka	2030	8	169	0.0239	0.5751
Sri Lanka	2031	9	173	0.0239	0.5753
Sri Lanka	2032	9	176	0.0239	0.5756
Sri Lanka	2033	9	180	0.0239	0.5759
Sri Lanka	2034	10	184	0.0239	0.5762

Sri Lanka	2035	10	187	0.0239	0.5762
Sri Lanka	2036	10	191	0.0239	0.5761
Sri Lanka	2037	11	194	0.0239	0.5761
Sri Lanka	2038	11	198	0.0239	0.5760
Sri Lanka	2039	11	201	0.0239	0.5760
Sri Lanka	2040	11	204	0.0239	0.5759
Sudan	2022	6	261	0.0237	0.6768
Sudan	2023	6	270	0.0240	0.6864
Sudan	2024	6	279	0.0243	0.6959
Sudan	2025	6	287	0.0245	0.7028
Sudan	2026	7	296	0.0247	0.7097
Sudan	2027	7	304	0.0249	0.7165
Sudan	2028	7	312	0.0251	0.7234
Sudan	2029	8	321	0.0253	0.7303
Sudan	2030	8	329	0.0254	0.7342
Sudan	2031	8	337	0.0255	0.7380
Sudan	2032	8	345	0.0255	0.7418
Sudan	2033	9	353	0.0256	0.7456
Sudan	2034	9	361	0.0257	0.7493
Sudan	2035	9	368	0.0257	0.7499
Sudan	2036	10	376	0.0257	0.7504
Sudan	2037	10	383	0.0256	0.7509
Sudan	2038	10	391	0.0256	0.7514
Sudan	2039	11	399	0.0256	0.7519
Sudan	2040	11	408	0.0255	0.7525
Suriname	2022	0	3	0.0121	0.5226
Suriname	2023	0	3	0.0121	0.5257
Suriname	2024	0	3	0.0122	0.5289

Suriname	2025	0	3	0.0122	0.5310
Suriname	2026	0	3	0.0123	0.5332
Suriname	2027	0	3	0.0123	0.5353
Suriname	2028	0	3	0.0124	0.5374
Suriname	2029	0	3	0.0124	0.5395
Suriname	2030	0	3	0.0124	0.5407
Suriname	2031	0	3	0.0124	0.5419
Suriname	2032	0	3	0.0125	0.5431
Suriname	2033	0	3	0.0125	0.5443
Suriname	2034	0	3	0.0125	0.5455
Suriname	2035	0	3	0.0125	0.5459
Suriname	2036	0	3	0.0125	0.5464
Suriname	2037	0	3	0.0125	0.5468
Suriname	2038	0	3	0.0125	0.5472
Suriname	2039	0	3	0.0125	0.5476
Suriname	2040	0	3	0.0125	0.5481
Sweden	2022	165	2613	0.6391	12.2977
Sweden	2023	165	2592	0.6196	11.9221
Sweden	2024	166	2570	0.6001	11.5458
Sweden	2025	167	2569	0.5889	11.3411
Sweden	2026	168	2567	0.5777	11.1364
Sweden	2027	169	2562	0.5666	10.9320
Sweden	2028	170	2555	0.5554	10.7276
Sweden	2029	171	2545	0.5443	10.5231
Sweden	2030	173	2557	0.5389	10.4317
Sweden	2031	175	2567	0.5335	10.3406
Sweden	2032	176	2576	0.5282	10.2496
Sweden	2033	178	2584	0.5228	10.1586

Sweden	2034	179	2590	0.5175	10.0675
Sweden	2035	181	2617	0.5167	10.0718
Sweden	2036	184	2644	0.5160	10.0762
Sweden	2037	186	2669	0.5153	10.0807
Sweden	2038	188	2694	0.5146	10.0853
Sweden	2039	189	2719	0.5139	10.0899
Sweden	2040	191	2744	0.5132	10.0945
Switzerland	2022	63	984	0.2959	5.9925
Switzerland	2023	65	1010	0.2973	6.0338
Switzerland	2024	67	1038	0.2988	6.0751
Switzerland	2025	69	1068	0.3000	6.1086
Switzerland	2026	71	1099	0.3012	6.1420
Switzerland	2027	73	1129	0.3023	6.1751
Switzerland	2028	76	1161	0.3035	6.2082
Switzerland	2029	78	1193	0.3047	6.2413
Switzerland	2030	80	1226	0.3057	6.2691
Switzerland	2031	83	1258	0.3067	6.2968
Switzerland	2032	85	1290	0.3076	6.3244
Switzerland	2033	88	1321	0.3086	6.3519
Switzerland	2034	90	1353	0.3095	6.3793
Switzerland	2035	93	1384	0.3102	6.3996
Switzerland	2036	95	1414	0.3110	6.4199
Switzerland	2037	98	1443	0.3117	6.4401
Switzerland	2038	100	1472	0.3124	6.4603
Switzerland	2039	103	1500	0.3131	6.4805
Switzerland	2040	105	1529	0.3138	6.5006
Syrian Arab Republic	2022	3	102	0.0245	0.6452
Syrian Arab Republic	2023	3	104	0.0247	0.6521

Syrian Arab Republic	2024	3	106	0.0250	0.6591
Syrian Arab Republic	2025	4	108	0.0251	0.6641
Syrian Arab Republic	2026	4	111	0.0253	0.6692
Syrian Arab Republic	2027	4	113	0.0254	0.6742
Syrian Arab Republic	2028	4	117	0.0256	0.6793
Syrian Arab Republic	2029	4	120	0.0258	0.6844
Syrian Arab Republic	2030	4	123	0.0258	0.6871
Syrian Arab Republic	2031	5	127	0.0259	0.6898
Syrian Arab Republic	2032	5	130	0.0260	0.6926
Syrian Arab Republic	2033	5	134	0.0260	0.6953
Syrian Arab Republic	2034	5	138	0.0261	0.6980
Syrian Arab Republic	2035	6	141	0.0261	0.6982
Syrian Arab Republic	2036	6	145	0.0261	0.6984
Syrian Arab Republic	2037	6	149	0.0261	0.6986
Syrian Arab Republic	2038	6	152	0.0260	0.6988
Syrian Arab Republic	2039	7	156	0.0260	0.6989
Syrian Arab Republic	2040	7	159	0.0260	0.6992
Taiwan (Province of China)	2022	50	934	0.1167	2.5897
Taiwan (Province of China)	2023	51	963	0.1175	2.6076
Taiwan (Province of China)	2024	53	994	0.1184	2.6256
Taiwan (Province of China)	2025	55	1021	0.1187	2.6338
Taiwan (Province of China)	2026	57	1049	0.1189	2.6420
Taiwan (Province of China)	2027	59	1076	0.1192	2.6504
Taiwan (Province of China)	2028	61	1104	0.1196	2.6588
Taiwan (Province of China)	2029	63	1133	0.1199	2.6674
Taiwan (Province of China)	2030	65	1158	0.1197	2.6661
Taiwan (Province of China)	2031	67	1183	0.1196	2.6648
Taiwan (Province of China)	2032	69	1208	0.1194	2.6635

Taiwan (Province of China)	2033	71	1232	0.1193	2.6623
Taiwan (Province of China)	2034	74	1258	0.1192	2.6611
Taiwan (Province of China)	2035	76	1279	0.1186	2.6502
Taiwan (Province of China)	2036	78	1300	0.1180	2.6393
Taiwan (Province of China)	2037	80	1319	0.1174	2.6284
Taiwan (Province of China)	2038	82	1337	0.1168	2.6175
Taiwan (Province of China)	2039	85	1356	0.1162	2.6067
Taiwan (Province of China)	2040	87	1375	0.1156	2.5959
Tajikistan	2022	3	99	0.0540	1.2577
Tajikistan	2023	3	102	0.0542	1.2619
Tajikistan	2024	3	105	0.0543	1.2663
Tajikistan	2025	3	108	0.0545	1.2699
Tajikistan	2026	3	111	0.0546	1.2736
Tajikistan	2027	3	114	0.0548	1.2771
Tajikistan	2028	4	117	0.0549	1.2806
Tajikistan	2029	4	121	0.0550	1.2841
Tajikistan	2030	4	124	0.0551	1.2853
Tajikistan	2031	4	127	0.0551	1.2866
Tajikistan	2032	4	130	0.0551	1.2878
Tajikistan	2033	4	134	0.0551	1.2889
Tajikistan	2034	5	138	0.0551	1.2899
Tajikistan	2035	5	141	0.0550	1.2886
Tajikistan	2036	5	145	0.0549	1.2873
Tajikistan	2037	5	149	0.0548	1.2860
Tajikistan	2038	5	153	0.0547	1.2846
Tajikistan	2039	6	157	0.0546	1.2833
Tajikistan	2040	6	161	0.0545	1.2820
Thailand	2022	24	504	0.0225	0.5673

Thailand	2023	25	518	0.0225	0.5676
Thailand	2024	26	534	0.0225	0.5679
Thailand	2025	28	549	0.0225	0.5680
Thailand	2026	29	565	0.0225	0.5682
Thailand	2027	30	581	0.0225	0.5684
Thailand	2028	31	597	0.0225	0.5686
Thailand	2029	33	614	0.0225	0.5688
Thailand	2030	34	631	0.0225	0.5690
Thailand	2031	36	648	0.0225	0.5691
Thailand	2032	37	665	0.0225	0.5693
Thailand	2033	39	682	0.0225	0.5694
Thailand	2034	40	700	0.0225	0.5696
Thailand	2035	42	718	0.0225	0.5697
Thailand	2036	44	735	0.0225	0.5698
Thailand	2037	45	752	0.0225	0.5699
Thailand	2038	47	769	0.0225	0.5700
Thailand	2039	49	786	0.0225	0.5701
Thailand	2040	51	804	0.0225	0.5702
Timor-Leste	2022	0	6	0.0201	0.5338
Timor-Leste	2023	0	7	0.0202	0.5366
Timor-Leste	2024	0	7	0.0203	0.5394
Timor-Leste	2025	0	7	0.0204	0.5413
Timor-Leste	2026	0	7	0.0204	0.5432
Timor-Leste	2027	0	7	0.0205	0.5451
Timor-Leste	2028	0	7	0.0205	0.5469
Timor-Leste	2029	0	8	0.0206	0.5488
Timor-Leste	2030	0	8	0.0206	0.5496
Timor-Leste	2031	0	8	0.0206	0.5505

Timor-Leste	2032	0	8	0.0206	0.5514
Timor-Leste	2033	0	8	0.0206	0.5523
Timor-Leste	2034	0	8	0.0206	0.5532
Timor-Leste	2035	0	9	0.0206	0.5532
Timor-Leste	2036	0	9	0.0205	0.5533
Timor-Leste	2037	0	9	0.0205	0.5533
Timor-Leste	2038	0	9	0.0205	0.5534
Timor-Leste	2039	0	9	0.0205	0.5534
Timor-Leste	2040	0	9	0.0205	0.5535
Togo	2022	2	70	0.0476	1.1809
Togo	2023	2	72	0.0478	1.1875
Togo	2024	2	74	0.0480	1.1940
Togo	2025	2	76	0.0482	1.1996
Togo	2026	2	78	0.0484	1.2052
Togo	2027	2	80	0.0486	1.2108
Togo	2028	2	82	0.0488	1.2163
Togo	2029	2	85	0.0490	1.2219
Togo	2030	2	87	0.0491	1.2253
Togo	2031	3	89	0.0492	1.2287
Togo	2032	3	91	0.0493	1.2320
Togo	2033	3	94	0.0495	1.2354
Togo	2034	3	96	0.0496	1.2387
Togo	2035	3	98	0.0496	1.2398
Togo	2036	3	101	0.0496	1.2408
Togo	2037	3	103	0.0496	1.2419
Togo	2038	3	106	0.0497	1.2429
Togo	2039	3	108	0.0497	1.2439
Togo	2040	4	111	0.0497	1.2449

Tonga	2022	0	1	0.0296	0.7020
Tonga	2023	0	1	0.0297	0.7047
Tonga	2024	0	1	0.0298	0.7075
Tonga	2025	0	1	0.0299	0.7095
Tonga	2026	0	1	0.0300	0.7115
Tonga	2027	0	1	0.0301	0.7135
Tonga	2028	0	1	0.0301	0.7155
Tonga	2029	0	1	0.0302	0.7175
Tonga	2030	0	1	0.0302	0.7186
Tonga	2031	0	1	0.0303	0.7196
Tonga	2032	0	1	0.0303	0.7207
Tonga	2033	0	1	0.0303	0.7218
Tonga	2034	0	1	0.0304	0.7229
Tonga	2035	0	1	0.0304	0.7230
Tonga	2036	0	1	0.0304	0.7231
Tonga	2037	0	1	0.0304	0.7233
Tonga	2038	0	1	0.0304	0.7234
Tonga	2039	0	1	0.0304	0.7236
Tonga	2040	0	1	0.0304	0.7237
Trinidad and Tobago	2022	0	8	0.0146	0.5795
Trinidad and Tobago	2023	0	8	0.0147	0.5840
Trinidad and Tobago	2024	0	8	0.0147	0.5885
Trinidad and Tobago	2025	0	8	0.0148	0.5915
Trinidad and Tobago	2026	0	8	0.0149	0.5944
Trinidad and Tobago	2027	0	8	0.0149	0.5974
Trinidad and Tobago	2028	0	8	0.0150	0.6004
Trinidad and Tobago	2029	0	9	0.0151	0.6034
Trinidad and Tobago	2030	0	9	0.0151	0.6052

Trinidad and Tobago	2031	0	9	0.0151	0.6071
Trinidad and Tobago	2032	0	9	0.0151	0.6090
Trinidad and Tobago	2033	0	9	0.0152	0.6109
Trinidad and Tobago	2034	0	9	0.0152	0.6128
Trinidad and Tobago	2035	0	9	0.0152	0.6136
Trinidad and Tobago	2036	0	9	0.0152	0.6145
Trinidad and Tobago	2037	0	9	0.0152	0.6153
Trinidad and Tobago	2038	0	9	0.0152	0.6162
Trinidad and Tobago	2039	0	9	0.0152	0.6170
Trinidad and Tobago	2040	0	9	0.0152	0.6179
Tunisia	2022	3	78	0.0250	0.6465
Tunisia	2023	3	80	0.0252	0.6525
Tunisia	2024	3	82	0.0254	0.6586
Tunisia	2025	3	85	0.0256	0.6628
Tunisia	2026	3	87	0.0257	0.6670
Tunisia	2027	4	89	0.0259	0.6712
Tunisia	2028	4	91	0.0260	0.6755
Tunisia	2029	4	94	0.0261	0.6798
Tunisia	2030	4	96	0.0262	0.6821
Tunisia	2031	4	98	0.0263	0.6844
Tunisia	2032	4	101	0.0263	0.6867
Tunisia	2033	4	103	0.0264	0.6890
Tunisia	2034	5	106	0.0265	0.6913
Tunisia	2035	5	108	0.0265	0.6914
Tunisia	2036	5	110	0.0264	0.6916
Tunisia	2037	5	112	0.0264	0.6917
Tunisia	2038	5	115	0.0264	0.6918
Tunisia	2039	6	117	0.0264	0.6920

Tunisia	2040	6	120	0.0264	0.6921
Turkey	2022	22	534	0.0213	0.5822
Turkey	2023	24	555	0.0214	0.5912
Turkey	2024	25	578	0.0215	0.6003
Turkey	2025	26	600	0.0216	0.6073
Turkey	2026	27	622	0.0216	0.6144
Turkey	2027	28	644	0.0217	0.6215
Turkey	2028	30	667	0.0218	0.6286
Turkey	2029	31	691	0.0219	0.6358
Turkey	2030	32	712	0.0219	0.6401
Turkey	2031	33	735	0.0219	0.6444
Turkey	2032	35	757	0.0219	0.6488
Turkey	2033	36	781	0.0220	0.6532
Turkey	2034	38	805	0.0220	0.6576
Turkey	2035	39	827	0.0220	0.6587
Turkey	2036	41	849	0.0219	0.6599
Turkey	2037	42	871	0.0219	0.6610
Turkey	2038	44	894	0.0219	0.6621
Turkey	2039	46	917	0.0218	0.6633
Turkey	2040	48	942	0.0218	0.6645
Turkmenistan	2022	2	71	0.0600	1.5434
Turkmenistan	2023	2	73	0.0607	1.5613
Turkmenistan	2024	2	75	0.0614	1.5790
Turkmenistan	2025	2	78	0.0619	1.5903
Turkmenistan	2026	3	80	0.0623	1.6016
Turkmenistan	2027	3	82	0.0627	1.6128
Turkmenistan	2028	3	84	0.0631	1.6238
Turkmenistan	2029	3	86	0.0635	1.6348

Turkmenistan	2030	3	89	0.0637	1.6418
Turkmenistan	2031	3	91	0.0639	1.6487
Turkmenistan	2032	3	93	0.0642	1.6556
Turkmenistan	2033	3	96	0.0644	1.6625
Turkmenistan	2034	4	98	0.0646	1.6692
Turkmenistan	2035	4	101	0.0647	1.6723
Turkmenistan	2036	4	103	0.0647	1.6753
Turkmenistan	2037	4	106	0.0648	1.6782
Turkmenistan	2038	4	108	0.0649	1.6812
Turkmenistan	2039	4	111	0.0649	1.6841
Turkmenistan	2040	5	114	0.0650	1.6871
Uganda	2022	9	399	0.0487	1.2432
Uganda	2023	9	417	0.0492	1.2582
Uganda	2024	10	437	0.0497	1.2733
Uganda	2025	10	456	0.0501	1.2850
Uganda	2026	11	475	0.0505	1.2967
Uganda	2027	11	495	0.0508	1.3085
Uganda	2028	12	515	0.0512	1.3203
Uganda	2029	12	536	0.0516	1.3321
Uganda	2030	13	555	0.0518	1.3396
Uganda	2031	13	575	0.0521	1.3471
Uganda	2032	14	595	0.0523	1.3545
Uganda	2033	14	615	0.0525	1.3620
Uganda	2034	15	635	0.0527	1.3695
Uganda	2035	15	654	0.0528	1.3725
Uganda	2036	16	673	0.0529	1.3756
Uganda	2037	17	692	0.0529	1.3786
Uganda	2038	17	712	0.0530	1.3816

Uganda	2039	18	731	0.0531	1.3847
Uganda	2040	19	752	0.0531	1.3877
Ukraine	2022	29	909	0.0459	1.8176
Ukraine	2023	30	938	0.0475	1.8799
Ukraine	2024	30	967	0.0491	1.9421
Ukraine	2025	32	992	0.0510	1.9963
Ukraine	2026	33	1018	0.0529	2.0504
Ukraine	2027	34	1043	0.0548	2.1045
Ukraine	2028	36	1068	0.0567	2.1587
Ukraine	2029	37	1093	0.0586	2.2127
Ukraine	2030	39	1111	0.0608	2.2526
Ukraine	2031	40	1129	0.0630	2.2926
Ukraine	2032	42	1146	0.0652	2.3325
Ukraine	2033	43	1163	0.0674	2.3724
Ukraine	2034	44	1178	0.0695	2.4122
Ukraine	2035	46	1184	0.0718	2.4321
Ukraine	2036	47	1189	0.0741	2.4519
Ukraine	2037	49	1195	0.0764	2.4717
Ukraine	2038	50	1200	0.0787	2.4915
Ukraine	2039	51	1206	0.0809	2.5112
Ukraine	2040	53	1211	0.0832	2.5309
United Arab Emirates	2022	2	74	0.0351	0.9070
United Arab Emirates	2023	2	76	0.0354	0.9138
United Arab Emirates	2024	2	78	0.0357	0.9206
United Arab Emirates	2025	2	79	0.0359	0.9250
United Arab Emirates	2026	2	81	0.0361	0.9293
United Arab Emirates	2027	2	83	0.0363	0.9335
United Arab Emirates	2028	2	85	0.0365	0.9381

United Arab Emirates	2029	2	87	0.0367	0.9429
United Arab Emirates	2030	3	89	0.0369	0.9452
United Arab Emirates	2031	3	91	0.0370	0.9472
United Arab Emirates	2032	3	93	0.0371	0.9494
United Arab Emirates	2033	3	96	0.0373	0.9519
United Arab Emirates	2034	3	99	0.0375	0.9547
United Arab Emirates	2035	3	101	0.0375	0.9546
United Arab Emirates	2036	4	104	0.0376	0.9543
United Arab Emirates	2037	4	107	0.0376	0.9539
United Arab Emirates	2038	4	111	0.0377	0.9539
United Arab Emirates	2039	4	114	0.0377	0.9539
United Arab Emirates	2040	4	118	0.0378	0.9537
United Kingdom	2022	413	6174	0.2710	4.7965
United Kingdom	2023	425	6304	0.2738	4.8119
United Kingdom	2024	437	6447	0.2767	4.8273
United Kingdom	2025	451	6598	0.2790	4.8378
United Kingdom	2026	464	6752	0.2812	4.8481
United Kingdom	2027	477	6906	0.2835	4.8583
United Kingdom	2028	491	7066	0.2858	4.8684
United Kingdom	2029	507	7238	0.2881	4.8785
United Kingdom	2030	522	7406	0.2896	4.8823
United Kingdom	2031	536	7574	0.2911	4.8860
United Kingdom	2032	551	7735	0.2927	4.8897
United Kingdom	2033	565	7897	0.2942	4.8933
United Kingdom	2034	580	8064	0.2957	4.8969
United Kingdom	2035	594	8222	0.2966	4.8931
United Kingdom	2036	608	8375	0.2975	4.8892
United Kingdom	2037	621	8523	0.2983	4.8853

United Kingdom	2038	634	8667	0.2992	4.8814
United Kingdom	2039	648	8813	0.3000	4.8774
United Kingdom	2040	661	8959	0.3009	4.8734
United Republic of Tanzania	2022	14	559	0.0492	1.2269
United Republic of Tanzania	2023	15	581	0.0495	1.2370
United Republic of Tanzania	2024	16	604	0.0498	1.2470
United Republic of Tanzania	2025	16	627	0.0501	1.2547
United Republic of Tanzania	2026	17	650	0.0504	1.2624
United Republic of Tanzania	2027	18	674	0.0506	1.2701
United Republic of Tanzania	2028	18	699	0.0509	1.2778
United Republic of Tanzania	2029	19	724	0.0511	1.2855
United Republic of Tanzania	2030	20	748	0.0513	1.2901
United Republic of Tanzania	2031	21	773	0.0515	1.2948
United Republic of Tanzania	2032	21	799	0.0516	1.2996
United Republic of Tanzania	2033	22	825	0.0518	1.3043
United Republic of Tanzania	2034	23	852	0.0519	1.3090
United Republic of Tanzania	2035	24	877	0.0519	1.3106
United Republic of Tanzania	2036	25	903	0.0520	1.3122
United Republic of Tanzania	2037	26	930	0.0520	1.3139
United Republic of Tanzania	2038	27	957	0.0521	1.3155
United Republic of Tanzania	2039	28	986	0.0521	1.3172
United Republic of Tanzania	2040	29	1015	0.0521	1.3189
United States of America	2022	7519	130573	1.1831	22.8564
United States of America	2023	7584	131129	1.1610	22.3905
United States of America	2024	7662	131738	1.1389	21.9245
United States of America	2025	7790	133215	1.1244	21.6365
United States of America	2026	7917	134597	1.1099	21.3486
United States of America	2027	8036	135814	1.0955	21.0607

United States of America	2028	8157	136935	1.0810	20.7728
United States of America	2029	8285	138014	1.0666	20.4848
United States of America	2030	8469	140024	1.0595	20.3487
United States of America	2031	8648	141894	1.0525	20.2126
United States of America	2032	8813	143564	1.0455	20.0766
United States of America	2033	8972	145078	1.0384	19.9407
United States of America	2034	9130	146480	1.0314	19.8047
United States of America	2035	9342	148708	1.0310	19.7958
United States of America	2036	9542	150778	1.0306	19.7870
United States of America	2037	9724	152656	1.0301	19.7782
United States of America	2038	9894	154369	1.0297	19.7695
United States of America	2039	10058	155952	1.0293	19.7608
United States of America	2040	10209	157387	1.0289	19.7522
United States Virgin Islands	2022	0	1	0.0155	0.5795
United States Virgin Islands	2023	0	1	0.0156	0.5871
United States Virgin Islands	2024	0	1	0.0158	0.5947
United States Virgin Islands	2025	0	1	0.0158	0.5982
United States Virgin Islands	2026	0	1	0.0159	0.6017
United States Virgin Islands	2027	0	1	0.0160	0.6052
United States Virgin Islands	2028	0	1	0.0160	0.6087
United States Virgin Islands	2029	0	1	0.0161	0.6122
United States Virgin Islands	2030	0	1	0.0161	0.6142
United States Virgin Islands	2031	0	1	0.0162	0.6161
United States Virgin Islands	2032	0	1	0.0162	0.6181
United States Virgin Islands	2033	0	1	0.0162	0.6201
United States Virgin Islands	2034	0	1	0.0163	0.6222
United States Virgin Islands	2035	0	1	0.0163	0.6228
United States Virgin Islands	2036	0	1	0.0162	0.6235

United States Virgin Islands	2037	0	1	0.0162	0.6242
United States Virgin Islands	2038	0	1	0.0162	0.6249
United States Virgin Islands	2039	0	1	0.0162	0.6256
United States Virgin Islands	2040	0	1	0.0162	0.6264
Uruguay	2022	7	120	0.1071	2.4635
Uruguay	2023	7	123	0.1079	2.4860
Uruguay	2024	7	125	0.1087	2.5086
Uruguay	2025	7	127	0.1093	2.5245
Uruguay	2026	7	129	0.1098	2.5405
Uruguay	2027	7	131	0.1103	2.5564
Uruguay	2028	7	133	0.1109	2.5723
Uruguay	2029	7	136	0.1114	2.5883
Uruguay	2030	8	138	0.1116	2.5973
Uruguay	2031	8	139	0.1119	2.6063
Uruguay	2032	8	141	0.1121	2.6152
Uruguay	2033	8	143	0.1123	2.6242
Uruguay	2034	8	145	0.1125	2.6332
Uruguay	2035	8	147	0.1125	2.6351
Uruguay	2036	8	149	0.1124	2.6370
Uruguay	2037	9	151	0.1124	2.6389
Uruguay	2038	9	152	0.1123	2.6408
Uruguay	2039	9	154	0.1122	2.6426
Uruguay	2040	9	156	0.1122	2.6445
Uzbekistan	2022	10	381	0.0520	1.3753
Uzbekistan	2023	11	392	0.0526	1.3915
Uzbekistan	2024	11	403	0.0533	1.4080
Uzbekistan	2025	11	414	0.0537	1.4174
Uzbekistan	2026	12	425	0.0540	1.4268

Uzbekistan	2027	12	437	0.0543	1.4362
Uzbekistan	2028	13	448	0.0546	1.4458
Uzbekistan	2029	13	460	0.0549	1.4554
Uzbekistan	2030	14	471	0.0552	1.4625
Uzbekistan	2031	14	483	0.0555	1.4696
Uzbekistan	2032	15	494	0.0557	1.4768
Uzbekistan	2033	15	506	0.0560	1.4840
Uzbekistan	2034	16	518	0.0563	1.4912
Uzbekistan	2035	17	529	0.0565	1.4955
Uzbekistan	2036	17	540	0.0567	1.4998
Uzbekistan	2037	18	551	0.0568	1.5040
Uzbekistan	2038	18	562	0.0570	1.5083
Uzbekistan	2039	19	573	0.0572	1.5127
Uzbekistan	2040	20	585	0.0574	1.5170
Vanuatu	2022	0	2	0.0258	0.6296
Vanuatu	2023	0	2	0.0259	0.6308
Vanuatu	2024	0	2	0.0259	0.6321
Vanuatu	2025	0	2	0.0259	0.6329
Vanuatu	2026	0	2	0.0259	0.6337
Vanuatu	2027	0	2	0.0260	0.6345
Vanuatu	2028	0	2	0.0260	0.6353
Vanuatu	2029	0	2	0.0260	0.6361
Vanuatu	2030	0	2	0.0260	0.6363
Vanuatu	2031	0	2	0.0260	0.6365
Vanuatu	2032	0	2	0.0260	0.6366
Vanuatu	2033	0	2	0.0260	0.6369
Vanuatu	2034	0	2	0.0260	0.6371
Vanuatu	2035	0	2	0.0259	0.6367

Vanuatu	2036	0	2	0.0259	0.6362
Vanuatu	2037	0	2	0.0259	0.6359
Vanuatu	2038	0	2	0.0258	0.6355
Vanuatu	2039	0	2	0.0258	0.6351
Vanuatu	2040	0	2	0.0258	0.6348
Venezuela (Bolivarian Republic of)	2022	10	374	0.0336	1.2063
Venezuela (Bolivarian Republic of)	2023	11	382	0.0337	1.2130
Venezuela (Bolivarian Republic of)	2024	11	390	0.0338	1.2198
Venezuela (Bolivarian Republic of)	2025	11	396	0.0339	1.2239
Venezuela (Bolivarian Republic of)	2026	12	403	0.0340	1.2281
Venezuela (Bolivarian Republic of)	2027	12	409	0.0341	1.2322
Venezuela (Bolivarian Republic of)	2028	13	416	0.0341	1.2363
Venezuela (Bolivarian Republic of)	2029	13	422	0.0342	1.2405
Venezuela (Bolivarian Republic of)	2030	13	428	0.0343	1.2429
Venezuela (Bolivarian Republic of)	2031	14	434	0.0343	1.2453
Venezuela (Bolivarian Republic of)	2032	14	440	0.0343	1.2478
Venezuela (Bolivarian Republic of)	2033	15	447	0.0344	1.2502
Venezuela (Bolivarian Republic of)	2034	15	453	0.0344	1.2527
Venezuela (Bolivarian Republic of)	2035	15	459	0.0344	1.2538
Venezuela (Bolivarian Republic of)	2036	16	465	0.0344	1.2548
Venezuela (Bolivarian Republic of)	2037	16	471	0.0344	1.2559
Venezuela (Bolivarian Republic of)	2038	17	477	0.0344	1.2570
Venezuela (Bolivarian Republic of)	2039	17	483	0.0344	1.2581
Venezuela (Bolivarian Republic of)	2040	18	489	0.0344	1.2592
Viet Nam	2022	20	514	0.0221	0.5430
Viet Nam	2023	21	527	0.0222	0.5456
Viet Nam	2024	21	541	0.0223	0.5482
Viet Nam	2025	22	555	0.0224	0.5499

Viet Nam	2026	23	569	0.0225	0.5516
Viet Nam	2027	24	583	0.0225	0.5533
Viet Nam	2028	24	598	0.0226	0.5550
Viet Nam	2029	25	614	0.0226	0.5567
Viet Nam	2030	26	629	0.0227	0.5578
Viet Nam	2031	27	645	0.0227	0.5588
Viet Nam	2032	28	661	0.0227	0.5599
Viet Nam	2033	30	677	0.0227	0.5609
Viet Nam	2034	31	694	0.0228	0.5620
Viet Nam	2035	32	711	0.0228	0.5623
Viet Nam	2036	33	728	0.0228	0.5626
Viet Nam	2037	34	744	0.0228	0.5629
Viet Nam	2038	36	761	0.0228	0.5632
Viet Nam	2039	37	778	0.0228	0.5635
Viet Nam	2040	39	795	0.0228	0.5638
Yemen	2022	4	185	0.0217	0.6311
Yemen	2023	4	192	0.0220	0.6405
Yemen	2024	4	199	0.0223	0.6501
Yemen	2025	4	205	0.0225	0.6566
Yemen	2026	4	211	0.0227	0.6631
Yemen	2027	5	216	0.0229	0.6695
Yemen	2028	5	222	0.0230	0.6760
Yemen	2029	5	228	0.0232	0.6825
Yemen	2030	5	234	0.0233	0.6862
Yemen	2031	5	239	0.0234	0.6900
Yemen	2032	5	244	0.0235	0.6937
Yemen	2033	5	249	0.0235	0.6974
Yemen	2034	6	255	0.0236	0.7011

Yemen	2035	6	259	0.0236	0.7020
Yemen	2036	6	264	0.0236	0.7029
Yemen	2037	6	268	0.0235	0.7037
Yemen	2038	6	272	0.0235	0.7046
Yemen	2039	6	277	0.0235	0.7054
Yemen	2040	7	282	0.0235	0.7063
Zambia	2022	5	206	0.0561	1.4630
Zambia	2023	5	215	0.0566	1.4778
Zambia	2024	5	223	0.0571	1.4927
Zambia	2025	5	232	0.0574	1.5039
Zambia	2026	5	240	0.0578	1.5152
Zambia	2027	6	249	0.0582	1.5264
Zambia	2028	6	258	0.0586	1.5377
Zambia	2029	6	267	0.0589	1.5490
Zambia	2030	6	276	0.0592	1.5560
Zambia	2031	7	284	0.0594	1.5630
Zambia	2032	7	293	0.0596	1.5700
Zambia	2033	7	303	0.0598	1.5771
Zambia	2034	8	312	0.0600	1.5841
Zambia	2035	8	321	0.0601	1.5867
Zambia	2036	8	330	0.0602	1.5892
Zambia	2037	8	339	0.0602	1.5917
Zambia	2038	9	348	0.0603	1.5943
Zambia	2039	9	358	0.0603	1.5968
Zambia	2040	9	368	0.0604	1.5993
Zimbabwe	2022	4	150	0.0522	1.2861
Zimbabwe	2023	4	153	0.0520	1.2797
Zimbabwe	2024	4	156	0.0519	1.2735

Zimbabwe	2025	4	159	0.0518	1.2715
Zimbabwe	2026	4	162	0.0518	1.2697
Zimbabwe	2027	4	166	0.0518	1.2678
Zimbabwe	2028	4	169	0.0518	1.2660
Zimbabwe	2029	5	172	0.0518	1.2643
Zimbabwe	2030	5	176	0.0518	1.2634
Zimbabwe	2031	5	179	0.0518	1.2625
Zimbabwe	2032	5	183	0.0518	1.2617
Zimbabwe	2033	5	186	0.0518	1.2608
Zimbabwe	2034	5	190	0.0518	1.2601
Zimbabwe	2035	5	194	0.0518	1.2594
Zimbabwe	2036	5	198	0.0518	1.2588
Zimbabwe	2037	6	202	0.0518	1.2582
Zimbabwe	2038	6	206	0.0518	1.2575
Zimbabwe	2039	6	210	0.0518	1.2569
Zimbabwe	2040	6	214	0.0518	1.2562

Abbreviations: CDI, *Clostridioides difficile* infections; ASMRs, age-standardized mortality rates; ASDRs, age-standardized DALY rates; DALY, disability-adjusted life-year.

Figure S1: Global age- and sex-specific CDI-related deaths and DALYs in 2021, and their AAPC from 1990 to 2021.

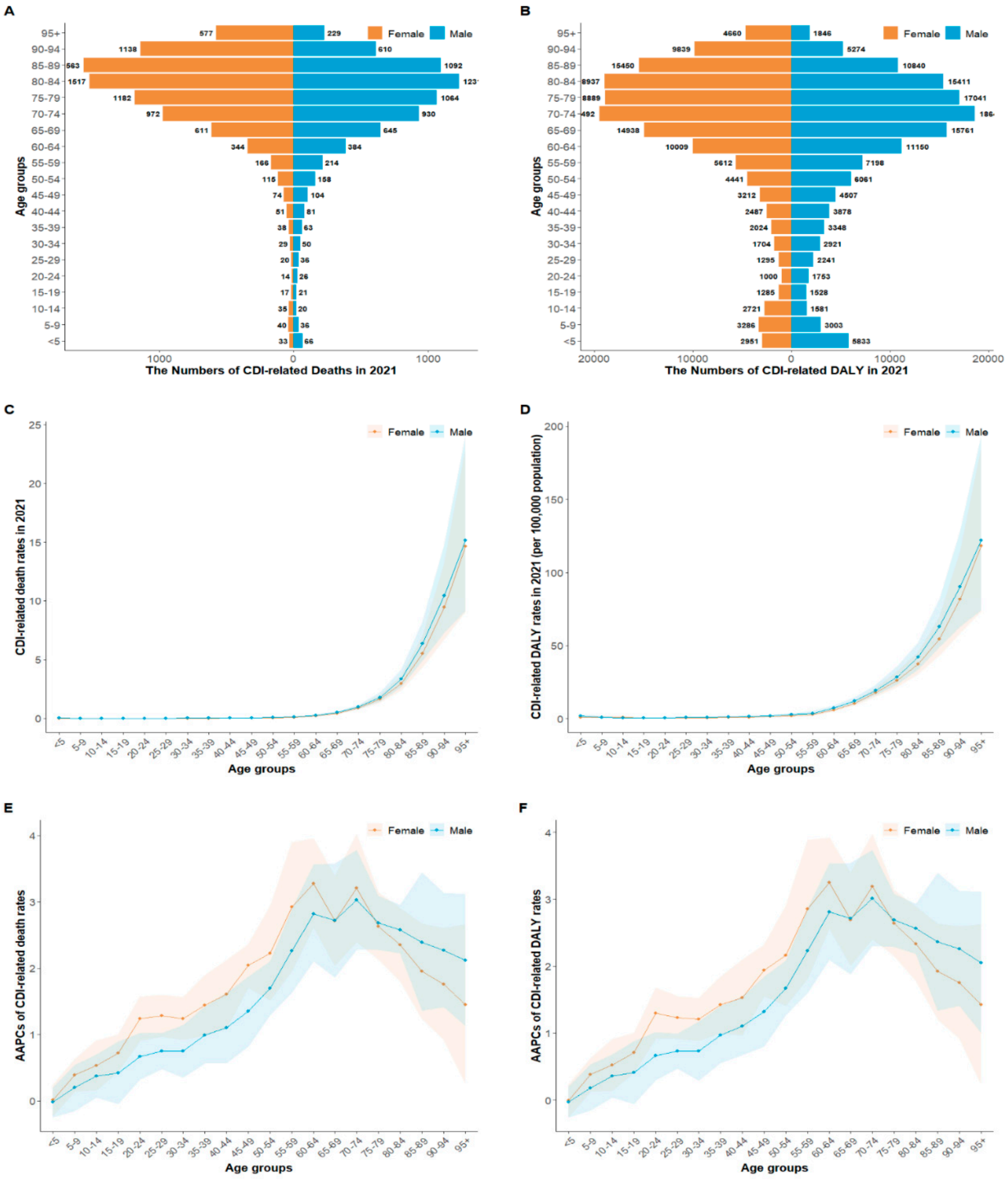

(A) CDI-related deaths number globally in 2021, by age and sex. (B) CDI-related DALYs number globally in 2021, by age and sex. (C) CDI-related deaths rates globally in 2021, by age and sex. (D) CDI-related DALYs rates globally in 2021, by age and sex. (E) Global annual changes of CDI-related death rates from 1990 to 2021, by age and sex. (F) Global annual changes of CDI-related DALY rates from 1990 to 2021, by age and sex. Shaded areas indicate the 95%UI or 95% CI. Abbreviations: DALY, disability-adjusted life-year; ASR, age-standardized rate; UI, uncertainty interval; AAPC, average annual percent change; CI, confidence interval.
